# Identification of Potential Pathway Mediation Targets in Toll-like Receptor Signaling

**DOI:** 10.1371/journal.pcbi.1000292

**Published:** 2009-02-20

**Authors:** Fan Li, Ines Thiele, Neema Jamshidi, Bernhard Ø. Palsson

**Affiliations:** 1 Department of Bioengineering, University of California San Diego, La Jolla, California, United States of America; 2 Ph.D. Program in Bioinformatics, University of California San Diego, La Jolla, California, United States of America; University of Illinois at Urbana-Champaign, United States of America

## Abstract

Recent advances in reconstruction and analytical methods for signaling networks have spurred the development of large-scale models that incorporate fully functional and biologically relevant features. An extended reconstruction of the human Toll-like receptor signaling network is presented herein. This reconstruction contains an extensive complement of kinases, phosphatases, and other associated proteins that mediate the signaling cascade along with a delineation of their associated chemical reactions. A computational framework based on the methods of large-scale convex analysis was developed and applied to this network to characterize input–output relationships. The input–output relationships enabled significant modularization of the network into ten pathways. The analysis identified potential candidates for inhibitory mediation of TLR signaling with respect to their specificity and potency. Subsequently, we were able to identify eight novel inhibition targets through constraint-based modeling methods. The results of this study are expected to yield meaningful avenues for further research in the task of mediating the Toll-like receptor signaling network and its effects.

## Author Summary

The human innate immune system, as the first line of defense against pathogens, is a vital component of our survival. One component of the innate immune system is the Toll-like receptor signaling network, which is responsible for transmitting activation signals from the outside of the cell to molecular machinery inside the cell. The innate immune system must be properly balanced, as excessive activation can lead to potentially lethal septic shock. Therefore, there is much interest in developing drugs that can mediate Toll-like receptor signaling so as to alleviate effects of excess activation. We present an *in silico* reconstruction of the Toll-like receptor signaling network and convert it into a mathematical framework that is suitable for constraint-based modeling and analysis. This approach leads to the identification of potential candidates for drug-based mediation. In addition to identifying targets for drug mediation of the Toll-like receptor network, we also supply a network model that may be continually updated and maintained.

## Introduction

Toll-like receptors (TLRs) are a group of conserved pattern recognition receptors that activate the processes of innate and adaptive immunity [1]. Recent activity has focused on the characterization of the TLR network and its involvement in the apoptotic, inflammatory, and innate immune responses [1]–[3]. TLR signaling is a primary contributor to inflammatory responses and has been implicated in several diseases including cardiovascular disease [4],[5]. Indeed, even in cases of desired inflammatory response, excessive activation of signaling pathways can lead to septic shock and other serious conditions [6].

As such, there is much interest in the development of methods to attenuate or modulate TLR signaling in a targeted fashion. For example, one approach involves the inhibition of specific reactions or components within the TLR network that will dampen undesired signaling pathways while not adversely affecting other signaling components [7],[8]. These reactions or components should ideally be highly specific to the TLR network and also to one transcription target. Therefore, the available, comprehensive data sets of the TLR network need to be put into a more structured, systematic format that enables better understanding of the associated signaling cascades, pathways, and connections to other cellular networks. Such a systemic approach is necessary to achieve the ultimate goal of mediating the effects of Toll-like receptor signaling upon the inflammatory, immune, and apoptotic responses. This need is particularly important given the amount of experimental data about TLR signaling that is already too large to be analyzed by simply viewing the complex web of overlapping interactions. So far, relatively few attempts have been made to organize the plethora of experimental data into a single unified representation [9]. Hence, there is clearly a need to investigate the function and capabilities of this network using a computational model, particularly to yield further insights into the mechanistic action of the TLRs and their immunoadjuvant effects.

Constraint-based reconstruction and analysis (COBRA) methods represent a systems approach for computational modeling of biological networks [10]. Briefly, all known biochemical transformations for a particular system (e.g., metabolic network, signaling pathway) are collected from various data sources listing genomic, biochemical, and physiological data [11],[12]. The reconstruction is built on existing knowledge in bottom-up fashion and can be subsequently converted into a condition-specific model (see below) [10],[13] allowing the investigation of its functional properties [14],[15]. This conversion involves translating the reaction list into a so-called stoichiometric matrix by extracting the stoichiometric coefficients of substrates and products from each network reaction and placing lower and upper bounds (constraints) on the network reactions. These constraints can include mass-balancing, thermodynamic considerations (e.g., reaction directionality), and reaction rates (e.g., maximal possible known reaction rate) [14]. Additionally, environmental constraints can be applied to represent different availabilities of medium components (e.g., various carbon sources). Many computational analysis tools have been developed [14], including Flux balance analysis (FBA). FBA is a formalism in which a reconstructed network is framed as a linear programming optimization problem and a specific objective function (e.g., growth, by-product secretion) is maximized or minimized [14]. COBRA methods are well established for metabolic networks and both reconstruction and analysis tools are widely used [16]. Furthermore, these methods have been successfully applied to other important cellular functions such as transcription and translation [17], transcriptional regulation [18], and signaling, including JAK-STAT [19] and angiogenesis [20].

In this study, we present an extended and reformulated model for the TLR network, reconstructed based on the publicly available TLR map [9] and the COBRA approach [11],[12]. Signaling networks have been analyzed using extreme pathway (ExPa) analysis [19] and FBA [20]. However, since ExPa analysis becomes computationally challenging in large-scale, mass-balanced networks [21], we could not apply this method to the TLR network. In contrast, network modularization has been established as a method for reducing large-scale networks into more manageable units [22]–[24]. Another approach for reducing network complexity is to focus on input–output relationships [20],[25]. We used FBA to simplify the mesh of network reactions into ten functionally distinct input–output (DIOS) pathways, which show different patterns of signal activation control. Furthermore, we used this modular representation of the complex TLR signaling network to determine control points in the network, which are specific for a DIOS pathway. These control points allow for the modulation of TLR signaling in a targeted fashion, which will induce a change in undesired signaling while not having an adverse effect on other signaling components. Taken together, we show in this study how a signaling network reconstruction and FBA can be used to identify potential candidates for drug targeting.

## Results

### Reconstruction Approach

**Figure 1:**
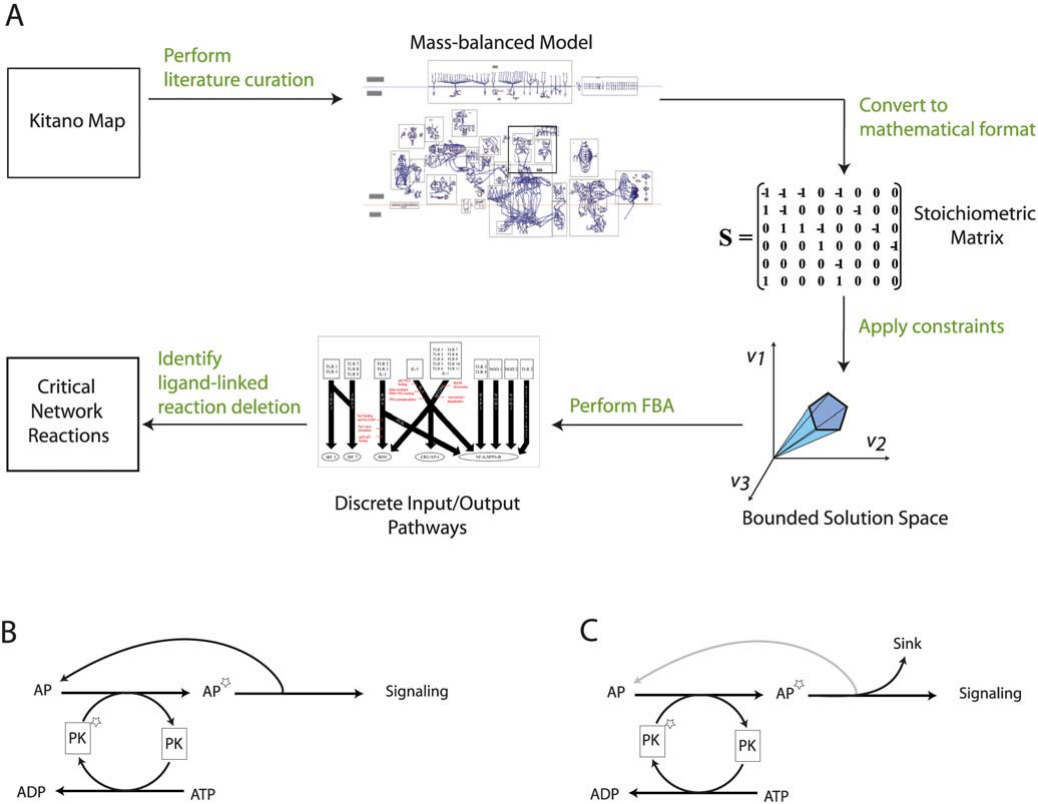
*ihs*TLR v1.0 reconstruction process. (A) Flowchart illustrating the necessary steps to convert the Kitano-TLR map [9] into a stoichiometric, mass-balanced model that can be functionally characterized using COBRA method and FBA. Using these computational tools, it was possible to determine a set of critical network reactions that are highly-specific candidates for TLR signaling mediation as changes in their activity attenuate the flux through their corresponding discrete input–output signaling (DIOS) pathways but have no adverse effect on the TLR network reactions. (B) The transfer of functional groups, such as phosphate groups, is very common in signaling pathways. We accounted for proteins explicitly in the corresponding network reaction. This created cycles that are artifacts of the modeling and decouple the phosphorylation/dephosphorylation reactions. Panel B illustrates such a case. The dephosphorylation of PK* to PK can run completely independently of the phosphorylation reaction of AP to AP* since AP is recovered in a subsequent step. The downstream signaling output is thus not dependent on the presence of PK*. (C) This panel illustrates how we circumvented this issue during the modeling by creating a sink reaction for AP* and thus interrupting the cycle formerly present. Since the modeling is only qualitative, the simulation result (e.g., signal yes/no) is not affected by this trick.

The basis for the network reconstruction was the recently published Kitano-TLR map, which visualizes the TLR network in great detail [9]. Since we intended to apply COBRA methods [14],[26], the Kitano-TLR map had to be converted into a self-consistent, mass- and charge balanced reaction network. Consequently, various modifications and extensions needed to be made in order to represent the TLR network comprehensively in the stoichiometric reaction format (Figure 1). These extensions were as follows:

- Kinase and phosphatase reactions were added to quantify the energy (ATP/GTP) consumption by the network reactions.
- The addition of ubiquitin ligase components and their substrate binding allowed for an explicit representation of the ubiquitination reactions. Additionally, internal transport reactions were added to enable the transport of network component between the cellular compartments.
- Binding proteins, which induce activation by conformational change, were added to accurately represent all requisites of a network reaction.
- The Kitano-TLR map represented some reactions in a manner unsuitable for COBRA modeling purposes by requiring a number of inputs jointly to activate a certain output whereas *in vivo* any single input can trigger the downstream output. The corresponding reactions were updated to allow the signal transfer from any ligand to the corresponding output.

These changes were necessary to create a more biologically relevant model and also to take into account the metabolic and transport requirements of signaling networks. The resulting model is able to make predictions in the context of environmental and energy constraints. Taken together, the conversion to the stoichiometric representation of the TLR network required intensive literature search to clarify the status and function of proteins in the Kitano-TLR map, thereby leading to a comprehensive curation process. The resulting network was deemed *ihs*TLR v1.0, where ‘*i*’ stands for *in silico*, ‘hs’ for homo sapiens and v1.0 is the version number of this *in silico* TLR network. The formalism underlying *ihs*TLR v1.0 is in analogy to that of metabolic networks and thus enables the usage of COBRA methods [14],[26].

The use of COBRA methods is heavily dependent on the configuration of network constraints that model biochemical properties of the network. For example, in metabolic networks, enzyme suppression can be modeled by constraining the appropriate reaction to carry zero net flux. However, in signaling networks, such as the TLR network, many reactions involve activation/inactivation of a signaling complex via phosphorylation. In these cases, the mechanism involves the transfer of a phosphate group from the kinase to the signaling complex followed by re-activation of the kinase by the appropriate ATP-driven reaction (Figure 1B and 1C). When coupled with dephosphorylation of the signaling complex after signaling, this mechanism introduces a number of loops into the network whereby an adequate supply of ATP would seemingly induce active signaling without actually requiring the presence of a ligand. Therefore, in order to perform constraint-based analyses on the TLR network, we manually added constraints on such loop reactions to require both ATP-fueled phosphorylation as an energy source and ligand-based signaling input to drive active signaling (see Materials and Methods).

### Reconstruction Content

**Figure 2:**
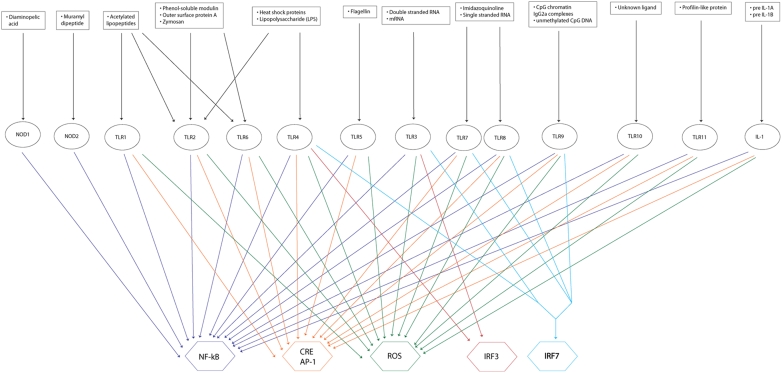
The input–output (I/O) relationships of the TLR network at the ligand-receptor-output level. There were a total of 49 ligands (see Table S3 for complete list), 14 receptors, and 6 outputs. Because the ligands are already well characterized with respect to their receptor specificity, it is unnecessary to carry out the input–output analysis at the level of the external ligand. Rather, the inputs can simply be considered to be signals from the receptors—this reduces the number of inputs from forty-nine to a mere fourteen. NF-kappa-B, CRE/AP-1, and ROS production were all highly redundant targets as almost all of the receptor inputs activated these outputs. IRF3 and IRF7 were much less redundant and were only activated in the case of a small subset of receptor inputs.

The *ihs*TLR v1.0 reconstruction comprised 909 reactions, which linked 752 distinct chemical species into a self-consistent network (Table 1). The reconstruction accounted for 14 Toll-like receptors, 49 ligands, and 6 outputs (see Figure 2 and Tables S1, S2, S3, S4). A confidence level was assigned to each network reaction on a scale from one to five, with one being a lack of conclusive literature evidence and a five being strong, conclusive literature evidence including review articles (see Materials and Methods). The average confidence level for the entire network was 3.21, with a total of 306 unique article citations (see Table S7). Chemical formulae and cellular localization information were also included. For instance, each species was assigned a chemical formula for the covalent modification groups (e.g., phosphate, ubiquitin), which accounted for the metabolic costs of signaling. A total of six compartments (extra-organism, cytosol, nucleus, lysosome, endoplasmic reticulum, and vesicle) were considered to accurately represent the intracellular trafficking. These additions allowed for a more biologically accurate representation of the TLR network and for finer control over the network fluxes through transport and metabolic reactions.

**Table 1:** Statistics of *ihs*TLR v1.0.

**Total number of network reactions**	**909**
Number of internal network reactions	641
Number of exchange reactions	268
Average confidence level	3.21
**Total number of network species**	**752**
Number of ligands	49
Number of receptors	14
Number of metabolites	23
Number of kinases	158
Number of phosphatases	16
Number of outputs	6
**Total number of discrete signaling pathways**	**10**

The addition of kinases, phosphatases, and binding proteins as well as the stoichiometric accounting of metabolites, greatly increased the number of reactions and species from the Kitano-TLR map [9]. This increase in complexity was necessary to enable the usage of COBRA methods.

### Network Map

To visualize the network content we created a map of the *ihs*TLR v1.0 reconstruction using SimPheny (Genomatica) software. All six compartments were represented in the map, with the appropriate localization for the reactions and components. Internal transport reactions allowed for the transfer of network components between the compartments and these reactions were explicitly positioned on the boundaries of the compartments within the map. In many cases, reactions that shared substrates or products were joined on the map to show the interconnections between the reactions of the TLR network. Overall, an organization of the map was chosen that enabled the visualization of the parallel structure of TLR signaling from the extra-cellular ligand to the transcription-level targets (signal output). The complete network map can be found in Figure S1.

### Network Connectivity

**Figure 3:**
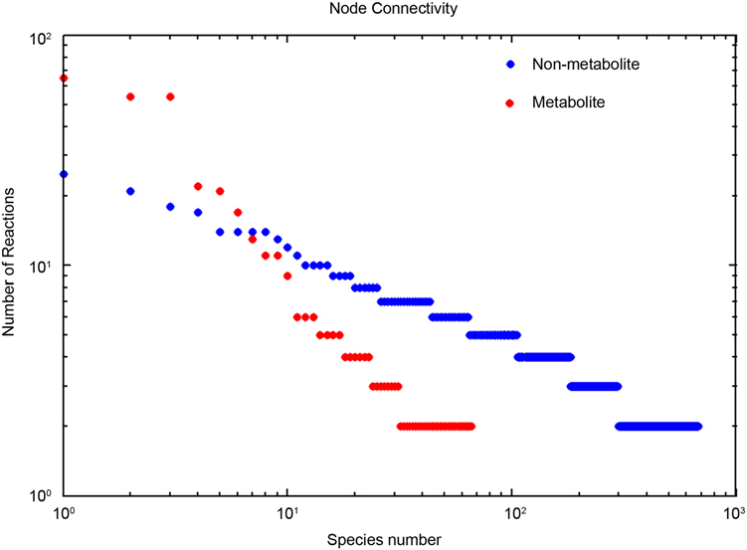
Node connectivity in *ihs*TLR v1.0. The rank-ordered results were separated for metabolic and non-metabolic species. The non-metabolic species include: ligands, receptors, signaling proteins, outputs (see also Table 1). The three most highly connected species were ATP, ADP, and H+, which participated in 57, 57, and 68 reactions, respectively. In contrast, no non-metabolic species participated in more than 24 reactions. The node connectivity distribution of metabolic and non-metabolic species followed a power law distribution. The fact that the higher connectivities were associated with metabolites illustrates the importance of mass- and charge- balanced network reconstructions for biological accuracy.

The topological properties of the *ihs*TLR v1.0 network were assessed by determining its node connectivity distribution. The node distribution defines the degree to which a particular network component is connected to the entire network, and can be easily computed. The three most highly connected species were ATP, ADP, and H+, which participated in 57, 57, and 68 reactions, respectively (Figure 3). Furthermore, inhibitor of kappa light polypeptide gene enhancer in B cells kinase (IKK), a non-metabolic component, was found to participate in 24 reactions, implying its central role in TLR signaling. Other highly connected non-metabolic species were a phosphorylated version of IKK and the MyD88 dimer, which is known to be a key TLR adaptor protein. Additionally, although both metabolic and non-metabolic species followed the general power law distribution (Figure 3), it is notable that most of the more highly connected (participating in more than 24 reactions) species were metabolites, highlighting the importance of mass- and charge- balancing of a signaling network to accurately represent its biological properties.

The normalized connectivity centralization is a commonly used index of the node connectivity distribution and measures the extent to which certain nodes are more central than others independent of actual network size [27],[28]. The centralization is measured from 0 to 1 with a higher value corresponding to the presence of more highly connected nodes. For the TLR network, we calculated a centralization index of 0.08, which indicated a lower level of centralization. For comparison, the centralization indices of the core metabolic networks of *S. pneumoniae* and *P. furiosus* have been calculated to be 0.24 and 0.10, respectively [28]. This observation suggests that the network contains fewer nodes critical for the network functionality and that deletion of these nodes may disrupt the entire network functionality.

### Input–Output Relationships

Input–output (I/O) relationships define the set of possible outputs from a defined set of input cues regardless of the internal paths connecting the inputs and outputs. As the characterization of external ligands with their respective TLRs is well established [29]–[31], the I/O relationships were considered rather on the level of a receptor input to a transcription level output. The I/O relationships were calculated using FBA.

The results of the I/O relationship analysis identified NF-kappa-B to be the most commonly activated output, as it was induced by all signaling inputs except for TLR3 (Figure 2). Indeed, seven of the ten functionally distinct DIOS pathways (discussed below) resulted in NF-kappa-B activation. The other outputs had varying degrees of expression, with IRF7 being a single output that required multiple inputs for activation. Consequently, these *in silico* results suggest a relative prevalence of the network to promote NF-kappa-B activation caused by pathway redundancy.

Furthermore, some ligands can bind to multiple receptors, which can lead to the activation of an overlapping set of outputs (Figure 2). For instance, lipopolysaccharide (LPS) binds to TLR2 and TLR4; however, TLR2 activates NF-kappa-B, AP-1, CRE, and reactive oxygen species (ROS) production, while TLR4 activates the same outputs except for the ROS production, which is replaced by the IRF3 activation. This redundancy from the overlapping I/O relationship confers robustness to the network, since LPS could activate an output, e.g., NF-kappa-B, despite inhibition of a receptor. An example of this robustness might be the activation of the NF-kappa-B output in the presence of both the LPS ligand and a decoy soluble TLR2 receptor. In contrast to the observed overlapping activation of some outputs, IRF7 was found to be the only output requiring multiple receptor-ligand binding for activation (TLR3/4 and TLR7/8/9) (Figure 2). IRF7 has been shown to play a role in the transcriptional activation of interferon beta chain genes. The reason for the multiple ligand input is the functional overlap of two activation pathways: complex formation of MyD88 and IRF7 followed by TRAF6-dependent phosphorylation of IRF7 and dissociation of the ubiquitinated TRAF6-MyD88 complex [32]–[35]. This transactivation induction mechanism suggests a high level of control for this output.

### Distinct I/O Signaling Pathways—DIOS Pathways

**Figure 4:**
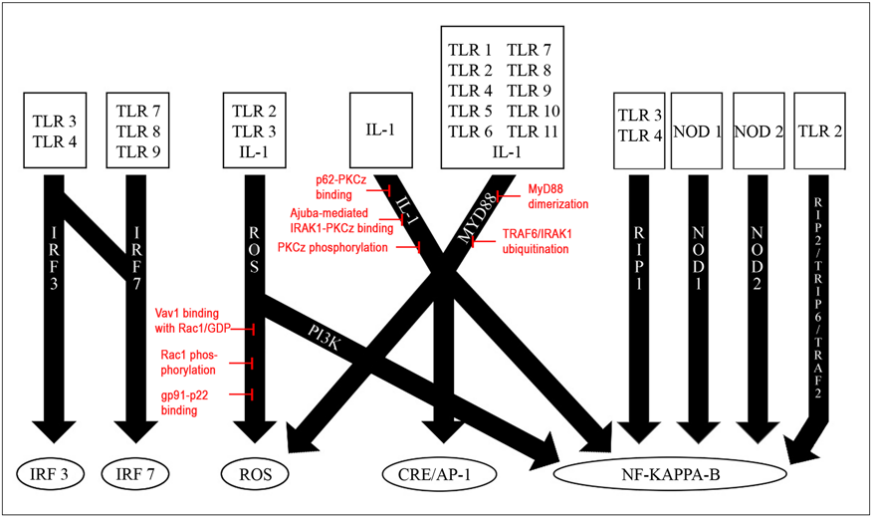
An overview of the discrete signaling (DIOS) pathways defined in the TLR network. There were a total of ten pathways that signaled from input receptor signals to output transcription-level objectives. These ten pathways shared fourteen receptor signals and five output objectives. The most redundant objective was NF-kappa-B activation, which was the target for a majority of the pathways. Indeed, four of the pathways—RIP1, NOD1, NOD2, and RIP2/TRIP6/TRAF2—signaled only to NF-kappa-B. However, also note that IL-1 and a large subset of the TLRs signaled to multiple objectives through a variety of pathways such as PI3K, IL-1, and MyD88. Overall, this receptor-pathway-output format allowed for a better understanding of the TLR network and its input–output relationships, and also for the calculation of essential reactions as candidates for signaling mediation. Red: A summary of the eight critical network reactions identified through our analysis (see text). These control points were located within the ROS production, IL-1, and MyD88 pathways. Although some essential network reactions were identified for the other discrete signaling pathways, they were unsuitable for selective inhibition due either to their role in other signaling processes or their lack of specificity to a particular pathway.

We wished to identify candidates for mediation in the TLR signaling network. To qualify as competent drug targets these candidates were required to attenuate the TLR signaling in a targeted fashion, i.e., by inducing changes in the target signaling pathway while not having an adverse effect on other signaling components in the TLR network. Subsequently, our calculated I/O relationships could be used to determine such mediation candidates as they represented the structure of the complex TLR signaling network. To further modularize and simplify the network, we applied FBA to identify sets of signaling reactions associated with a given input. In a further step, we grouped these sets of signaling reactions based on their intermediate products. By doing those, we obtained 10 functionally distinct groups of signaling reactions, the so-called *distinct I/O signaling pathways*, or DIOS pathways. The signaling pathways summarized within one DIOS pathway thus share the same input, same output, and some, but not necessarily all, intermediate reactions. In contrast, two DIOS pathways differ by an input, an output, or an intermediate reaction (determined by function and experimental evidence). The 10 DIOS pathways triggered signaling from 14 receptors (inputs) to 6 outputs within the TLR network (Figure 4). Whereas some signal outputs could be activated by numerous overlapping DIOS pathways (e.g., NF-kappa-B), other signal outputs (e.g., IRF7) required multiple receptor-ligand binding events along one single DIOS pathway for signal mediation. This functional grouping of network reactions led to a dramatic reduction in complexity by introducing the DIOS pathways as functional modules of the TLR signaling network.

### Ligand-Linked Reaction Deletion

As defined above, potential signaling mediation targets should be unique to a DIOS pathway and alteration of the flux through such targets should affect the performance of the entire DIOS pathway. Note that such flux alteration will not affect the remaining TLR signaling network. To identify such control points, we again employed FBA by determining essential network reactions along the DIOS pathways. This approach enabled us to focus on one activation pathway by disregarding alternate signaling routes that may be activated by a ligand. Therefore, by limiting the scope of analysis to individual pathways, the redundancy inherent in the TLR network was bypassed and control points in the pathway could be readily identified. These essential network reactions, or control points, are suitable candidates for TLR signaling mediation. A further subset of the essential network reactions was determined by requiring the reactions to have the properties of (i) being specific to a DIOS pathway, (ii) not affecting the flux through other DIOS pathways, and (iii) being capable to completely control the flux through a particular DIOS pathway. This refined subset comprised the so-called critical network reactions of the TLR network, and represents the best candidates for TLR signaling mediation.

**Figure 5:**
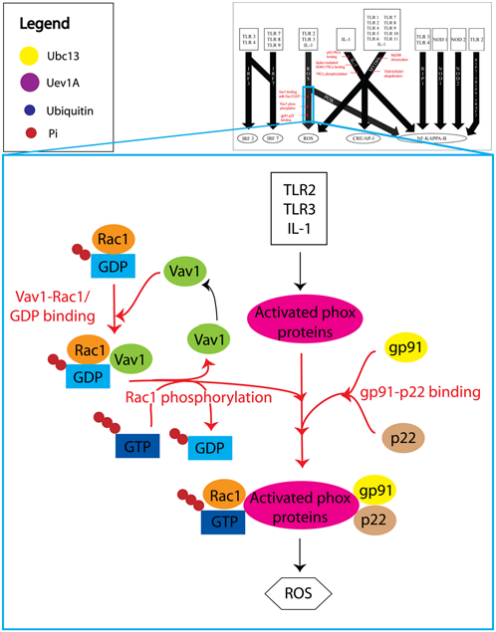
A simplified illustration of the reactive oxygen species (ROS) production DIOS pathway. The three critical network reactions are highlighted in red. Although there were over forty reactions in the ROS production pathway, most were associated with the TLR-induced activation of various phox proteins by the protein kinases PDK1 and PKCz. However, because PDK1 and PKCz work in parallel, none of these reactions could control the flux through the entire pathway, and therefore were not critical network reactions. On the other hand, the three critical network reactions *Vav1-Rac1/GDP binding*, *Rac1 phosphorylation*, and *gp91-p22 binding*, produced the two other components that comprised the final phox protein complex, and were therefore critical to the overall output ROS production. Note also that these critical network reactions were localized to the ROS production pathway and did not interfere with other cellular processes. Thus, they represent ideal targets for mediation of TLR-induced ROS production.

**Figure 6:**
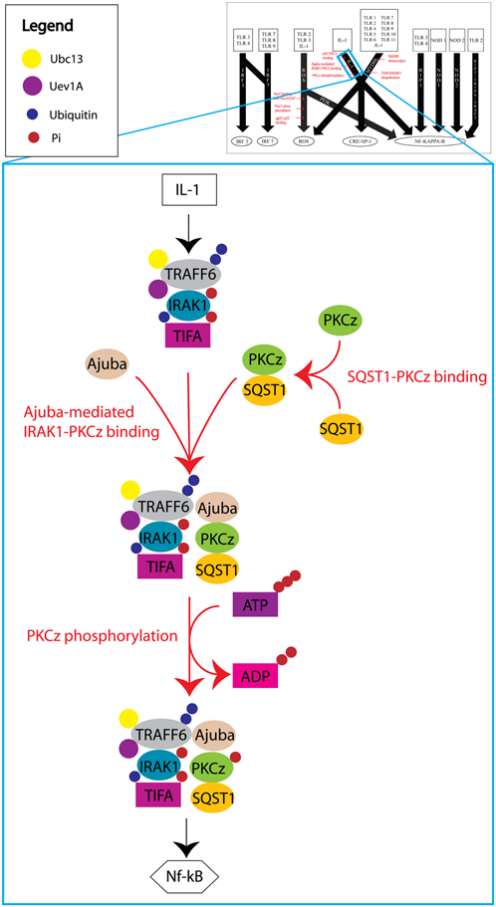
A simplified illustration of the IL-1 DIOS pathway. There were three critical network reactions that controlled the IL-1 induced activation of NF-kappa-B. Uninhibited IL-1 signaling induced formation of a TRAF6/Ajuba/PKCz/SQST1 complex followed by autophosphorylation at the Thr-560 residue of PKCz. This activated complex then signaled downstream to NF-kappa-B via IKK phosphorylation. The three critical network reactions inhibited IL-1 induced NF-kappa-B activation by preventing the formation and subsequent autophosphorylation of the TRAF6/Ajuba/PKCz/SQST1 complex. Unlike inhibitors such as IL-1R2 and soluble IL-1R, which mediate IL-1 signaling by preventing the activation of the IL-1 receptors, the three critical network reactions worked by disrupting other components of the IL-1 DIOS pathway and did not affect the activation of IL-1 receptors.

**Figure 7:**
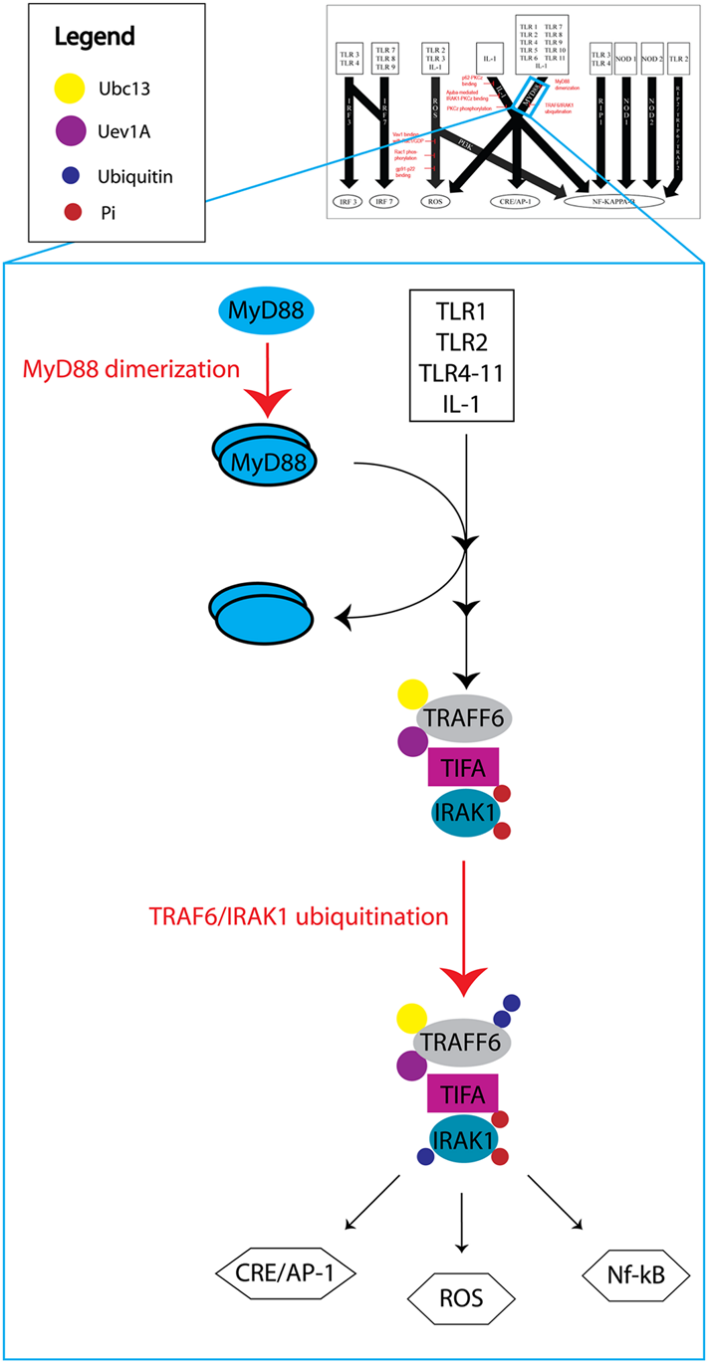
A simplified depiction of the MyD88 DIOS pathway. The two critical network reactions *MyD88 dimerization* and *TRAF6/IRAK1 ubiquitination* are highlighted in red. Formation of the MyD88 homodimer favors recruitment of IRAK1 into a complex with TRAF6 [65]. The MyD88 dimer then dissociated from this complex to be either degraded or reused. The second critical network reaction, *TRAF6/IRAK1 ubiquitination*, occurred via the ubiquitin-conjugating enzymes Ubc13 and Uev1A, and was necessary for activation of NF-kappa-B and AP-1 through canonical IKK phosphorylation. Either of the two critical network reactions could completely abrogate the flux through the MyD88 pathway even though the TIR- or TIRAP-dependent TLR signaling was almost always active.

A total of 41 essential network reactions were found along the 10 DIOS pathways (see Table S6). After applying our specificity requirements, a subset of eight critical network reactions was identified to be present in the *ihs*TLR network along three DIOS pathways (Figures 5, 6, and 7). These critical network reactions as well as their known potential as candidates for TLR signaling mediation are discussed in the following.

#### ROS production

Reactive oxygen species (ROS) have been implicated in a variety of cellular processes including proliferation, differentiation, and apoptosis [36]. In our analysis of the *ihs*TLR network, we identified three critical network reactions in the ROS production DIOS pathway (Figure 5). Two of these reactions, *Vav1-Rac1/GDP binding* and *Rac1 phosphorylation*, were associated with the activation of the Rho family GTPase Rac1, which has been shown to be involved in the production of ROS [37]. The third critical network reaction, *gp91-p22 binding*, has been shown to be necessary for the assembly of NADPH oxidase, which in turn produces ROS [38]. Deletion of any of these three critical network reactions reduced the ROS production output through this DIOS pathway to zero. Additionally, none of the three reactions was found to have an effect on other DIOS pathways. As such, these three reactions are strong candidates for mediation of TLR-induced ROS production.

#### IL-1

The various members of the interleukin-1 (IL-1) family have been implicated in many processes including inflammation, hematopoiesis, and apoptosis [39]. Various inhibitory factors such as IL-1R2, soluble IL-1R, and IL-1R antagonist have been identified and shown to mediate the effects of IL-1 signaling [40],[41]. However, none of these factors can mediate specific IL-1 signaling targets. To this end, we identified three critical network reactions local to the IL-1 DIOS pathway that could specifically mediate the IL-1 induced activation of NF-kappa-B (Figure 6). The three critical network reactions were *Ajuba-mediated IRAK1-PKCz binding*, *SQST1-PKCz binding*, and *PKCz phosphorylation* (see Figure 7). Ajuba and SQST1 have been previously shown to influence IL-1 induced activation of NF-kappa-B [42],[43]. Autophosphorylation of the Thr-560 residue on PKCz has also been independently shown as a prerequisite for enzymatic activity [44]. Importantly, deletion of any of the three critical network reactions completely inhibited NF-kappa-B activation via IL-1 signaling without disruption of the IL-1 receptor. Therefore, these three reactions are suitable candidates for the mediation of IL-1 induced NF-kappa-B activation.

#### MyD88

MyD88 has been well characterized as an essential adaptor protein for TLR signaling, and has been linked with both NF-kappa-B and AP-1 activation [1],[32]. Indeed, MyD88 has been shown to associate with all known TLRs except for TLR3, and thus the MyD88 DIOS pathway is of vital importance to the overall TLR signaling. Our analysis identified two critical network reactions in the MyD88 pathway (Figure 7). These two reactions, *MyD88 dimerization* and *TRAF6/IRAK1 ubiquitination*, were both local to the MyD88 DIOS pathway and did not affect the flux through other pathways. Moreover, deletion of either of the two reactions resulted in a complete abrogation of the flux through the MyD88 pathway, which in turn disrupted the NF-kappa-B and AP-1 outputs.

Taken together, the identification of critical network reactions in *ihs*TLR compiled a list of strong candidates for TLR signaling mediation. Moreover, most of these candidates were non-obvious targets for signaling mediation as they were not distinguishable simply by their node connectivities.

## Discussion

In this study, we presented the first large-scale, stoichiometric reconstruction of the human TLR signaling network, *ihs*TLR. The initial reconstruction was based on the Kitano-TLR map [9] and manually converted into a format suitable for steady-state constraint-based modeling by (i) mass- and charge balancing network reactions, and (ii) adding proteins and energy currency to the reactions, where appropriate, using TLR-specific literature (Figure 1 and Table 1). *ihs*TLR was subsequently converted into a mathematical model and analyzed with respect to network connectivity, input–output relationships, and discrete input–output signaling (DIOS) pathways. A total of 10 DIOS pathways were identified and 8 critical network reactions were found along these pathways representing candidates for TLR signaling mediation. We showed that the combination of signaling network reconstruction with constraint-based modeling techniques can lead to highly relevant functional and topological insight into the network and identification of potential high-specificity drug targets.

The presented network *ihs*TLR v1.0 is a comprehensive reconstruction of the TLR signaling pathways and adjacent signaling pathways. In addition, metabolic cost associated with signaling was accounted for by including metabolites, such as ATP, in the network reactions and by creating transport and exchange reactions for the metabolites. This will enable future integration of the TLR signaling network with the existing human metabolic network [23]. Integrated models of metabolism and signaling have been recently published for small scale networks [45],[46]. Furthermore, the network was analyzed in terms of pathway activation or inactivation (i.e., ‘on’ versus ‘off’); hence, the magnitudes of the fluxes through the reactions were not a focus of the analysis nor were they necessary to determine I/O relationships. In the future, if data for signaling fluxes through different pathways become available, they can be directly applied to the network for more nuanced analysis of pathway activation/inaction.

To date few signaling networks have been reconstructed and modeled using COBRA approaches. Dasika et al. [20] presented recently how FBA can be successfully applied to study signaling networks. Here, we presented a related method to structure the complex network content and obtain insight into the functional network topology. Extreme pathway analysis (ExPa), which was useful for the topological characterization of the JAK-STAT network [19], could not be applied to *ihs*TLR, as the size of the network and the connectivity of the species made it infeasible. The number of ExPas correlates to the size and complexity of the network [21], rendering the enumeration of the ExPas computationally challenging in large networks. The determination of input–output (I/O) relationships is a great simplification of complex signaling networks as it treats the network as a “black box” and asks the simple question of which input instances activate which output targets [25],[47]. Overlapping I/O relationships illustrate a network's redundancy and robustness within this black box [46]. In the reconstructed TLR network, we considered 14 distinct input receptors and 6 distinct signaling outputs (Figure 2). The transcription factor NF-kappa-B was activated by all but one Toll-like receptor upon ligand binding. This functional redundancy illustrates the importance of this signaling output for the entire network, and greatly reflects the involvement of the TLR signaling network in the inflammatory response, as NF-kappa-B plays a key role in immune response regulation. In contrast, the interferon regulatory factor 7 (IRF7) needed the ligand binding of two independent TLRs for activation, which implies stringent regulation of this factor's activity. This transcription factor IRF7 has been found to be directly involved in immune response to viral infections by activating IFN-α/β genes [48].

These I/O relationships, in conjunction with FBA, were used to identify the specific reactions involved in triggering the signal between an input and an output. Hereby, input-specific reaction subsets were identified and grouped based on common intermediate products identified through previous experimental validation. We deemed these reaction sets *discrete I/O signaling (DIOS) pathways*, as they spanned the entire signaling network and modularized the reaction mesh to clearly defined subsets that could be studied independently (Figure 4). All network reactions were grouped into 10 DIOS pathways, illustrating again the redundant, overlapping structure of the TLR network. These DIOS pathways were then analyzed for essential reactions to identify control points, or critical network reactions, that allowed for attenuation of the overall flux through the DIOS pathways. These critical network reactions controlled the flux through a DIOS pathway but did not affect the flux through other pathways, and are therefore ideally suited to the method of selective inhibition. Selective inhibition of TLR-specific signals involves mediation of output fluxes without the disruption of components that are known to play a role in other cellular processes. For example, the IL-1 inhibitors IL-1R2, soluble IL-1R1, and IL-1R1 antagonist are not suitable for selective inhibition because they disrupt IL-1 signaling on the whole instead of targeting specific IL-1 targets. The critical network reactions identified in the IL-1 DIOS pathway can be used for selective inhibition because they do not disrupt IL-1 signaling, but rather prevent the products of IL-1 signaling from reaching their output objective (in this case, NF-kappa-B) (Figure 6). Selective inhibition of TLR signaling is especially important because it is essential for maintaining the innate immune response and also for enhancing the adaptive immune response; over-inhibition could lead to a reduction in the body's defenses against pathogens, whereas dysfunctional inhibition can lead to various autoimmune disorders. Therefore, it is crucial that mediation targets be highly specific.

Our analysis yielded a total of eight critical network reactions along three of the DIOS pathways (ROS production, IL-1, and MyD88). A summary of these critical network reactions can be seen in Figure 4. Although these critical network reactions can completely mediate the flux through a specific DIOS pathway, they do not always completely zero out an output. For example, because NF-kappa-B is a highly activated target, disruption of the MyD88 pathway does not completely stop NF-kappa-B activation, as it still occurs via the IL-1 and other pathways (Figure 4). Only in the absence of other DIOS pathways, the disruption of the MyD88 pathway completely abrogates NF-kappa-B activation. Additionally, the ROS production target is also incompletely inhibited by disruption of a single DIOS pathway. This robustness appears to come at a price of specificity as ROS production will be enabled in multiple environments. However, the two DIOS pathways that lead to ROS production have different ligand effectors, and are therefore specific to certain TLRs. The ROS production pathway has TLR3 as an input, whereas the MyD88 DIOS pathway does not. Recent studies have shown that TLR3 is vital in host defense against a variety of infections including West Nile virus [49], and this DIOS pathway specificity may hold clues as to why TLR3 plays such an important role. We believe that the inherent redundancy of the TLR network leads to such crosstalk between pathways and therefore makes necessary the development of inhibition combinations that can effectively mediate multiple DIOS pathways.

One such case may be the atypical protein kinase PKCz, which is found in two of the reactions (in the ROS production and IL-1 pathways). Removal of the PKCz component from the TLR network resulted in complete abrogation of both ROS production and IL-1 induced NF-kappa-B activation, showing that PKCz has the ability to control multiple objective outputs. In both pathways, PKCz enzymatic activity is activated by phosphorylation at critical residues [44]. Disruption of this process would be the physical equivalent of removing the PKCz component from the TLR network and could have a powerful inhibitory effect on both ROS production and IL-1 induced NF-kappa-B activation.

Our constraint-based analysis allowed us to characterize the aforementioned eight critical network reactions as targets for selective mediation. Next, we looked to validate these predictions by searching for experimental evidence of inhibitory roles for species involved in these critical network reactions. For example, Vav1, which plays a critical role in the ROS production pathway in our model, was recently shown to be required as an upstream signaling protein for NADPH oxidase activity [50]. The role of the PKCz isozyme in the NF-kappa-B pathway and downstream cellular functions such as apoptosis has also been heavily studied [51],[52]. Overall, our model is strongly consistent with published evidence regarding inhibition targets within the TLR signaling network, but also predicts several novel targets that have not been well studied. Experimental investigation of these critical network reactions may yield important methods for mediating TLR signaling and its inflammatory and immune responses. Some examples of known intermediates involved in diseases are listed in Table 2. These examples highlight the relevance and importance of computational characterization of the complex TLR signaling network to promote further understanding of its role in common diseases. Additionally, continued curation of the *ihs*TLR model as the TLR network is elucidated will allow for further functional insights into the TLR signaling process. Future additions to this model may include quantitative fluxes that would allow for the characterization of relative attenuation quantities and signaling thresholds. Another avenue of interest would be to study the dynamics of the TLR network in order to better understand the temporal nature of the signaling cascade.

**Table 2:** Clinical correlates of DIOS pathways.

DIOS Pathway	Intermediate Species	Related Diseases	References
ROS production	Rho family GTPases	Pro-cancer/neoplastic processes, vascular disease	[57],[58]
IL-1	IL-1	Rheumatoid arthritis, ankylosing spondylitis, Alzheimer's disease	[59]–[61]
MyD88	MyD88	Malaria, pneumococcal infections	[62]–[64]

Disruption of the TLR pathways can result in a wide range of pathophysiological conditions. This table summarizes some of the conditions and diseases in which particular intermediate species are involved, supported by *in vivo* human and animal studies. The corresponding DIOS pathways are listed in the far left column.

## Materials and Methods

### Network Reconstruction

The TLR network was reconstructed using SimPheny, version 1.12.0.0 (Genomatica), based on a previously described reconstruction approach [11],[12]. An initial framework of reactions and species was retrieved from a previously published TLR map [9]. Additional reactions and species were manually added to this framework with the goal of achieving greater biological relevance and accuracy. Most of the additions made were taken directly from literature sources. Some sink and source reactions were added to eliminate gap conditions and provide for system boundaries. Chemical formulae were assigned where appropriate: the generic R group was used for any network compound that was involved in a mass transfer equation, and all modifications (i.e., phosphorylation, ubiquitination, and dimerization) were also included in the formulae. Additionally, because all of the network compounds were cellular species, the R group could also be interpreted as a general fatty acyl chain. Six different compartments were associated with all of the network components and necessitated the addition of internal transport reactions. These six compartments were extra-organism, cytosol, nucleus, lysosome, endoplasmic reticulum, and vesicle. Components that participated in reactions in multiple compartments were represented by separate species (e.g., ATP[c], ATP[n], etc.).

Confidence scores were assigned on a scale from zero to five to every reaction to represent the reliability of the literature sources. The scale is shown in Table 3.

**Table 3:** Confidence scores for network reactions.

Confidence Score	Interpretation
0	No literature support. Reaction is added for gap closure.
1	Conflicting/unsubstantiated literature evidence.
2	Some literature support on an assay level—no mechanistic characterization.
3	Some literature support including mechanistic characterization.
4	Strong literature support with repeated results.
5	Conclusive literature support.

These scores represent the reliability of the experimental evidence for a given reaction in the model.

All of the network reactions were mass- and charge-balanced and were labeled as either reversible or irreversible. Most of the transport and all of the exchange reactions were irreversible, and all of the internal reactions were irreversible on the basis of corresponding thermodynamic considerations. A list of the network content can be found in Table S1, S2, S3, S4.

### Constraint-Based Modeling

The reconstructed network was represented by a stoichiometric matrix, **S** (m×n), where m was the number of network components (metabolites, proteins, and complexes) and n was the number of network reactions. Reactions within the network were mass-balanced such that **S**·**v** = 0, where **v** was a steady-state flux vector [53],[54]. Additional constraints on each reaction had the form α_i_≤**v**_i_≤β_i_, where α_i_ and β_i_ represented the lower and upper limits of the corresponding reaction flux. These additional constraints were added to reactions that allowed for loops in the model due either to the recycling of various kinases and phosphatases or to internal feedback cycles such as those present in the MAPK pathway (Figure 1B and 1C). These loops occurred when the activation of a signaling complex was linked with kinase-driven phosphorylation. Because kinases are recycled for re-use inside the cell, each kinase-driven phosphorylation reaction was linked to another reaction that involved the re-activation of the kinase by either autophosphorylation or some other mechanism as given in literature (Figure 1B and 1C). When these two reactions were added to the network, a loop resulted and therefore required the additional of these manual constraints to prevent false negative signaling. In non-loop cases, the lower limits α_i_ were set to zero for irreversible reactions; whereas β_i_ were used to vary the constraints on internal network reactions, and to limit the amount of metabolite available through exchange reactions. For reversible reactions, α_i_ was set to -β_i_. The unit for each reaction flux was defined to be µmol/g_protein_/min. The TLR network model, including some simulation constraints, can be found in Dataset S1.

### Network Connectivity

The network connectivity was calculated by converting the stoichiometric matrix **S** into a binary matrix **Ŝ** such that: **Ŝ_ij_** = 0, if **S_ij_** = 0 and **Ŝ_ij_** = 1, if **S_ij_**≠0. From here, the network connectivity for each network component x_i_ was calculated simply by summing over all j for the row **Ŝ_ij_**.

The distribution of the network connectivity can be represented by the normalized connectivity centralization, given by
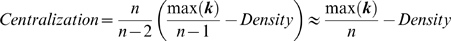
where n is the number of network components and **k** is a vector of the node connectivity values. The density is a measure of the mean off-diagonal adjacency and is given by
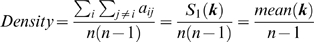
These network properties are well established and have been discussed recently [27]. The normalized connectivity centralization ranges from zero to one, with a higher value indicative of the presence of nodes that are far more central than other nodes.

### Input–Output Analysis

The I/O relationships were calculated using the flux balance analysis (FBA) encoded in the COBRA toolbox [26]. This analysis takes as input a single objective reaction and then attempts to optimize for this objective while maintaining a set of manually determined constraints. We performed this analysis independently for six objective reactions—*NF-kappa-B phosphorylation*, *IRF3/ISRE binding*, *IRF7/ISRE binding*, *PKC-induced Phox complex formation*, *Fos-Jun-AP1 binding*, and *CREB-CRE site binding*. These six objective reactions were selected on the basis of their importance as products of TLR signaling and their role in physiological symptoms. Baseline flux values were first obtained for each objective reaction by performing FBA with zero ligand input. Then, for each objective reaction, we iterated over the set of single receptor inputs and recorded the objective flux values for each receptor input with the receptor input flux constrained to **v**_i_ = 1.0 µmol/g_protein_/min. These values were then compared with the baseline flux through the objective reaction that existed even without ligand input. Any net positive gain in the flux through the objective reaction was interpreted to be active signaling. The input–output relationships of the network were represented in a matrix format, with each column corresponding to an objective reaction and each column corresponding to a single receptor input.

### Discrete Input–Output Signaling (DIOS) Pathways

The DIOS pathways were calculated using the same six objection reactions as in the I/O analysis (see above), and with the 14 input receptors as defined in Table S3. For each receptor-output pair, we used FBA to optimize for the output flux. Each **v**_i_ for the receptor input was set to be 1.0 µmol/g_protein_/min. The optimized network fluxes were then filtered by removal of loop reactions (reactions that carried flux even without ligand input). These loop reactions exist because some enzymes and binding proteins are recycled after a reaction and therefore are necessary to accurately represent the TLR network. These loop reactions are also thermodynamically infeasible without some external balance, and therefore warrant the application of manual constraints [55],[56]. The effect of these removals is negated by the addition of sources for any component that may be affected. Thus, control of the components that participate in loop reactions in essentially shifted from the loop to a source reaction in our model. For each loop, we constrained the corresponding enzyme/binding protein deactivation reaction to **v**_i_ = 0 µmol/g_protein_/min. This deleted the feedback mechanism that would trigger false signaling. A sink reaction **s**_i_ was added to the model using SimPheny for the deactivated protein to construct a complete network. From this modified model, a baseline set of network fluxes was then obtained by FBA. We then iterated FBA over the set of input–output pairs without these constraints and subtracted the baseline set to obtain a reduced set of network fluxes. The reduced set of network fluxes was then the set of network reactions differentially activated by the presence of a receptor input. This set was then broken down into DIOS pathways according to the experimentally verified intermediate components found in a pathway. For example, the MyD88 DIOS pathway utilizes the MyD88 adaptor protein to signal downstream. A complete list of these intermediate components is given in Table S5. Visual inspection of this differentially activated set was sometimes necessary to distinguish parallel pathways in which one receptor signaled to the same output through different DIOS pathways. The process of identifying DIOS pathways is summarized in the following pseudo code:

*for each receptor input*

* for each output objective*

*  optimize for maximum objective flux using FBA*

*  remove loop reactions by subtracting baseline reaction set*

*  visual inspection (if necessary) to identify parallel pathways*

*  reduced set of network reactions is a DIOS pathway*

* end*

*end*

This process was carried out using the SimPheny Simulation module. All transport and metabolite exchange fluxes were constrained to the arbitrary values of v_min_ = −500 µmol/g_protein_/min and v_max_ = 500 µmol/g_protein_/min. All internal reaction fluxes were constrained to v_min_ = 0 µmol/g_protein_/min and v_max_ = 10 µmol/g_protein_/min. The objective function was defined to maximize for the output flux.

### Critical Network Reactions

Intermediate reactions were selected to represent the flux through each DIOS pathway. The intermediate reactions selected were unique to a single DIOS pathway, and accurately represented signaling through the pathway (see Table S5 for list of intermediate reactions). Ligand-linked reaction deletion was then used to analyze each DIOS pathway. Ligand-linked reaction deletion differed from the typical reaction deletion study in that only a single DIOS pathway was considered per study, and all other reactions were constrained to v_min_ = 0 µmol/g_protein_/min and v_max_ = 0 µmol/g_protein_/min. All reactions included in the DIOS pathway were constrained to v_min_ = 0 µmol/g_protein_/min and v_max_ = 10 µmol/g_protein_/min, and all transport and metabolite exchange reactions were constrained to v_min_ = −500 µmol/g_protein_/min and v_max_ = 500 µmol/g_protein_/min. The objective function was defined to be the intermediate reaction. This new approach allowed us to bypass the redundancy of the TLR network and focus on identification of critical network reactions for each DIOS pathway. For each DIOS pathway, reaction deletion was performed for each reaction and the flux values of the intermediate reaction were recorded. These flux values were then compared with the baseline flux value obtained under normal conditions. Any reaction that resulted in a complete impairment of the objective flux value was label to be a critical network reaction.

All calculations for this study were done using Matlab (Mathworks, Natick, MA) with Tomlab (Tomlab Optimization, Inc, Pullman, WA) as the linear programming solver.

## Supporting Information

Dataset S1 TLR_model in Matlab format (zip file)
(0.05 MB ZIP)

Figure S1 Map of the reconstructed TLR signaling network
(3.29 MB PDF)

Table S1 TLR network species
(0.39 MB PDF)

Table S2 TLR network reactions
(0.30 MB PDF)

Table S3 TLR network inputs
(0.02 MB PDF)

Table S4 TLR network outputs
(0.01 MB PDF)

Table S5 Intermediate reactions
(0.01 MB PDF)

Table S6 Critical network reactions
(0.01 MB PDF)

Table S7 Reaction references
(0.36 MB PDF)
