# Correction: Identification of Potential Pathway Mediation Targets in Toll-like Receptor Signaling

**DOI:** 10.1371/annotation/5cc0d918-83b8-44e4-9778-b96a249d4099

**Published:** 2009-11-13

**Authors:** Fan Li, Ines Thiele, Neema Jamshidi, Bernhard Ø. Palsson

Dataset S1 contains some errors. The correct dataset is available here:
