# Supplementary figures and images for: Identification of Potential Pathway Mediation Targets in Toll-like Receptor Signaling
Source: PLoS Comput Biol. 2009 Feb 20;5(2):e1000292. doi: 10.1371/journal.pcbi.1000292 (PMC2634968; doi:10.1371/journal.pcbi.1000292)

Figure S1: Map of the reconstructed TLR signaling network

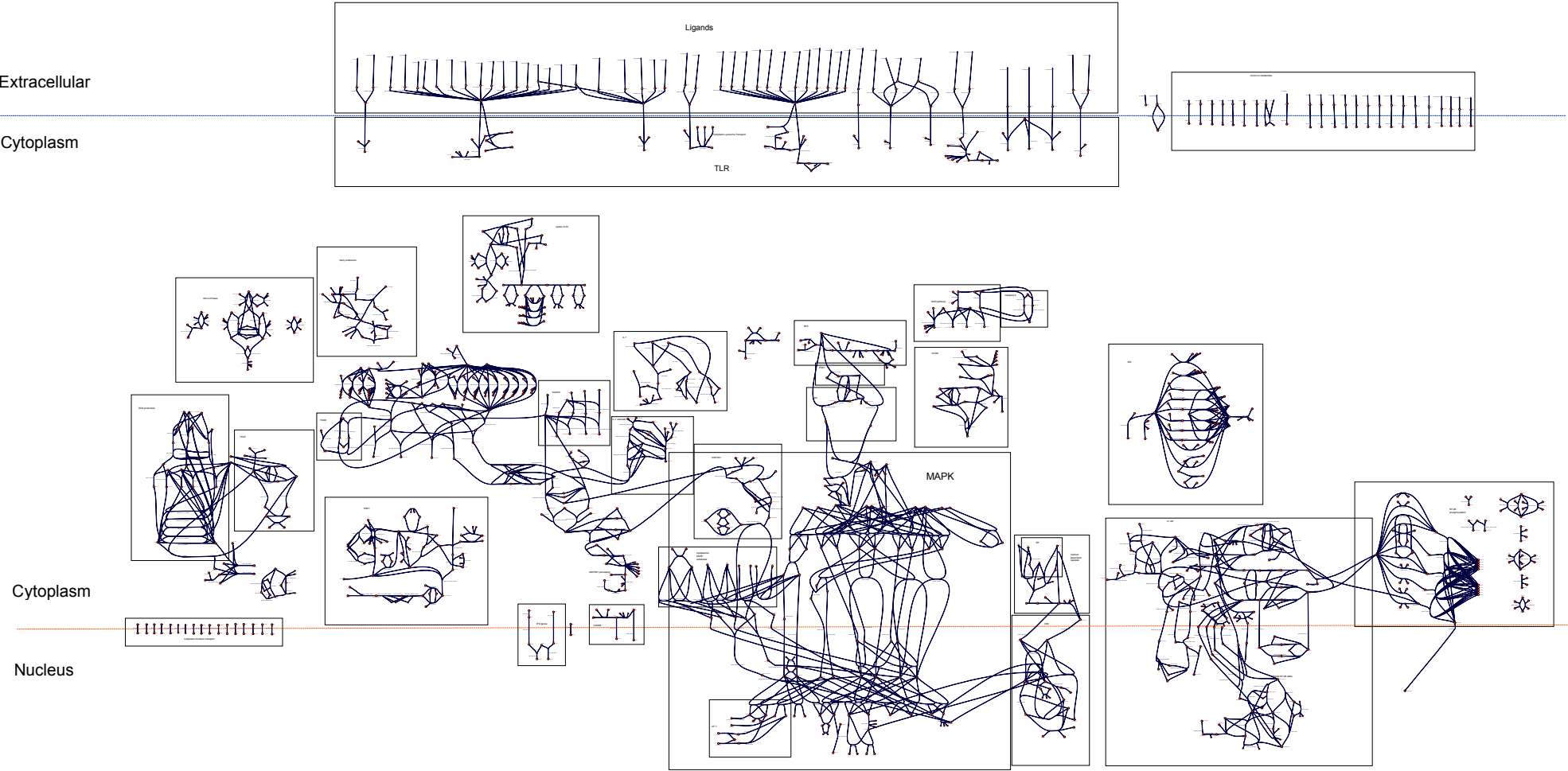

Supplement: Figure S1 — Map of the reconstructed TLR signaling network (3.29 MB PDF) [file pcbi.1000292.s002.pdf]
