# Supplementary material for: Identification of Potential Pathway Mediation Targets in Toll-like Receptor Signaling
Source: PLoS Comput Biol. 2009 Feb 20;5(2):e1000292. doi: 10.1371/journal.pcbi.1000292 (PMC2634968; doi:10.1371/journal.pcbi.1000292)
Supplement: Table S1 — TLR network species (0.39 MB PDF) [file pcbi.1000292.s003.pdf]

Table S1: TLR network species

* refers to Figure 2 in paper.

SPECIES NUMBER*	PARTICIPATION *	ABBREVIATION	NAME	COMPARTMENT
1	68	h(c)	H+	Cytosol
2	57	adp(c)	ADP	Cytosol
3	57	atp(c)	ATP	Cytosol
4	25	IKK(c)	inhibitor of kappa light polypeptide gene enhancer in B-cells kinase alpha(dimer)/beta(dimer)/gamma (tetramer) complex	Cytosol
5	23	UBIQ(c)	Ubiquitin	Cytosol
6	22	h2o(c)	H2O	Cytosol
7	21	IKK-2P(c)	inhibitor of kappa light polypeptide gene enhancer in B-cells kinase alpha(dimer)/beta(dimer)/gamma (tetramer) complex (4 phosphorylated)	Cytosol
8	18	TLRL2(e)	Toll-like receptor 2 ligand (generic)	Extra-organism
9	17	MYD88-D(c)	Myeloid differentiation primary response 88 (dimer)	Cytosol
10	17	pi(c)	Phosphate	Cytosol
11	16	IRAK1_TIFA-2P3U(c)	IRAK1/TIFA/TRAF6 (dimer)/Ubc13/Uev1A complex (2 phosphorylated	Cytosol
12	14	TIR(c)	Toll/interleukin-1 receptor domain	Cytosol
13	14	TRAF6-D(c)	tumor necrosis factor receptor associated factor 6 (dimer)	Cytosol
14	14	pail345p_hs(c)	phosphatidylinositol-3	Cytosol
15	14	TLRL4(e)	Toll-like receptor 4 ligand (generic)	Extra-organism
16	13	NFKB(p50/p65)(c)	NF-kappa-B (p50/p65) complex	Cytosol
17	12	TIR_MYD(c)	TIR/MyD88(dimer) complex	Cytosol
18	12	ca2(c)	Calcium	Cytosol
19	11	dag_hs(c)	diacylglycerol (homo sapiens)	Cytosol
20	11	CBP(n)	cAMP responsive element binding protein 1 binding protein	Nucleus
21	10	MAP3K7IP_TRAF6-2P3U(c)	MAP3K7/IP23/1/TRAF6 (dimer)/Ubc13/Uev1A complex (2 phosphorylated	Cytosol
22	10	MAP3K7IP_TRAF6-3P3U(c)	MAP3K7/IP23/1/TRAF6 (dimer)/Ubc13/Uev1A complex (3 phosphorylated	Cytosol
23	10	NFKB(p50)(c)	NF-kappa-B (p50)	Cytosol
24	10	TLR2(c)	Toll-like receptor 2	Cytosol
25	9	CD14(c)	CD14	Cytosol
26	9	PKCZ(c)	protein kinase C (zeta isoform)	Cytosol
27	9	ps_hs(c)	phosphatidylserine (homo sapiens)	Cytosol

28	9	MAPK14(n)	mitogen-activated protein kinase 14	Nucleus
29	9	MAPK14-2P(n)	mitogen-activated protein kinase 14 (2 phosphorylated)	Nucleus
30	8	NFKB_P5K(c)	NF-kB P5 kinase	Cytosol
31	8	NFKB_P5K-4P(c)	NF-kB P5 kinase (4 phosphorylated)	Cytosol
32	8	RIP2(c)	receptor-interacting serine-threonine kinase 2	Cytosol
33	8	TLRL2/6(e)	Toll-like receptor 2/6 ligand (generic)	Extra-organism
34	8	MAPK11(n)	mitogen-activated protein kinase 11	Nucleus
35	8	MAPK11-2P(n)	mitogen-activated protein kinase 11 (2 phosphorylated)	Nucleus
36	7	AKT(c)	Akt	Cytosol
37	7	AKT-2P(c)	Akt (2 phosphorylated)	Cytosol
38	7	IKKE(c)	inhibitor of kappa light polypeptide gene enhancer in B-cells kinase epsilon	Cytosol
39	7	IKKE-P(c)	inhibitor of kappa light polypeptide gene enhancer in B-cells kinase epsilon (phosphorylated)	Cytosol
40	7	IL1RAP(c)	interleukin 1 receptor accessory protein	Cytosol
41	7	MAP2K4(c)	mitogen-activated protein kinase kinase 4	Cytosol
42	7	MAP2K4-2P(c)	mitogen-activated protein kinase kinase 4 (2 phosphorylated)	Cytosol
43	7	MAP3K5(c)	mitogen-activated protein kinase kinase kinase 5	Cytosol
44	7	NFKB(p105)(c)	NF-kappa-B (p105)	Cytosol
45	7	NFKB_P3K(c)	NF-kB P3 kinase	Cytosol
46	7	NFKB_P3K-4P(c)	NF-kB P3 kinase (4 phosphorylated)	Cytosol
47	7	P47PHOX(c)	p47-phox protein	Cytosol
48	7	PKAC(c)	protein kinase A	Cytosol
49	7	PKCZ-P(c)	protein kinase C (zeta isoform) (phosphorylated)	Cytosol
50	7	RAC1_GTP(c)	Rho family small GTP-binding protein Rac1/gtp complex	Cytosol
51	7	RAF1(c)	Raf-1	Cytosol
52	7	SRCK(c)	Src family kinase (generic)	Cytosol
53	7	MSK1(n)	mitogen- and stress-activated protein kinase 1	Nucleus
54	6	A1901_DEGR2(c)	A1901 degraded 2	Cytosol
55	6	BTRCP2(c)	beta-transducin repeat containing protein 2	Cytosol
56	6	MAP3K1-P(c)	mitogen-activated protein kinase kinase kinase 1 (phosphorylated)	Cytosol
57	6	MAP3K3(c)	mitogen-activated protein kinase kinase kinase 3	Cytosol

58	6	MAPK3/1(c)	mitogen-activated protein kinase 3/1	Cytosol
59	6	MAPK3/1-2P(c)	mitogen-activated protein kinase 3/1 (2 phosphorylated)	Cytosol
60	6	MD2(c)	MD-2	Cytosol
61	6	NFKB(p50/p65)-5P(c)	NF-kappa-B (p50/p65) complex (5 phosphorylated)	Cytosol
62	6	NFKB(p65)(c)	NF-kappa-B (p65)	Cytosol
63	6	NFKB_P1K(c)	NF-kB P1 kinase	Cytosol
64	6	NFKB_P1K-5P(c)	NF-kB P1 kinase (5 phosphorylated)	Cytosol
65	6	P47PHOX-8P(c)	p47-phox protein (8 phosphorylated)	Cytosol
66	6	PAK1(c)	p21/Cdc42/Rac1-activated kinase 1	Cytosol
67	6	PDK1(c)	phosphoinositide-dependent kinase 1	Cytosol
68	6	PDK1_pail345p_hs-P(c)	phosphoinositide-dependent kinase 1 (activated	Cytosol
69	6	RAB5_GTP(c)	Ras-associated GTP-binding protein Rab5/gtp complex	Cytosol
70	6	TIRAP(c)	toll-interleukin 1 receptor (TIR) domain containing adaptor protein	Cytosol
71	6	TLR10(c)	Toll-like receptor 10	Cytosol
72	6	gdp(c)	GDP	Cytosol
73	6	gtp(c)	GTP	Cytosol
74	6	pa_hs(c)	phosphatidic acid (homo sapiens)	Cytosol
75	6	CREB_CRE-P(n)	CREB(dimer)/CRE site complex (phosphorylated)	Nucleus
76	6	MAPK3/1-2PD(n)	mitogen-activated protein kinase 3/1 (2 phosphorylated	Nucleus
77	6	MAPK3/1-D(n)	mitogen-activated protein kinase 3/1 (dimer)	Nucleus
78	5	CDC42_GTP(c)	Cdc42/GTP complex	Cytosol
79	5	GENERIC_PP(c)	unknown phosphatase (generic)	Cytosol
80	5	GENERIC_PP-P(c)	unknown phosphatase (generic) (phosphorylated)	Cytosol
81	5	GSK3B(c)	glycogen synthase kinase 3 beta	Cytosol
82	5	GSK3B-P(c)	glycogen synthase kinase 3 beta (phosphorylated)	Cytosol
83	5	IL1R1(c)	interleukin 1 receptor	Cytosol
84	5	IL1R1/L/AP(c)	interleukin 1 receptor type 1/ligand/accessory protein complex	Cytosol
85	5	IL1R1_LIG(c)	interleukin 1 receptor	Cytosol
86	5	IRAK1_MAP3K3-4P3U(c)	IRAK1/TIFA/TRAF6 (dimer)/Ubc13/Uev1A/MAP3K3 complex (3 phosphorylated	Cytosol
87	5	IRAK1_TIFA-2P(c)	IRAK1/TIFA/TRAF6 (dimer)/Ubc13/Uev1A complex (2 phosphorylated)	Cytosol

88	5	MAP2K1(c)	mitogen-activated protein kinase kinase 1	Cytosol
89	5	MAP3K8(p58)-P(c)	mitogen-activated protein kinase kinase kinase 8 (p58) (phosphorylated)	Cytosol
90	5	MYD88_IRF7(c)	MyD88 (dimer)/TRAF6 (dimer)/IRF7 complex	Cytosol
91	5	NFKB(p105)-2PU(c)	NF-kappa-B (p105) (2 phosphorylated	Cytosol
92	5	NFKB(p50/p65)-P5(c)	NF-kappa-B (p50/p65) complex (phosphorylated) 5	Cytosol
93	5	NFKB_IKBA(c)	NF-kappa-B (p50/p65)/I-kappa-B-alpha complex	Cytosol
94	5	NFKB_P2K(c)	NF-kB P2 kinase	Cytosol
95	5	NFKB_P2K-P(c)	NF-kB P2 kinase (phosphorylated)	Cytosol
96	5	NFKB_P4K(c)	NF-kB P4 kinase	Cytosol
97	5	NFKB_P4K-P(c)	NF-kB P4 kinase (phosphorylated)	Cytosol
98	5	PAK1P-P(c)	p21/Cdc42/Rac1-activated kinase 1 (activated	Cytosol
99	5	PI3K1A(c)	phosphoinositide 3-kinase	Cytosol
100	5	PI3K1A-P(c)	phosphoinositide 3-kinase	Cytosol
101	5	RAS_GTP(c)	Ras family small GTP-binding protein (generic)/gtp complex	Cytosol
102	5	SIGIRR(c)	single immunoglobulin IL-1R-related molecule	Cytosol
103	5	SRCK-P(c)	Src family kinase (generic) (phosphorylated)	Cytosol
104	5	TBK1(c)	TRAF family member-associated NFKB activator-binding kinase 1	Cytosol
105	5	TBK1-P(c)	TRAF family member-associated NFKB activator-binding kinase 1 (phosphorylated)	Cytosol
106	5	TICAM1(c)	toll-like receptor adaptor molecule 1	Cytosol
107	5	TICAM1P(c)	toll-like receptor adaptor molecule 1 (activated)	Cytosol
108	5	TIFA(c)	tumor necrosis factor (TNF)-associated factor (TRAF)-interacting protein with a forkhead-associated domain	Cytosol
109	5	TIR_TIRAP(c)	TIRAP-bound toll-interleukin 1 receptor domain	Cytosol
110	5	TLR4(c)	Toll-like receptor 4	Cytosol
111	5	TOLLIP(c)	Toll interacting protein	Cytosol
112	5	mi145p(c)	1D-myo-Inositol 1	Cytosol
113	5	pail3p_hs(c)	1-Phosphatidyl-1D-myo-inositol 3-phosphate (Homo sapiens)	Cytosol
114	5	TLRL7(e)	Toll-like receptor 7 ligand (generic)	Extra-organism
115	5	CREB_CRE(n)	CREB (dimer)/CRE site complex	Nucleus

116	5	ELK1(n)	ELK1 (protein)	Nucleus
117	5	ELK1-2P(n)	ELK1 (2 phosphorylated)	Nucleus
118	5	FOS(n)	c-Fos (protein)	Nucleus
119	5	KB_SITE(n)	kappa-B site (gene)	Nucleus
120	5	MSK1-5P(n)	mitogen- and stress-activated protein kinase 1 (5 phosphorylated)	Nucleus
121	5	NFKB(p50/p65)-5P(n)	NF-kappa-B (p50/p65) complex (5 phosphorylated)	Nucleus
122	5	NFKB(p50/p65)-5P3A(n)	NF-kappa-B (p50/p65) complex (5 phosphorylated	Nucleus
123	5	NFKB(p50/p65)-5P5A(n)	NF-kappa-B (p50/p65) complex (5 phosphorylated	Nucleus
124	5	accoa(n)	Acetyl-CoA	Nucleus
125	5	coa(n)	Coenzyme A	Nucleus
126	5	h(n)	H+	Nucleus
127	4	14-3-3(c)	14-3-3	Cytosol
128	4	A20(c)	A20 (protein)	Cytosol
129	4	BTK(c)	Bruton agammaglobulinemia tyrosine kinase	Cytosol
130	4	BTK_pail345p_hs(c)	Bruton's tyrosine kinase/phosphoinositol 3	Cytosol
131	4	BTK_pail345p_hs-2P(c)	Bruton agammaglobulinemia tyrosine kinase (2 phosphorylated	Cytosol
132	4	CAM_CAMK2(c)	Calmodulin/calmodulin-dependent kinase 2 complex	Cytosol
133	4	CAM_CAMK2-P(c)	Calmodulin/calmodulin-dependent kinase 2 (phosphorylated)	Cytosol
134	4	CK2(c)	Casein kinase 2	Cytosol
135	4	CK2-P(c)	casein kinase 2 (phosphorylated)	Cytosol
136	4	IKBA(c)	I-kappa-B-alpha (protein)	Cytosol
137	4	IKBA-2P2U(c)	I-kappa-B-alpha (2 phosphorylated	Cytosol
138	4	IKBB(c)	I-kappa-B-beta	Cytosol
139	4	IRAK1(c)	interleukin-1 receptor associated kinase 1	Cytosol
140	4	IRAK1_MAP3K3-2P3U(c)	IRAK1/TIFA/TRAF6 (dimer)/Ubc13/Uev1A/MAP3K3 complex (2 phosphorylated	Cytosol
141	4	IRAK1_MAP3K5(c)	IRAK1/TIFA/TRAF6 (dimer)/Ubc13/Uev1A/MAP3K5 complex (3 phosphorylated	Cytosol
142	4	IRF7(c)	interferon regulatory factor 7	Cytosol
143	4	LBP(c)	lipopolysaccharide binding protein	Cytosol
144	4	MAP2K3(c)	mitogen-activated protein kinase kinase 3	Cytosol
145	4	MAP2K3-2P(c)	mitogen-activated protein kinase kinase 3 (2 phosphorylated)	Cytosol
146	4	MAP2K6(c)	mitogen-activated protein kinase kinase 6	Cytosol

147	4	MAP2K6-P(c)	mitogen-activated protein kinase kinase 6 (phosphorylated)	Cytosol
148	4	MAP2K7(c)	mitogen-activated protein kinase kinase 7	Cytosol
149	4	MAP2K7-2P(c)	mitogen-activated protein kinase kinase 7 (2 phosphorylation)	Cytosol
150	4	MAP3K14(c)	mitogen-activated protein kinase kinase kinase 14	Cytosol
151	4	MAP3K14-P(c)	mitogen-activated protein kinase kinase kinase 14 (phosphorylated)	Cytosol
152	4	MAP3K8(p52)(c)	mitogen-activated protein kinase kinase kinase 8 (p52)	Cytosol
153	4	MAP3K8(p58)(c)	mitogen-activated protein kinase kinase kinase 8 (p58)	Cytosol
154	4	MAPK14(c)	mitogen-activated protein kinase 14	Cytosol
155	4	MAPK14-2P(c)	mitogen-activated protein kinase 14 (2 phosphorylated)	Cytosol
156	4	MBP(c)	myelin basic protein	Cytosol
157	4	MBP-P(c)	myelin basic protein (phosphorylated)	Cytosol
158	4	MKNK1(c)	MAP kinase interacting serine/threonine kinase 1	Cytosol
159	4	MKNK1-P(c)	MAP kinase interacting serine/threonine kinase 1	Cytosol
160	4	NFKB(p105)-2P(c)	NF-kappa-B (p105) (2 phosphorylated)	Cytosol
161	4	NFKB(p50/p65)-P(c)	NF-kappa-B (p50/p65) complex (phosphorylated)	Cytosol
162	4	NFKB(p50/p65)-P3(c)	NF-kappa-B (p50/p65) complex (phosphorylated) 3	Cytosol
163	4	NFKB_IKBA-2P(c)	NF-kappa-B (p50/p65)/I-kappa-B-alpha complex (2 phosphorylated)	Cytosol
164	4	NFKB_IKBA-2P2U(c)	NF-kappa-B (p50/p65)/i-kappa-B-alpha complex (2 phosphorylated	Cytosol
165	4	NOD1(c)	nucleotide-binding oligomerization domain protein 1	Cytosol
166	4	NOD1P(c)	nucleotide-binding oligomerization domain protein 1 (activated)	Cytosol
167	4	PKAC-P(c)	protein kinase A	Cytosol
168	4	PKR-D(c)	double stranded RNA-dependent protein kinase (dimer)	Cytosol
169	4	PP2A_ABC(c)	Protein Phosphatase 2A (A	Cytosol
170	4	PP2A_ABC-P(c)	Protein phosphatase 2A (A	Cytosol
171	4	TIR_MYD_IRAK4(c)	TIR/MyD88(dimer)/IRAK4 complex	Cytosol
172	4	TIR_TIRAP_IRAK4(c)	TIR/TIRAP/MyD88(dimer)/IRAK4 complex	Cytosol

173	4	TIR_TIRAP_MYD(c)	TIR domain/TIRAP/MyD88(dimer) complex	Cytosol
174	4	TLR1(c)	Toll-like receptor 1	Cytosol
175	4	TLR2/L-D(c)	Toll-like receptor 2 (dimer) ligand/CD14 complex	Cytosol
176	4	TLR4/L_MD2-D(c)	Toll-like receptor 4 (dimer) ligand/MD-2/CD14/LBP complex	Cytosol
177	4	TLR5(c)	Toll-like receptor 5	Cytosol
178	4	TLRL2(c)	Toll-like receptor 2 ligand (generic)	Cytosol
179	4	TOLLIP-P(c)	Toll interacting protein (phosphorylated)	Cytosol
180	4	TRAF6(c)	Tumor necrosis factor receptor associated factor 6	Cytosol
181	4	TRD3A(c)	Triad3A protein	Cytosol
182	4	TRIP6(c)	thyroid hormone receptor interactor 6	Cytosol
183	4	UBC13(c)	ubiquitin-conjugating enzyme E2N (UBC13 homolog)	Cytosol
184	4	UEV1A(c)	ubiquitin-conjugating enzyme E2 variant 1	Cytosol
185	4	VAV1(c)	vav 1 proto-oncogene	Cytosol
186	4	VAV1-P(c)	vav 1 proto-oncogene (phosphorylated)	Cytosol
187	4	ac(c)	Acetate	Cytosol
188	4	pail45p_hs(c)	phosphatidylinositol 4	Cytosol
189	4	26dap-LL(e)	LL-2	Extra-organism
190	4	IL1R1_LIG(e)	interleukin 1 receptor	Extra-organism
191	4	ATF2(n)	Activating transcription factor 2	Nucleus
192	4	ATF2-P(n)	Activating transcription factor 2 (phosphorylated)	Nucleus
193	4	CAM_CAMK2(n)	Calmodulin/calmodulin-dependent kinase 2 complex	Nucleus
194	4	CAM_CAMK2-P(n)	Calmodulin/calmodulin-dependent kinase 2 (phosphorylated)	Nucleus
195	4	CRE_GENE(n)	cAMP responsive element site	Nucleus
196	4	FOS-6P(n)	c-Fos (6 phosphorylated)	Nucleus
197	4	IKBA(n)	I-kappa-B-alpha (protein)	Nucleus
198	4	MAPK8(n)	mitogen-activated protein kinase 8	Nucleus
199	4	MAPK8-2P(n)	mitogen-activated protein kinase 8 (2 phosphorylated)	Nucleus
200	4	MAPKAPK2(n)	mitogen-activated protein kinase activated protein kinase 2	Nucleus
201	4	MAPKAPK2-2P(n)	mitogen-activated protein kinase activated protein kinase 2 (2 phosphorylated)	Nucleus
202	4	MSK1-2P(n)	mitogen- and stress-activated protein kinase 1 (2 phosphorylated)	Nucleus
203	4	UBIQ(n)	Ubiquitin	Nucleus

204	4	adp(n)	ADP	Nucleus
205	4	atp(n)	ATP	Nucleus
206	4	h2o(n)	H2O	Nucleus
207	4	TLR9(v)	Toll-like receptor 9	Vacuole
208	4	TLR9/L-D(v)	Toll-like receptor 9 (dimer) ligand complex	Vacuole
209	3	26dap-LL(c)	LL-2	Cytosol
210	3	A2129_DEGR(c)	A2129 (degraded)	Cytosol
211	3	BCL3(c)	B-cell CLL/lymphoma 3	Cytosol
212	3	CASP9(c)	caspase 9	Cytosol
213	3	DSRNA(c)	Double stranded RNA	Cytosol
214	3	EEA1(c)	early endosome antigen 1	Cytosol
215	3	FADD(c)	Fas (TNFRSF6)-associated via death domain	Cytosol
216	3	FADD_PCASP8(c)	Fas-associated death domain/pro-caspase 8 complex	Cytosol
217	3	GENERIC_DA(c)	unknown deacetylase (generic)	Cytosol
218	3	GENERIC_DA-A(c)	unknown deacetylase (generic) (acetylated)	Cytosol
219	3	IKK-P(c)	inhibitor of kappa light polypeptide gene enhancer in B-cells kinase alpha(dimer)/beta(dimer)/gamma (tetramer) complex (2 phosphorylated)	Cytosol
220	3	IL1R1/L/AP-P(c)	interleukin 1 receptor type 1/ligand/accessory protein complex (phosphorylated)	Cytosol
221	3	IRAK1-2PU(c)	interleukin-1 receptor associated kinase 1 (2 phosphorylated	Cytosol
222	3	IRAK1C_TOLLIP(c)	IRAK1C/TOLLIP complex	Cytosol
223	3	IRAK1_MAP3K3-3P3U(c)	IRAK1/TIFA/TRAF6 (dimer)/Ubc13/Uev1A/MAP3K3 complex (3 phosphorylated	Cytosol
224	3	IRAK1_TOLLIP(c)	IRAK1/TOLLIP complex	Cytosol
225	3	IRAK4(c)	Interleukin-1 receptor associated kinase 4	Cytosol
226	3	IRAK_MYD_TIR-2P(c)	IRAK1/IRAK4/MyD88 (dimer)/TIR complex (2 phosphorylated)	Cytosol
227	3	IRF3(c)	interferon regulatory factor 3	Cytosol
228	3	IRF3-2P(c)	interferon regulatory factor 3 (2 phosphorylated)	Cytosol
229	3	IRF7-2P(c)	interferon regulatory factor 7 (2 phosphorylated)	Cytosol
230	3	KSR1_RAF1_MAPK3/1-4P(c)	KSR1/MAP2K1/MAPK3/1/Raf-1 complex (4 phosphorylated)	Cytosol
231	3	KSR1_RAF1_MAPK3/1-5P(c)	KSR1/MAP2K1/MAPK3/1/Raf-1 complex (5 phosphorylated)	Cytosol
232	3	MAP2K1-2P(c)	mitogen-activated protein kinase kinase 1 (2 phosphorylated)	Cytosol

233	3	MAP3K1(c)	mitogen-activated protein kinase kinase kinase 1	Cytosol
234	3	MAP3K5-P(c)	mitogen-activated protein kinase kinase kinase 5 (phosphorylated)	Cytosol
235	3	MAP3K7IP_TRAF6-5P3U(c)	MAP3K7/IP23/1/TRAF6 (dimer)/Ubc13/Uev1A complex (5 phosphorylated	Cytosol
236	3	MAP3K7IP_TRAF6-6P3U(c)	MAP3K7/IP23/1/TRAF6 (dimer)/Ubc13/Uev1A complex (6 phosphorylated	Cytosol
237	3	MAPK11(c)	mitogen-activated protein kinase 11	Cytosol
238	3	MAPK11-2P(c)	mitogen-activated protein kinase 11 (2 phosphorylated)	Cytosol
239	3	MAPKAPK2-2P(c)	mitogen-activated protein kinase activated protein kinase 2 (2 phosphorylated)	Cytosol
240	3	MSK1(c)	mitogen- and stress-activated protein kinase 1	Cytosol
241	3	MSK1-5P(c)	mitogen- and stress-activated protein kinase 1 (5 phosphorylated)	Cytosol
242	3	MYD88(c)	Myeloid differentiation primary response 88	Cytosol
243	3	NFKB(p50)-PD(c)	NF-kappa-B (p50) (phosphorylated	Cytosol
244	3	NFKB_PKAC(c)	NF-kappa-B (p50/p65)/protein kinase A (catalytic subunit) complex	Cytosol
245	3	NOD2(c)	nucleotide-binding oligomerization domain protein 2	Cytosol
246	3	NOD2P(c)	nucleotide-binding oligomerization domain protein 2 (activated)	Cytosol
247	3	NSF(c)	N-ethylmaleimide-sensitive factor	Cytosol
248	3	P40PHOX(c)	p40-phox protein	Cytosol
249	3	P67PHOX(c)	p67-phox protein	Cytosol
250	3	PAK1P(c)	p21/Cdc42/Rac1-activated kinase 1 (activated)	Cytosol
251	3	PCASP8(c)	procaspase 8	Cytosol
252	3	PCASP9(c)	procaspase 9	Cytosol
253	3	PKCA(c)	protein kinase C	Cytosol
254	3	PKCA-3P(c)	protein kinase C	Cytosol
255	3	PKCB2(c)	protein kinase C	Cytosol
256	3	PKCB2-3P(c)	protein kinase C	Cytosol
257	3	PKR(c)	double stranded RNA-dependent protein kinase	Cytosol
258	3	PKR-2PD(c)	double stranded RNA-dependent protein kinase (2 phosphorylated	Cytosol
259	3	PLNO1(c)	Pellino 1	Cytosol
260	3	PRE_IL1A(c)	pre interleukin 1 alpha	Cytosol

261	3	RAB5_GDP(c)	Ras-associated GTP-binding protein Rab5/gdp complex	Cytosol
262	3	RAC1_GDP(c)	Rho family small GTP-binding protein Rac1/gdp complex	Cytosol
263	3	RAF1-2P(c)	Raf-1 (2 phosphorylated)	Cytosol
264	3	RAF1-4P(c)	Raf-1 (4 phosphorylated)	Cytosol
265	3	RAF1-P(c)	Raf-1 (phosphorylated)	Cytosol
266	3	RAS_GDP(c)	Ras family small GTP-binding protein (generic)/gdp complex	Cytosol
267	3	RIP1(c)	receptor (TNFRSF)-interacting serine-threonine kinase 1	Cytosol
268	3	RKIP(c)	Raf kinase inhibitory protein	Cytosol
269	3	STLR2(c)	soluble TLR2	Cytosol
270	3	STXN13(c)	Syntaxin 13	Cytosol
271	3	TLRL3(c)	Toll-like receptor 3 ligand (generic)	Cytosol
272	3	TLRL4(c)	Toll-like receptor 4 ligand (generic)	Cytosol
273	3	TLRL7(c)	Toll-like receptor 7 ligand (generic)	Cytosol
274	3	TLRL8(c)	Toll-like receptor 8 ligand (generic)	Cytosol
275	3	TLRL9(c)	Toll-like receptor 9 ligand (generic)	Cytosol
276	3	TRAF6-UD(c)	tumor necrosis factor (TNF) receptor-associated factor 6 (ubiquitinated	Cytosol
277	3	TRXOX(c)	oxidized thioredoxin (Homo sapiens)	Cytosol
278	3	TRXRED(c)	reduced thioredoxin (Homo sapiens)	Cytosol
279	3	h2o2(c)	Hydrogen peroxide	Cytosol
280	3	DSRNA(e)	Double stranded RNA	Extra-organism
281	3	HSP70(e)	Heat shock protein 70	Extra-organism
282	3	IMQ(e)	Imidazoquinoline	Extra-organism
283	3	LPS_HS(e)	Lipopolysaccharide (Homo sapiens)	Extra-organism
284	3	LTA(e)	Lipoteichoic acid	Extra-organism
285	3	MRAP(e)	Mannuronic acid polymer	Extra-organism
286	3	PSM(e)	Phenol-soluble modulin	Extra-organism
287	3	SSRNA(e)	Single stranded RNA	Extra-organism
288	3	TLRL1/2(e)	Toll-like receptor 1/2 ligand (generic)	Extra-organism
289	3	TLRL11(e)	Toll-like receptor 11 ligand (generic)	Extra-organism
290	3	TLRL3(e)	Toll-like receptor 3 ligand (generic)	Extra-organism
291	3	TLRL8(e)	Toll-like receptor 8 ligand (generic)	Extra-organism
292	3	TLRL9(e)	Toll-like receptor 9 ligand (generic)	Extra-organism
293	3	ZMS(e)	Zymosan	Extra-organism
294	3	ac(e)	Acetate	Extra-organism
295	3	h(e)	H+	Extra-organism
296	3	TLR3(l)	Toll-like receptor 3	Lysosome

297	3	TLR3/L-D(l)	Toll-like receptor 3 (dimer) ligand complex	Lysosome
298	3	AP1_GENE(n)	AP-1 (gene)	Nucleus
299	3	CREB_CBP(n)	CREB (dimer)/CBP complex (2 phosphorylated)	Nucleus
300	3	CREB_CRE-2P(n)	CREB/CRE site complex (2 phosphorylated)	Nucleus
301	3	HDAC3(n)	histone deacetylase 3	Nucleus
302	3	HDAC3-A(n)	histone deacetylase 3 (acetylated)	Nucleus
303	3	ISRE(n)	interferon stimulated response element	Nucleus
304	3	JUN(n)	c-Jun (protein)	Nucleus
305	3	JUN-2P(n)	c-Jun (2 phosphorylated)	Nucleus
306	3	MAP2K3(n)	mitogen-activated protein kinase kinase 3	Nucleus
307	3	MAP2K3-2P(n)	mitogen-activated protein kinase kinase 3 (2 phosphorylated)	Nucleus
308	3	MAP2K6(n)	mitogen-activated protein kinase kinase 6	Nucleus
309	3	MAP2K6-P(n)	mitogen-activated protein kinase kinase 6 (phosphorylated)	Nucleus
310	3	MAPKAPK2-P(n)	mitogen-activated protein kinase activated protein kinase 2 (phosphorylated)	Nucleus
311	3	NFKB(p50)(n)	NF-kappa-B (p50)	Nucleus
312	3	NFKB(p50)-PD(n)	NF-kappa-B (p50) (phosphorylated	Nucleus
313	3	NFKB(p50)_BCL3(n)	NF-kappa-B (p50 dimer)/Bcl-3 complex (2 phosphorylated)	Nucleus
314	3	NFKB(p50/p65)_CBP-5P(n)	NF-kappa-B (p50/p65)/CBP complex (5 phosphorylated)	Nucleus
315	3	NFKB_CBP_KB(n)	NF-kappa-B (p50/p65)/CREB binding protein/kB site complex (phosphorylated	Nucleus
316	3	NFKB_IKBA-2P(n)	NF-kappa-B (p50/p65)/I-kappa-B-alpha complex (2 phosphorylated)	Nucleus
317	3	NFKB_IKBA-2P2U(n)	NF-kappa-B (p50/p65)/i-kappa-B-alpha complex (2 phosphorylated	Nucleus
318	3	PKA(n)	protein kinase A	Nucleus
319	3	PKA-2P(n)	protein kinase A (2 phosphorylated)	Nucleus
320	3	PP2A_ABC(n)	Protein Phosphatase 2A (A	Nucleus
321	3	PP2A_ABC-P(n)	Protein phosphatase 2A (A	Nucleus
322	3	pi(n)	Phosphate	Nucleus
323	3	TLR9(r)	Toll-like receptor 9	Endoplasmic Reticulum
324	3	ca2(r)	Calcium	Endoplasmic Reticulum
325	3	GP91_P22(v)	gp91/p22-phox protein complex	Vacuole
326	3	NSF_EEA1_RAB5(v)	NSF/Syntaxin 13/EEA1 (dimer)/PI3P/Rab5/GTP complex	Vacuole

327	3	TLR7(v)	Toll-like receptor 7	Vacuole
328	3	TLR7/L-D(v)	Toll-like receptor 7 (dimer) ligand complex	Vacuole
329	3	TLR8(v)	Toll-like receptor 8	Vacuole
330	3	TLR8/L-D(v)	Toll-like receptor 8 (dimer) ligand complex	Vacuole
331	2	14-3-3_KSR1(c)	14-3-3/KSR1/MAP2K1 complex	Cytosol
332	2	14-3-3_RAF1(c)	14-3-3/Raf-1 complex (phosphorylated)	Cytosol
333	2	A001_DEGR(c)	A001 degraded	Cytosol
334	2	A1115_DEGR(c)	A1115 (degraded)	Cytosol
335	2	A1146_DEGR(c)	A1146 (degraded)	Cytosol
336	2	A1159_DEGR(c)	A1159 (degraded)	Cytosol
337	2	A153_DEGR(c)	A153 (degraded)	Cytosol
338	2	A1901_DEGR(c)	A1901 (degraded)	Cytosol
339	2	A2069_DEGR(c)	A2069 (degraded)	Cytosol
340	2	A2156_DEGR(c)	A2156 (degraded)	Cytosol
341	2	A2382_DEGR(c)	A2382 (degraded)	Cytosol
342	2	A385_DEGR(c)	A385 (degraded)	Cytosol
343	2	A399_DEGR(c)	A399 (degraded)	Cytosol
344	2	A399_DEGR2(c)	A399 (degraded) (unphosphorylated)	Cytosol
345	2	A454_DEGR(c)	A454 (degraded)	Cytosol
346	2	A523_DEGR(c)	A523 (degraded)	Cytosol
347	2	A796_DEGR(c)	A796 (degraded)	Cytosol
348	2	A839_DEGR(c)	A839 (degraded)	Cytosol
349	2	ABIN(c)	A20-binding inhibitor of NF-kB activation	Cytosol
350	2	ABIN_A20(c)	ABIN/A20 complex	Cytosol
351	2	AJUBA(c)	Ajuba	Cytosol
352	2	AJUBA_CPX-2P3U(c)	Ajuba/p62/IRAK1/TRAF6 (dimer)/TIFA/Ubc13/Uev1A/PKCz complex (2 phos, 3 ubi)	Cytosol
353	2	AJUBA_CPX-3P3U(c)	Ajuba/p62/IRAK1/TRAF6 (dimer)/TIFA/Ubc13/Uev1A/PKCz complex (3 phos, 3 ubi)	Cytosol
354	2	AKT_pail345p_hs(c)	Akt/phosphatidylinositol 3-phosphate complex	Cytosol
355	2	AKT_pail345p_hs-2P(c)	Akt/phosphatidylinositol 3-phosphate complex (2 phosphorylated)	Cytosol
356	2	AKT_pail345p_hs-P(c)	Akt/phosphatidylinositol 3-phosphate complex (phosphorylated)	Cytosol
357	2	BTK-2PU(c)	Bruton agammaglobulinemia tyrosine kinase (2 phosphorylated	Cytosol
358	2	BTK_CBL(c)	BTK/c-Cbl complex	Cytosol
359	2	BTK_IBTK(c)	BTK/iBTK complex	Cytosol
360	2	BTK_SH3BP5(c)	BTK/SH3BP5 complex	Cytosol
361	2	BTK_pail345p_hs-P(c)	Bruton agammaglobulinemia tyrosine kinase (1 phosphorylated)	Cytosol

362	2	CALPN(c)	calpain	Cytosol
363	2	CALPN_3ca2(c)	calpain/ca2 complex	Cytosol
364	2	CAM(c)	Calmodulin 1	Cytosol
365	2	CAMK2(c)	Calmodulin-dependent kinase 2	Cytosol
366	2	CAM_3ca2(c)	Calmodulin (activated)	Cytosol
367	2	CASP1(c)	caspase 1	Cytosol
368	2	CASP3(c)	caspase 3	Cytosol
369	2	CASP8(c)	caspase 8	Cytosol
370	2	CBL(c)	c-Cbl	Cytosol
371	2	CBL-P(c)	c-Cbl (phosphorylated)	Cytosol
372	2	CDC42_GDP(c)	Cdc42/GDP complex	Cytosol
373	2	CDC42_VAV1(c)	Cdc42/Vav1 complex	Cytosol
374	2	CTAK1(c)	MAP/microtubule affinity-regulating kinase 3	Cytosol
375	2	CTAK1-P(c)	MAP/microtubule affinity-regulating kinase 3 (phosphorylated)	Cytosol
376	2	CYLD(c)	cylindromatosis (turban tumor syndrome)	Cytosol
377	2	DAGK(c)	diacylglycerol kinase (generic)	Cytosol
378	2	DAGK-P(c)	diacylglycerol kinase (phosphorylated)	Cytosol
379	2	ECSIT(c)	ECSIT	Cytosol
380	2	EEA1-D(c)	early endosome antigen 1 (dimer)	Cytosol
381	2	EIF2A(c)	eukaryotic translation initiation factor 2	Cytosol
382	2	EIF2A-P(c)	eukaryotic translation initiation factor 2 (phosphorylated)	Cytosol
383	2	EIF4E(c)	eukaryotic translation initiation factor 4E	Cytosol
384	2	EIF4E-P(c)	eukaryotic translation initiation factor 4E (phosphorylated)	Cytosol
385	2	FMAP3K1(c)	Full length mitogen activated protein kinase kinase kinase 1	Cytosol
386	2	GENERIC_K(c)	unknown kinase (generic)	Cytosol
387	2	GENERIC_K-P(c)	unknown kinase (generic) (phosphorylated)	Cytosol
388	2	GP91PHOX(c)	gp91-phox protein	Cytosol
389	2	GSK3B-2P(c)	glycogen synthase kinase 3 beta (2 phosphorylated)	Cytosol
390	2	HSP27(c)	heat shock protein 27kDa	Cytosol
391	2	HSP27-3P(c)	heat shock protein 27kDa (3 phosphorylated)	Cytosol
392	2	IBTK(c)	inhibitor of Bruton agammaglobulinemia tyrosine kinase	Cytosol
393	2	IKBB-2P(c)	I-kappa-B-beta (2 phosphorylated)	Cytosol
394	2	IKBB-2P2U(c)	I-kappa-B-beta (2 phosphorylated	Cytosol
395	2	IKBB-2PU(c)	I-kappa-B-beta (2 phosphorylated	Cytosol

396	2	IKK-3P(c)	inhibitor of kappa light polypeptide gene enhancer in B-cells kinase alpha(dimer)/beta(dimer)/gamma (tetramer) complex (6 phosphorylated)	Cytosol
397	2	IKK_PKR(c)	IKK/PKR (dimer) complex	Cytosol
398	2	IKK_PKR_TRAF6(c)	IKK/PKR (dimer)/TRAF6 (dimer) complex	Cytosol
399	2	IKK_RIP1_TICAM1P(c)	IKK/RIP1/TICAM1P complex	Cytosol
400	2	IKK_RIP2_NOD1P(c)	IKK/RIP2/NOD1P complex	Cytosol
401	2	IKK_RIP2_NOD2P(c)	IKK/RIP2/NOD2P complex	Cytosol
402	2	IKK_RIP2_TRIP6_TRAF2(c)	IKK/RIP2/TRIP6/TRAF2 complex	Cytosol
403	2	IKK_SRC(c)	inhibitor of kappa light polypeptide gene enhancer in B-cells kinase alpha/beta/gamma(dimer)/c-Src complex	Cytosol
404	2	IL1A(c)	interleukin 1 alpha	Cytosol
405	2	IL1B(c)	interleukin 1 beta	Cytosol
406	2	IL1R1/L(c)	interleukin 1 receptor type 1/ligand complex	Cytosol
407	2	IL1R2(c)	interleukin 1 receptor	Cytosol
408	2	IL1R2/AP(c)	interleukin 1 receptor type 2/accessory protein complex	Cytosol
409	2	IMPA3(c)	importin alpha 3	Cytosol
410	2	IRAK1C(c)	Interleukin-1 receptor associated kinase 1c	Cytosol
411	2	IRAK1_ECSIT(c)	IRAK1/TIFA/TRAF6 (dimer)/Ubc13/Uev1A/ECSIT complex (2 phosphorylated	Cytosol
412	2	IRAK1_ECSIT_FMAP3K1(c)	IRAK1/TIFA/TRAF6 (dimer)/Ubc13/Uev1A/ECSIT/full length MAP3K1/caspase3 complex (2 phosphorylated	Cytosol
413	2	IRAK1_MAP3K7-2P3U(c)	IRAK1/TIFA/TRAF6 (dimer)/Ubc13/Uev1A/MAP3K7/IP123 complex (2 phosphorylated	Cytosol
414	2	IRAK1_MAP3K7-4P3U(c)	IRAK1/TIFA/TRAF6 (dimer)/Ubc13/Uev1A/MAP3K7/IP123 complex (4 phosphorylated	Cytosol
415	2	IRAK1_PLNO3(c)	IRAK1/Pellino3 complex	Cytosol
416	2	IRAK1_TIFA_A20(c)	IRAK1/TIFA/TRAF6 (dimer)/Ubc13/Uev1A/A20 complex (2 phosphorylated	Cytosol
417	2	IRAK1_TIFA_CYLD(c)	IRAK1/TIFA/TRAF6 (dimer)/Ubc13/Uev1A/CYLD complex (2 phosphorylated	Cytosol
418	2	IRAK2(c)	Interleukin-1 receptor associated kinase 2	Cytosol
419	2	IRAK_MYD_TIR(c)	IRAK1/IRAK4/MyD88 (dimer)/TIR complex	Cytosol

420	2	IRAK_MYD_TIR-P(c)	IRAK1/IRAK4/MyD88 (dimer)/TIR complex (phosphorylated)	Cytosol
421	2	IRAK_MYD_TIRAP-2P(c)	TIR/TIRAP/MyD88(dimer)/IRAK1/IRAK4 complex (diphosphorylated)	Cytosol
422	2	IRAK_TOLLIP_TIR(c)	IRAK4/IRAK1c/MyD88(dimer)/TIR/TOLLIP/TRAF6-D complex	Cytosol
423	2	IRAK_TRAF6(c)	IRAK1/IRAK4/MyD88 (dimer)/TIR/TRAF6 (dimer)/Ubc13/Uev1A/TIFA complex (2 phosphorylated)	Cytosol
424	2	IRAK_TRAF6_TIRAP(c)	IRAK1/TIFA/TIR/IRAK4/MyD88(dimer)/TIRAP/TRAF6 (dimer)/Ubc13/Uev1A (2 phosphorylated)	Cytosol
425	2	IRF3-2PD(c)	interferon regulatory factor 3 (2 phosphorylated	Cytosol
426	2	KSR1(c)	kinase suppressor of ras 1	Cytosol
427	2	KSR1-P(c)	kinase suppressor of ras 1 (phosphorylated)	Cytosol
428	2	KSR1_MAP2K1(c)	KSR1/MAP2K1 complex	Cytosol
429	2	KSR1_RAF1-7P(c)	KSR1/MAP2K1/Raf-1 complex (7 phosphorylated)	Cytosol
430	2	KSR1_RAF1_MAPK3/1-7P(c)	KSR1/MAP2K1/MAPK3/1/Raf-1 complex (7 phosphorylated)	Cytosol
431	2	KSR1_RAF1_MAPK3/1-9P(c)	KSR1/MAP2K1/MAPK3/1/Raf-1 complex (9 phosphorylated)	Cytosol
432	2	KSR2(c)	kinase suppressor of ras 2	Cytosol
433	2	MAP3K1-PU(c)	mitogen-activated protein kinase kinase kinase 1 (phosphorylated	Cytosol
434	2	MAP3K5-2P(c)	mitogen-activated protein kinase kinase kinase 5 (2 phosphorylated)	Cytosol
435	2	MAP3K5_TRXRED(c)	MAP3K5/reduced thioredoxin complex	Cytosol
436	2	MAP3K7(c)	mitogen-activated protein kinase kinase kinase 7	Cytosol
437	2	MAP3K7/IP123(c)	MAP3K7/MAP3K7IP1/MAP3K7IP2/3 complex	Cytosol
438	2	MAP3K7IP1(c)	mitogen-activated protein kinase kinase kianse 7 interacting protein 1	Cytosol
439	2	MAP3K7IP2/3(c)	mitogen-activated protein kinase kinase kinase 7 interacting protein 2/3	Cytosol
440	2	MAP3K7IP_TRAF6-2P2U(c)	MAP3K7IP23/1/MAP3K7/TRAF6 (dimer)/Ubc13/Uev1A complex (2 phosphorylated	Cytosol
441	2	MAP3K8(p52)_NFKB(c)	mitogen-activated protein kinase kinase kinase 8 (p52)/NFkB (p105) complex	Cytosol
442	2	MAP3K8(p58)_KSR2(c)	mitogen-activated protein kinase kinase kinase 8 (p58)/KSR2 complex (phosphorylated)	Cytosol

443	2	MAP3K8_NFKB(c)	mitogen-activated protein kinase kinase kinase 8 (p52/p58)/NFkB (p105) complex	Cytosol
444	2	MAP3K8_NFKB-P(c)	mitogen-activated protein kinase kinase kinase 8 (p52/p58)/NFkB (p105) complex (phosphorylated)	Cytosol
445	2	MAPK3/1-2PD(c)	mitogen-activated protein kinase 3/1 (2 phosphorylated	Cytosol
446	2	MAPK8(c)	mitogen-activated protein kinase 8	Cytosol
447	2	MAPK8-2P(c)	mitogen-activated protein kinase 8 (2 phosphorylated)	Cytosol
448	2	MAPK8-P(c)	mitogen-activated protein kinase 8 (phosphorylated)	Cytosol
449	2	MAPKAPK2(c)	mitogen-activated protein kinase activated protein kinase 2	Cytosol
450	2	MD2-D(c)	MD-2 (dimer)	Cytosol
451	2	MKNK2(c)	MAP kinase interacting serine/threonine kinase 2	Cytosol
452	2	MKNK2-P(c)	MAP kinase interacting serine/threonine kinase 2	Cytosol
453	2	MYD88S(c)	Myeloid differentiation primary response 88 short	Cytosol
454	2	MYD88_IRAK_TOLLIP(c)	MyD88 (dimer)/TIR/TOLLIP/IRAK1/IRAk 4 complex	Cytosol
455	2	MYD88_IRAK_TOLLIP-2P(c)	MyD88 (dimer)/TIR/TOLLIP/IRAK1/IRAK 4 (2 phosphorylated)	Cytosol
456	2	MYD88_IRAK_TOLLIP-P(c)	MyD88 (dimer)/TIR/TOLLIP/IRAK1/IRAK 4 complex (phosphorylated)	Cytosol
457	2	MYD88_MyD88S(c)	MyD88/MyD88s complex	Cytosol
458	2	NFKB(p105)_BTRCP2(c)	NF-kB(p105)-beta TrCP2 complex	Cytosol
459	2	NFKB(p105)_UBCH5A(c)	NF-kB(p105)-UbcH5A complex	Cytosol
460	2	NFKB(p50)_BCL3(c)	NF-kappa-B (p50 dimer)/Bcl-3 complex (2 phosphorylated)	Cytosol
461	2	NFKB(p50)_IMPA3(c)	NF-kB(p50)-importin alpha 3 complex	Cytosol
462	2	NFKB(p50/p105)(c)	NF-kappa-B (p50/p105) complex	Cytosol
463	2	NFKB(p50/p65)-P2(c)	NF-kappa-B (p50/p65) complex (phosphorylated) 2	Cytosol
464	2	NFKB(p50/p65)-P4(c)	NF-kappa-B (p50/p65) complex (phosphorylated) 4	Cytosol
465	2	NFKB(p65)_SOCS1(c)	NF-kB (p65)-SOCS1 complex	Cytosol
466	2	NFKB_FRAG(c)	NF-kB processing fragment	Cytosol
467	2	NFKB_FRAG2(c)	NF-kB fragment 2	Cytosol
468	2	NFKB_IKBA-5P(c)	NF-kappa-B (p50/p65)/I-kappa-B-alpha complex (5 phosphorylated)	Cytosol

469	2	NFKB_IKBA-5P2A(c)	NF-kappa-B (p50/p65)/I-kappa-B-alpha complex (5 phosphorylated	Cytosol
470	2	NFKB_IKBA-5P5A(c)	NF-kappa-B (p50/p65)/I-kappa-B-alpha complex (5 phosphorylated	Cytosol
471	2	NFKB_IKBA_BTRCP2(c)	NF-kB (p50/p65)/IkBa/beta TrCP2 complex	Cytosol
472	2	NFKB_IKBA_UBCH5(c)	NFkB/IkBa/UbcH5 complex	Cytosol
473	2	NFKB_IKBB(c)	NF-kappa-B (p50/p65)/I-kappa-B-beta complex	Cytosol
474	2	NFKB_IKBB-2P(c)	NF-kappa-B (p50/p65)/I-kappa-B-beta complex (2 phosphorylated)	Cytosol
475	2	NFKB_IKBB-2PU(c)	NF-kappa-B (p50/p65)/I-kappa-B-beta complex (2 phosphorylated	Cytosol
476	2	NFKB_IKBB-7P(c)	NF-kappa-B (p50/p65)/I-kappa-B-beta complex (7 phosphorylated)	Cytosol
477	2	NFKB_IKBB_BTRCP2(c)	NFkB/IkBb/betaTrCP2 complex	Cytosol
478	2	NFKB_PKAC_IKBA(c)	NF-kappa-B (p50/p65)/protein kinase A (catalytic subunit)/IkBa complex	Cytosol
479	2	NFKB_PKAC_IKBB(c)	NF-kappa-B(p50/p65)/PKAc/IkBb complex	Cytosol
480	2	NSF_STXN13(c)	NSF/Syntaxin 13 complex	Cytosol
481	2	P22PHOX(c)	p22-phox protein	Cytosol
482	2	P47PHOX-3P(c)	p47-phox protein (3 phosphorylated)	Cytosol
483	2	PCASP9_NOD1P(c)	procaspase 9/nucleotide-binding oligomerization domain protein 1 (activated) complex	Cytosol
484	2	PCASP9_NOD2P(c)	procaspase 9/nucleotide-binding oligomerization domain protein 2 (activated) complex	Cytosol
485	2	PDK1_pail345p_hs(c)	phosphoinositide-dependent kinase 1 (activated)	Cytosol
486	2	PI34P5K(c)	phosphatidylinositol 3	Cytosol
487	2	PI34P5K-P(c)	phosphatidylinositol 3	Cytosol
488	2	PI3K(p150)(c)	phosphoinositide 3-kinase	Cytosol
489	2	PI3K(vps34)(c)	phosphoinositide 3-kinase	Cytosol
490	2	PI3K3(c)	phosphoinositide 3-kinase	Cytosol
491	2	PI3K3_RAB5(c)	phosphoinositide 3-kinase/Rab5/GTP complex	Cytosol
492	2	PI3K3_RAB5_pail_hs(c)	PI3K3/Rab5/GTP/phosphatidylinositol complex	Cytosol
493	2	PI3P4K(c)	phosphatidylinositol 3-phosphate 4-kinase	Cytosol
494	2	PI3P4K-P(c)	phosphatidylinositol 3-phosphate 4-kinase (phosphorylated)	Cytosol
495	2	PI4P5K(c)	phosphatidylinositol-4-phosphate 5-kinase	Cytosol

496	2	PI4P5K-P(c)	phosphatidylinositol-4-phosphate 5-kinase (phosphorylated)	Cytosol
497	2	PKA(c)	protein kinase A	Cytosol
498	2	PKA-2P(c)	protein kinase A (2 phosphorylated)	Cytosol
499	2	PKCA-P(c)	protein kinase C	Cytosol
500	2	PKCA_ca2(c)	PKCalpha/calcium ion complex	Cytosol
501	2	PKCA_dag_ps_hs(c)	PKCalpha/diacylglycerol/phosphatidylserine complex	Cytosol
502	2	PKCB2-P(c)	protein kinase C	Cytosol
503	2	PKCB2_ca2(c)	PKCbeta2/calcium ion complex	Cytosol
504	2	PKCB2_dag_ps_hs(c)	PKCbeta2/diacylglycerol/phosphatidylserine complex	Cytosol
505	2	PKCD(c)	protein kinase C (delta isoform)	Cytosol
506	2	PKCD-2P(c)	protein kinase C (delta isoform) (2 phosphorylated)	Cytosol
507	2	PKCD-P(c)	protein kinase C (delta isoform) (phosphorylated)	Cytosol
508	2	PKCD_dag_ps_hs-2P(c)	protein kinase C/diacylglycerol/phosphatidylserine complex (delta isoform) (2 phosphorylated	Cytosol
509	2	PKCZ_pa_hs(c)	PKCzeta/phosphatidic acid complex	Cytosol
510	2	PKCZ_ps_hs(c)	PKCzeta/phosphatidylserine complex	Cytosol
511	2	PKR_TRAF6-2PD(c)	PKR (dimer)/TRAF6 (dimer) complex (4 phosphorylated)	Cytosol
512	2	PLCB2(c)	phospholipase C	Cytosol
513	2	PLCB2P(c)	phospholipase C	Cytosol
514	2	PLCG(c)	phospholipase C	Cytosol
515	2	PLCG-P(c)	phospholipase C	Cytosol
516	2	PLD(c)	phosphalipase D1	Cytosol
517	2	PLDP(c)	phospholipase D1	Cytosol
518	2	PLNO1_IRAK4(c)	pellino1/TIR/MyD88(dimer)/IRAK4 complex	Cytosol
519	2	PLNO1_IRAK4_TIRAP(c)	pellino1/TIR/TIRAP/MyD88 (dimer)/IRAK4 complex	Cytosol
520	2	PLNO3(c)	Pellino 3	Cytosol
521	2	PP2C_B1(c)	protein phosphatase 2C	Cytosol
522	2	PP2C_B1-P(c)	protein phosphatase 2C	Cytosol
523	2	PP2C_E(c)	protein phosphatase 2C	Cytosol
524	2	PP2C_E-P(c)	protein phosphatase 2C	Cytosol
525	2	PPAP(c)	phosphatidic acid phosphatase (generic)	Cytosol
526	2	PPAP-P(c)	phosphatidic acid phosphatase (generic) (phosphorylated)	Cytosol
527	2	PRE_IL1A_CALPN_3ca2(c)	pre IL-1a/calpain/calcium ion complex	Cytosol
528	2	PRE_IL1B(c)	pre interleukin 1 beta	Cytosol

529	2	PRE_IL1B_CASP1(c)	pre IL1beta/caspase 1 complex	Cytosol
530	2	RAB5_RBPTIN5_RBX5(c)	Rab5/Rabaptin5/Rabex5 complex	Cytosol
531	2	RAC1_VAV1(c)	Rac1/Vav1 complex	Cytosol
532	2	RAS_GTP_RAF1(c)	Ras/GTP/Raf-1 complex	Cytosol
533	2	RAS_GTP_RIN1(c)	Ras family small GTP-binding protein (generic)/gtp/Ras and Rab interactor 1 complex	Cytosol
534	2	RBPTIN5(c)	Rab family small GTP-binding effector protein 1	Cytosol
535	2	RBPTIN5_RBX5(c)	Rab family small GTP-binding effector protein 1/Rab5 guanine nucleotide exchange factor 1 complex	Cytosol
536	2	RBX5(c)	Rab5 guanine nucleotide exchange factor 1	Cytosol
537	2	RHOA_GDP(c)	Rho family small GTP-binding protein RhoA/gdp complex	Cytosol
538	2	RHOA_GTP(c)	Rho family small GTP-binding protein RhoA/gtp complex	Cytosol
539	2	RIN1(c)	Ras and Rab interactor 1	Cytosol
540	2	RIP1_TICAM1P(c)	RIP1/TRAF6 (dimer)/TICAM1P complex	Cytosol
541	2	RIP2_NOD1P(c)	RIP2/NOD1P complex	Cytosol
542	2	RIP2_NOD2P(c)	RIP2/NOD2P complex	Cytosol
543	2	RIP2_TRIP6_NOD1P(c)	RIP2/TRIP6/NOD1P complex	Cytosol
544	2	RIP2_TRIP6_TRAF2(c)	RIP2/TRIP6/TRAF2 complex	Cytosol
545	2	RKIP_MAP3K14(c)	RKIP/MAP3K14 (NIK) complex	Cytosol
546	2	RKIP_MAP3K7IP_TRAF6(c)	RKIP/MAP3K7/IP/Ubc13/Uev1A/TRAF6 (dimer) complex	Cytosol
547	2	SCD14(c)	soluble CD14	Cytosol
548	2	SGK(c)	serum/glucocorticoid regulated kinase	Cytosol
549	2	SGK-P(c)	serum/glucocorticoid regulated kinase (phosphorylated)	Cytosol
550	2	SH3BP5(c)	SH3-domain binding protein 5 (BTK-associated)	Cytosol
551	2	SIGIRR-D(c)	single immunoglobulin IL-1R-related molecule (dimer)	Cytosol
552	2	SOCS1(c)	suppressor of cytokine signaling 1	Cytosol
553	2	SQSTM1(c)	sequestosome 1	Cytosol
554	2	SQSTM1_PKCZ(c)	sequestosome 1/protein kinase C (zeta isoform) complex	Cytosol
555	2	SRTK(c)	Src-related tyrosine kinase	Cytosol
556	2	SRTK-P(c)	Src-related tyrosine kinase (phosphorylated)	Cytosol
557	2	ST2L(c)	ST2L	Cytosol
558	2	ST2L_TIRAP(c)	ST2L/TIRAP/MyD88(dimer) complex	Cytosol
559	2	STLR2/L_SCD14(c)	soluble TLR2/ligand/soluble CD14 complex	Cytosol
560	2	STLR2_CD14(c)	soluble TLR2/CD14 complex	Cytosol

561	2	STLR4(c)	soluble TLR4	Cytosol
562	2	STLR4/L_MD2(c)	soluble TLR4/ligand/MD-2 complex	Cytosol
563	2	STLR4_MD2(c)	MD-2/sTLR4 complex	Cytosol
564	2	TICAM1P_IKKE(c)	TICAM1P/IKKe complex	Cytosol
565	2	TICAM1P_TBK1(c)	TICAM1P/TBK1 complex	Cytosol
566	2	TICAM2(c)	toll-like receptor adaptor molecule 2	Cytosol
567	2	TICAM2_TLR4/L_MD2(c)	TICAM2/TLR4/L/MD2 complex	Cytosol
568	2	TIR_MYD_FADD(c)	TIR/MyD88 (dimer)/FADD complex	Cytosol
569	2	TIR_MYD_TOLLIP(c)	TIR/TIRAP/MyD88(dimer)/IRAK1C/IRAK4/TOLLIP complex	Cytosol
570	2	TIR_TIRAP_IRAK2(c)	TIR/TIRAP/MyD88(dimer)/IRAK4/IRAK2 complex	Cytosol
571	2	TIR_TIRAP_MYD_FADD(c)	TIR/TIRAP/MyD88 (dimer)/FADD complex	Cytosol
572	2	TIR_TIRAP_TOLLIP(c)	TIR/TIRAP/MyD88(dimer)/IRAK1/IRAK4/TOLLIP complex	Cytosol
573	2	TIR_TIRAP_TOLLIP-2P(c)	TIR/TIRAP/MyD88(dimer)/IRAK1/IRAK4/TOLLIP complex (diphosphorylated)	Cytosol
574	2	TIR_TIRAP_TOLLIP-P(c)	TIR/TIRAP/MyD88(dimer)/IRAK1/IRAK4/TOLLIP complex (phosphorylated)	Cytosol
575	2	TLR1/10/L(c)	Toll-like receptor 1/10 ligand complex	Cytosol
576	2	TLR1/2/L(c)	Toll-like receptor 1/2 ligand complex	Cytosol
577	2	TLR10/L-D(c)	Toll-like receptor 10 (dimer) ligand complex	Cytosol
578	2	TLR11(c)	Toll-like receptor 11	Cytosol
579	2	TLR11/L-D(c)	Toll-like receptor 11 (dimer) ligand complex	Cytosol
580	2	TLR2/10/L(c)	Toll-like receptor 2/10 ligand complex	Cytosol
581	2	TLR2/6/L(c)	Toll-like receptor 2/6 ligand complex	Cytosol
582	2	TLR2/L-2PD(c)	Toll-like receptor 2 (dimer) ligand/CD14 complex (4 phosphorylated)	Cytosol
583	2	TLR4/L_MD2_TRD3A(c)	Toll-like receptor 4 (dimer) ligand/MD-2/CD14/LBP/TRIAD3A complex	Cytosol
584	2	TLR4_MD2(c)	Toll-like receptor 4/MD-2 complex	Cytosol
585	2	TLR4_SIGIRR(c)	Toll-like receptor 4/SIGIRR complex	Cytosol
586	2	TLR4_SIGIRR_TRAF6(c)	TLR4/SIGIRR/TRAF6 (dimer) complex	Cytosol
587	2	TLR5/L-D(c)	Toll-like receptor 5 (dimer) ligand complex	Cytosol
588	2	TLR5_SIGIRR(c)	TLR5/SIGIRR complex	Cytosol
589	2	TLR5_SIGIRR_TRAF6(c)	TLR5/SIGIRR/TRAF6 (dimer) complex	Cytosol

590	2	TLR6(c)	Toll-like receptor 6	Cytosol
591	2	TLR9/L_TRD3A(c)	TLR9/L/TRIAD3A complex	Cytosol
592	2	TLR9_SIGIRR(c)	TLR9/SIGIRR complex	Cytosol
593	2	TLR9_SIGIRR_TRAF6(c)	TLR9/SIGIRR/TRAF6 (dimer) complex	Cytosol
594	2	TLRL1/10(c)	Toll-like receptor 1/10 ligand (generic)	Cytosol
595	2	TLRL1/2(c)	Toll-like receptor 1/2 ligand (generic)	Cytosol
596	2	TLRL10(c)	Toll-like receptor 10 ligand (generic)	Cytosol
597	2	TLRL11(c)	Toll-like receptor 11 ligand (generic)	Cytosol
598	2	TLRL2/10(c)	Toll-like receptor 2/10 ligand (generic)	Cytosol
599	2	TLRL2/6(c)	Toll-like receptor 2/6 ligand (generic)	Cytosol
600	2	TLRL5(c)	Toll-like receptor 5 ligand (generic)	Cytosol
601	2	TOLLIP-PU(c)	Toll interacting protein (phosphorylated	Cytosol
602	2	TOLLIP_pail345p_hs(c)	Toll interacting protein/phosphatidylinositol 3	Cytosol
603	2	TOLLIP_pail3p_hs(c)	Toll-interacting protein/phosphatidylinositol 3-phosphate	Cytosol
604	2	TRAF2(c)	tumor necrosis factor (TNF) receptor-associated factor 2	Cytosol
605	2	TRAF6_SIGIRR(c)	TRAF6 (dimer)/SIGIRR (dimer) complex	Cytosol
606	2	TRXRD(c)	Thioredoxin reductase	Cytosol
607	2	TRX_NADPH(c)	oxidized thioredoxin/thioredoxin reductase/NADPH complex	Cytosol
608	2	UBCH5(c)	ubiquitin-conjugating enzyme E2D (Ubc 4/5 homolog) (generic)	Cytosol
609	2	UBCH5A(c)	ubiquitin-conjugating enzyme E2D 1 (Ubc4/5 homolog)	Cytosol
610	2	accoa(c)	Acetyl-CoA	Cytosol
611	2	chol(c)	Choline	Cytosol
612	2	coa(c)	Coenzyme A	Cytosol
613	2	nadp(c)	Nicotinamide adenine dinucleotide phosphate	Cytosol
614	2	nadph(c)	Nicotinamide adenine dinucleotide phosphate - reduced	Cytosol
615	2	pail34p_hs(c)	phosphatidylinositol-3	Cytosol
616	2	pail45p_hs_PLCB2(c)	PI(4	Cytosol
617	2	pail45p_hs_PLCG(c)	PI(4	Cytosol
618	2	pail4p_hs(c)	1-Phosphatidyl-1D-myo-inositol 4-phosphate (Homo sapiens)	Cytosol
619	2	pail_hs(c)	phosphatidylinositol (homo sapiens)	Cytosol

620	2	pchol_hs(c)	Phosphatidylcholine (homo sapiens)	Cytosol
621	2	pchol_hs_PLDP(c)	phosphatidylcholine-phospholipase D (activated) complex	Cytosol
622	2	ALPS(e)	Atypical lipopolysaccharide	Extra-organism
623	2	BDFN2(e)	beta defensin 2	Extra-organism
624	2	BPM(e)	Bropirimine	Extra-organism
625	2	CPGCIGC(e)	CpG chromatin IgG2a complexes	Extra-organism
626	2	CSGA(e)	CsgA	Extra-organism
627	2	DCLDLPP(e)	Diacetylated lipopeptides	Extra-organism
628	2	DCLLPP(e)	Diacyl lipopeptides	Extra-organism
629	2	ENVP(e)	Envelope protein	Extra-organism
630	2	FBNG(e)	Fibrinogen	Extra-organism
631	2	FLGN(e)	Flagellin	Extra-organism
632	2	FUSP(e)	Fusion protein	Extra-organism
633	2	GCSPL(e)	Glycoinositol phospholipids	Extra-organism
634	2	GLC(e)	Glycolipids	Extra-organism
635	2	HSP60(e)	Heat shock protein 60	Extra-organism
636	2	IL1A(e)	interleukin 1 alpha	Extra-organism
637	2	IL1B(e)	interleukin 1 beta	Extra-organism
638	2	LAM(e)	Lipoarabinomannan	Extra-organism
639	2	LP(e)	Lipoprotein	Extra-organism
640	2	LPPS(e)	Lipopeptides	Extra-organism
641	2	LXR(e)	Loxoribine	Extra-organism
642	2	MRDP(e)	muramyl dipeptide	Extra-organism
643	2	MRNA(e)	mRNA (generic)	Extra-organism
644	2	OLSCHYA(e)	Oligosaccharides of hyaluronic acid	Extra-organism
645	2	OMPA(e)	Outer membrane protein A	Extra-organism
646	2	OSPALP(e)	Outer surface protein A lipoprotein	Extra-organism
647	2	PLP(e)	Profilin-like protein	Extra-organism
648	2	PRE_IL1A(e)	pre interleukin 1 alpha	Extra-organism
649	2	PRNS(e)	Porins	Extra-organism
650	2	PSCHPS(e)	Polysaccharide fragment of heparan sulphate	Extra-organism
651	2	PTG_HS(e)	Peptidoglycan (Homo sapiens)	Extra-organism
652	2	SF(e)	Soluble factors	Extra-organism
653	2	STF(e)	Small molecule transcription factors	Extra-organism
654	2	T3RFBN(e)	Type III repeat extra domain A of fibronectin	Extra-organism
655	2	TCLDLPP(e)	Triacetylated lipopeptides	Extra-organism
656	2	TLRL1/10(e)	Toll-like receptor 1/10 ligand (generic)	Extra-organism
657	2	TLRL10(e)	Toll-like receptor 10 ligand (generic)	Extra-organism
658	2	TLRL2/10(e)	Toll-like receptor 2/10 ligand (generic)	Extra-organism
659	2	TLRL5(e)	Toll-like receptor 5 ligand (generic)	Extra-organism

660	2	TXL(e)	Taxol	Extra-organism
661	2	UBIQ(e)	Ubiquitin	Extra-organism
662	2	UMLCPGD(e)	Unmethylated CpG DNA	Extra-organism
663	2	UNKN(e)	Unknown TLR11 ligand	Extra-organism
664	2	accoa(e)	Acetyl-CoA	Extra-organism
665	2	adp(e)	ADP	Extra-organism
666	2	atp(e)	ATP	Extra-organism
667	2	ca2(e)	Calcium	Extra-organism
668	2	chol(e)	Choline	Extra-organism
669	2	coa(e)	Coenzyme A	Extra-organism
670	2	dag_hs(e)	diacylglycerol (homo sapiens)	Extra-organism
671	2	gdp(e)	GDP	Extra-organism
672	2	gtp(e)	GTP	Extra-organism
673	2	h2o(e)	H2O	Extra-organism
674	2	h2o2(e)	Hydrogen peroxide	Extra-organism
675	2	mi145p(e)	1D-myo-Inositol 1	Extra-organism
676	2	nadp(e)	Nicotinamide adenine dinucleotide phosphate	Extra-organism
677	2	nadph(e)	Nicotinamide adenine dinucleotide phosphate - reduced	Extra-organism
678	2	pa_hs(e)	phosphatidic acid (homo sapiens)	Extra-organism
679	2	pail345p_hs(e)	phosphatidylinositol-3	Extra-organism
680	2	pail4p_hs(e)	1-Phosphatidyl-1D-myo-inositol 4-phosphate (Homo sapiens)	Extra-organism
681	2	pail_hs(e)	phosphatidylinositol (homo sapiens)	Extra-organism
682	2	pchol_hs(e)	Phosphatidylcholine (homo sapiens)	Extra-organism
683	2	pi(e)	Phosphate	Extra-organism
684	2	ps_hs(e)	phosphatidylserine (homo sapiens)	Extra-organism
685	2	TLR3/L-2PD(l)	Toll-like receptor 3 (dimer) ligand complex (4 phosphorylated)	Lysosome
686	2	adp(l)	ADP	Lysosome
687	2	atp(l)	ATP	Lysosome
688	2	h(l)	H+	Lysosome
689	2	A1909_DEGR(n)	A1909 (degraded)	Nucleus
690	2	A1910_DEGR(n)	A1910 (degraded)	Nucleus
691	2	A2170_DEGR(n)	A2170 (degraded)	Nucleus
692	2	AKT-2P(n)	Akt (2 phosphorylated)	Nucleus
693	2	AP1_FOS_JUN(n)	AP-1/c-Fos/c-Jun complex (8 phosphorylated)	Nucleus
694	2	AP1_JUN(n)	AP-1/c-Jun (dimer) complex (4 phosphorylated)	Nucleus
695	2	BCL3(n)	B-cell CLL/lymphoma 3	Nucleus
696	2	BTRCP1(n)	beta-transducin repeat containing protein 1	Nucleus
697	2	CREB(n)	cAMP responsive element binding protein	Nucleus
698	2	CREB-2PD(n)	cAMP responsive element binding protein (2 phosphorylated	Nucleus
699	2	CREB-D(n)	cAMP responsive element binding protein (dimer)	Nucleus

700	2	ELK1_SRE(n)	ELK1 (2 phosphorylated)/SRE gene complex	Nucleus
701	2	FOS_JUN(n)	c-Fos/c-Jun complex (8 phosphorylated)	Nucleus
702	2	GSK3B(n)	glycogen synthase kinase 3 beta	Nucleus
703	2	GSK3B-P(n)	glycogen synthase kinase 3 beta (phosphorylated)	Nucleus
704	2	HDAC1(n)	histone deacetylase 1 (inactive)	Nucleus
705	2	HH3(n)	histone H3	Nucleus
706	2	HH3-P(n)	histone H3 (phosphorylated)	Nucleus
707	2	HMG14(n)	high-mobility group nucleosome binding domain 1	Nucleus
708	2	HMG14-P(n)	high-mobility group nucleosome binding domain 1 (phosphorylated)	Nucleus
709	2	HNRNPA1(n)	heterogeneous nuclear ribonucleoprotein A1	Nucleus
710	2	HNRNPA1-3P(n)	heterogeneous nuclear ribonucleoprotein A1 (3 phosphorylated)	Nucleus
711	2	IKBB-2P(n)	I-kappa-B-beta (2 phosphorylated)	Nucleus
712	2	IKBZ(n)	I-kappa-B-zeta (protein)	Nucleus
713	2	IMPA3(n)	importin alpha 3	Nucleus
714	2	IRF3-2PD(n)	interferon regulatory factor 3 (2 phosphorylated	Nucleus
715	2	IRF7-2P(n)	interferon regulatory factor 7 (2 phosphorylated)	Nucleus
716	2	ISRE_IRF3(n)	ISRE/IRF3 complex (2 phosphorylated)	Nucleus
717	2	ISRE_IRF7(n)	ISRE/IRF7 complex (2 phosphorylated)	Nucleus
718	2	JUN-2PD(n)	c-Jun (2 phosphorylated	Nucleus
719	2	MAPK11_MAPKAPK2(n)	MAPK11/MAPKAPK2 complex	Nucleus
720	2	MAPK14_MAPKAPK2(n)	MAPK14/MAPKAPK2 complex	Nucleus
721	2	MKP(n)	MAP kinase phosphatase	Nucleus
722	2	MKP-P(n)	MAP kinase phosphatase (phosphorylated)	Nucleus
723	2	MYC(n)	c-Myc	Nucleus
724	2	MYC-2P(n)	c-Myc (2 phosphorylated)	Nucleus
725	2	NFKB(p50)_IKBZ(n)	NF-kappa-B (p50 dimer)/I-kappa-B-zeta complex (2 phosphorylated)	Nucleus
726	2	NFKB(p50)_IMPA3(n)	NF-kB(p50)-importin alpha 3 complex	Nucleus
727	2	NFKB(p50)_KB_SITE(n)	NF-kappa-B (p50 dimer)/kB site/HDAC1 complex	Nucleus
728	2	NFKB(p50/p65)-5P2A(n)	NF-kappa-B (p50/p65) complex (5 phosphorylated	Nucleus
729	2	NFKB(p50/p65)_CBP-5P3A(n)	NF-kappa-B (p50/p65)/CBP complex (5 phosphorylated	Nucleus
730	2	NFKB(p65)-5P3A(n)	NF-kappa-B (p65) (5 phosphorylated	Nucleus

731	2	NFKB(p65)-5P3AU(n)	NF-kappa-B (p65) (5phosphorylated	Nucleus
732	2	NFKB_IKBA-5P(n)	NF-kappa-B (p50/p65)/I-kappa-B-alpha complex (5 phosphorylated)	Nucleus
733	2	NFKB_IKBA-5P2A(n)	NF-kappa-B (p50/p65)/I-kappa-B-alpha complex (5 phosphorylated	Nucleus
734	2	NFKB_IKBA-5P5A(n)	NF-kappa-B (p50/p65)/I-kappa-B-alpha complex (5 phosphorylated	Nucleus
735	2	NFKB_IKBA_BTRCP1(n)	NFkB/IkBa/betaTrCP1 complex	Nucleus
736	2	NFKB_IKBA_UBCH5(n)	NFkB/IkBa/UbcH5 complex	Nucleus
737	2	NFKB_IKBB-7P(n)	NF-kappa-B (p50/p65)/I-kappa-B-beta complex (7 phosphorylated)	Nucleus
738	2	PKCZ(n)	protein kinase C (zeta isoform)	Nucleus
739	2	PKCZ-P(n)	protein kinase C (zeta isoform) (phosphorylated)	Nucleus
740	2	PP2B(n)	protein phosphatase 2B	Nucleus
741	2	PP2B-P(n)	protein phosphatase 2B (phosphorylated)	Nucleus
742	2	SRE_GENE(n)	c-Fos serum response element (gene)	Nucleus
743	2	UBCH5(n)	ubiquitin-conjugating enzyme E2D (Ubc 4/5 homolog) (generic)	Nucleus
744	2	ac(n)	Acetate	Nucleus
745	2	IP3R(r)	inositol 1	Endoplasmic Reticulum
746	2	IP3R_mi145p(r)	inositol 1	Endoplasmic Reticulum
747	2	SERCA(r)	sarco/endoplasmic reticulum Ca(2+)-ATPase	Endoplasmic Reticulum
748	2	SERCA-P(r)	sarco/endoplasmic reticulum Ca(2+)-ATPase (phosphorylated)	Endoplasmic Reticulum
749	2	EEA1_pail3p_hs(v)	EEA1(dimer)/PI3P complex	Vacuole
750	2	PHOX_GTP-3P(v)	gp91/p22/p40/p47 (3 phosphorylated)/p67PHOX/Rac1 /GTP complex	Vacuole
751	2	PHOX_GTP-8P(v)	gp91/p22/p40/p47 (8 phosphorylated)/p67PHOX/Rac1 /GTP complex	Vacuole
752	2	TLRL9(v)	Toll-like receptor 9 ligand (generic)	Vacuole
