# Supplementary material for: Identification of Potential Pathway Mediation Targets in Toll-like Receptor Signaling
Source: PLoS Comput Biol. 2009 Feb 20;5(2):e1000292. doi: 10.1371/journal.pcbi.1000292 (PMC2634968; doi:10.1371/journal.pcbi.1000292)
Supplement: Table S2 — TLR network reactions (0.30 MB PDF) [file pcbi.1000292.s004.pdf]

Table S2: TLR network reactions

ABBREVIATION	OFFICIAL NAME	DIRECTION
DM_A001_DEGR(c)	A001 degraded demand	Irreversible
DM_A1115_DEGR(c)	A1115 (degraded) demand	Irreversible
DM_A1146_DEGR(c)	A1146 (degraded) demand	Irreversible
DM_A1159_DEGR(c)	A1159 (degraded) demand	Irreversible
DM_A153_DEGR(c)	A153 (degraded) demand	Irreversible
DM_A1901_DEGR(c)	A1901 (degraded) demand	Irreversible
DM_A1901_DEGR2(c)	A1901 degraded 2 demand	Irreversible
DM_A1909_DEGR(n)	A1909 (degraded) demand	Irreversible
DM_A1910_DEGR(n)	A1910 (degraded) demand	Irreversible
DM_A2069_DEGR(c)	A2069 (degraded) demand	Irreversible
DM_A2129_DEGR(c)	A2129 (degraded) demand	Irreversible
DM_A2156_DEGR(c)	A2156 (degraded) demand	Irreversible
DM_A2170_DEGR(n)	A2170 (degraded) demand	Irreversible
DM_A2382_DEGR(c)	A2382 (degraded) demand	Irreversible
DM_A385_DEGR(c)	A385 (degraded) demand	Irreversible
DM_A399_DEGR(c)	A399 (degraded) demand	Irreversible
DM_A399_DEGR2(c)	A399 (degraded) (unphosphorylated) demand	Irreversible
DM_A454_DEGR(c)	A454 (degraded) demand	Irreversible
DM_A523_DEGR(c)	A523 (degraded) demand	Irreversible
DM_A796_DEGR(c)	A796 (degraded) demand	Irreversible
DM_A839_DEGR(c)	A839 (degraded) demand	Irreversible
DM_ABIN_A20(c)	ABIN/A20 complex demand	Irreversible
DM_AKT-2P(n)	Akt (2 phosphorylated) demand	Irreversible
DM_AP1_FOS_JUN(n)	AP-1/c-Fos/c-Jun complex (8 phosphorylated) demand	Irreversible
DM_AP1_JUN(n)	AP-1/c-Jun (dimer) complex (4 phosphorylated) demand	Irreversible
DM_ATF2-P(n)	Activating transcription factor 2 (phosphorylated) demand	Irreversible
DM_BTK_IBTK(c)	BTK/iBTK complex demand	Irreversible
DM_BTK_SH3BP5(c)	BTK/SH3BP5 complex demand	Irreversible
DM_CASP8(c)	caspase 8 demand	Irreversible
DM_CASP9(c)	caspase 9 demand	Irreversible
DM_CREB-2PD(n)	cAMP responsive element binding protein (2 phosphorylated, dimer) demand	Irreversible
DM_CREB_CRE-2P(n)	CREB/CRE site complex (2 phosphorylated) demand	Irreversible
DM_DSRNA(c)	Double stranded RNA demand	Irreversible
DM_EIF2A-P(c)	eukaryotic translation initiation factor 2 (phosphorylated) demand	Irreversible
DM_EIF4E-P(c)	eukaryotic translation initiation factor 4E (phosphorylated) demand	Irreversible
DM_ELK1_SRE(n)	ELK1 (2 phosphorylated)/SRE gene complex demand	Irreversible
DM_GSK3B-2P(c)	glycogen synthase kinase 3 beta (2 phosphorylated) demand	Irreversible
DM_HH3-P(n)	histone H3 (phosphorylated) demand	Irreversible
DM_HMG14-P(n)	high-mobility group nucleosome binding domain 1 (phosphorylated) demand	Irreversible
DM_HSP27-3P(c)	heat shock protein 27kDa (3 phosphorylated) demand	Irreversible
DM_IKK-3P(c)	inhibitor of kappa light polypeptide gene enhancer in B-cells kinase alpha(dimer)/beta(dimer)/gamma(tetramer) complex (6 phosphorylated) demand	Irreversible
DM_IKK_SRC(c)	inhibitor of kappa light polypeptide gene enhancer in B-cells kinase alpha/beta/gamma(dimer)/c-Src complex demand	Irreversible
DM_IL1R1/L/AP-P(c)	interleukin 1 receptor type 1/ligand/accessory protein complex (phosphorylated) demand	Irreversible
DM_IL1R1_LIG(c)	interleukin 1 receptor, type 1 ligand (generic) demand	Irreversible
DM_IL1R2/AP(c)	interleukin 1 receptor type 2/accessory protein complex demand	Irreversible
DM_IRAK1_MAP3K3-3P3U(c)	IRAK1/TIFA/TRAF6 (dimer)/Ubc13/Uev1A/MAP3K3 complex (3 phosphorylated, 3 ubiquitinated) demand	Irreversible

DM_IRAK1_PLNO3(c)	IRAK1/Pellino3 complex demand	Irreversible
DM_IRAK_TOLLIP_TIR(c)	IRAK4/IRAK1c/MyD88(dimer)/TIR/TOLLIP/TRAF6-D complex demand	Irreversible
DM_ISRE_IRF3(n)	ISRE/IRF3 complex (2 phosphorylated) demand	Irreversible
DM_ISRE_IRF7(n)	ISRE/IRF7 complex (2 phosphorylated) demand	Irreversible
DM_KSR1_RAF1-7P(c)	KSR1/MAP2K1/Raf-1 complex (7 phosphorylated) demand	Irreversible
DM_MAP3K5-2P(c)	mitogen-activated protein kinase kinase kinase 5 (2 phosphorylated) demand	Irreversible
DM_MAP3K7IP_TRAF6-5P3U(c)	MAP3K7/IP23/1/TRAF6 (dimer)/Ubc13/Uev1A complex (5 phosphorylated, 3 ubiquitinated) demand	Irreversible
DM_MAP3K8(p58)_KSR2(c)	mitogen-activated protein kinase kinase kinase 8 (p58)/KSR2 complex (phosphorylated) demand	Irreversible
DM_MBP-P(c)	myelin basic protein (phosphorylated) demand	Irreversible
DM_MD2-D(c)	MD-2 (dimer) demand	Irreversible
DM_MKNK2-P(c)	MAP kinase interacting serine/threonine kinase 2 demand	Irreversible
DM_MYC-2P(n)	c-Myc (2 phosphorylated) demand	Irreversible
DM_MYD88_MyD88S(c)	MyD88/MyD88s complex demand	Irreversible
DM_NFKB(p50)(n)	NF-kappa-B (p50) demand	Irreversible
DM_NFKB(p50)_BCL3(n)	NF-kappa-B (p50 dimer)/Bcl-3 complex (2 phosphorylated) demand	Irreversible
DM_NFKB(p50)_IKBZ(n)	NF-kappa-B (p50 dimer)/I-kappa-B-zeta complex (2 phosphorylated) demand	Irreversible
DM_NFKB(p50/p65)-5P5A(n)	NF-kappa-B (p50/p65) complex (5 phosphorylated, 5 acetylated) demand	Irreversible
DM_NFKB_FRAG(c)	NF-kB processing fragment demand	Irreversible
DM_NFKB_FRAG2(c)	NF-kB fragment 2 demand	Irreversible
DM_NFKB_IKBB-7P(c)	NF-kappa-B (p50/p65)/I-kappa-B-beta complex (7 phosphorylated) demand	Irreversible
DM_NSF_EEA1_RAB5(v)	NSF/Syntaxin 13/EEA1 (dimer)/PI3P/Rab5/GTP complex demand	Irreversible
DM_PHOX_GTP-3P(v)	gp91/p22/p40/p47 (3 phosphorylated)/p67PHOX/Rac1/GTP complex demand	Irreversible
DM_PHOX_GTP-8P(v)	gp91/p22/p40/p47 (8 phosphorylated)/p67PHOX/Rac1/GTP complex demand	Irreversible
DM_PLNO1_IRAK4(c)	pellino1/TIR/MyD88(dimer)/IRAK4 complex demand	Irreversible
DM_PLNO1_IRAK4_TIRAP(c)	pellino1/TIR/TIRAP/MyD88 (dimer)/IRAK4 complex demand	Irreversible
DM_RAS_GTP_RIN1(c)	Ras family small GTP-binding protein (generic)/gtp/Ras and Rab interactor 1 complex demand	Irreversible
DM_RIP2_TRIP6_NOD1P(c)	RIP2/TRIP6/NOD1P complex demand	Irreversible
DM_RKIP_MAP3K14(c)	RKIP/MAP3K14 (NIK) complex demand	Irreversible
DM_RKIP_MAP3K7IP_TRAF6(c)	RKIP/MAP3K7/IP/Ubc13/Uev1A/TRAF6 (dimer) complex demand	Irreversible
DM_ST2L_TIRAP(c)	ST2L/TIRAP/MyD88(dimer) complex demand	Irreversible
DM_STLR2/L_SCD14(c)	soluble TLR2/ligand/soluble CD14 complex demand	Irreversible
DM_STLR2_CD14(c)	soluble TLR2/CD14 complex demand	Irreversible
DM_STLR4/L_MD2(c)	soluble TLR4/ligand/MD-2 complex demand	Irreversible
DM_TIR_MYD_TOLLIP(c)	TIR/TIRAP/MyD88(dimer)/IRAK1C/IRAK4/TOLLIP complex demand	Irreversible
DM_TIR_TIRAP_IRAK2(c)	TIR/TIRAP/MyD88(dimer)/IRAK4/IRAK2 complex demand	Irreversible
DM_TLR4_SIGIRR_TRAF6(c)	TLR4/SIGIRR/TRAF6 (dimer) complex demand	Irreversible
DM_TLR5_SIGIRR_TRAF6(c)	TLR5/SIGIRR/TRAF6 (dimer) complex demand	Irreversible
DM_TLR9_SIGIRR_TRAF6(c)	TLR9/SIGIRR/TRAF6 (dimer) complex demand	Irreversible
DM_TLRL1/10(c)	Toll-like receptor 1/10 ligand (generic) demand	Irreversible
DM_TLRL1/2(c)	Toll-like receptor 1/2 ligand (generic) demand	Irreversible
DM_TLRL10(c)	Toll-like receptor 10 ligand (generic) demand	Irreversible
DM_TLRL11(c)	Toll-like receptor 11 ligand (generic) demand	Irreversible
DM_TLRL2(c)	Toll-like receptor 2 ligand (generic) demand	Irreversible
DM_TLRL2/10(c)	Toll-like receptor 2/10 ligand (generic) demand	Irreversible
DM_TLRL2/6(c)	Toll-like receptor 2/6 ligand (generic) demand	Irreversible
DM_TLRL3(c)	Toll-like receptor 3 ligand (generic) demand	Irreversible
DM_TLRL4(c)	Toll-like receptor 4 ligand (generic) demand	Irreversible
DM_TLRL5(c)	Toll-like receptor 5 ligand (generic) demand	Irreversible
DM_TLRL7(c)	Toll-like receptor 7 ligand (generic) demand	Irreversible

DM_TLRL8(c)	Toll-like receptor 8 ligand (generic) demand	Irreversible
DM_TLRL9(c)	Toll-like receptor 9 ligand (generic) demand	Irreversible
DM_TOLLIP_pail345p_hs(c)	Toll interacting protein/phosphatidylinositol 3,4,5-trisphosphate complex demand	Irreversible
DM_TOLLIP_pail3p_hs(c)	Toll-interacting protein/phosphatidylinositol 3-phosphate demand	Irreversible
DM_TRAF6-UD(c)	tumor necrosis factor (TNF) receptor-associated factor 6 (ubiquitinated, dimer) demand	Irreversible
DM_TRAF6_SIGIRR(c)	TRAF6 (dimer)/SIGIRR (dimer) complex demand	Irreversible
EX_26dap-LL(e)	LL-2,6-Diaminoheptanedioate exchange	Reversible
EX_ALPS(e)	Atypical lipopolysaccharide exchange	Reversible
EX_BDFN2(e)	beta defensin 2 exchange	Reversible
EX_BPM(e)	Bropirimine exchange	Reversible
EX_CPGCIGC(e)	CpG chromatin IgG2a complexes exchange	Reversible
EX_CSGA(e)	CsgA exchange	Reversible
EX_DCLDLPP(e)	Diacetylated lipopeptides exchange	Reversible
EX_DCLLPP(e)	Diacyl lipopeptides exchange	Reversible
EX_DSRNA(e)	Double stranded RNA exchange	Reversible
EX_ENVP(e)	Envelope protein exchange	Reversible
EX_FBNG(e)	Fibrinogen exchange	Reversible
EX_FLGN(e)	Flagellin exchange	Reversible
EX_FUSP(e)	Fusion protein exchange	Reversible
EX_GCSPL(e)	Glycoinositol phospholipids exchange	Reversible
EX_GLC(e)	Glycolipids exchange	Reversible
EX_HSP60(e)	Heat shock protein 60 exchange	Reversible
EX_HSP70(e)	Heat shock protein 70 exchange	Reversible
EX_IMQ(e)	Imidazoquinoline exchange	Reversible
EX_LAM(e)	Lipoarabinomannan exchange	Reversible
EX_LP(e)	Lipoprotein exchange	Reversible
EX_LPPS(e)	Lipopeptides exchange	Reversible
EX_LPS_HS(e)	Lipopolysaccharide (Homo sapiens) exchange	Reversible
EX_LTA(e)	Lipoteichoic acid exchange	Reversible
EX_LXR(e)	Loxoribine exchange	Reversible
EX_MRAP(e)	Mannuronic acid polymer exchange	Reversible
EX_MRDP(e)	muramyl dipeptide exchange	Reversible
EX_MRNA(e)	mRNA (generic) exchange	Reversible
EX_OLSCHYA(e)	Oligosaccharides of hyaluronic acid exchange	Reversible
EX_OMPA(e)	Outer membrane protein A exchange	Reversible
EX_OSPALP(e)	Outer surface protein A lipoprotein exchange	Reversible
EX_PLP(e)	Profilin-like protein exchange	Reversible
EX_PRNS(e)	Porins exchange	Reversible
EX_PSCHPS(e)	Polysaccharide fragment of heparan sulphate exchange	Reversible
EX_PSM(e)	Phenol-soluble modulin exchange	Reversible
EX_PTG_HS(e)	Peptidoglycan (Homo sapiens) exchange	Reversible
EX_SF(e)	Soluble factors exchange	Reversible
EX_SSRNA(e)	Single stranded RNA exchange	Reversible
EX_STF(e)	Small molecule transcription factors exchange	Reversible
EX_T3RFBN(e)	Type III repeat extra domain A of fibronectin exchange	Reversible
EX_TCLDLPP(e)	Triacetylated lipopeptides exchange	Reversible
EX_TLRL1/10(e)	Toll-like receptor 1/10 ligand (generic) exchange	Reversible
EX_TLRL10(e)	Toll-like receptor 10 ligand (generic) exchange	Reversible
EX_TLRL2/10(e)	Toll-like receptor 2/10 ligand (generic) exchange	Reversible
EX_TXL(e)	Taxol exchange	Reversible
EX_UBIQ(e)	Ubiquitin exchange	Reversible
EX_UMLCPGD(e)	Unmethylated CpG DNA exchange	Reversible
EX_UNKN(e)	Unknown TLR11 ligand exchange	Reversible
EX_ZMS(e)	Zymosan exchange	Reversible
EX_ac(e)	Acetate exchange	Reversible
EX_accoa(e)	Acetyl-CoA exchange	Reversible

EX_adp	adp exchange	Reversible
EX_atp(e)	ATP exchange	Reversible
EX_ca2(e)	Calcium exchange	Reversible
EX_chol(e)	Choline exchange	Reversible
EX_coa(e)	Coenzyme A exchange	Reversible
EX_dag_hs(e)	diacylglycerol (homo sapiens) exchange	Reversible
EX_gdp(e)	GDP exchange	Reversible
EX_gtp(e)	GTP exchange	Reversible
EX_h(e)	H+ exchange	Reversible
EX_h2o(e)	H2O exchange	Reversible
EX_h2o2(e)	Hydrogen peroxide exchange	Reversible
EX_mi145p(e)	1D-myo-Inositol 1,4,5-trisphosphate exchange	Reversible
EX_nadp(e)	Nicotinamide adenine dinucleotide phosphate exchange	Reversible
EX_nadph(e)	Nicotinamide adenine dinucleotide phosphate - reduced exchange	Reversible
EX_pa_hs(e)	phosphatidic acid (homo sapiens) exchange	Reversible
EX_pail345p_hs(e)	phosphatidylinositol-3,4,5-trisphosphate (Homo sapiens) exchange	Reversible
EX_pail4p_hs(e)	1-Phosphatidyl-1D-myo-inositol 4-phosphate (Homo sapiens) exchange	Reversible
EX_pail_hs(e)	phosphatidylinositol (homo sapiens) exchange	Reversible
EX_pchol_hs(e)	Phosphatidylcholine (homo sapiens) exchange	Reversible
EX_pi(e)	Phosphate exchange	Reversible
EX_ps_hs(e)	phosphatidylserine (homo sapiens) exchange	Reversible
sink_A20	sink_A20	Reversible
sink_ABIN	sink_ABIN	Reversible
sink_AKT	sink_AKT	Reversible
sink_AP1_GENE	sink_AP1_GENE	Reversible
sink_ATF2	sink_ATF2	Reversible
sink_BCL3	sink_BCL3	Reversible
sink_BTK	sink_BTK	Reversible
sink_CD14	sink_CD14	Reversible
sink_CREB	sink_CREB	Reversible
sink_CRE_GENE	sink_CRE_GENE	Reversible
sink_EEA1	sink_EEA1	Reversible
sink_EIF2A	sink_EIF2A	Reversible
sink_EIF4E	sink_EIF4E	Reversible
sink_ELK1	sink_ELK1	Reversible
sink_FMAP3K1	sink_FMAP3K1	Reversible
sink_FOS	sink_FOS	Reversible
sink_GP91PHOX	sink_GP91PHOX	Reversible
sink_GSK3B	sink_GSK3B	Reversible
sink_HH3	sink_HH3	Reversible
sink_HMG14	sink_HMG14	Reversible
sink_HSP27	sink_HSP27	Reversible
sink_IBTK	sink_IBTK	Reversible
sink_IKBA	sink_IKBA	Reversible
sink_IKBB	sink_IKBB	Reversible
sink_IKBZ	sink_IKBZ	Reversible
sink_IKK	sink_IKK	Reversible
sink_IL1R2	sink_IL1R2	Reversible
sink_IL1RAP	sink_IL1RAP	Reversible
sink_IRAK1	sink_IRAK1	Reversible
sink_IRAK1C	sink_IRAK1C	Reversible
sink_IRAK2	sink_IRAK2	Reversible
sink_IRAK4	sink_IRAK4	Reversible
sink_IRF3	sink_IRF3	Reversible
sink_IRF7	sink_IRF7	Reversible
sink_ISRE	sink_ISRE	Reversible
sink_JUN	sink_JUN	Reversible

sink_KSR1	sink_KSR1	Reversible
sink_KSR2	sink_KSR2	Reversible
sink_LBP	sink_LBP	Reversible
sink_MAP2K1	sink_MAP2K1	Reversible
sink_MAP3K14	sink_MAP3K14	Reversible
sink_MAP3K3	sink_MAP3K3	Reversible
sink_MAP3K5	sink_MAP3K5	Reversible
sink_MAP3K7	sink_MAP3K7	Reversible
sink_MAP3K7IP1	sink_MAP3K7IP1	Reversible
sink_MAP3K7IP2/3	sink_MAP3K7IP2/3	Reversible
sink_MAP3K8(p52)	sink_MAP3K8(p52)	Reversible
sink_MAP3K8(p58)	sink_MAP3K8(p58)	Reversible
sink_MBP	sink_MBP	Reversible
sink_MD2	sink_MD2	Reversible
sink_MKNK2	sink_MKNK2	Reversible
sink_MYC	sink_MYC	Reversible
sink_MYD88	sink_MYD88	Reversible
sink_MYD88S	sink_MYD88S	Reversible
sink_NFKB(p105)	sink_NFKB(p105)	Reversible
sink_NFKB(p65)	sink_NFKB(p65)	Reversible
sink_NOD1	sink_NOD1	Reversible
sink_NSF	sink_NSF	Reversible
sink_P22PHOX	sink_P22PHOX	Reversible
sink_P40PHOX	sink_P40PHOX	Reversible
sink_P47PHOX	sink_P47PHOX	Reversible
sink_P67PHOX	sink_P67PHOX	Reversible
sink_PCASP8	sink_PCASP8	Reversible
sink_PCASP9	sink_PCASP9	Reversible
sink_PLNO1	sink_PLNO1	Reversible
sink_PLNO3	sink_PLNO3	Reversible
sink_PRE_IL1A	sink_PRE_IL1A	Reversible
sink_PRE_IL1B	sink_PRE_IL1B	Reversible
sink_RAB5_GDP	sink_RAB5_GDP	Reversible
sink_RAC1_GDP	sink_RAC1_GDP	Reversible
sink_RAF1	sink_RAF1	Reversible
sink_RAS_GDP	sink_RAS_GDP	Reversible
sink_RIN1	sink_RIN1	Reversible
sink_RIP1	sink_RIP1	Reversible
sink_RIP2	sink_RIP2	Reversible
sink_RKIP	sink_RKIP	Reversible
sink_SCD14	sink_SCD14	Reversible
sink_SH3BP5	sink_SH3BP5	Reversible
sink_SIGIRR	sink_SIGIRR	Reversible
sink_SRCK	sink_SRCK	Reversible
sink_SRE_GENE	sink_SRE_GENE	Reversible
sink_ST2L	sink_ST2L	Reversible
sink_STLR2	sink_STLR2	Reversible
sink_STLR4	sink_STLR4	Reversible
sink_STXN13	sink_STXN13	Reversible
sink_TIFA	sink_TIFA	Reversible
sink_TIR	sink_TIR	Reversible
sink_TIRAP	sink_TIRAP	Reversible
sink_TLR4	sink_TLR4	Reversible
sink_TLR5	sink_TLR5	Reversible
sink_TLR9	sink_TLR9	Reversible
sink_TOLLIP	sink_TOLLIP	Reversible
sink_TRAF6	sink_TRAF6	Reversible
sink_TRIP6	sink_TRIP6	Reversible

sink_UBC13	sink_UBC13	Reversible
sink_UEV1A	sink_UEV1A	Reversible
14-3-3_KSR1_BIND	14-3-3-KSR1-MKK1 binding	Irreversible
14-3-3_RAF1_BIND	14-3-3-Raf1 binding	Irreversible
26dap-LLti	diaminopimelic acid transporter, irreversible	Irreversible
26dap-LLti2	diaminopimelic acid transporter 2, irreversible	Irreversible
ABIN_A20_BIND	ABIN-A20 binding	Irreversible
ACCOAt	AcCoA transporter	Reversible
ACCOAtn	acetyl-CoA transport, nuclear	Reversible
ACt	Acetate exchange, diffusion	Irreversible
ACt2	acetate transport in via proton symport	Irreversible
ACtn	Acetate transport, nucleus	Reversible
ADPtl	ADP transporter, lysosome	Reversible
ADPtn	ADP transporter, nucleus	Reversible
AJUBA_CPX_BIND	Ajuba-IRAK1/TRAF6 (dimer)/TIFA/Ubc13/Uev1A-sequestosome1/PKCzeta binding	Irreversible
AJUBA_CPX_IKK	PKCzeta-mediated IKK phosphorylation	Irreversible
AJUBA_CPX_PHOS	Ajuba/TRAF6 (dimer)/IRAK1/PKCz/TIFA/Sequestosome 1/Ubc13/Uev1A autophosphorylation	Irreversible
AKT_GSK3B	Akt-mediated GSK3beta phosphorylation	Irreversible
AKT_IKK	Akt-mediated IKK phosphorylation	Irreversible
AKT_MAP3K3	Akt-mediated MAP3K3 phosphorylation	Irreversible
AKT_MAP3K5	Akt-mediated MAP3K5 diphosphorylation	Irreversible
AKT_PHOS	Akt autophosphorylation	Irreversible
AKT_RAF1	Akt-mediated Raf-1 phosphorylation	Irreversible
AKT_TRAN	Akt transport	Irreversible
AKT_pail345p_hs_BIND	Akt-phosphatidylinositol 3,4,5-trisphosphate binding	Irreversible
AKT_pail345p_hs_DISS	Akt-phosphatidylinositol 3,4,5-trisphosphate dissociation	Irreversible
ALPS_TLRL2	Toll-like receptor 2 ligand (Atypical lipopolysaccharide)	Irreversible
AP1_FOS_JUN_BIND	AP-1 site-cFos-cJun binding	Irreversible
AP1_JUN_BIND	AP-1 site-cJun binding	Irreversible
ATPtl	ATP transporter, lysosome	Reversible
ATPtn	ATP diffusion in nucleus	Reversible
BCL3_TRAN	Bcl-3 transport	Reversible
BDFN2_TLRL4	Toll-like receptor 4 ligand (beta defensin 2)	Irreversible
BPM_TLRL7	Toll-like receptor 7 ligand (Bropirimine)	Irreversible
BTK_2PHOS	BTK autophosphorylation	Irreversible
BTK_CBL_BIND	BTK-cCbl binding	Irreversible
BTK_DEGR	BTK degradation	Irreversible
BTK_IBTK_BIND	BTK-iBTK binding	Irreversible
BTK_PLCG	BTK-mediated PLCgamma phosphorylation	Irreversible
BTK_SH3BP5_BIND	BTK-SH3BP5(Sab) binding	Irreversible
BTK_UBIQ	BTK ubiquitination	Irreversible
BTK_VAV1	BTK-mediated Vav1 phosphorylation	Irreversible
BTK_pail345p_hs_BIND	BTK-phosphatidylinositol 3,4,5-trisphosphate binding	Irreversible
CA2t	calcium (Ca+2) transport via diffusion (extracellular to periplasm)	Reversible
CA2tr	calcium (Ca+2) transport, endoplasmic	Reversible
CALPN_3ca2_BIND	calpain-calcium ion binding	Irreversible
CAMK2_SERCA	CaMK2-mediated SERCA phosphorylation	Irreversible
CAM_3ca2_BIND	calmodulin-calcium ion binding	Irreversible
CAM_CAMK2_BIND	calmodulin (activated)-calmodulin dependent protein kinase 2 binding	Irreversible
CAM_CAMK2_CREB	CaM/CaMK2-mediated CREB phosphorylation	Irreversible
CAM_CAMK2_CREB-2	CaM/CaMK2-mediated CREB phosphorylation 2	Irreversible
CAM_CAMK2_IKK	CaM/CaMK2-mediated IKK phosphorylation	Irreversible
CAM_CAMK2_PHOS	CaM/CaMK2 autophosphorylation	Irreversible
CAM_CAMK2_TRAN	CaM/CaMK2 transport	Irreversible
CAM_CAMK2_TRAN2	CaM/CaMK2 transport 2	Irreversible
CDC42_DEPH	Cdc42 dephosphorylation	Irreversible

CDC42_PHOS	Cdc42 phosphorylation	Irreversible
CDC42_VAV1_BIND	Cdc42-Vav1 binding	Irreversible
CHOLtu	Choline uniport	Reversible
CK2_IKBB	CK2-mediated IkBb phosphorylation	Irreversible
CK2_NFKB	casein kinase 2-mediated NF-kB (p50/p65) phosphorylation	Irreversible
CK2_NFKB_P4K	NF-kB P4 kinase (CK2)	Irreversible
CK2_PHOS	casein kinase 2 autophosphorylation	Irreversible
COAt	CoA transporter	Reversible
COAtn	coenzyme A transport, nuclear	Reversible
CPGCIGC_TLRL9	Toll-like receptor 9 ligand (CpG chromatin IgG2a complexes)	Irreversible
CREB_CBP_BIND	CREB/CRE-CBP binding	Irreversible
CREB_CBP_DISS	CREB/CRE site/CBP dissociation	Irreversible
CREB_CBP_DISS2	CREB/CRE site/CBP dissociation 2	Irreversible
CREB_CRE_BIND	CREB-CRE site binding	Irreversible
CREB_DIME	CRE-binding protein dimerization	Irreversible
CSGA_TLRL2	Toll-like receptor 2 ligand (CsgA)	Irreversible
CTAK1_KSR1	CTAK1-mediated KSR1 phosphorylation	Irreversible
CTAK1_PHOS	C-TAK1 autophosphorylation	Irreversible
DAGK_PHOS	DAGK phosphorylation	Irreversible
DAGK_dag_hs	DAGK-mediated dag_hs phosphorylation	Irreversible
DAGt	diacylglycerol 2 (homo sapiens) transport	Reversible
DCLDLPP_TLRL2/6	Toll-like receptor 2/6 ligand (Diacetylated lipopeptides)	Irreversible
DCLLPP_TLRL2/6	Toll-like receptor 2/6 ligand (Diacyl lipopeptides)	Irreversible
DSRNA_TLRL3	Toll-like receptor 3 ligand (Double stranded RNA)	Irreversible
EEA1_DIME	EEA1 dimerization	Irreversible
EEA1_pail3p_hs_BIND	EEA1(dimer)-PI(3)P binding	Irreversible
ELK1_DEPH	PP2B-mediated Elk-1 dephosphorylation	Irreversible
ELK1_SRE_BIND	Elk1-SRE site binding	Irreversible
ENVP_TLRL4	Toll-like receptor 4 ligand (Envelope protein)	Irreversible
FADD_PCASP8_BIND	FADD-pro caspase8 binding	Irreversible
FADD_PCASP8_BIND2	FADD-pro caspase8 binding 2	Irreversible
FBNG_TLRL4	Toll-like receptor 4 ligand (Fibrinogen)	Irreversible
FLGN_TLRL5	Toll-like receptor 5 ligand (Flagellin)	Irreversible
FMAP3K1_PROC	full length MAP3K1 processing	Irreversible
FOS_DEGR	c-Fos degradation	Irreversible
FOS_DEPH	PP2A-mediated c-Fos dephosphorylation	Irreversible
FOS_JUN_BIND	cFos-cJun binding	Irreversible
FUSP_TLRL4	Toll-like receptor 4 ligand (Fusion protein)	Irreversible
GCSPL_TLRL2	Toll-like receptor 2 ligand (Glycoinositol phospholipids)	Irreversible
GENERIC_DA_DACL	unknown deacetylase deacetylation	Irreversible
GENERIC_K_PHOS	unknown kinase autophosphorylation	Irreversible
GENERIC_K_RAF1	unknown kinase phosphorylation of Raf-1	Irreversible
GENERIC_PPDA_NFKB	unknown mechanism of NF-kB (p50/p65) dephosphorylation/deacetylation	Irreversible
GENERIC_PPDA_NFKB2	unknown mechanism of NF-kB (p50/p65) dephosphorylation/deacetylation 2	Irreversible
GENERIC_PP_DEPH	unknown phosphatase dephosphorylation (regeneration)	Irreversible
GENERIC_PP_NFKB	unknown mechanism of NF-kB (p50/p65) dephosphorylation	Irreversible
GLC_TLRL2	Toll-like receptor 2 ligand (Glycolipids)	Irreversible
GP91_P22PHOX_BIND	gp91-p22 phox binding	Irreversible
GSK3B_NFKB	GSK3beta-mediated NF-kB (p50/p65) phosphorylation	Irreversible
GSK3B_NFKB_P3K	NF-kB P3 kinase (GSK3b)	Irreversible
GSK3B_PHOS	GSK3B autophosphorylation	Irreversible
GSK3B_TRAN	GSK3B transport	Irreversible
GSK3B_TRAN2	GSK3B transport 2	Irreversible
H2O2t	hydrogen peroxide transport via diffusion	Reversible
H2Ot	H2O transport via diffusion	Reversible
H2Otn	H2O transport, nuclear	Reversible

HDAC3_DACL	histone deacetylase 3 deacetylation	Irreversible
HNRNPA1_DEPH	PP2A-mediated hnRNPA1 dephosphorylation	Irreversible
HSP60_TLRL4	Toll-like receptor 4 ligand (Heat shock protein 60)	Irreversible
HSP70_TLRL2	Toll-like receptor 2 ligand (heat shock protein 70)	Irreversible
HSP70_TLRL4	Toll-like receptor 4 ligand (heat shock protein 70)	Irreversible
Ht	proton diffusion	Reversible
Htl	proton transport, lysosome	Reversible
Htn	proton transport, nuclear	Reversible
IKBA_DEGR	I-kappa-B-alpha degradation	Irreversible
IKBA_DEGR2	I-kappa-B-alpha degradation 2	Irreversible
IKBA_TRAN	IkBa transport	Irreversible
IKBA_UBIQ	IkBa ubiquitination	Irreversible
IKBA_UBIQ2	IkBa ubiquitination 2	Irreversible
IKBA_UBIQ3	IkBa ubiquitination 3	Irreversible
IKBA_UBIQ4	IkBa ubiquitination 4	Irreversible
IKBB_DEGR	I-kappa-B-beta degradation (2 phos, 1 ubi form)	Irreversible
IKBB_DEGR2	I-kappa-B-beta degradation 2 (2 phos, 2 ubi form)	Irreversible
IKBB_TRAN	IkBb transport	Irreversible
IKBB_UBIQ	IkBb ubiquitination	Irreversible
IKKE_IRF3	IKKe-mediated IRF3 phosphorylation	Irreversible
IKKE_NFKB	IKKepsilon-mediated NF-kB (p50/p65) phosphorylation	Irreversible
IKKE_NFKB2	IKKepsilon-mediated NF-kB (p50/p65) phosphorylation 2	Irreversible
IKKE_NFKB_P3K	NF-kB P3 kinase (IKKe)	Irreversible
IKKE_NFKB_P5K	NF-kB P5 kinase (IKKe)	Irreversible
IKKE_PHOS	IKKepsilon autophosphorylation	Irreversible
IKK_6PHOS	IKK autophosphorylation	Irreversible
IKK_IKBA	IKK-mediated IkBa phosphorylation	Irreversible
IKK_IKBB	IKK-mediated IkBb phosphorylation	Irreversible
IKK_MAP3K8	IKK-mediated MAP3K8 phosphorylation	Irreversible
IKK_MAP3K8(p52)_NFKB	IKK-mediated MAP3K8(p52)/NFkB phosphorylation	Irreversible
IKK_NFKB	IKK-mediated NF-kB (p50/p65) phosphorylation	Irreversible
IKK_NFKB(p105)	IKK-mediated NFkB(p105) precursor phosphorylation	Irreversible
IKK_NFKB2	IKK-mediated NF-kB (p50/p65) phosphorylation 2	Irreversible
IKK_NFKB3	IKK-mediated NF-kB (p50/p65) phosphorylation 3	Irreversible
IKK_NFKB_P3K	NF-kB P3 kinase (IKKbeta)	Irreversible
IKK_NFKB_P5K	NF-kB P5 kinase (IKKbeta)	Irreversible
IKK_NFKB_P5K2	NF-kB P5 kinase (IKKalpha)	Irreversible
IKK_PKR_BIND	IKK/PKR (dimer) binding	Irreversible
IKK_PKR_PHOS	IKK/PKR (dimer) phosphorylation	Irreversible
IKK_PKR_TRAF6_BIND	IKK-PKR (dimer)/TRAF6 (dimer) binding	Irreversible
IKK_PKR_TRAF6_PHOS	IKK/PKR (dimer)/TRAF6 (dimer) phosphorylation	Irreversible
IKK_RIP1_TICAM1P_BIND	IKK-RIP1/TICAM1P binding	Irreversible
IKK_RIP1_TICAM1P_PHOS	IKK/RIP1/TICAM1P phosphorylation	Irreversible
IKK_RIP2_NOD1P_BIND	IKK-RIP2/NOD1P binding	Irreversible
IKK_RIP2_NOD1P_PHOS	IKK/RIP2/NOD1P phosphorylation	Irreversible
IKK_RIP2_NOD2P_BIND	IKK-RIP2/NOD2P binding	Irreversible
IKK_RIP2_NOD2P_PHOS	IKK/RIP2/NOD2P phosphorylation	Irreversible
IKK_RIP2_TRIP6_TRAF2_BIND	IKK-RIP2/TRIP6/TRAF2 binding	Irreversible
IKK_RIP2_TRIP6_TRAF2_PHOS	IKK/RIP2/TRIP6/TRAF2 phosphorylation	Irreversible
IKK_SRC_BIND	IKKalpha/beta/gamma-cSrc binding	Irreversible
IL1/L/AP_PHOS	IL1R1/L/AP phosphorylation	Irreversible
IL1/L_IL1RAP_BIND	IL1/L-IL1RAP binding	Irreversible
IL1A_IL1R1L	IL-1R1 ligand (IL-1a)	Irreversible
IL1A_TRAN	IL-1a transport	Irreversible
IL1B_IL1R1L	IL-1R1 ligand (IL-1b)	Irreversible
IL1B_TRAN	IL-1b transport	Irreversible
IL1R1_BIND	IL-1R1 ligand binding	Irreversible
IL1R1_PI3K1A	IL1R1-mediated PI3K class 1A phosphorylation	Irreversible

IL1R2_IL1RAP_BIND	IL1R2-IL1RAP binding	Irreversible
IMPA3_TRAN	importin alpha 3 transport	Irreversible
IMQ_TLRL7	Toll-like receptor 7 ligand (Imidazoquinoline)	Irreversible
IMQ_TLRL8	Toll-like receptor 8 ligand (Imidazoquinoline)	Irreversible
IP3R_mi145p_BIND	inositol 1,4,5-trisphosphate-receptor binding	Irreversible
IRAK1C_TOLLIP_BIND	IRAK1C-TOLLIP binding	Irreversible
IRAK1_DEGR	IRAK1 degradation	Irreversible
IRAK1_ECSIT_BIND	IRAK1/TRAF6 (dimer)/TIFA/Ubc13/Uev1A-ECSIT binding	Irreversible
IRAK1_ECSIT_FMAP3K1_BIND	IRAK1/TIFA/TRAF6 (dimer)/Ubc13/Uev1A/ECSIT-full length MAP3K1-caspase3 binding	Irreversible
IRAK1_MAP3K3_4PHOS	IRAK1/TRAF6 (dimer)/TIFA/Ubc13/Uev1A/MAP3K3 autophosphorylation	Irreversible
IRAK1_MAP3K3_BIND	IRAK1/TRAF6 (dimer)/TIFA/Ubc13/Uev1A-MAP3K3 binding	Irreversible
IRAK1_MAP3K5_BIND	IRAK1/TRAF6 (dimer)/TIFA/Ubc13/Uev1A-MAP3K5 binding	Irreversible
IRAK1_MAP3K7_4PHOS	IRAK1/TRAF6 (dimer)/TIFA/Ubc13/Uev1A/MAP3K7/IP23/1 phosphorylation	Irreversible
IRAK1_MAP3K7_BIND	IRAK1/TRAF6 (dimer)/TIFA/Ubc13/Uev1A-MAP3K7/IP23/1 binding	Irreversible
IRAK1_MAP3K7_DISS	TRAF6 (dimer)/Ubc13/Uev1A/MAP3K7/IP23/1-IRAK1-TIFA dissociation	Irreversible
IRAK1_PLNO3_BIND	IRAK1-Pellino3 binding	Irreversible
IRAK1_TIFA_3UBIQ	IRAK1/TIFA/TRAF6 (dimer)/Ubc13/Uev1A triubiquitination	Irreversible
IRAK1_TIFA_A20_BIND	IRAK1/TIFA/TRAF6 (dimer)/Ubc13/Uev1A-A20 binding	Irreversible
IRAK1_TIFA_CYLD_BIND	IRAK1/TIFA/TRAF6 (dimer)/Ubc13/Uev1A-CYLD binding	Irreversible
IRAK1_TIFA_DEBQ	CYLD-mediated IRAK1/TIFA/TRAF6 (dimer)/Ubc13/Uev1A deubiquitination	Irreversible
IRAK1_TIFA_DEBQ2	A20-mediated IRAK1/TIFA/TRAF6 (dimer)/Ubc13/Uev1A deubiquitination	Irreversible
IRAK1_TOLLIP_BIND	IRAK1-TOLLIP binding	Irreversible
IRAK_MYD_TIR_2PHOS	IRAK1/IRAK4/MyD88 (dimer)/TIR autophosphorylation 2	Irreversible
IRAK_MYD_TIR_BIND	IRAK1-MyD88 (dimer)/TIR/IRAK4 binding	Irreversible
IRAK_MYD_TIR_PHOS	IRAK1/IRAK4/MyD88 (dimer)/TIR autophosphorylation	Irreversible
IRAK_TOLLIP_TRAF6_BIND	TIR/MyD88/IRAK4-IRAK1c/TOLLIP-TRAF6 binding	Irreversible
IRAK_TRAF6_BIND	IRAK1/IRAK4/MyD88 (dimer)/TIR-Ubc13-Uev1A-TIFA-TRAF6 (dimer) binding	Irreversible
IRAK_TRAF6_DISS	IRAK1/TRAF6 (dimer)/TIFA/Ubc13/Uev1A-TIR/MyD88 (dimer)/IRAK4 dissociation	Irreversible
IRAK_TRAF6_TIRAP_BIND	IRAK1/IRAK4/MyD88 (dimer)/TIRAP/TIR-TRAF6 (dimer)-Ubc13-Uev1A-TIFA binding	Irreversible
IRAK_TRAF6_TIRAP_DISS	IRAK1/TRAF6 (dimer)/TIFA/Ubc13/Uev1A-IRAK4/MyD88 (dimer)/TIR/TIRAP dissociation	Irreversible
IRF3_DIME	IRF3 (2 phosphorylated) dimerization	Irreversible
IRF3_ISRE_BIND	IRF3 (dimer)-ISRE binding	Irreversible
IRF3_TRAN	IRF3 (dimer) transport	Irreversible
IRF7_ISRE_BIND	IRF7-ISRE binding	Irreversible
IRF7_TRAN	IRF7 (dimer) transport	Irreversible
JUN_DEGR	c-Jun degradation	Irreversible
JUN_DIME	c-Jun dimerization	Irreversible
KSR1_DEPH1	14-3-3/KSR1/MKK1 disassociation	Irreversible
KSR1_RAF1_MAPK3/1_7PHOS	KSR1/Raf-1/MAPK3/1/MAP2K1 autophosphorylation	Irreversible
KSR1_RAF1_MAPK3/1_9PHOS	KSR1/Raf-1/MAPK3/1/MAP2K1 autophosphorylation 2	Irreversible
KSR1_RAF1_MAPK3/1_BIND	KSR1/MAP2K1-Raf1-MAPK3/1 binding	Irreversible
KSR1_RAF1_MAPK3/1_DISS	KSR1/MAP2K1-Raf1-MAPK3/1 dissocation	Irreversible
LAM_TLRL2	Toll-like receptor 2 ligand (Lipoarabinomannan)	Irreversible
LPPS_TLRL2	Toll-like receptor 2 ligand (Lipopeptides)	Irreversible
LPS_TLRL2	Toll-like receptor 2 ligand (Lipopolysaccharide)	Irreversible
LPS_TLRL4	Toll-like receptor 4 ligand (Lipopolysaccharide)	Irreversible
LP_TLRL2	Toll-like receptor 2 ligand (Lipoprotein)	Irreversible
LTA_TLRL2	Toll-like receptor 2 ligand (Lipoteichoic acid)	Irreversible

LTA_TLRL2/6	Toll-like receptor 2/6 ligand (Lipoteichoic acid)	Irreversible
LXR_TLRL7	Toll-like receptor 7 ligand (Loxoribine)	Irreversible
MAP2K1_MAPK3/1	MAP2K1-mediated MAPK3/1 phosphorylation	Irreversible
MAP2K3_MAPK11	MAP2K3-mediated MAPK11 phosphorylation	Irreversible
MAP2K3_MAPK14	MAP2K3-mediated MAPK14 phosphorylation	Irreversible
MAP2K3_TRAN	MAP2K3 transport	Irreversible
MAP2K3_TRAN2	MAP2K3 transport 2	Irreversible
MAP2K4_MAPK11	MAP2K4-mediated MAPK11 phosphorylation	Irreversible
MAP2K4_MAPK14	MAP2K4-mediated MAPK14 phosphorylation	Irreversible
MAP2K4_MAPK8	MAP2K4-mediated MAPK8 phosphorylation	Irreversible
MAP2K6_MAPK11	MAP2K6-mediated MAPK11 phosphorylation	Irreversible
MAP2K6_MAPK14	MAP2K6-mediated MAPK14 phosphorylation	Irreversible
MAP2K6_TRAN	MAP2K6 transport	Irreversible
MAP2K6_TRAN2	MAP2K6 transport 2	Irreversible
MAP2K7_MAPK8	MAP2K7-mediated MAPK8 phosphorylation	Irreversible
MAP3K14_IKK	MAP3K14-mediated IKK phosphorylation	Irreversible
MAP3K1_DEGR	MAP3K1 degradation	Irreversible
MAP3K1_DEGR2	MAP3K1 degradation 2	Irreversible
MAP3K1_IKK	MAP3K1-mediated IKK phosphorylation	Irreversible
MAP3K1_MAP2K1	MAP3K1-mediated MAP2K1 phosphorylation	Irreversible
MAP3K1_MAP2K4	MAP3K1-mediated MAP2K4 phosphorylation	Irreversible
MAP3K1_MAP2K7	MAP3K1-mediated MAP2K7 phosphorylation	Irreversible
MAP3K1_PHOS	MAP3K1 autophosphorylation	Irreversible
MAP3K1_UBIQ	MAP3K1 ubiquitination	Irreversible
MAP3K3_MAP2K3	MAP3K3-mediated MAP2K3 phosphorylation	Irreversible
MAP3K3_MAP2K4	MAP3K3-mediated MAP2K4 phosphorylation	Irreversible
MAP3K3_MAP2K6	MAP3K3-mediated MAP2K6 phosphorylation	Irreversible
MAP3K3_MAP2K7	MAP3K3-mediated MAP2K7 phosphorylation	Irreversible
MAP3K5_MAP2K3	MAP3K5-mediated MAP2K3 phosphorylation	Irreversible
MAP3K5_MAP2K4	MAP3K5-mediated MAP2K4 phosphorylation	Irreversible
MAP3K5_MAP2K6	MAP3K5-mediated MAP2K6 phosphorylation	Irreversible
MAP3K5_PHOS	MAP3K5 autophosphorylation	Irreversible
MAP3K5_TRXRED_BIND	MAP3K5-reduced thioredoxin binding	Irreversible
MAP3K5_TRXRED_DISS	MAP3K5-reduced thioredoxin dissociation	Irreversible
MAP3K7/IP123_BIND	MAP3K7-IP1-IP2/3 binding	Irreversible
MAP3K7IP_TRAF6_3PHOS	MAP3K7/IP23/1/TRAF6 (dimer)/Ubc13/Uev1A autophosphorylation	Irreversible
MAP3K7IP_TRAF6_DEGR	MAP3K7/IP/TRAF6(dimer)/Ubc13/Uev1A degradation	Irreversible
MAP3K7IP_TRAF6_UBIQ	MAP3K7/IP23/1/TRAF6 (dimer)/Ubc13/Uev1A autoubiquitination	Irreversible
MAP3K7_IKK	MAP3K7-mediated IKK phosphorylation	Irreversible
MAP3K7_MAP2K3	MAP3K7-mediated MAP2K3 phosphorylation	Irreversible
MAP3K7_MAP2K4	MAP3K7-mediated MAP2K4 phosphorylation	Irreversible
MAP3K7_MAP2K6	MAP3K7-mediated MAP2K6 phosphorylation	Irreversible
MAP3K7_MAP2K7	MAP3K7-mediated MAP2K7 phosphorylation	Irreversible
MAP3K7_MAP3K14	MAP3K7-mediated MAP3K14 phosphorylation	Irreversible
MAP3K7_MAPK14	MAP3K7-mediated MAP2K-independent MAPK14 phosphorylation	Irreversible
MAP3K8(p52)_DEGR	MAP3K8 (p52) degradation	Irreversible
MAP3K8(p58)_DEGR	MAP3K8 (p58) degradation	Irreversible
MAP3K8(p58)_KSR2_BIND	MAP3K8 (p58)-KSR2 binding	Irreversible
MAP3K8_MAP2K1	MAP3K8-mediated MAP2K1 phosphorylation	Irreversible
MAP3K8_MAP3K14	MAP3K8-mediated MAP3K14 phosphorylation	Irreversible
MAP3K8_NFKB_BIND	MAP3K8 (p58/p52)-NFkB(p105) binding	Irreversible
MAP3K8_NFKB_DISS	MAP3K8(p58)-p52/NFkB dissociation	Irreversible
MAPK11_ATF2	MAPK11-mediated ATF2 phosphorylation	Irreversible
MAPK11_ELK1	MAPK11-mediated Elk-1 phosphorylation	Irreversible
MAPK11_MAPKAPK2	MAPK11-mediated MAPKAPK2 phosphorylation	Irreversible
MAPK11_MAPKAPK2_BIND	MAPK11-MAPKAPK2 binding	Irreversible
MAPK11_MAPKAPK2_TRAN	MAPK11/MAPKAPK2 transport	Irreversible
MAPK11_MBP	MAPK11-mediated MBP phosphorylation	Irreversible

MAPK11_MKNK1	MAPK11-mediated MKNK1 phosphorylation	Irreversible
MAPK11_MSK1	MAPK11-mediated MSK1 phosphorylation	Irreversible
MAPK11_TRAN2	MAPK11 transport 2	Irreversible
MAPK14_ATF2	MAPK14-mediated ATF2 phosphorylation	Irreversible
MAPK14_ELK1	MAPK14-mediated Elk-1 phosphorylation	Irreversible
MAPK14_MAP3K7IP_TRAF6	MAPK14-mediated MAP3K7/IP23/1/TRAF6 (dimer)/Ubc13/Uev1A phosphorylation	Irreversible
MAPK14_MAPKAPK2	MAPK14-mediated MAPKAPK2 phosphorylation	Irreversible
MAPK14_MAPKAPK2_BIND	MAPK14-MAPKAPK2 binding	Irreversible
MAPK14_MAPKAPK2_TRAN	MAPK14/MAPKAPK2 transport	Irreversible
MAPK14_MBP	MAPK14-mediated MBP phosphorylation	Irreversible
MAPK14_MKNK1	MAPK14-mediated MKNK1 phosphorylation	Irreversible
MAPK14_MSK1	MAPK14-mediated MSK1 phosphorylation	Irreversible
MAPK14_TRAN2	MAPK14 transport 2	Irreversible
MAPK3/1_DEPH	MKP-mediated MAPK3/1 dephosphorylation	Irreversible
MAPK3/1_DIME	MAPK3/1 dimerization	Irreversible
MAPK3/1_ELK1	MAPK3/1-mediated Elk-1 phosphorylation	Irreversible
MAPK3/1_FOS	MAPK3/1-mediated c-Fos hexaphosphorylation	Irreversible
MAPK3/1_MBP	MAPK3/1-mediated MBP phosphorylation	Irreversible
MAPK3/1_MKNK1	MAPK3/1-mediated MKNK1 phosphorylation	Irreversible
MAPK3/1_MKNK2	MAPK3/1-mediated MKNK2 phosphorylation	Irreversible
MAPK3/1_MSK1	MAPK3/1-mediated MSK1 phosphorylation	Irreversible
MAPK3/1_MYC	MAPK3/1-mediated c-Myc phosphorylation	Irreversible
MAPK3/1_TRAN	MAPK3/1 transport	Irreversible
MAPK3/1_TRAN2	MAPK3/1 transport 2	Irreversible
MAPK8_ATF2	MAPK8-mediated AFT2 phosphorylation	Irreversible
MAPK8_FOS	MAPK8-mediated c-Fos hexaphosphorylation	Irreversible
MAPK8_JUN	MAPK8-mediated c-Jun diphosphorylation	Irreversible
MAPK8_TRAN	MAPK8 (JNK) transport	Irreversible
MAPK8_TRAN2	MAPK8 transport 2	Irreversible
MAPKAPK2_2PHOS	MAPKAPK2 autophosphorylation	Irreversible
MAPKAPK2_CREB	MAPKAPK2-mediated CREB phosphorylation	Irreversible
MAPKAPK2_HSP27	MAPKAPK2-mediated HSP27 phosphorylation	Irreversible
MAPKAPK2_TRAN2	MAPKAPK2 transport 2	Irreversible
MD2_DIME	MD-2 dimerization	Irreversible
MI145Pt	1D-myo-Inositol 1,4,5-trisphosphate transport, cytoplasm	Reversible
MKNK1_EIF4E	MKNK1-mediated eIF-4E phosphorylation	Irreversible
MKP_DEPH	MAP kinase phosphatase dephosphorylation	Irreversible
MRAP_TLRL2	Toll-like receptor 2 ligand (Mannuronic acid polymer)	Irreversible
MRAP_TLRL4	Toll-like receptor 4 ligand (Mannuronic acid polymer)	Irreversible
MRNA_TLRL3	Toll-like receptor 3 ligand (mRNA)	Irreversible
MSK1_5PHOS	MSK1 autophosphorylation	Irreversible
MSK1_CREB	MSK1-mediated CREB phosphorylation	Irreversible
MSK1_HH3	MSK1-mediated histone H3 phosphorylation	Irreversible
MSK1_HMG14	MSK1-mediated HMG-14 phosphorylation	Irreversible
MSK1_NFKB	MSK1-mediated NF-kB (p50/p65) phosphorylation	Irreversible
MSK1_NFKB_P1K	NF-kB P1 kinase (MSK1)	Irreversible
MSK1_TRAN	MSK1-5P transport	Reversible
MSK1_TRAN2	MSK1 transport	Reversible
MYD88_DIME	MyD88 dimerization	Irreversible
MYD88_IRAK_TOLLIP_2PHOS	MyD88/TIR/IRAK1/IRAK4/TOLLIP autophosphorylation 2	Irreversible
MYD88_IRAK_TOLLIP_BIND	IRAK1/TOLLIP-MyD88/TIR/IRAK4 binding	Irreversible
MYD88_IRAK_TOLLIP_DISS	MyD88 (dimer)/IRAK1/IRAK4/TIR-TOLLIP dissociation	Irreversible
MYD88_IRAK_TOLLIP_PHOS	MyD88/TIR/IRAK1/IRAK4/TOLLIP autophosphorylation	Irreversible
MYD88_IRF7_BIND	TLR9-mediated MyD88-IRF7-TRAF6 (dimer) binding	Irreversible
MYD88_IRF7_BIND2	TLR7-mediated MyD88-IRF7-TRAF6 (dimer) binding	Irreversible
MYD88_IRF7_BIND3	TLR8-mediated MyD88-IRF7-TRAF6 (dimer) binding	Irreversible
MYD88_IRF7_DISS	TBK1-mediated MyD88/IRF7/TRAF6 (dimer) dissociation	Irreversible

MYD88_IRF7_DISS2	IKKe-mediated MyD88/IRF7/TRAF6 (dimer) dissociation	Irreversible
MYD88_MYD88S_BIND	MyD88-MyD88s binding	Irreversible
NADPHt	NADPH transporter, cytoplasm	Reversible
NADPt	NADP transporter	Reversible
NFKB(p105)_BTRCP2_BIND	NF-kB(p105)-beta TrCP2 binding	Irreversible
NFKB(p105)_DEGR	NF-kB (p105) degradation	Irreversible
NFKB(p105)_DEGR2	NF-kB (p105) degradation 2	Irreversible
NFKB(p105)_DEGR3	NF-kB (p105) degradation 3	Irreversible
NFKB(p105)_PROC	NF-kB (p105) processing	Irreversible
NFKB(p105)_PROC2	NF-kB (p105) processing 2	Irreversible
NFKB(p105)_UBCH5A_BIND	NF-kB (p105)-UbcH5A binding	Irreversible
NFKB(p105)_UBIQ	NF-kB (p105) ubiquitination	Irreversible
NFKB(p105)_UBIQ2	NF-kB (p105) ubiquitination 2	Irreversible
NFKB(p105/p50)_BIND	NF-kB (p105/p50) binding	Irreversible
NFKB(p105/p50)_DISS	NF-kB (p105/p50) dissociation	Irreversible
NFKB(p50)_BCL3_BIND	NFkB(p50) (dimer)-Bcl3 binding	Irreversible
NFKB(p50)_BCL3_TRAN	NFkB(p50) (dimer)/Bcl3 transport	Irreversible
NFKB(p50)_IKBZ_BIND	NFkB(p50) (dimer)-IkBzeta binding	Irreversible
NFKB(p50)_IMPA3_BIND	NFkB (p50)-importin alpha 3 binding	Irreversible
NFKB(p50)_IMPA3_DISS	NFkB (p50)-importin alpha 3 dissociation	Irreversible
NFKB(p50)_IMPA3_TRAN	NF-kB(p50)/importin alpha 3 transport	Irreversible
NFKB(p50)_KB_SITE_BIND	NFkB(p50) (dimer)-kB site binding	Irreversible
NFKB(p50)_KB_SITE_DISS	NFkB(p50) (dimer)-kB site-HDAC1 dissociation	Irreversible
NFKB(p50)_TRAN	NF-kB (p50) transport	Irreversible
NFKB(p50/p65)_CBP-5P3A_BIND	NF-kappa-B (p50/p65)-CBP binding 2	Irreversible
NFKB(p50/p65)_CBP-5P_BIND	NF-kappa-B (p50/p65)-CBP binding	Irreversible
NFKB(p65)_DEGR	NF-kB (p65) degradation	Irreversible
NFKB(p65)_DEGR2	NF-kB (p65) degradation 2	Irreversible
NFKB(p65)_SOCS1_BIND	NF-kB (p65)-SOCS1 binding	Irreversible
NFKB(p65)_UBIQ	NF-kB (p65) ubiquitination	Irreversible
NFKB_2ACTL	CBP-mediated NF-kappa-B acetylation 3	Irreversible
NFKB_3ACTL	CBP-mediated NF-kappa-B acetylation	Irreversible
NFKB_3DACL	HDAC3-mediated NF-kappa-B deacetylation	Irreversible
NFKB_5ACTL	CBP-mediated NF-kappa-B acetylation 2	Irreversible
NFKB_5DACL	HDAC3-mediated NF-kappa-B deacetylation 2	Irreversible
NFKB_CBP_KB_BIND	NF-kB (p50/p65)-CBP-kB site binding	Irreversible
NFKB_CBP_KB_DISS	NF-kB(p5p/p65)-CBP-KB site dissociation	Irreversible
NFKB_CBP_KB_DISS2	NF-kB(p5p/p65)-CBP-KB site dissociation 2	Irreversible
NFKB_IKBA_BIND	NFkB (p50/p65)-IkBa binding	Irreversible
NFKB_IKBA_BIND2	NFkB (p50/p65)-IkBa binding 2	Irreversible
NFKB_IKBA_BIND3	NFkB (p50/p65)-IkBa binding 3	Irreversible
NFKB_IKBA_BIND4	NFkB (p50/p65)-IkBa binding 4	Irreversible
NFKB_IKBA_BTRCP1_BIND	NFkB/IkBa-betaTrCP1 binding	Irreversible
NFKB_IKBA_BTRCP2_BIND	NFkB/IkBa-betaTrCP2 binding	Irreversible
NFKB_IKBA_DISS	NFkB (p50/p65)-IkBa dissociation	Irreversible
NFKB_IKBA_TRAN	NFkB (p50/p65)/IkBa transport	Irreversible
NFKB_IKBA_TRAN2	NFkB (p50/p65)/IkBa transport 2	Irreversible
NFKB_IKBA_TRAN3	NFkB (p50/p65)/IkBa transport 3	Irreversible
NFKB_IKBA_TRAN4	NFkB (p50/p65)/IkBa transport 4	Irreversible
NFKB_IKBA_TRAN5	NFkB (p50/p65)/IkBa transport 5	Irreversible
NFKB_IKBA_UBCH5_BIND	NFkB/IkBa/UbcH5 binding	Irreversible
NFKB_IKBA_UBCH5_BIND2	NFkB/IkBa/UbcH5 binding 2	Irreversible
NFKB_IKBB_BIND	NFkB(p50/p65)-IkBb binding	Irreversible
NFKB_IKBB_BIND2	NFkB (p50/p65)-IkBb binding 2	Irreversible
NFKB_IKBB_BTRCP2_BIND	NFkB/IkBb-bTrCP2 binding	Irreversible
NFKB_IKBB_DISS	NFkB (p50/p65)-IkBb dissociation	Irreversible
NFKB_IKBB_TRAN	NFkB (p50/p65)/IkBb transport	Irreversible
NFKB_P1_PHOS	NF-kB(P1 phosphorylated) (p50/p65) phosphorylation	Irreversible

NFKB_P2_PHOS	NF-kB(P2 phosphorylated) (p50/p65) phosphorylation	Irreversible
NFKB_P3_PHOS	NF-kB(P3 phosphorylated) (p50/p65) phosphorylation	Irreversible
NFKB_P4_PHOS	NF-kB(P4 phosphorylated) (p50/p65) phosphorylation	Irreversible
NFKB_P5_PHOS	NF-kB(P5 phosphorylated) (p50/p65) phosphorylation	Irreversible
NFKB_PKAC_DISS	NF-kB(p50)/NF-kB(p65)-PKAc dissociation	Irreversible
NFKB_PKAC_IKBA_BIND	NFkB(p50)-NFkB(p65)-IkBa-PKAc binding	Irreversible
NFKB_PKAC_IKBA_DISS	NFkB(p50)/NFkB(p65)/PKAc-IkBa dissociation	Irreversible
NFKB_PKAC_IKBB_BIND	NFkB(p50)-NFkB(p65)-IkBb-PKAc binding	Irreversible
NFKB_PKAC_IKBB_DISS	NFkB(p50)/NFkB(p65)/PKAc-IkBb dissociation	Irreversible
NFKB_TRAN	NF-kB (p50/p65) (5 phosphorylated) transport	Irreversible
NOD1P_BIND	diaminopimelic acid-mediated NOD1 activation	Irreversible
NOD2P_BIND	NOD2-muramyl dipeptide binding	Irreversible
NSF_EEA1_RAB5_BIND	NSF/stxn13-GTP/Rab5-EEA1/PI(3)P binding	Irreversible
NSF_STXN13_BIND	NSF-Syntaxin 13 binding	Irreversible
OLSCHYA_TLRL4	Toll-like receptor 4 ligand (Oligosaccharides of hyaluronic acid)	Irreversible
OMPA_TLRL2	Toll-like receptor 2 ligand (Outer membrane protein A)	Irreversible
OSPALP_TLRL2/6	Toll-like receptor 2/6 ligand (Outer surface protein A lipoprotein)	Irreversible
PAIL345P_HSt	phosphatidylinositol 3,4,5-trisphosphate transport, cytoplasm	Reversible
PAIL4P_HSt	phosphatidylinositol 4-phosphate cytoplasmic transport (diffusion)	Reversible
PAIL_HSt	phosphatidylinositol cytoplasmic transport (diffusion)	Reversible
PAK1_CDC42_BIND	PAK1-Cdc42/GTP binding	Irreversible
PAK1_MAP2K1	PAK1-mediated MAP2K1 phosphorylation	Irreversible
PAK1_MAP2K1-2	PAK1-mediated MAP2K1 phosphorylation 2	Irreversible
PAK1_PHOS	PAK1 autophosphorylation	Irreversible
PAK1_RAC1_BIND	PAK1-Rac1/GTP binding	Irreversible
PAK1_SRCK_RAF1	PAK1- and SRCK-mediated Raf-1 phosphorylation	Irreversible
PAK1_SRCK_RAF1-2	PAK1- and SRCK-mediated Raf-1 phosphorylation 2	Irreversible
PA_HSt	phosphatidate transport, cytoplasm	Reversible
PCASP8_PROC	pro-caspase8 processing	Irreversible
PCASP9_NOD1P_BIND	pro caspase9-NOD1 (activated) binding	Irreversible
PCASP9_NOD2P_BIND	pro caspase9-NOD2 (activated) binding	Irreversible
PCASP9_PROC	pro-caspase 9 processing	Irreversible
PCASP9_PROC2	pro-caspase 9 processing 2	Irreversible
PCHOLt	phosphatidylcholine (homo sapiens) transport	Reversible
PDK1_AKT	PDK1-mediated Akt phosphorylation	Irreversible
PDK1_PHOS	PDK1 (activated) autophosphorylation	Irreversible
PDK1_PKCA	PDK1-mediated PKCa phosphorylation	Irreversible
PDK1_PKCB2	PDK1-mediated PKCb2 phosphorylation	Irreversible
PDK1_PKCD	PDK1-mediated PKCd phosphorylation	Irreversible
PDK1_PKCZ	PDK1-mediated PKCzeta phosphorylation	Irreversible
PDK1_pail345p_hs_BIND	PDK1-phosphatidylinositol(3,4,5)-trisphosphate binding	Irreversible
PHOX_GTP_BIND	gp91/p22-p40-p47-p67-Rac1/GTP binding	Irreversible
PHOX_GTP_BIND2	gp91/p22-p40-p47-p67-Rac1/GTP binding 2	Irreversible
PI34P5K_PHOS	PI(3,4)P5K phosphorylation	Irreversible
PI34P5K_pail34p_hs	PI(3,4)P5K mediated PI(3,4)P2 phosphorylation	Irreversible
PI3K1A_PHOS	PI3K class 1A phosphorylation	Irreversible
PI3K1A_pail45p_hs	PI3K class 1A mediated PI(4,5)P2 phosphorylation	Irreversible
PI3K3_BIND	phosphoinositide 3-kinase class 3 formation	Irreversible
PI3K3_RAB5_BIND	PI3K3-Rab5/GTP binding	Irreversible
PI3K3_RAB5_pail_hs_BIND	PI3K3/Rab5/GTP-phosphatidylinositol binding	Irreversible
PI3P4K_PHOS	PI(3)P4K phosphorylation	Irreversible
PI3P4K_pail3p_hs	PI(3)P4K-mediated PI(3)P phosphorylation	Irreversible
PI4P5K_PHOS	PI(4)P5K phosphorylation	Irreversible
PI4P5K_pail4p_hs	PI4P5K-mediated PI4P phosphorylation	Irreversible
PIt	Inorganic phosphate exchange, diffusion	Reversible
PItn	phosphate transport, nuclear	Reversible
PKAC_NFKB	PKAc-mediated NF-kB (p50/p65) phosphorylation	Irreversible
PKAC_NFKB(p50)	PKAc-mediated NFkB(p50) phosphorylation	Irreversible

PKAC_NFKB_P1K	NF-kB P1 kinase (PKAc)	Irreversible
PKAC_PHOS	PKAc autophosphorylation	Irreversible
PKA_CREB	PKA-mediated CREB phosphorylation	Irreversible
PKA_PHOS	protein kinase A autophosphorylation	Irreversible
PKA_RAF1	PKA-mediated Raf-1 phosphorylation	Irreversible
PKA_TRAN	protein kinase A transport	Reversible
PKA_TRAN2	protein kinase A (2 phosphorylated) transport	Reversible
PKCA_3PHOS	PKCalpha autophosphorylation	Irreversible
PKCA_ca2_BIND	PKCalpha-calcium ion binding	Irreversible
PKCA_ca2_P47PHOX	PKCalpha/calcium ion-mediated p47phox phosphorylation	Irreversible
PKCA_dag_ps_hs_BIND	PKCalpha-diacylglycerol-phosphatidylserine binding	Irreversible
PKCA_dag_ps_hs_P47PHOX	PKCalpha/diacylglycerol/phosphatidylserine-mediated p47phox phosphorylation	Irreversible
PKCB2_3PHOS	PKCbeta2 autophosphorylation	Irreversible
PKCB2_ca2_BIND	PKCbeta2-calcium ion binding	Irreversible
PKCB2_ca2_P47PHOX	PKCbeta2/calcium ion-mediated p47phox phosphorylation	Irreversible
PKCB2_dag_ps_hs_BIND	PKCbeta2-diacylglycerol-phosphatidylserine binding	Irreversible
PKCB2_dag_ps_hs_P47PHOX	PKCbeta2/diacylglycerol/phosphatidylserine-mediated p47phox phosphorylation	Irreversible
PKCD_2PHOS	PKCd autophosphorylation	Irreversible
PKCD_dag_ps_hs_BIND	PKCdelta-diacylglycerol-phosphatidylserine binding	Irreversible
PKCD_dag_ps_hs_P47PHOX	PKCdelta-mediated p47phox phosphorylation	Irreversible
PKCZ_HNRNPA1	PKCzeta-mediated hnRNPA1 phosphorylation	Irreversible
PKCZ_NFKB	PKCzeta-mediated NF-kB (p50/p65) phosphorylation	Irreversible
PKCZ_NFKB_P2K	NF-kB P2 kinase (PKCz)	Irreversible
PKCZ_P47PHOX	PKCzeta-mediated p47phox phosphorylation	Irreversible
PKCZ_TRAN	protein kinase C, zeta isoform transport	Irreversible
PKCZ_TRAN2	protein kinase C, zeta isoform transport 2	Irreversible
PKCZ_pa_hs_BIND	PKCzeta-phosphatidyl acid binding	Irreversible
PKCZ_pa_hs_PHOS	PKCzeta/phosphatidic acid autophosphorylation	Irreversible
PKCZ_ps_hs_BIND	PKCzeta-phosphatidylserine binding	Irreversible
PKCZ_ps_hs_PHOS	PKCzeta/phosphatidylserine autophosphorylation	Irreversible
PKR_2PHOS	PKR (dimer) autophosphorylation	Irreversible
PKR_DIME	PKR dimerization	Irreversible
PKR_EIF2A	PKR-mediated eIF2alpha phosphorylation	Irreversible
PKR_TRAF6_BIND	PKR(dimer)-TRAF6(dimer) binding	Irreversible
PLCB2_ACTI	PLC beta II activation	Irreversible
PLD_ACTI	phospholipase D activation	Irreversible
PLP_TLRL11	Toll-like receptor 11 ligand (Profilin-like protein)	Irreversible
PP2A_ABC_TRAN	protein phosphatase 2A transport	Reversible
PP2A_ABC_TRAN2	protein phosphatase (phosphorylated) 2A transport	Reversible
PP2A_DEPH	PP2A dephosphorylation (regeneration)	Irreversible
PP2B_DEPH	PP2B dephosphorylation	Irreversible
PP2C_B1_DEPH	protein phosphatase 2C, beta 1 isoform dephosphorylation	Irreversible
PP2C_B1_MAP3K7IP_TRAF6	PP2C beta 1 isoform mediated MAP3K7/IP23/1/TRAF6(dimer)/Ubc13/Uev1A dephosphorylation	Irreversible
PP2C_E_DEPH	protein phosphatase 2C, epsilon isoform dephosphorylation	Irreversible
PP2C_E_MAP3K7IP_TRAF6	PP2C epsilon isoform mediated MAP3K7/IP23/1/TRAF6(dimer)/Ubc13/Uev1A dephosphorylation	Irreversible
PPAP_DEPH	phosphatidic acid phosphatase dephosphorylation	Irreversible
PRE_IL1A_CALPN_3ca2_BIND	pre IL1a-calpain/calcium ion binding	Irreversible
PRE_IL1A_IL1R1L	IL-1R1 ligand (pre IL-1a)	Irreversible
PRE_IL1A_PROC	pre IL-1a processing	Irreversible
PRE_IL1A_TRAN	pre-IL1A transport	Reversible
PRE_IL1B_CASP1_BIND	pre IL1beta-caspase 1 binding	Irreversible
PRE_IL1B_PROC	pre IL-1beta processing	Irreversible
PRNS_TLRL2	Toll-like receptor 2 ligand (Porins)	Irreversible

PSCHPS_TLRL4	Toll-like receptor 4 ligand (Polysaccharide fragment of heparan sulphate)	Irreversible
PSM_TLRL2	Toll-like receptor 2 ligand (Phenol-soluble modulin)	Irreversible
PSM_TLRL2/6	Toll-like receptor 2/6 ligand (Phenol-soluble modulin)	Irreversible
PSt3	phosphatidylserine (homo sapiens) transport	Reversible
PTG_TLRL2	Toll-like receptor 2 ligand (Peptidoglycan)	Irreversible
RAB5_DEPH	Rab5 dephosphorylation	Irreversible
RAB5_PHOS	Rab5 phosphorylation	Irreversible
RAB5_RBPTIN5_RBX5_BIND	Rab5-Rabaptin5/Rabex5 binding	Irreversible
RAC1_DEPH	Rac1 dephosphorylation	Irreversible
RAC1_PHOS	Rac1 phosphorylation	Irreversible
RAC1_VAV1_BIND	Rac1-Vav1 binding	Irreversible
RAF1_DEPH1	PP2A-mediated Raf-1 dephosphorylation	Irreversible
RAF1_DEPH2	Unknown phosphatase-mediated Raf-1 dephosphorylation	Irreversible
RAS_DEPH	Ras dephosphorylation	Irreversible
RAS_GTP_RAF1_BIND	Ras/GTP-Raf1 binding	Irreversible
RAS_GTP_RIN1_BIND	Ras/GTP-Rin1 binding	Irreversible
RAS_PHOS	Ras phosphorylation	Irreversible
RBPTIN5_RBX5_BIND	Rabaptin5-Rabex5 binding	Irreversible
RHOA_DEPH	RhoA dephosphorylation	Irreversible
RHOA_PHOS	RhoA phosphorylation	Irreversible
RIP1_TICAM1P_BIND	RIP1-TICAM1P-TRAF6 (dimer) binding	Irreversible
RIP2_NOD1P_BIND	RIP2-NOD1P binding	Irreversible
RIP2_NOD2P_BIND	RIP2-NOD2P binding	Irreversible
RIP2_TRIP6_NOD1P_BIND	RIP2-TRIP6-NOD1P binding	Irreversible
RIP2_TRIP6_TRAF2_BIND	RIP2-TRIP6-TRAF2 binding	Irreversible
RKIP_MAP3K14_BIND	RKIP-MAP3K14 binding	Irreversible
RKIP_MAP3K7IP_TRAF6_BIND	RKIP-MAP3K7/IP/Ubc13/Uev1A/TRAF6 (dimer) binding	Irreversible
SF_TLRL1/2	Toll-like receptor 1/2 ligand (Soluble factors)	Irreversible
SGK_MAP3K3	SGK-mediated MAP3K3 phosphorylation	Irreversible
SGK_PHOS	SGK autophosphorylation	Irreversible
SIGIRR_DIME	SIGIRR dimerization	Irreversible
SQSTM1_PKCZ_BIND	Sequestosome 1 (p62)-PKCzeta binding	Irreversible
SRCK_BTK	Src family tyrosine kinase-mediated BTK phosphorylation	Irreversible
SRCK_CBL	Src kinase-mediated c-Cbl phosphorylation	Irreversible
SRCK_PHOS	Src kinase autophosphorylation	Irreversible
SRTK_PHOS	Src-related tyrosine kinase autophosphorylation	Irreversible
SRTK_VAV1	Src-related tyrosine kinase-mediated Vav1 phosphorylation	Irreversible
SSRNA_TLRL7	Toll-like receptor 7 ligand (Single stranded RNA)	Irreversible
SSRNA_TLRL8	Toll-like receptor 8 ligand (Single stranded RNA)	Irreversible
ST2L_TIRAP_BIND	ST2L-TIRAP-MyD88 binding	Irreversible
STF_TLRL2/6	Toll-like receptor 2/6 ligand (Small molecule transcription factors)	Irreversible
STLR2/L_SCD14_BIND	sTLR2-ligand-sCD14 binding	Irreversible
STLR2_CD14_BIND	sTLR2-CD14 binding	Irreversible
STLR4_BIND	soluble TLR4/MD2-ligand binding	Irreversible
STLR4_MD2_BIND	sTLR4-MD2 binding	Irreversible
T3RFBN_TLRL4	Toll-like receptor 4 ligand (Type III repeat extra domain A of fibronectin)	Irreversible
TBK1_IRF3	TBK1-mediated IRF3 phosphorylation	Irreversible
TBK1_NFKB	TBK1-mediated NF-kB (p50/p65) phosphorylation	Irreversible
TBK1_NFKB_P5K	NF-kB P5 kinase (TBK1)	Irreversible
TBK1_PHOS	TBK1 autophosphorylation	Irreversible
TCLDLPP_TLRL1/2	Toll-like receptor 1/2 ligand (Triacetylated lipopeptides)	Irreversible
TICAM1P_IKKE_BIND	TICAM1P-IKKE binding	Irreversible
TICAM1P_TBK1_BIND	TICAM1P-TBK1 binding	Irreversible
TICAM2_TICAM1	TICAM2/TLR4/L/MD2-mediated activation of TICAM1	Irreversible
TICAM2_TLR4/L_MD2_BIND	TICAM2-TLR4/L/MD2 binding	Irreversible
TIR_MYD_BIND	TLR9-mediated TIR-MyD88 binding	Irreversible

TIR_MYD_BIND2	TLR1/10-mediated TIR-MyD88 binding	Irreversible
TIR_MYD_BIND3	TLR2/10-mediated TIR-MyD88 binding	Irreversible
TIR_MYD_BIND4	TLR10-mediated TIR-MyD88 binding	Irreversible
TIR_MYD_BIND5	TLR11-mediated TIR-MyD88 binding	Irreversible
TIR_MYD_BIND6	IL1R1-mediated TIR-MyD88 binding	Irreversible
TIR_MYD_BIND7	TLR5-mediated TIR-MyD88 binding	Irreversible
TIR_MYD_BIND8	TLR8-mediated TIR-MyD88 binding	Irreversible
TIR_MYD_BIND9	TLR7-mediated TIR-MyD88 binding	Irreversible
TIR_MYD_FADD_BIND	TIR/MyD88 (dimer)/FADD complex	Irreversible
TIR_MYD_IRAK4_BIND	TIR/MyD88-IRAK4 binding	Irreversible
TIR_MYD_TOLLIP_BIND	TIR/TIRAP/MyD88/IRAK-IRAK1C/TOLLIP binding	Irreversible
TIR_TIRAP_BIND	TLR1/2-mediated TIR domain TIRAP binding	Irreversible
TIR_TIRAP_BIND2	TLR2/6-mediated TIR domain TIRAP binding	Irreversible
TIR_TIRAP_BIND3	TLR2-mediated TIR domain TIRAP binding	Irreversible
TIR_TIRAP_BIND4	TLR4-mediated TIR domain TIRAP binding	Irreversible
TIR_TIRAP_IRAK2_BIND	TIR/TIRAP/MyD88/IRAK4-IRAK2 binding	Irreversible
TIR_TIRAP_IRAK4_BIND	TIR/TIRAP/MyD88-IRAK4 binding	Irreversible
TIR_TIRAP_MYD_BIND	TIR/TIRAP-MyD88 binding	Irreversible
TIR_TIRAP_MYD_FADD_BIND	TIR/TIRAP/MyD88 (dimer)/FADD complex	Irreversible
TIR_TIRAP_TOLLIP_2PHOS	TIR/TIRAP/MyD88/IRAK1/IRAK4/TOLLIP diphosphorylation	Irreversible
TIR_TIRAP_TOLLIP_BIND	TIR/TIRAP/MyD88/IRAK4-IRAK1/TOLLIP binding	Irreversible
TIR_TIRAP_TOLLIP_PHOS	TIR/TIRAP/MyD88/IRAK1/IRAK4/TOLLIP phosphorylation	Irreversible
TLR1/10_BIND	Toll-like receptor 1/10 ligand binding	Irreversible
TLR1/2_BIND	Toll-like receptor 1/2 ligand binding	Irreversible
TLR10_BIND	Toll like receptor 10 ligand binding	Irreversible
TLR11_BIND	Toll like receptor 11 ligand binding	Irreversible
TLR2/10_BIND	Toll-like receptor 2/10 ligand binding	Irreversible
TLR2/6_BIND	Toll-like receptor 2/6 ligand binding	Irreversible
TLR2/L-D_BIND	TLR2-ligand-CD14 binding	Irreversible
TLR2_2PHOS	TLR2 autophosphorylation	Irreversible
TLR2_PI3K1A	TLR2-mediated PI3K class 1A phosphorylation	Irreversible
TLR3_2PHOS	TLR3 diphosphorylation	Irreversible
TLR3_BIND	Toll like receptor 3 ligand binding	Irreversible
TLR3_PI3K1A	TLR3-mediated PI3K class 1A phosphorylation	Irreversible
TLR3_TICAM1	TLR3-mediated TICAM1 activation	Irreversible
TLR4/L_MD2_BIND	TLR4/MD2-ligand-LBP binding	Irreversible
TLR4/L_MD2_TRD3A_BIND	TLR4/L/MD2-TRIAD3A binding	Irreversible
TLR4/L_MD2_TRD3A_DEGR	TLR4/L/MD2/TRIAD3A degradation	Irreversible
TLR4_MD2_BIND	TLR4-MD2 binding	Irreversible
TLR4_SIGIRR_BIND	TLR4-SIGIRR binding	Irreversible
TLR4_SIGIRR_TRAF6_BIND	TLR4/SIGIRR-TRAF6 (dimer) binding	Irreversible
TLR5_BIND	Toll-like receptor 5 ligand binding	Irreversible
TLR5_SIGIRR_BIND	TLR5-SIGIRR binding	Irreversible
TLR5_SIGIRR_TRAF6_BIND	TLR5/SIGIRR-TRAF6 (dimer) binding	Irreversible
TLR7_BIND	Toll-like receptor 7 ligand binding	Irreversible
TLR8_BIND	Toll-like receptor 8 ligand binding	Irreversible
TLR9/L_TRD3A_BIND	TLR9/L-TRIAD3A binding	Irreversible
TLR9/L_TRD3A_DEGR	TLR9/L/TRIAD3A degradation	Irreversible
TLR9_BIND	Toll-like receptor 9 ligand binding	Irreversible
TLR9_SIGIRR_BIND	TLR9-SIGIRR binding	Irreversible
TLR9_SIGIRR_TRAF6_BIND	TLR9/SIGIRR-TRAF6 (dimer) binding	Irreversible
TLR9_TRAN	TLR9 transport	Irreversible
TLRL9_TRAN	TLR9 ligand (generic) transport	Irreversible
TOLLIP_DEGR	Toll interacting protein degradation	Irreversible
TOLLIP_DEGR2	Toll interacting protein degradation 2	Irreversible
TOLLIP_PHOS	Toll interacting protein phosphorylation	Irreversible
TOLLIP_UBIQ	Toll interacting protein (phosphorylated) ubiquitination	Irreversible
TOLLIP_pail345p_hs_BIND	TOLLIP-PI(3,4,5)P3 binding	Irreversible

TOLLIP_pail3p_hs_BIND	TOLLIP-PI(3)P binding	Irreversible
TRAF6_DIME	Tumor necrosis factor receptor associated factor 6 dimerization	Irreversible
TRAF6_SIGIRR_BIND	TRAF6 (dimer)-SIGIRR (dimer) binding	Irreversible
TRX_NADPH_BIND	thioredoxin-thioredoxin reductase-NADPH binding	Irreversible
TRX_OXID	thioredoxin oxidation	Irreversible
TRX_REDU	thioredoxin reduction	Irreversible
TXL_TLRL4	Toll-like receptor 4 ligand (Taxol)	Irreversible
UBIQ_TRAN	ubiquitin transport, cytoplasm	Reversible
UBIQ_TRAN2	ubiquitin transport, nucleus	Reversible
UMLCPGD_TLRL9	Toll-like receptor 9 ligand (Unmethylated CpG DNA)	Irreversible
UNKN_TLRL11	Toll-like receptor 11 ligand (Unknown)	Irreversible
ZMS_TLRL2	Toll-like receptor 2 ligand (Zymosan)	Irreversible
ZMS_TLRL2/6	Toll-like receptor 2/6 ligand (Zymosan)	Irreversible
adp_exch	adp_exch	Reversible
atp_exch	atp_exch	Reversible
ca2_TRAN	calcium ion transport	Irreversible
ca2_TRAN2	calcium ion transport 2	Irreversible
gdp_exch	gdp transport	Reversible
gtp_exch	gtp transport	Reversible
pa_hs_DEPH	phosphatidic acid dephosphorylatoin	Irreversible
pail45p_hs_DISS	PI(4,5)P2 hydrolysis	Irreversible
pail45p_hs_DISS2	PI(4,5)P2 hydrolysis 2	Irreversible
pail45p_hs_PLCB2_BIND	PI(4,5)P2-phospholipase C beta II binding	Irreversible
pail45p_hs_PLCG_BIND	PI(4,5)P2-phospholipase C gamma binding	Irreversible
pail_hs_PHOS	PI3K3-mediated pail_hs phosphorylation	Irreversible
pchol_hs_DISS	phosphatidylcholine hydrolysis	Irreversible
pchol_hs_PLDP_BIND	phosphatidylcholine-phospholipase D (activated) binding	Irreversible
