# Supplementary material for: Identification of Potential Pathway Mediation Targets in Toll-like Receptor Signaling
Source: PLoS Comput Biol. 2009 Feb 20;5(2):e1000292. doi: 10.1371/journal.pcbi.1000292 (PMC2634968; doi:10.1371/journal.pcbi.1000292)
Supplement: Table S3 — TLR network inputs (0.02 MB PDF) [file pcbi.1000292.s005.pdf]

Table S3: TLR network inputs

Receptor	Ligand abbreviation	Ligand name
IL1	PRE_IL1A	pre IL-1A
IL1	PRE_IL1B	pre IL-1B
NOD1	26dap-LL	diaminopimelic acid
NOD2	MRDP	muramyl dipeptide
TLR1	SF	soluble factors
TLR1	TCLDLPP	triacetylated lipoproteins
TLR1	TLRL1/10	TLR1/10 ligand
TLR2	ALPS	atypical lipopolysaccharide
TLR2	CSGA	CsgA
TLR2	DCLDLPP	diacetylated lipopeptides
TLR2	DCLLPP	diacyl lipopeptides
TLR2	GCSPL	glycoinositol phospholipids
TLR2	GLC	glycolipids
TLR2	HSP70	heat shock protein (70kDa)
TLR2	LAM	lipoarabinomannan
TLR2	LP	lipoprotein
TLR2	LPPS	lipopeptides
TLR2	LPS_HS	lipopolysaccharide (Homo sapiens)
TLR2	LTA	lipoteichoic acid
TLR2	MRAP	mannuronic acid polymer
TLR2	OMPA	outer membrane protein A
TLR2	OSPALP	outer surface protein A
TLR2	PRNS	porins
TLR2	PSM	phenol-soluble modulin
TLR2	PTG_HS	peptidoglycan (Homo sapiens)
TLR2	SF	soluble factors
TLR2	STF	soluble tuberculosis factor
TLR2	TCLDLPP	triacetylated lipoproteins
TLR2	TLRL2/10	TLR2/10 ligand
TLR2	ZMS	zymosan
TLR3	DSRNA	double stranded RNA
TLR3	MRNA	mRNA
TLR4	BDFN2	beta defensin 2
TLR4	ENVP	envelope protein
TLR4	FBNG	fibrinogen
TLR4	FUSP	fusion protein
TLR4	HSP60	heat shock protein (60kDa)
TLR4	HSP70	heat shock protein (70kDa)
TLR4	LPS_HS	lipopolysaccharide (Homo sapiens)
TLR4	MRAP	mannuronic acid polymer
TLR4	OLSCHYA	oligosaccharides of hyaluronic acid
TLR4	PSCHPS	polysaccharide fragment of heparan sulphate
TLR4	T3RFBN	type III repeat extra domain A of fibronectin
TLR4	TXL	taxol
TLR5	FLGN	flagellin
TLR6	DCLDLPP	diacetylated lipopeptides
TLR6	DCLLPP	diacyl lipopeptides
TLR6	LTA	lipoteichoic acid
TLR6	OSPALP	outer surface protein A
TLR6	PSM	phenol-soluble modulin
TLR6	STF	soluble tuberculosis factor
TLR6	ZMS	zymosan
TLR7	BPM	bropirimine
TLR7	IMQ	imidazoquinoline

TLR7	LXR	loxoribine
TLR7	SSRNA	single stranded RNA
TLR8	IMQ	imidazoquinoline
TLR8	SSRNA	single stranded RNA
TLR9	CPGCIGC	CpG chromatic IgG2a complexs
TLR9	UMLCPGD	unmethylated CpG DNA
TLR10	TLRL10	TLR10 ligand
TLR11	PLP	profilin-like protein
TLR11	UNKN	unknown TLR11 ligand
