# Supplementary material for: Identification of Potential Pathway Mediation Targets in Toll-like Receptor Signaling
Source: PLoS Comput Biol. 2009 Feb 20;5(2):e1000292. doi: 10.1371/journal.pcbi.1000292 (PMC2634968; doi:10.1371/journal.pcbi.1000292)
Supplement: Table S4 — TLR network outputs (0.01 MB PDF) [file pcbi.1000292.s006.pdf]

Table S4: TLR network outputs

Output	Associated Reactions	Reaction Names
ROS production	DM_PHOX_GTP-3P(v)	gp91/p22/p40/p47 (3 phosphorylated)/p67PHOX/Rac1/GTP complex demand
ROS production	DM_PHOX_GTP-8P(v)	gp91/p22/p40/p47 (8 phosphorylated)/p67PHOX/Rac1/GTP complex demand
IRF3	DM_ISRE_IRF3(n)	ISRE/IRF3 complex (2 phosphorylated) demand
IRF7	DM_ISRE_IRF7(n)	ISRE/IRF7 complex (2 phosphorylated) demand
CRE	CREB_CRE_BIND	CREB-CRE site binding
AP-1	AP1_FOS_JUN_BIND	AP-1 site-cFos-cJun binding
AP-1	AP1_JUN_BIND	AP-1 site-cJun binding
NF-kappa-B	NFKB_IKBA_DISS	NFkB (p50/p65)-IkBa dissociation
NF-kappa-B	NFKB_IKBB_DISS	NFkB (p50/p65)-IkBb dissociation
