# Supplementary material for: Identification of Potential Pathway Mediation Targets in Toll-like Receptor Signaling
Source: PLoS Comput Biol. 2009 Feb 20;5(2):e1000292. doi: 10.1371/journal.pcbi.1000292 (PMC2634968; doi:10.1371/journal.pcbi.1000292)
Supplement: Table S5 — Intermediate reactions (0.01 MB PDF) [file pcbi.1000292.s007.pdf]

Table S5: Intermediate Reactions

Pathway	Intermediate Reaction	Reaction Name
IRF3	DM_ISRE_IRF3(n)	ISRE/IRF3 complex (2 phosphorylated) demand
IRF7	DM_ISRE_IRF7(n)	ISRE/IRF7 complex (2 phosphorylated) demand
ROS production	DM_PHOX_GTP-3P(v)	gp91/p22/p40/p47 (3 phosphorylated)/p67PHOX/Rac1/GTP complex demand
IL-1	AJUBA_CPX_IKK	PKCzeta-mediated IKK phosphorylation
MyD88	IRAK1_TIFA_3UBIQ	IRAK1/TIFA/TRAF6 (dimer)/Ubc13/Uev1A triubiquitination
RIP1	IKK_RIP1_TICAM1P_BIND	IKK-RIP1/TICAM1P binding
NOD1	IKK_RIP2_NOD1P_BIND	IKK-RIP2/NOD1P binding
NOD2	IKK_RIP2_NOD2P_BIND	IKK-RIP2/NOD2P binding
RIP2/TRIP6/TRAF2	IKK_RIP2_TRIP6_TRAF2_BIND	IKK-RIP2/TRIP6/TRAF2 binding
PI3K	PI3K1A_pail45p_hs	PI3K class 1A mediated PI(4,5)P2 phosphorylation
