# Supplementary material for: Identification of Potential Pathway Mediation Targets in Toll-like Receptor Signaling
Source: PLoS Comput Biol. 2009 Feb 20;5(2):e1000292. doi: 10.1371/journal.pcbi.1000292 (PMC2634968; doi:10.1371/journal.pcbi.1000292)
Supplement: Table S6 — Critical network reactions (0.01 MB PDF) [file pcbi.1000292.s008.pdf]

Table S6: Critical Network Reactions

Note: Critical network reactions are bolded.

Pathway	IRF3	IRF7	ROS production	IL-1	RIP1
**Essential network reactions**	IRF3_DIME	IRF7_ISRE_BIND	**GP91_P22PHOX_BIND**	**AJUBA_CPX_BIND**	IKK_RIP1_TICAM1P_BIND
	IRF3_ISRE	IRF7_TRAN	PHOX_GTP_BIND2	AJUBA_CPX_IKK	IKK_RIP1_TICAM1P_PHOS
	IRF3_TRA	TRAF6_DIME	Plt	**AJUBA_CPX_PHOS**	RIP1_TICAM1P_BIND
		UBIQ_TRAN	**RAC1_PHOS**	IL1/L_IL1RAP_BIND	TRAF6_DIME
			**RAC1_VAV1_BIND**	IL1R1_BIND	
				**SQSTM1_PKCZ_BIND**	

Pathway	NOD1	NOD2	RIP2/TRIP6/TRAF2	MYD88	PI3K
**Essential network reactions**	IKK_RIP2_	IKK_RIP2_NOD2P_	IKK_RIP2_TRIP6_TRAF2	**IRAK1_TIFA_3UBIQ**	PAIL4P_HSt
	IKK_RIP2_	IKK_RIP2_NOD2P_	IKK_RIP2_TRIP6_TRAF2	**MYD88_DIME**	PI3K1A_pail45p_hs
	NOD1P_BI	NOD2P_BIND	RIP2_TRIP6_TRAF2_BIN	UBIQ_TRAN	PI4P5K_PHOS
	RIP2_NOD	RIP2_NOD2P_BIND	TLR2/L-D_BIND		PI4P5K_pail4p_hs
