# Supplementary material for: Identification of Potential Pathway Mediation Targets in Toll-like Receptor Signaling
Source: PLoS Comput Biol. 2009 Feb 20;5(2):e1000292. doi: 10.1371/journal.pcbi.1000292 (PMC2634968; doi:10.1371/journal.pcbi.1000292)
Supplement: Table S7 — Reaction references (0.36 MB PDF) [file pcbi.1000292.s009.pdf]

Table S7: Reaction References

Reaction Abbreviation	Citation (Title, authors, journal, PMID, publication year, publication month, publication day)
14-3-3_KSR1_BIND	C-TAK1 regulates Ras signaling by phosphorylating the MAPK scaffold, KSR1.,Müller J, Ory S, Copeland T, Piwnica-Worms H, Morrison DK.,Mol Cell.,11741534,2001,11
14-3-3_RAF1_BIND	Phosphorylation and regulation of Raf by Akt (protein kinase B).,Zimmermann S, Moelling K.,Science.,10576742,1999,11,26
14-3-3_RAF1_BIND	Untying the regulation of the Raf-1 kinase.,Dhillon AS, Kolch W.,Arch Biochem Biophys.,12127063,2002,8,1
ABIN_A20_BIND	Structure-function analysis of the A20-binding inhibitor of NF-kappa B activation, ABIN-1.,Heyninck K, Kreike MM, Beyaert R.,FEBS Lett.,12586352,2003,2,11
ABIN_A20_BIND	The zinc finger protein A20 inhibits TNF-induced NF-kappaB-dependent gene expression by interfering with an RIP- or TRAF2-mediated transactivation signal and directly binds to a novel NF-kappaB-inhibiting protein ABIN.,Heyninck K, De Valck D, Vanden Berghe W, Van Criekinge W, Contreras R, Fiers W, Haegeman G, Beyaert R.,J Cell Biol.,10385526,1999,6,28
AJUBA_CPX_BIND	The LIM protein Ajuba influences interleukin-1-induced NF-kappaB activation by affecting the assembly and activity of the protein kinase Czeta/p62/TRAF6 signaling complex.,Feng Y, Longmore GD.,Mol Cell Biol.,15870274,2005,5
AJUBA_CPX_BIND	The atypical PKC-interacting protein p62 channels NF-kappaB activation by the IL-1-TRAF6 pathway.,Sanz L, Diaz-Meco MT, Nakano H, Moscat J.,EMBO J.,10747026,2000,4,3
AJUBA_CPX_IKK	Activation of IkappaB kinase beta by protein kinase C isoforms.,Lallena MJ, Diaz-Meco MT, Bren G, Payá CV, Moscat J.,Mol Cell Biol.,10022904,1999,3
AJUBA_CPX_PHOS	Insulin and PIP3 activate PKC-zeta by mechanisms that are both dependent and independent of phosphorylation of activation loop (T410) and autophosphorylation (T560) sites.,Standaert ML, Bandyopadhyay G, Kanoh Y, Sajan MP, Farese RV.,Biochemistry,11141077,2001,1,9
AJUBA_CPX_PHOS	The LIM protein Ajuba influences interleukin-1-induced NF-kappaB activation by affecting the assembly and activity of the protein kinase Czeta/p62/TRAF6 signaling complex.,Feng Y, Longmore GD.,Mol Cell Biol.,15870274,2005,5
AKT_GSK3B	Inhibition of glycogen synthase kinase-3 by insulin mediated by protein kinase B.,Cross DA, Alessi DR, Cohen P, Andjelkovich M, Hemmings BA.,Nature.,8524413,1998,12,21
AKT_GSK3B	Toll-like receptor-mediated cytokine production is differentially regulated by glycogen synthase kinase 3.,Martin M, Rehani K, Jope RS, Michalek SM.,Nat Immunol.,16007092,2005,8
AKT_IKK	NF-kappaB activation by tumour necrosis factor requires the Akt serine-threonine kinase.,Ozes ON, Mayo LD, Gustin JA, Pfeffer SR, Pfeffer LM, Donner DB.,Nature,10485710,1999,9,2
AKT_IKK	Production of matrix metalloproteinase-9 by activated human monocytes involves a phosphatidylinositol-3 kinase/Akt/IKKalpha/NF-kappaB pathway.,Lu Y, Wahl LM.,J Leukoc Biol.,15800029,2005,7
AKT_MAP3K3	Akt down-regulation of p38 signaling provides a novel mechanism of vascular endothelial growth factor-mediated cytoprotection in endothelial cells.,Gratton JP, Morales-Ruiz M, Kureishi Y, Fulton D, Walsh K, Sessa WC.,J Biol Chem.,11387313,2001,8,10
AKT_MAP3K3	PI3K and negative regulation of TLR signaling.,Fukao T, Koyasu S.,Trends Immunol.,12860525,2003,7
AKT_MAP3K5	Akt phosphorylates and negatively regulates apoptosis signal-regulating kinase 1.,Kim AH, Khursigara G, Sun X, Franke TF, Chao MV.,Mol Cell Biol.,11154276,2001,2

AKT_MAP3K5	PI3K and negative regulation of TLR signaling.,Fukao T, Koyasu S.,Trends Immunol.,12860525,2003,7
AKT_PHOS	Unravelling the activation mechanisms of protein kinase B/Akt.,Scheid MP, Woodgett JR.,FEBS Lett.,12829245,2003,7,3
AKT_RAF1	Phosphorylation and regulation of Raf by Akt (protein kinase B).,Zimmermann S, Moelling K.,Science.,10576742,1999,11,26
AKT_RAF1	Untying the regulation of the Raf-1 kinase.,Dhillon AS, Kolch W.,Arch Biochem Biophys.,12127063,2002,8,1
AKT_TRAN	Translocation of Akt/PKB to the nucleus of osteoblast-like MC3T3-E1 cells exposed to proliferative growth factors.,Borgatti P, Martelli AM, Bellacosa A, Casto R, Massari L, Capitani S, Neri LM.,FEBS Lett.,10899305,2000,7,14
AKT_pail345p_hs_BIND	Unravelling the activation mechanisms of protein kinase B/Akt.,Scheid MP, Woodgett JR.,FEBS Lett.,12829245,2003,7,3
AKT_pail345p_hs_DISS	Unravelling the activation mechanisms of protein kinase B/Akt.,Scheid MP, Woodgett JR.,FEBS Lett.,12829245,2003,7,3
AP1_FOS_JUN_BIND	AP-1 function and regulation,Karin M, Liu Z, Zandi E.,Curr Opin Cell Biol.,9069263,1997,4
AP1_JUN_BIND	AP-1 function and regulation,Karin M, Liu Z, Zandi E.,Curr Opin Cell Biol.,9069263,1997,4
BTK_2PHOS	Bruton's tyrosine kinase (Btk)-the critical tyrosine kinase in LPS signalling?,Jefferies CA, O'Neill LA.,Immunol Lett.,15081522,2004,3,29
BTK_CBL_BIND	Bruton's tyrosine kinase (Btk)-the critical tyrosine kinase in LPS signalling?,Jefferies CA, O'Neill LA.,Immunol Lett.,15081522,2004,3,29
BTK_CBL_BIND	Cbl-b negatively regulates B cell antigen receptor signaling in mature B cells through ubiquitination of the tyrosine kinase Syk.,Sohn HW, Gu H, Pierce SK.,J Exp Med.,12771181,2003,6,2
BTK_CBL_BIND	The protein product of the c-cbl protooncogene is phosphorylated after B cell receptor stimulation and binds the SH3 domain of Bruton's tyrosine kinase.,Cory GO, Lovering RC, Hinshelwood S, MacCarthy-Morrogh L, Levinsky RJ, Kinnon C.,J Exp Med.,7629518,1995,8,1
BTK_IBTK_BIND	Direct inhibition of Bruton's tyrosine kinase by IBtk, a Btk-binding protein.,Liu W, Quinto I, Chen X, Palmieri C, Rabin RL, Schwartz OM, Nelson DL, Scala G.,Nat Immunol.,11577348,2001,10
BTK_PLCG	Bruton's tyrosine kinase (Btk)-the critical tyrosine kinase in LPS signalling?,Jefferies CA, O'Neill LA.,Immunol Lett.,15081522,2004,3,29
BTK_PLCG	Phospholipase D: a lipid centric review.,Jenkins GM, Frohman MA.,Cell Mol Life Sci.,16143829,2005,10
BTK_SH3BP5_BIND	Bruton's tyrosine kinase activity is negatively regulated by Sab, the Btk-SH3 domain-binding protein.,Yamadori T, Baba Y, Matsushita M, Hashimoto S, Kurosaki M, Kurosaki T, Kishimoto T, Tsukada S.,Proc Natl Acad Sci USA.,10339589,1999,5,25
BTK_UBIQ	Bruton's tyrosine kinase (Btk)-the critical tyrosine kinase in LPS signalling?,Jefferies CA, O'Neill LA.,Immunol Lett.,15081522,2004,3,29
BTK_UBIQ	Cbl-b negatively regulates B cell antigen receptor signaling in mature B cells through ubiquitination of the tyrosine kinase Syk.,Sohn HW, Gu H, Pierce SK.,J Exp Med.,12771181,2003,6,2
BTK_UBIQ	The protein product of the c-cbl protooncogene is phosphorylated after B cell receptor stimulation and binds the SH3 domain of Bruton's tyrosine kinase.,Cory GO, Lovering RC, Hinshelwood S, MacCarthy-Morrogh L, Levinsky RJ, Kinnon C.,J Exp Med.,7629518,1995,8,1
BTK_VAV1	Bruton's Tyrosine Kinase is involved in innate and adaptive immunity.,Brunner C, Müller B, Wirth T.,Histol Histopathol.,15944945,2005,7
BTK_VAV1	Bruton's tyrosine kinase (Btk)-the critical tyrosine kinase in LPS signalling?,Jefferies CA, O'Neill LA.,Immunol Lett.,15081522,2004,3,29

BTK_pail345p_hs_BIND	Bruton's tyrosine kinase (Btk)-the critical tyrosine kinase in LPS signalling?,Jefferies CA, O'Neill LA.,Immunol Lett.,15081522,2004,3,29
CALPN_3ca2_BIND	Calcium-activated neutral protease (calpain) system: structure, function, and regulation.,Croall DE, DeMartino GN.,Physiol Rev.,2057527,1991,7
CALPN_3ca2_BIND	Differential sensitivity of interleukin-1 alpha and -beta precursor proteins to cleavage by calpain, a calcium-dependent protease.,Kavita U, Mizel SB.,J Biol Chem.,7499244,1995,11,17
CAMK2_SERCA	Identification of Ser38 as the site in cardiac sarcoplasmic reticulum Ca(2+)-ATPase that is phosphorylated by Ca2+/calmodulin-dependent protein kinase.,Toyofuku T, Curotto Kurzydlowski K, Narayanan N, MacLennan DH.,J Biol Chem.,7929371,1994,10,21
CAM_3ca2_BIND	Calcium/calmodulin-dependent protein kinase II.,Colbran RJ, Schworer CM, Hashimoto Y, Fong YL, Rich DP, Smith MK, Soderling TR.,Biochem J.,2539803,1989,3,1
CAM_3ca2_BIND	Interactive properties of calmodulin.,Cox JA.,Biochem J.,3281652,1988,2,1
CAM_CAMK2_BIND	Calcium/calmodulin-dependent protein kinase II.,Colbran RJ, Schworer CM, Hashimoto Y, Fong YL, Rich DP, Smith MK, Soderling TR.,Biochem J.,2539803,1989,3,1
CAM_CAMK2_CREB	Calmodulin kinase II attenuation of gene transcription by preventing cAMP response element-binding protein (CREB) dimerization and binding of the CREB-binding protein.,Wu X, McMurray CT.,J Biol Chem.,11013247,2001,1,19
CAM_CAMK2_CREB-2	Calmodulin kinase II attenuation of gene transcription by preventing cAMP response element-binding protein (CREB) dimerization and binding of the CREB-binding protein.,Wu X, McMurray CT.,J Biol Chem.,11013247,2001,1,19
CAM_CAMK2_IKK	Calmodulin-dependent kinase II mediates T cell receptor/CD3- and phorbol ester-induced activation of IkappaB kinase.,Hughes K, Edin S, Antonsson A, Grundström T.,J Biol Chem.,11470799,2001,9,21
CAM_CAMK2_PHOS	Regulation of the activities of multifunctional Ca2+/calmodulin-dependent protein kinases.,Fujisawa H.,J Biochem (Tokyo).,11173518,2001,2
CDC42_DEPH	Rho GTPases and their effector proteins.,Bishop AL, Hall A.,Biochem J.,10816416,2000,6,1
CDC42_PHOS	Rho GTPases and their effector proteins.,Bishop AL, Hall A.,Biochem J.,10816416,2000,6,1
CDC42_PHOS	VAV proteins as signal integrators for multi-subunit immune-recognition receptors.,Turner M, Billadeau DD.,Nat Rev Immunol.,12094222,2002,7
CDC42_VAV1_BIND	Rho GTPases and their effector proteins.,Bishop AL, Hall A.,Biochem J.,10816416,2000,6,1
CDC42_VAV1_BIND	VAV proteins as signal integrators for multi-subunit immune-recognition receptors.,Turner M, Billadeau DD.,Nat Rev Immunol.,12094222,2002,7
CK2_IKBB	Basal phosphorylation of the PEST domain in the I(kappa)B(beta) regulates its functional interaction with the c-rel proto-oncogene product.,Chu ZL, McKinsey TA, Liu L, Qi X, Ballard DW.,Mol Cell Biol.,8887627,1996,9
CK2_IKBB	Distinct functional properties of IkappaB alpha and IkappaB beta.,Tran K, Merika M, Thanos D.,Mol Cell Biol.,9271416,1997,9
CK2_NFKB	Post-translational modifications regulating the activity and function of the nuclear factor kappa B pathway.,Perkins ND.,Oncogene.,17072324,2006,10,30
CK2_NFKB	Tumor necrosis factor alpha-induced phosphorylation of RelA/p65 on Ser529 is controlled by casein kinase II.,Wang D, Westerheide SD, Hanson JL, Baldwin AS. ,J Biol Chem.,10938077,2000,10,20
CK2_NFKB_P4K	Post-translational modifications regulating the activity and function of the nuclear factor kappa B pathway.,Perkins ND.,Oncogene.,17072324,2006,10,30
CK2_NFKB_P4K	Tumor necrosis factor alpha-induced phosphorylation of RelA/p65 on Ser529 is controlled by casein kinase II.,Wang D, Westerheide SD, Hanson JL, Baldwin AS. ,J Biol Chem.,10938077,2000,10,20

CREB_CBP_BIND	Phosphorylated CREB binds specifically to the nuclear protein CBP.,Chrivia JC, Kwok RP, Lamb N, Hagiwara M, Montminy MR, Goodman RH.,Nature,8413673,1993,10,28
CREB_CBP_DISS	CREB DNA binding activity is inhibited by glycogen synthase kinase-3 beta and facilitated by lithium.,Grimes CA, Jope RS.,J Neurochem.,11579131,2001,9
CREB_CBP_DISS2	Calmodulin kinase II attenuation of gene transcription by preventing cAMP response element-binding protein (CREB) dimerization and binding of the CREB-binding protein.,Wu X, McMurray CT.,J Biol Chem.,11013247,2001,1,19
CREB_DIME	Cyclic adenosine 3',5'-monophosphate response element binding protein (CREB) and related transcription-activating deoxyribonucleic acid-binding proteins.,Meyer TE, Habener JF.,Endocr Rev.,8319595,1993,6
CTAK1_KSR1	C-TAK1 regulates Ras signaling by phosphorylating the MAPK scaffold, KSR1.,Müller J, Ory S, Copeland T, Piwnica-Worms H, Morrison DK.,Mol Cell.,11741534,2001,11
CTAK1_PHOS	C-TAK1 regulates Ras signaling by phosphorylating the MAPK scaffold, KSR1.,Müller J, Ory S, Copeland T, Piwnica-Worms H, Morrison DK.,Mol Cell.,11741534,2001,11
DAGK_PHOS	Characterization of a Rac1- and RhoGDI-associated lipid kinase signaling complex.,Tolias KF, Couvillon AD, Cantley LC, Carpenter CL.,Mol Cell Biol.,9447972,1998,2
DAGK_PHOS	Molecules in focus: diacylglycerol kinase.,Sakane F, Kanoh H.,Int J Biochem Cell Biol.,9438377,1997,10
DAGK_dag_hs	Mammalian diacylglycerol kinases, a family of lipid kinases with signaling functions.,Topham MK, Prescott SM.,J Biol Chem.,10206945,1999,4,23
EEA1_DIME	The endosome fusion regulator early-endosomal autoantigen 1 (EEA1) is a dimer.,Callaghan J, Simonsen A, Gaullier JM, Toh BH, Stenmark H.,Biochem J.,10024533,1999,3,1
EEA1_pail3p_hs_BIND	Signal transduction pathways mediated by the interaction of CpG DNA with Toll-like receptor 9.,Takeshita F, Gursel I, Ishii KJ, Suzuki K, Gursel M, Klinman DM.,Semin Immunol.,14751759,2004,2
ELK1_DEPH	Stimulation of Elk1 transcriptional activity by mitogen-activated protein kinases is negatively regulated by protein phosphatase 2B (calcineurin).,Tian J, Karin M.,J Biol Chem.,10329725,1999,5,21
ELK1_SRE_BIND	The SRF accessory protein Elk-1 contains a growth factor-regulated transcriptional activation domain.,Marais R, Wynne J, Treisman R.,Cell,8386592,1993,4,23
FADD_PCASP8_BIND	Membrane oligomerization and cleavage activates the caspase-8 (FLICE/MACHalpha1) death signal.,Martin DA, Siegel RM, Zheng L, Lenardo MJ.,J Biol Chem.,9468483,1998,2,20
FADD_PCASP8_BIND	The apoptotic signaling pathway activated by Toll-like receptor-2.,Aliprantis AO, Yang RB, Weiss DS, Godowski P, Zychlinsky A.,EMBO J.,10880445,2000,7,3
FADD_PCASP8_BIND2	Membrane oligomerization and cleavage activates the caspase-8 (FLICE/MACHalpha1) death signal.,Martin DA, Siegel RM, Zheng L, Lenardo MJ.,J Biol Chem.,9468483,1998,2,20
FADD_PCASP8_BIND2	The apoptotic signaling pathway activated by Toll-like receptor-2.,Aliprantis AO, Yang RB, Weiss DS, Godowski P, Zychlinsky A.,EMBO J.,10880445,2000,7,3
FMAP3K1_PROC	ECSIT is an evolutionarily conserved intermediate in the Toll/IL-1 signal transduction pathway.,Kopp E, Medzhitov R, Carothers J, Xiao C, Douglas I, Janeway CA, Ghosh S.,Genes Dev.,10465784,1999,8,15
FMAP3K1_PROC	Fas-induced proteolytic activation and intracellular redistribution of the stress-signaling kinase MEKK1.,Deak JC, Cross JV, Lewis M, Qian Y, Parrott LA, Distelhorst CW, Templeton DJ.,Proc Natl Acad Sci USA.,9576928,1998,5,12

FOS_DEGR	c-Fos phosphorylation induced by H2O2 prevents proteasomal degradation of c-Fos in cardiomyocytes,Coronella-Wood J, Terrand J, Sun H, Chen QM.,J Biol Chem,15136564,2004,8,6
FOS_DEPH	c-Fos phosphorylation induced by H2O2 prevents proteasomal degradation of c-Fos in cardiomyocytes,Coronella-Wood J, Terrand J, Sun H, Chen QM.,J Biol Chem,15136564,2004,8,6
FOS_JUN_BIND	AP-1 function and regulation,Karin M, Liu Z, Zandi E.,Curr Opin Cell Biol.,9069263,1997,4
GENERIC_K_RAF1	Phosphorylation of Raf-1 by p21-activated kinase 1 and Src regulates Raf-1 autoinhibition.,Tran NH, Frost JA.,J Biol Chem.,12551923,2003,3,28
GENERIC_K_RAF1	Positive and negative regulation of Raf kinase activity and function by phosphorylation.,Chong H, Lee J, Guan KL.,EMBO J.,11447113,2001,7,16
GENERIC_K_RAF1	Untying the regulation of the Raf-1 kinase.,Dhillon AS, Kolch W.,Arch Biochem Biophys.,12127063,2002,8,1
GENERIC_PP_DEPH	Structure and regulation of MAPK phosphatases.,Farooq A, Zhou MM.,Cell Signal.,15115656,2004,7
GP91_P22PHOX_BIND	Phagocyte NADPH oxidase: a multicomponent enzyme essential for host defenses.,El-Benna J, Dang PM, Gougerot-Pocidalo MA, Elbim C.,Arch Immunol Ther Exp (Warsz).,15995580,2005,6
GP91_P22PHOX_BIND	Rac binding to p67(phox). Structural basis for interactions of the Rac1 effector region and insert region with components of the respiratory burst oxidase.,Nisimoto Y, Freeman JL, Motalebi SA, Hirshberg M, Lambeth JD.,J Biol Chem.,9228059,1997,7,25
GP91_P22PHOX_BIND	Rho GTPases and their effector proteins.,Bishop AL, Hall A.,Biochem J.,10816416,2000,6,1
GSK3B_NFKB	Phosphorylation of serine 468 by GSK-3beta negatively regulates basal p65 NF-kappaB activity.,Buss H, Dörrie A, Schmitz ML, Frank R, Livingstone M, Resch K, Kracht M.,J Biol Chem.,15465828,2004,11,26
GSK3B_NFKB	Post-translational modifications regulating the activity and function of the nuclear factor kappa B pathway.,Perkins ND.,Oncogene.,17072324,2006,10,30
GSK3B_NFKB_P3K	Phosphorylation of serine 468 by GSK-3beta negatively regulates basal p65 NF-kappaB activity.,Buss H, Dörrie A, Schmitz ML, Frank R, Livingstone M, Resch K, Kracht M.,J Biol Chem.,15465828,2004,11,26
GSK3B_NFKB_P3K	Post-translational modifications regulating the activity and function of the nuclear factor kappa B pathway.,Perkins ND.,Oncogene.,17072324,2006,10,30
HNRNPA1_DEPH	Regulation of in vitro nucleic acid strand annealing activity of heterogeneous nuclear ribonucleoprotein protein A1 by reversible phosphorylation.,Idriss H, Kumar A, Casas-Finet JR, Guo H, Damuni Z, Wilson SH.,Biochemistry.,7727389,1994,9,20
IKBA_DEGR	Missing pieces in the NF-kappaB puzzle.,Ghosh S, Karin M.,Cell.,11983155,2002,4
IKBA_DEGR2	Missing pieces in the NF-kappaB puzzle.,Ghosh S, Karin M.,Cell.,11983155,2002,4
IKBA_TRAN	Distinct functional properties of IkappaB alpha and IkappaB beta.,Tran K, Merika M, Thanos D.,Mol Cell Biol.,9271416,1997,9
IKBA_TRAN	Nuclear localization of I kappa B alpha promotes active transport of NF-kappa B from the nucleus to the cytoplasm.,Arenzana-Seisdedos F, Turpin P, Rodriguez M, Thomas D, Hay RT, Virelizier JL, Dargemont C.,J Cell Sci.,9057089,1997,2
IKBA_UBIQ	Inducible degradation of IkappaBalpha by the proteasome requires interaction with the F-box protein h-betaTrCP.,Kroll M, Margottin F, Kohl A, Renard P, Durand H, Concordet JP, Bachelerie F, Arenzana-Seisdedos F, Benarous R.,J Biol Chem.,10075690,1999,3,19
IKBA_UBIQ	Signal-induced ubiquitination of IkappaBalpha by the F-box protein Slimb/beta-TrCP.,Spencer E, Jiang J, Chen ZJ.,Genes Dev.,9990853,1999,2,1

IKBA_UBIQ	Signaling to NF-kappaB.,Hayden MS, Ghosh S.,Genes Dev.,15371334,2004,9,15
IKBA_UBIQ	beta-TrCP mediates the signal-induced ubiquitination of IkappaBbeta.,Wu C, Ghosh S.,J Biol Chem.,10514424,1999,10,15
IKBA_UBIQ2	Inhibition of NF-kappa-B cellular function via specific targeting of the I-kappa-B-ubiquitin ligase.,Yaron A, Gonen H, Alkalay I, Hatzubai A, Jung S, Beyth S, Mercurio F, Manning AM, Ciechanover A, Ben-Neriah Y.,EMBO J.,9351830,1998,11,3
IKBA_UBIQ3	Inhibition of NF-kappa-B cellular function via specific targeting of the I-kappa-B-ubiquitin ligase.,Yaron A, Gonen H, Alkalay I, Hatzubai A, Jung S, Beyth S, Mercurio F, Manning AM, Ciechanover A, Ben-Neriah Y.,EMBO J.,9351830,1998,11,3
IKBA_UBIQ3	Missing pieces in the NF-kappaB puzzle.,Ghosh S, Karin M.,Cell.,11983155,2002,4
IKBA_UBIQ4	Impaired degradation of inhibitory subunit of NF-kappa B (I kappa B) and beta-catenin as a result of targeted disruption of the beta-TrCP1 gene.,Nakayama K, Hatakeyama S, Maruyama S, Kikuchi A, Onoé K, Good RA, Nakayama KI.,Proc Natl Acad Sci USA,12843402,2003,7,22
IKBA_UBIQ4	Missing pieces in the NF-kappaB puzzle.,Ghosh S, Karin M.,Cell.,11983155,2002,4
IKBB_DEGR	Missing pieces in the NF-kappaB puzzle.,Ghosh S, Karin M.,Cell.,11983155,2002,4
IKBB_DEGR2	Missing pieces in the NF-kappaB puzzle.,Ghosh S, Karin M.,Cell.,11983155,2002,4
IKBB_TRAN	Basal phosphorylation of the PEST domain in the I(kappa)B(beta) regulates its functional interaction with the c-rel proto-oncogene product.,Chu ZL, McKinsey TA, Liu L, Qi X, Ballard DW.,Mol Cell Biol.,8887627,1996,9
IKBB_TRAN	Distinct functional properties of IkappaB alpha and IkappaB beta.,Tran K, Merika M, Thanos D.,Mol Cell Biol.,9271416,1997,9
IKBB_UBIQ	Inducible degradation of IkappaBalpha by the proteasome requires interaction with the F-box protein h-betaTrCP.,Kroll M, Margottin F, Kohl A, Renard P, Durand H, Concordet JP, Bachelerie F, Arenzana-Seisdedos F, Benarous R.,J Biol Chem.,10075690,1999,3,19
IKBB_UBIQ	Signal-induced ubiquitination of IkappaBalpha by the F-box protein Slimb/beta-TrCP.,Spencer E, Jiang J, Chen ZJ.,Genes Dev.,9990853,1999,2,1
IKBB_UBIQ	Signaling to NF-kappaB.,Hayden MS, Ghosh S.,Genes Dev.,15371334,2004,9,15
IKBB_UBIQ	beta-TrCP mediates the signal-induced ubiquitination of IkappaBbeta.,Wu C, Ghosh S.,J Biol Chem.,10514424,1999,10,15
IKKE_IRF3	A20 is a negative regulator of IFN regulatory factor 3 signaling.,Saitoh T, Yamamoto M, Miyagishi M, Taira K, Nakanishi M, Fujita T, Akira S, Yamamoto N, Yamaoka S.,J Immunol.,15661910,2005,2,1
IKKE_IRF3	IKKepsilon and TBK1 are essential components of the IRF3 signaling pathway.,Fitzgerald KA, McWhirter SM, Faia KL, Rowe DC, Latz E, Golenbock DT, Coyle AJ, Liao SM, Maniatis T.,Nat Immunol.,12692549,2003,5
IKKE_IRF3	Identification of Ser-386 of interferon regulatory factor 3 as critical target for inducible phosphorylation that determines activation.,Mori M, Yoneyama M, Ito T, Takahashi K, Inagaki F, Fujita T.,J Biol Chem.,14703513,2004,3,12
IKKE_IRF3	Structural and functional analysis of interferon regulatory factor 3: localization of the transactivation and autoinhibitory domains.,Lin R, Mamane Y, Hiscott J.,Mol Cell Biol.,10082512,1999,4
IKKE_NFKB	Inducible phosphorylation of NF-kappa B p65 at serine 468 by T cell costimulation is mediated by IKK epsilon.,Mattioli I, Geng H, Sebald A, Hodel M, Bucher C, Kracht M, Schmitz ML.,J Biol Chem.,16407239,2006,3,10

IKKE_NFKB	Post-translational modifications regulating the activity and function of the nuclear factor kappa B pathway.,Perkins ND.,Oncogene.,17072324,2006,10,30
IKKE_NFKB2	Post-translational modifications regulating the activity and function of the nuclear factor kappa B pathway.,Perkins ND.,Oncogene.,17072324,2006,10,30
IKKE_NFKB2	TOO LONG: See PubMed ID,Buss H, Dörrie A, Schmitz ML, Hoffmann E, Resch K, Kracht M.,J Biol Chem.,15489227,2004,12,31
IKKE_NFKB_P3K	Inducible phosphorylation of NF-kappa B p65 at serine 468 by T cell costimulation is mediated by IKK epsilon.,Mattioli I, Geng H, Sebald A, Hodel M, Bucher C, Kracht M, Schmitz ML.,J Biol Chem.,16407239,2006,3,10
IKKE_NFKB_P3K	Post-translational modifications regulating the activity and function of the nuclear factor kappa B pathway.,Perkins ND.,Oncogene.,17072324,2006,10,30
IKKE_NFKB_P5K	Post-translational modifications regulating the activity and function of the nuclear factor kappa B pathway.,Perkins ND.,Oncogene.,17072324,2006,10,30
IKKE_NFKB_P5K	TOO LONG: See PubMed ID,Buss H, Dörrie A, Schmitz ML, Hoffmann E, Resch K, Kracht M.,J Biol Chem.,15489227,2004,12,31
IKKE_PHOS	TOO LONG: See pubmed ID,Sato S, Sugiyama M, Yamamoto M, Watanabe Y, Kawai T, Takeda K, Akira S.,J Immunol.,14530355,2003,10,15
IKKE_PHOS	Triggering the interferon antiviral response through an IKK-related pathway.,Sharma S, tenOever BR, Grandvaux N, Zhou GP, Lin R, Hiscott J.,Science.,12702806,2003,5,16
IKK_6PHOS	Positive and negative regulation of IkappaB kinase activity through IKKbeta subunit phosphorylation.,Delhase M, Hayakawa M, Chen Y, Karin M.,Science.,10195894,1999,4,9
IKK_IKBA	IKKalpha limits macrophage NF-kappaB activation and contributes to the resolution of inflammation.,Lawrence T, Bebien M, Liu GY, Nizet V, Karin M.,Nature.,15858576,2005,4,28
IKK_IKBA	Signaling to NF-kappaB.,Hayden MS, Ghosh S.,Genes Dev.,15371334,2004,9,15
IKK_IKBA	TRAIL-R as a negative regulator of innate immune cell responses.,Diehl GE, Yue HH, Hsieh K, Kuang AA, Ho M, Morici LA, Lenz LL, Cado D, Riley LW, Winoto A.,Immunity.,15589175,2004,12
IKK_IKBB	IKKalpha limits macrophage NF-kappaB activation and contributes to the resolution of inflammation.,Lawrence T, Bebien M, Liu GY, Nizet V, Karin M.,Nature.,15858576,2005,4,28
IKK_IKBB	Signaling to NF-kappaB.,Hayden MS, Ghosh S.,Genes Dev.,15371334,2004,9,15
IKK_IKBB	TRAIL-R as a negative regulator of innate immune cell responses.,Diehl GE, Yue HH, Hsieh K, Kuang AA, Ho M, Morici LA, Lenz LL, Cado D, Riley LW, Winoto A.,Immunity.,15589175,2004,12
IKK_MAP3K8	Phosphorylation at Thr-290 regulates Tpl2 binding to NF-kappaB1/p105 and Tpl2 activation and degradation by lipopolysaccharide.,Cho J, Tsichlis PN.,Proc Natl Acad Sci USA,15699325,2005,2,15
IKK_MAP3K8	Tpl2 (tumor progression locus 2) phosphorylation at Thr290 is induced by lipopolysaccharide via an Ikappa-B Kinase-beta-dependent pathway and is required for Tpl2 activation by external signals.,Cho J, Melnick M, Solidakis GP, Tsichlis PN.,J Biol Chem.,15778223,2005,5,27
IKK_MAP3K8(p52)_NFKB	IkappaB kinase is an essential component of the Tpl2 signaling pathway.,Waterfield M, Jin W, Reiley W, Zhang M, Sun SC.,Mol Cell Biol.,15199157,2004,7
IKK_MAP3K8(p52)_NFKB	Tpl2 (tumor progression locus 2) phosphorylation at Thr290 is induced by lipopolysaccharide via an Ikappa-B Kinase-beta-dependent pathway and is required for Tpl2 activation by external signals.,Cho J, Melnick M, Solidakis GP, Tsichlis PN.,J Biol Chem.,15778223,2005,5,27
IKK_NFKB	IKKbeta phosphorylates p65 at S468 in transactivaton domain 2.,Schwabe RF, Sakurai H. ,FASEB J.,16046471,2005,10

IKK_NFKB	Post-translational modifications regulating the activity and function of the nuclear factor kappa B pathway.,Perkins ND.,Oncogene.,17072324,2006,10,30
IKK_NFKB(p105)	Direct phosphorylation of NF-kappaB1 p105 by the IkappaB kinase complex on serine 927 is essential for signal-induced p105 proteolysis.,Salmerón A, Janzen J, Soneji Y, Bump N, Kamens J, Allen H, Ley SC.,J Biol Chem.,11297557,2001,6,22
IKK_NFKB(p105)	Shared pathways of IkappaB kinase-induced SCF(betaTrCP)-mediated ubiquitination and degradation for the NF-kappaB precursor p105 and IkappaBalpha.,Heissmeyer V, Krappmann D, Hatada EN, Scheidereit C.,Mol Cell Biol.,11158290,2001,2
IKK_NFKB(p105)	Signaling to NF-kappaB.,Hayden MS, Ghosh S.,Genes Dev.,15371334,2004,9,15
IKK_NFKB2	IKKbeta phosphorylates p65 at S468 in transactivaton domain 2.,Schwabe RF, Sakurai H. ,FASEB J.,16046471,2005,10
IKK_NFKB2	Post-translational modifications regulating the activity and function of the nuclear factor kappa B pathway.,Perkins ND.,Oncogene.,17072324,2006,10,30
IKK_NFKB3	IKKbeta phosphorylates p65 at S468 in transactivaton domain 2.,Schwabe RF, Sakurai H. ,FASEB J.,16046471,2005,10
IKK_NFKB3	Post-translational modifications regulating the activity and function of the nuclear factor kappa B pathway.,Perkins ND.,Oncogene.,17072324,2006,10,30
IKK_NFKB_P3K	IKKbeta phosphorylates p65 at S468 in transactivaton domain 2.,Schwabe RF, Sakurai H. ,FASEB J.,16046471,2005,10
IKK_NFKB_P3K	Post-translational modifications regulating the activity and function of the nuclear factor kappa B pathway.,Perkins ND.,Oncogene.,17072324,2006,10,30
IKK_NFKB_P5K	IKKbeta phosphorylates p65 at S468 in transactivaton domain 2.,Schwabe RF, Sakurai H. ,FASEB J.,16046471,2005,10
IKK_NFKB_P5K	Post-translational modifications regulating the activity and function of the nuclear factor kappa B pathway.,Perkins ND.,Oncogene.,17072324,2006,10,30
IKK_NFKB_P5K2	IKKbeta phosphorylates p65 at S468 in transactivaton domain 2.,Schwabe RF, Sakurai H. ,FASEB J.,16046471,2005,10
IKK_NFKB_P5K2	Post-translational modifications regulating the activity and function of the nuclear factor kappa B pathway.,Perkins ND.,Oncogene.,17072324,2006,10,30
IKK_PKR_BIND	Missing pieces in the NF-kappaB puzzle.,Ghosh S, Karin M.,Cell.,11983155,2002,4
IKK_PKR_BIND	PKR stimulates NF-kappaB irrespective of its kinase function by interacting with the IkappaB kinase complex.,Bonnet MC, Weil R, Dam E, Hovanessian AG, Meurs EF.,Mol Cell Biol.,10848580,2000,7
IKK_PKR_BIND	The N-terminus of PKR is responsible for the activation of the NF-kappaB signaling pathway by interacting with the IKK complex.,Bonnet MC, Daurat C, Ottone C, Meurs EF.,Cell Signal.,16600570,2006,9
IKK_PKR_PHOS	Missing pieces in the NF-kappaB puzzle.,Ghosh S, Karin M.,Cell.,11983155,2002,4
IKK_PKR_PHOS	PKR stimulates NF-kappaB irrespective of its kinase function by interacting with the IkappaB kinase complex.,Bonnet MC, Weil R, Dam E, Hovanessian AG, Meurs EF.,Mol Cell Biol.,10848580,2000,7
IKK_PKR_PHOS	The N-terminus of PKR is responsible for the activation of the NF-kappaB signaling pathway by interacting with the IKK complex.,Bonnet MC, Daurat C, Ottone C, Meurs EF.,Cell Signal.,16600570,2006,9
IKK_PKR_TRAF6_BIND	Missing pieces in the NF-kappaB puzzle.,Ghosh S, Karin M.,Cell.,11983155,2002,4
IKK_PKR_TRAF6_BIND	PKR stimulates NF-kappaB irrespective of its kinase function by interacting with the IkappaB kinase complex.,Bonnet MC, Weil R, Dam E, Hovanessian AG, Meurs EF.,Mol Cell Biol.,10848580,2000,7

IKK_PKR_TRAF6_BIND	The N-terminus of PKR is responsible for the activation of the NF-kappaB signaling pathway by interacting with the IKK complex.,Bonnet MC, Daurat C, Ottone C, Meurs EF.,Cell Signal.,16600570,2006,9
IKK_PKR_TRAF6_PHOS	Missing pieces in the NF-kappaB puzzle.,Ghosh S, Karin M.,Cell.,11983155,2002,4
IKK_PKR_TRAF6_PHOS	PKR stimulates NF-kappaB irrespective of its kinase function by interacting with the IkappaB kinase complex.,Bonnet MC, Weil R, Dam E, Hovanessian AG, Meurs EF.,Mol Cell Biol.,10848580,2000,7
IKK_PKR_TRAF6_PHOS	The N-terminus of PKR is responsible for the activation of the NF-kappaB signaling pathway by interacting with the IKK complex.,Bonnet MC, Daurat C, Ottone C, Meurs EF.,Cell Signal.,16600570,2006,9
IKK_RIP1_TICAM1P_BIND	TOO LONG: See pubmed ID,Sato S, Sugiyama M, Yamamoto M, Watanabe Y, Kawai T, Takeda K, Akira S.,J Immunol.,14530355,2003,10,15
IKK_RIP1_TICAM1P_PHOS	TOO LONG: See pubmed ID,Sato S, Sugiyama M, Yamamoto M, Watanabe Y, Kawai T, Takeda K, Akira S.,J Immunol.,14530355,2003,10,15
IKK_RIP2_NOD1P_BIND	CARD4/Nod1 mediates NF-kappaB and JNK activation by invasive Shigella flexneri.,Girardin SE, Tournebize R, Mavris M, Page AL, Li X, Stark GR, Bertin J, DiStefano PS, Yaniv M, Sansonetti PJ, Philpott DJ.,EMBO Rep.,11463746,2001,8
IKK_RIP2_NOD1P_PHOS	CARD4/Nod1 mediates NF-kappaB and JNK activation by invasive Shigella flexneri.,Girardin SE, Tournebize R, Mavris M, Page AL, Li X, Stark GR, Bertin J, DiStefano PS, Yaniv M, Sansonetti PJ, Philpott DJ.,EMBO Rep.,11463746,2001,8
IKK_RIP2_NOD2P_BIND	Nod2, a Nod1/Apaf-1 family member that is restricted to monocytes and activates NF-kappaB.,Ogura Y, Inohara N, Benito A, Chen FF, Yamaoka S, Nunez G.,J Biol Chem.,11087742,2001,2,16
IKK_RIP2_NOD2P_PHOS	Nod2, a Nod1/Apaf-1 family member that is restricted to monocytes and activates NF-kappaB.,Ogura Y, Inohara N, Benito A, Chen FF, Yamaoka S, Nunez G.,J Biol Chem.,11087742,2001,2,16
IKK_RIP2_TRIP6_TRAF2_BIND	TRIP6 is a RIP2-associated common signaling component of multiple NF-kappaB activation pathways.,Li L, Bin LH, Li F, Liu Y, Chen D, Zhai Z, Shu HB.,J Cell Sci.,15657077,2005,2,1
IKK_RIP2_TRIP6_TRAF2_PHOS	TRIP6 is a RIP2-associated common signaling component of multiple NF-kappaB activation pathways.,Li L, Bin LH, Li F, Liu Y, Chen D, Zhai Z, Shu HB.,J Cell Sci.,15657077,2005,2,1
IKK_SRC_BIND	Functional role of c-Src in IL-1-induced NF-kappa B activation: c-Src is a component of the IKK complex.,Funakoshi-Tago M, Tago K, Andoh K, Sonoda Y, Tominaga S, Kasahara T.,J Biochem (Tokyo).,15749833,2005,2
IL1/L/AP_PHOS	Phosphatidylinositol 3-kinase is recruited to a specific site in the activated IL-1 receptor I.,Marmiroli S, Bavelloni A, Faenza I, Sirri A, Ognibene A, Cenni V, Tsukada J, Koyama Y, Ruzzene M, Ferri A, Auron PE, Toker A, Maraldi NM.,FEBS Lett.,9821957,1998,10,30
IL1/L_IL1RAP_BIND	Molecular cloning and characterization of a second subunit of the interleukin 1 receptor complex.,Greenfeder SA, Nunes P, Kwee L, Labow M, Chizzonite RA, Ju G.,J Biol Chem.,7775431,1995,6,9
IL1A_TRAN	Differential sensitivity of interleukin-1 alpha and -beta precursor proteins to cleavage by calpain, a calcium-dependent protease.,Kavita U, Mizel SB.,J Biol Chem.,7499244,1995,11,17
IL1B_TRAN	Differential sensitivity of interleukin-1 alpha and -beta precursor proteins to cleavage by calpain, a calcium-dependent protease.,Kavita U, Mizel SB.,J Biol Chem.,7499244,1995,11,17
IL1R1_BIND	Decoy receptors: a strategy to regulate inflammatory cytokines and chemokines.,Mantovani A, Locati M, Vecchi A, Sozzani S, Allavena P.,Trends Immunol.,11377293,2001,6

IL1R1_BIND	Soluble type II interleukin 1 (IL-1) receptor binds and blocks processing of IL-1 beta precursor and loses affinity for IL-1 receptor antagonist.,Symons JA, Young PR, Duff GW.,Proc Natl Acad Sci USA,7878046,1995,2,28
IL1R1_BIND	The membrane form of the type II IL-1 receptor accounts for inhibitory function.,Neumann D, Kollewe C, Martin MU, Boraschi D.,J Immunol.,10975853,2000,9,15
IL1R1_BIND	The type II 'decoy' receptor: a novel regulatory pathway for interleukin 1.,Colotta F, Dower SK, Sims JE, Mantovani A.,Immunol Today.,7848516,1994,12
IL1R1_PI3K1A	Phosphatidylinositol 3-kinase is recruited to a specific site in the activated IL-1 receptor I.,Marmiroli S, Bavelloni A, Faenza I, Sirri A, Ognibene A, Cenni V, Tsukada J, Koyama Y, Ruzzene M, Ferri A, Auron PE, Toker A, Maraldi NM.,FEBS Lett.,9821957,1998,10,30
IL1R1_PI3K1A	The IL-1 receptor accessory protein is essential for PI 3-kinase recruitment and activation.,Reddy SA, Lin YF, Huang HJ, Samanta AK, Liao WS.,Biochem Biophys Res Commun.,15044087,2004,4,16
IL1R2_IL1RAP_BIND	Decoy receptors: a strategy to regulate inflammatory cytokines and chemokines.,Mantovani A, Locati M, Vecchi A, Sozzani S, Allavena P.,Trends Immunol.,11377293,2001,6
IL1R2_IL1RAP_BIND	The type II IL-1 receptor interacts with the IL-1 receptor accessory protein: a novel mechanism of regulation of IL-1 responsiveness.,Lang D, Knop J, Wesche H, Raffetseder U, Kurrle R, Boraschi D, Martin MU.,J Immunol.,9862719,1998,12,15
IMPA3_TRAN	NF-{kappa}B is transported into the nucleus by importin {alpha}3 and importin {alpha}4.,Fagerlund R, Kinnunen L, Köhler M, Julkunen I, Melén K.,J Biol Chem.,15677444,2005,4,22
IP3R_mi145p_BIND	Inositol 1,4,5-trisphosphate receptors as signal integrators.,Patterson RL, Boehning D, Snyder SH.,Annu Rev Biochem.,15189149,2004
IRAK1C_TOLLIP_BIND	A novel splice variant of interleukin-1 receptor (IL-1R)-associated kinase 1 plays a negative regulatory role in Toll/IL-1R-induced inflammatory signaling.,Rao N, Nguyen S, Ngo K, Fung-Leung WP.,Mol Cell Biol.,16024789,2005,8
IRAK1_DEGR	Interleukin-1 (IL-1) receptor-associated kinase-dependent IL-1-induced signaling complexes phosphorylate TAK1 and TAB2 at the plasma membrane and activate TAK1 in the cytosol.,Jiang Z, Ninomiya-Tsuji J, Qian Y, Matsumoto K, Li X.,Mol Cell Biol.,12242293,2002,10
IRAK1_ECSIT_BIND	ECSIT is an evolutionarily conserved intermediate in the Toll/IL-1 signal transduction pathway.,Kopp E, Medzhitov R, Carothers J, Xiao C, Douglas I, Janeway CA, Ghosh S.,Genes Dev.,10465784,1999,8,15
IRAK1_ECSIT_FMAP3K1_BIND	ECSIT is an evolutionarily conserved intermediate in the Toll/IL-1 signal transduction pathway.,Kopp E, Medzhitov R, Carothers J, Xiao C, Douglas I, Janeway CA, Ghosh S.,Genes Dev.,10465784,1999,8,15
IRAK1_ECSIT_FMAP3K1_BIND	Fas-induced proteolytic activation and intracellular redistribution of the stress-signaling kinase MEKK1.,Deak JC, Cross JV, Lewis M, Qian Y, Parrott LA, Distelhorst CW, Templeton DJ.,Proc Natl Acad Sci USA.,9576928,1998,5,12
IRAK1_MAP3K3_BIND	Differential regulation of interleukin 1 receptor and Toll-like receptor signaling by MEKK3.,Huang Q, Yang J, Lin Y, Walker C, Cheng J, Liu ZG, Su B.,Nat Immunol.,14661019,2004,1
IRAK1_MAP3K5_BIND	ROS-dependent activation of the TRAF6-ASK1-p38 pathway is selectively required for TLR4-mediated innate immunity.,Matsuzawa A, Saegusa K, Noguchi T, Sadamitsu C, Nishitoh H, Nagai S, Koyasu S, Matsumoto K, Takeda K, Ichijo H.,Nat Immunol.,15864310,2005,6
IRAK1_MAP3K7_4PHOS	Interleukin-1 (IL-1) receptor-associated kinase-dependent IL-1-induced signaling complexes phosphorylate TAK1 and TAB2 at the plasma membrane and activate TAK1 in the cytosol.,Jiang Z, Ninomiya-Tsuji J, Qian Y, Matsumoto K, Li X.,Mol Cell Biol.,12242293,2002,10

IRAK1_MAP3K7_BIND	Interleukin-1 (IL-1) receptor-associated kinase-dependent IL-1-induced signaling complexes phosphorylate TAK1 and TAB2 at the plasma membrane and activate TAK1 in the cytosol.,Jiang Z, Ninomiya-Tsuji J, Qian Y, Matsumoto K, Li X.,Mol Cell Biol.,12242293,2002,10
IRAK1_MAP3K7_BIND	TAB2 and TAB3 activate the NF-kappaB pathway through binding to polyubiquitin chains.,Kanayama A, Seth RB, Sun L, Ea CK, Hong M, Shaito A, Chiu YH, Deng L, Chen ZJ.,Mol Cell.,15327770,2004,8,27
IRAK1_MAP3K7_DISS	Interleukin-1 (IL-1) receptor-associated kinase-dependent IL-1-induced signaling complexes phosphorylate TAK1 and TAB2 at the plasma membrane and activate TAK1 in the cytosol.,Jiang Z, Ninomiya-Tsuji J, Qian Y, Matsumoto K, Li X.,Mol Cell Biol.,12242293,2002,10
IRAK1_PLNO3_BIND	Pellino3 is a novel upstream regulator of p38 MAPK and activates CREB in a p38-dependent manner.,Butler MP, Hanly JA, Moynagh PN.,J Biol Chem.,15917247,2005,7,29
IRAK1_TIFA_3UBIQ	Activation of the IkappaB kinase complex by TRAF6 requires a dimeric ubiquitin-conjugating enzyme complex and a unique polyubiquitin chain.,Deng L, Wang C, Spencer E, Yang L, Braun A, You J, Slaughter C, Pickart C, Chen ZJ.,Cell,11057907,2000,10,13
IRAK1_TIFA_A20_BIND	The ubiquitin-modifying enzyme A20 is required for termination of Toll-like receptor responses.,Boone DL, Turer EE, Lee EG, Ahmad RC, Wheeler MT, Tsui C, Hurley P, Chien M, Chai S, Hitotsumatsu O, McNally E, Pickart C, Ma A.,Nat Immunol.,15334086,2004,10
IRAK1_TIFA_CYLD_BIND	TAB2 and TAB3 activate the NF-kappaB pathway through binding to polyubiquitin chains.,Kanayama A, Seth RB, Sun L, Ea CK, Hong M, Shaito A, Chiu YH, Deng L, Chen ZJ.,Mol Cell.,15327770,2004,8,27
IRAK1_TIFA_DEBQ	TAB2 and TAB3 activate the NF-kappaB pathway through binding to polyubiquitin chains.,Kanayama A, Seth RB, Sun L, Ea CK, Hong M, Shaito A, Chiu YH, Deng L, Chen ZJ.,Mol Cell.,15327770,2004,8,27
IRAK1_TIFA_DEBQ2	The ubiquitin-modifying enzyme A20 is required for termination of Toll-like receptor responses.,Boone DL, Turer EE, Lee EG, Ahmad RC, Wheeler MT, Tsui C, Hurley P, Chien M, Chai S, Hitotsumatsu O, McNally E, Pickart C, Ma A.,Nat Immunol.,15334086,2004,10
IRAK1_TOLLIP_BIND	Negative regulation of toll-like receptor-mediated signaling by Tollip.,Zhang G, Ghosh S.,J Biol Chem.,11751856,2002,3,1
IRAK1_TOLLIP_BIND	Tollip, a new component of the IL-1RI pathway, links IRAK to the IL-1 receptor.,Burns K, Clatworthy J, Martin L, Martinon F, Plumpton C, Maschera B, Lewis A, Ray K, Tschopp J, Volpe F.,Nat Cell Biol.,10854325,2000,6
IRAK_MYD_TIR_2PHOS	Sequential autophosphorylation steps in the interleukin-1 receptor-associated kinase-1 regulate its availability as an adapter in interleukin-1 signaling.,Kollewe C, Mackensen AC, Neumann D, Knop J, Cao P, Li S, Wesche H, Martin MU.,J Biol Chem.,14625308,2004,2,13
IRAK_MYD_TIR_BIND	IRAK-4: a novel member of the IRAK family with the properties of an IRAK-kinase.,Li S, Strelow A, Fontana EJ, Wesche H.,Proc Natl Acad Sci USA,11960013,2002,4,16
IRAK_MYD_TIR_BIND	MyD88: an adapter that recruits IRAK to the IL-1 receptor complex.,Wesche H, Henzel WJ, Shillinglaw W, Li S, Cao Z.,Immunity,9430229,1997,12
IRAK_MYD_TIR_PHOS	IRAK-4: a novel member of the IRAK family with the properties of an IRAK-kinase.,Li S, Strelow A, Fontana EJ, Wesche H.,Proc Natl Acad Sci USA,11960013,2002,4,16
IRAK_MYD_TIR_PHOS	Inhibition of interleukin 1 receptor/Toll-like receptor signaling through the alternatively spliced, short form of MyD88 is due to its failure to recruit IRAK-4.,Burns K, Janssens S, Brissoni B, Olivos N, Beyaert R, Tschopp J.,J Exp Med.,12538665,2003,1,20

IRAK_MYD_TIR_PHOS	Regulation of interleukin-1- and lipopolysaccharide-induced NF-kappaB activation by alternative splicing of MyD88.,Janssens S, Burns K, Tschopp J, Beyaert R.,Curr Biol.,11909531,2002,3,19
IRAK_MYD_TIR_PHOS	Sequential autophosphorylation steps in the interleukin-1 receptor-associated kinase-1 regulate its availability as an adapter in interleukin-1 signaling.,Kollewe C, Mackensen AC, Neumann D, Knop J, Cao P, Li S, Wesche H, Martin MU.,J Biol Chem.,14625308,2004,2,13
IRAK_TOLLIP_TRAF6_BIND	A novel splice variant of interleukin-1 receptor (IL-1R)-associated kinase 1 plays a negative regulatory role in Toll/IL-1R-induced inflammatory signaling.,Rao N, Nguyen S, Ngo K, Fung-Leung WP.,Mol Cell Biol.,16024789,2005,8
IRAK_TRAF6_BIND	Activation of the IkappaB kinase complex by TRAF6 requires a dimeric ubiquitin-conjugating enzyme complex and a unique polyubiquitin chain.,Deng L, Wang C, Spencer E, Yang L, Braun A, You J, Slaughter C, Pickart C, Chen ZJ.,Cell,11057907,2000,10,13
IRAK_TRAF6_BIND	Identification of TIFA as an adapter protein that links tumor necrosis factor receptor-associated factor 6 (TRAF6) to interleukin-1 (IL-1) receptor-associated kinase-1 (IRAK-1) in IL-1 receptor signaling.,Takatsuna H, Kato H, Gohda J, Akiyama T, Moriya A, Okamoto Y, Yamagata Y, Otsuka M, Umezawa K, Semba K, Inoue J.,J Biol Chem.,12566447,2003,4,4
IRAK_TRAF6_BIND	Structure and interactions of the ubiquitin-conjugating enzyme variant human Uev1a: implications for enzymatic synthesis of polyubiquitin chains.,Hau DD, Lewis MJ, Saltibus LF, Pastushok L, Xiao W, Spyracopoulos L.,Biochemistry,16893187,2006,8,15
IRAK_TRAF6_DISS	IRAK-M is a negative regulator of Toll-like receptor signaling.,Kobayashi K, Hernandez LD, Galán JE, Janeway CA, Medzhitov R, Flavell RA.,Cell,12150927,2002,7,26
IRAK_TRAF6_DISS	IRAK-M is a novel member of the Pelle/interleukin-1 receptor-associated kinase (IRAK) family.,Wesche H, Gao X, Li X, Kirschning CJ, Stark GR, Cao Z.,J Biol Chem.,10383454,1999,7,2
IRAK_TRAF6_DISS	Pellino 1 is required for interleukin-1 (IL-1)-mediated signaling through its interaction with the IL-1 receptor-associated kinase 4 (IRAK4)-IRAK-tumor necrosis factor receptor-associated factor 6 (TRAF6) complex.,Jiang Z, Johnson HJ, Nie H, Qin J, Bird TA, Li X.,J Biol Chem.,12496252,2003,3,28
IRAK_TRAF6_DISS	Sequential autophosphorylation steps in the interleukin-1 receptor-associated kinase-1 regulate its availability as an adapter in interleukin-1 signaling.,Kollewe C, Mackensen AC, Neumann D, Knop J, Cao P, Li S, Wesche H, Martin MU.,J Biol Chem.,14625308,2004,2,13
IRAK_TRAF6_DISS	The murine IRAK2 gene encodes four alternatively spliced isoforms, two of which are inhibitory.,Hardy MP, O'Neill LA.,J Biol Chem.,15082713,2004,6,25
IRAK_TRAF6_DISS	Toll-like receptor signaling.,Akira S.,J Biol Chem.,12893815,2003,10,3
IRAK_TRAF6_TIRAP_BIND	Activation of the IkappaB kinase complex by TRAF6 requires a dimeric ubiquitin-conjugating enzyme complex and a unique polyubiquitin chain.,Deng L, Wang C, Spencer E, Yang L, Braun A, You J, Slaughter C, Pickart C, Chen ZJ.,Cell,11057907,2000,10,13
IRAK_TRAF6_TIRAP_BIND	Identification of TIFA as an adapter protein that links tumor necrosis factor receptor-associated factor 6 (TRAF6) to interleukin-1 (IL-1) receptor-associated kinase-1 (IRAK-1) in IL-1 receptor signaling.,Takatsuna H, Kato H, Gohda J, Akiyama T, Moriya A, Okamoto Y, Yamagata Y, Otsuka M, Umezawa K, Semba K, Inoue J.,J Biol Chem.,12566447,2003,4,4
IRAK_TRAF6_TIRAP_BIND	Structure and interactions of the ubiquitin-conjugating enzyme variant human Uev1a: implications for enzymatic synthesis of polyubiquitin chains.,Hau DD, Lewis MJ, Saltibus LF, Pastushok L, Xiao W, Spyracopoulos L.,Biochemistry,16893187,2006,8,15

IRAK_TRAF6_TIRAP_DISS	IRAK-M is a negative regulator of Toll-like receptor signaling.,Kobayashi K, Hernandez LD, Galán JE, Janeway CA, Medzhitov R, Flavell RA.,Cell,12150927,2002,7,26
IRAK_TRAF6_TIRAP_DISS	IRAK-M is a novel member of the Pelle/interleukin-1 receptor-associated kinase (IRAK) family.,Wesche H, Gao X, Li X, Kirschning CJ, Stark GR, Cao Z.,J Biol Chem.,10383454,1999,7,2
IRAK_TRAF6_TIRAP_DISS	Pellino 1 is required for interleukin-1 (IL-1)-mediated signaling through its interaction with the IL-1 receptor-associated kinase 4 (IRAK4)-IRAK-tumor necrosis factor receptor-associated factor 6 (TRAF6) complex.,Jiang Z, Johnson HJ, Nie H, Qin J, Bird TA, Li X.,J Biol Chem.,12496252,2003,3,28
IRAK_TRAF6_TIRAP_DISS	Sequential autophosphorylation steps in the interleukin-1 receptor-associated kinase-1 regulate its availability as an adapter in interleukin-1 signaling.,Kollewe C, Mackensen AC, Neumann D, Knop J, Cao P, Li S, Wesche H, Martin MU.,J Biol Chem.,14625308,2004,2,13
IRAK_TRAF6_TIRAP_DISS	The murine IRAK2 gene encodes four alternatively spliced isoforms, two of which are inhibitory.,Hardy MP, O'Neill LA.,J Biol Chem.,15082713,2004,6,25
IRAK_TRAF6_TIRAP_DISS	Toll-like receptor signaling.,Akira S.,J Biol Chem.,12893815,2003,10,3
IRF3_DIME	Identification of Ser-386 of interferon regulatory factor 3 as critical target for inducible phosphorylation that determines activation.,Mori M, Yoneyama M, Ito T, Takahashi K, Inagaki F, Fujita T.,J Biol Chem.,14703513,2004,3,12
IRF3_DIME	Structural and functional analysis of interferon regulatory factor 3: localization of the transactivation and autoinhibitory domains.,Lin R, Mamane Y, Hiscott J.,Mol Cell Biol.,10082512,1999,4
IRF3_ISRE_BIND	Transcriptional activation of alpha/beta interferon genes: interference by nonsegmented negative-strand RNA viruses.,Conzelmann KK.,J Virol.,15827138,2005,5
IRF3_TRAN	Structural and functional analysis of interferon regulatory factor 3: localization of the transactivation and autoinhibitory domains.,Lin R, Mamane Y, Hiscott J.,Mol Cell Biol.,10082512,1999,4
IRF7_ISRE_BIND	IRF family of transcription factors as regulators of host defense.,Taniguchi T, Ogasawara K, Takaoka A, Tanaka N.,Annu Rev Immunol.,11244049,2001
IRF7_TRAN	IRF family of transcription factors as regulators of host defense.,Taniguchi T, Ogasawara K, Takaoka A, Tanaka N.,Annu Rev Immunol.,11244049,2001
JUN_DEGR	c-Fos phosphorylation induced by H2O2 prevents proteasomal degradation of c-Fos in cardiomyocytes,Coronella-Wood J, Terrand J, Sun H, Chen QM.,J Biol Chem,15136564,2004,8,6
JUN_DIME	AP-1 function and regulation,Karin M, Liu Z, Zandi E.,Curr Opin Cell Biol.,9069263,1997,4
KSR1_DEPH1	Protein phosphatase 2A positively regulates Ras signaling by dephosphorylating KSR1 and Raf-1 on critical 14-3-3 binding sites.,Ory S, Zhou M, Conrads TP, Veenstra TD, Morrison DK.,Curr Biol.,12932319,2003,8,19
KSR1_RAF1_MAPK3/1_7PHOS	Properties of MEKs, the kinases that phosphorylate and activate the extracellular signal-regulated kinases.,Zheng CF, Guan KL.,J Biol Chem.,8226933,1993,11,15
KSR1_RAF1_MAPK3/1_9PHOS	KSR: a MAPK scaffold of the Ras pathway?,Morrison DK.,J Cell Sci.,11309192,2001,5
KSR1_RAF1_MAPK3/1_9PHOS	Properties of MEKs, the kinases that phosphorylate and activate the extracellular signal-regulated kinases.,Zheng CF, Guan KL.,J Biol Chem.,8226933,1993,11,15
KSR1_RAF1_MAPK3/1_BIND	Protein phosphatase 2A positively regulates Ras signaling by dephosphorylating KSR1 and Raf-1 on critical 14-3-3 binding sites.,Ory S, Zhou M, Conrads TP, Veenstra TD, Morrison DK.,Curr Biol.,12932319,2003,8,19

KSR1_RAF1_MAPK3/1_BIND	Untying the regulation of the Raf-1 kinase.,Dhillon AS, Kolch W.,Arch Biochem Biophys.,12127063,2002,8,1
KSR1_RAF1_MAPK3/1_DISS	KSR: a MAPK scaffold of the Ras pathway?,Morrison DK.,J Cell Sci.,11309192,2001,5
KSR1_RAF1_MAPK3/1_DISS	Properties of MEKs, the kinases that phosphorylate and activate the extracellular signal-regulated kinases.,Zheng CF, Guan KL.,J Biol Chem.,8226933,1993,11,15
MAP2K1_MAPK3/1	Wiring diagrams of MAPK regulation by MEKK1, 2, and 3.,Uhlik MT, Abell AN, Cuevas BD, Nakamura K, Johnson GL.,Biochem Cell Biol.,15674433,2004,12
MAP2K3_MAPK11	Activation of NF-kappa B by nontypeable Hemophilus influenzae is mediated by toll-like receptor 2-TAK1-dependent NIK-IKK alpha /beta-I kappa B alpha and MKK3/6-p38 MAP kinase signaling pathways in epithelial cells.,Shuto T, Xu H, Wang B, Han J, Kai H, Gu XX, Murphy TF, Lim DJ, Li JD.,Proc Natl Acad Sci USA,11438700,2001,7,17
MAP2K3_MAPK11	Pro-inflammatory cytokines and environmental stress cause p38 mitogen-activated protein kinase activation by dual phosphorylation on tyrosine and threonine.,Raingeaud J, Gupta S, Rogers JS, Dickens M, Han J, Ulevitch RJ, Davis RJ.,J Biol Chem.,7535770,1995,3,31
MAP2K3_MAPK11	Wiring diagrams of MAPK regulation by MEKK1, 2, and 3.,Uhlik MT, Abell AN, Cuevas BD, Nakamura K, Johnson GL.,Biochem Cell Biol.,15674433,2004,12
MAP2K3_MAPK14	Activation of NF-kappa B by nontypeable Hemophilus influenzae is mediated by toll-like receptor 2-TAK1-dependent NIK-IKK alpha /beta-I kappa B alpha and MKK3/6-p38 MAP kinase signaling pathways in epithelial cells.,Shuto T, Xu H, Wang B, Han J, Kai H, Gu XX, Murphy TF, Lim DJ, Li JD.,Proc Natl Acad Sci USA,11438700,2001,7,17
MAP2K3_MAPK14	Pro-inflammatory cytokines and environmental stress cause p38 mitogen-activated protein kinase activation by dual phosphorylation on tyrosine and threonine.,Raingeaud J, Gupta S, Rogers JS, Dickens M, Han J, Ulevitch RJ, Davis RJ.,J Biol Chem.,7535770,1995,3,31
MAP2K3_MAPK14	Wiring diagrams of MAPK regulation by MEKK1, 2, and 3.,Uhlik MT, Abell AN, Cuevas BD, Nakamura K, Johnson GL.,Biochem Cell Biol.,15674433,2004,12
MAP2K3_TRAN	Nuclear export of the stress-activated protein kinase p38 mediated by its substrate MAPKAP kinase-2.,Ben-Levy R, Hooper S, Wilson R, Paterson HF, Marshall CJ.,Curr Biol.,9768359,1998,9,24
MAP2K3_TRAN2	Nuclear export of the stress-activated protein kinase p38 mediated by its substrate MAPKAP kinase-2.,Ben-Levy R, Hooper S, Wilson R, Paterson HF, Marshall CJ.,Curr Biol.,9768359,1998,9,24
MAP2K4_MAPK11	Wiring diagrams of MAPK regulation by MEKK1, 2, and 3.,Uhlik MT, Abell AN, Cuevas BD, Nakamura K, Johnson GL.,Biochem Cell Biol.,15674433,2004,12
MAP2K4_MAPK14	Wiring diagrams of MAPK regulation by MEKK1, 2, and 3.,Uhlik MT, Abell AN, Cuevas BD, Nakamura K, Johnson GL.,Biochem Cell Biol.,15674433,2004,12
MAP2K4_MAPK8	Mitogen-activated protein kinase kinase 4 (MKK4).,Cuenda A.,Int J Biochem Cell Biol.,10785355,2000,6
MAP2K4_MAPK8	Synergistic activation of SAPK1/JNK1 by two MAP kinase kinases in vitro.,Lawler S, Fleming Y, Goedert M, Cohen P.,Curr Biol.,9889102,1998,12,17
MAP2K4_MAPK8	The JNK signal transduction pathway.,Weston CR, Davis RJ.,Curr Opin Cell Biol.,17303404,2007,4
MAP2K4_MAPK8	Wiring diagrams of MAPK regulation by MEKK1, 2, and 3.,Uhlik MT, Abell AN, Cuevas BD, Nakamura K, Johnson GL.,Biochem Cell Biol.,15674433,2004,12
MAP2K6_MAPK11	MKK3- and MKK6-regulated gene expression is mediated by the p38 mitogen-activated protein kinase signal transduction pathway.,Raingeaud J, Whitmarsh AJ, Barrett T, Dérijard B, Davis RJ.,Mol Cell Biol.,8622669,1996,3

MAP2K6_MAPK14	MKK3- and MKK6-regulated gene expression is mediated by the p38 mitogen-activated protein kinase signal transduction pathway.,Raingeaud J, Whitmarsh AJ, Barrett T, Dérijard B, Davis RJ.,Mol Cell Biol.,8622669,1996,3
MAP2K6_TRAN	Nuclear export of the stress-activated protein kinase p38 mediated by its substrate MAPKAP kinase-2.,Ben-Levy R, Hooper S, Wilson R, Paterson HF, Marshall CJ.,Curr Biol.,9768359,1998,9,24
MAP2K6_TRAN2	Nuclear export of the stress-activated protein kinase p38 mediated by its substrate MAPKAP kinase-2.,Ben-Levy R, Hooper S, Wilson R, Paterson HF, Marshall CJ.,Curr Biol.,9768359,1998,9,24
MAP2K7_MAPK8	Mitogen-activated protein kinase kinase 4 (MKK4).,Cuenda A.,Int J Biochem Cell Biol.,10785355,2000,6
MAP2K7_MAPK8	Synergistic activation of SAPK1/JNK1 by two MAP kinase kinases in vitro.,Lawler S, Fleming Y, Goedert M, Cohen P.,Curr Biol.,9889102,1998,12,17
MAP2K7_MAPK8	The JNK signal transduction pathway.,Weston CR, Davis RJ.,Curr Opin Cell Biol.,17303404,2007,4
MAP2K7_MAPK8	Wiring diagrams of MAPK regulation by MEKK1, 2, and 3.,Uhlik MT, Abell AN, Cuevas BD, Nakamura K, Johnson GL.,Biochem Cell Biol.,15674433,2004,12
MAP3K14_IKK	Functional interactions of transforming growth factor beta-activated kinase 1 with IkappaB kinases to stimulate NF-kappaB activation.,Sakurai H, Miyoshi H, Toriumi W, Sugita T.,J Biol Chem.,10187861,1999,4,9
MAP3K14_IKK	NF-kappaB-inducing kinase activates IKK-alpha by phosphorylation of Ser-176.,Ling L, Cao Z, Goeddel DV.,Proc Natl Acad Sci USA,9520446,1998,3,31
MAP3K1_DEGR	Ubiquitylation of MEKK1 inhibits its phosphorylation of MKK1 and MKK4 and activation of the ERK1/2 and JNK pathways.,Witowsky JA, Johnson GL.,J Biol Chem.,12456688,2003,1,17
MAP3K1_IKK	Differential regulation of IkappaB kinase alpha and beta by two upstream kinases, NF-kappaB-inducing kinase and mitogen-activated protein kinase/ERK kinase kinase-1.,Nakano H, Shindo M, Sakon S, Nishinaka S, Mihara M, Yagita H, Okumura K.,Proc Natl Acad Sci USA,9520401,1998,3,31
MAP3K1_MAP2K1	MEKK1 phosphorylates MEK1 and MEK2 but does not cause activation of mitogen-activated protein kinase.,Xu S, Robbins D, Frost J, Dang A, Lange-Carter C, Cobb MH.,Proc Natl Acad Sci USA,7624324,1995,7,18
MAP3K1_MAP2K1	Raf kinase inhibitor protein interacts with NF-kappaB-inducing kinase and TAK1 and inhibits NF-kappaB activation.,Yeung KC, Rose DW, Dhillon AS, Yaros D, Gustafsson M, Chatterjee D, McFerran B, Wyche J, Kolch W, Sedivy JM.,Mol Cell Biol.,11585904,2001,11
MAP3K1_MAP2K1	Wiring diagrams of MAPK regulation by MEKK1, 2, and 3.,Uhlik MT, Abell AN, Cuevas BD, Nakamura K, Johnson GL.,Biochem Cell Biol.,15674433,2004,12
MAP3K1_MAP2K4	Wiring diagrams of MAPK regulation by MEKK1, 2, and 3.,Uhlik MT, Abell AN, Cuevas BD, Nakamura K, Johnson GL.,Biochem Cell Biol.,15674433,2004,12
MAP3K1_MAP2K7	Differential activation of two JNK activators, MKK7 and SEK1, by MKN28-derived nonreceptor serine/threonine kinase/mixed lineage kinase 2., Hirai S, Noda K, Moriguchi T, Nishida E, Yamashita A, Deyama T, Fukuyama K, Ohno S.,J Biol Chem.,9516438,1998,3,27
MAP3K1_PHOS	Signaling by proinflammatory cytokines: oligomerization of TRAF2 and TRAF6 is sufficient for JNK and IKK activation and target gene induction via an amino-terminal effector domain.,Baud V, Liu ZG, Bennett B, Suzuki N, Xia Y, Karin M.,Genes Dev.,10346818,1999,5,15
MAP3K1_UBIQ	Ubiquitylation of MEKK1 inhibits its phosphorylation of MKK1 and MKK4 and activation of the ERK1/2 and JNK pathways.,Witowsky JA, Johnson GL.,J Biol Chem.,12456688,2003,1,17

MAP3K3_MAP2K3	Characterization of the mitogen-activated protein kinase kinase 4 (MKK4)/c-Jun NH2-terminal kinase 1 and MKK3/p38 pathways regulated by MEK kinases 2 and 3. MEK kinase 3 activates MKK3 but does not cause activation of p38 kinase in vivo.,Deacon K, Blank JL.,J Biol Chem.,9162092,1997,5,30
MAP3K3_MAP2K3	Wiring diagrams of MAPK regulation by MEKK1, 2, and 3.,Uhlik MT, Abell AN, Cuevas BD, Nakamura K, Johnson GL.,Biochem Cell Biol.,15674433,2004,12
MAP3K3_MAP2K4	Characterization of the mitogen-activated protein kinase kinase 4 (MKK4)/c-Jun NH2-terminal kinase 1 and MKK3/p38 pathways regulated by MEK kinases 2 and 3. MEK kinase 3 activates MKK3 but does not cause activation of p38 kinase in vivo.,Deacon K, Blank JL.,J Biol Chem.,9162092,1997,5,30
MAP3K3_MAP2K4	Wiring diagrams of MAPK regulation by MEKK1, 2, and 3.,Uhlik MT, Abell AN, Cuevas BD, Nakamura K, Johnson GL.,Biochem Cell Biol.,15674433,2004,12
MAP3K3_MAP2K6	MEK kinase 3 directly activates MKK6 and MKK7, specific activators of the p38 and c-Jun NH2-terminal kinases.,Deacon K, Blank JL.,J Biol Chem.,10347227,1999,6,4
MAP3K3_MAP2K6	Wiring diagrams of MAPK regulation by MEKK1, 2, and 3.,Uhlik MT, Abell AN, Cuevas BD, Nakamura K, Johnson GL.,Biochem Cell Biol.,15674433,2004,12
MAP3K3_MAP2K7	MEK kinase 3 directly activates MKK6 and MKK7, specific activators of the p38 and c-Jun NH2-terminal kinases.,Deacon K, Blank JL.,J Biol Chem.,10347227,1999,6,4
MAP3K3_MAP2K7	Wiring diagrams of MAPK regulation by MEKK1, 2, and 3.,Uhlik MT, Abell AN, Cuevas BD, Nakamura K, Johnson GL.,Biochem Cell Biol.,15674433,2004,12
MAP3K5_MAP2K3	Induction of apoptosis by ASK1, a mammalian MAPKKK that activates SAPK/JNK and p38 signaling pathways.,Ichijo H, Nishida E, Irie K, ten Dijke P, Saitoh M, Moriguchi T, Takagi M, Matsumoto K, Miyazono K, Gotoh Y.,Science,8974401,1997,1,3
MAP3K5_MAP2K4	Induction of apoptosis by ASK1, a mammalian MAPKKK that activates SAPK/JNK and p38 signaling pathways.,Ichijo H, Nishida E, Irie K, ten Dijke P, Saitoh M, Moriguchi T, Takagi M, Matsumoto K, Miyazono K, Gotoh Y.,Science,8974401,1997,1,3
MAP3K5_MAP2K6	Induction of apoptosis by ASK1, a mammalian MAPKKK that activates SAPK/JNK and p38 signaling pathways.,Ichijo H, Nishida E, Irie K, ten Dijke P, Saitoh M, Moriguchi T, Takagi M, Matsumoto K, Miyazono K, Gotoh Y.,Science,8974401,1997,1,3
MAP3K5_TRXRED_BIND	Activation of apoptosis signal-regulating kinase 1 by the stress-induced activating phosphorylation of pre-formed oligomer.,Tobiume K, Saitoh M, Ichijo H.,J Cell Physiol.,11920685,2002,4
MAP3K5_TRXRED_BIND	Mammalian thioredoxin is a direct inhibitor of apoptosis signal-regulating kinase (ASK) 1.,Saitoh M, Nishitoh H, Fujii M, Takeda K, Tobiume K, Sawada Y, Kawabata M, Miyazono K, Ichijo H.,EMBO J.,9564042,1998,5,1
MAP3K5_TRXRED_DISS	Activation of apoptosis signal-regulating kinase 1 by the stress-induced activating phosphorylation of pre-formed oligomer.,Tobiume K, Saitoh M, Ichijo H.,J Cell Physiol.,11920685,2002,4
MAP3K5_TRXRED_DISS	Mammalian thioredoxin is a direct inhibitor of apoptosis signal-regulating kinase (ASK) 1.,Saitoh M, Nishitoh H, Fujii M, Takeda K, Tobiume K, Sawada Y, Kawabata M, Miyazono K, Ichijo H.,EMBO J.,9564042,1998,5,1
MAP3K7/IP123_BIND	TAB3, a new binding partner of the protein kinase TAK1.,Cheung PC, Nebreda AR, Cohen P.,Biochem J.,14670075,2004,2,15
MAP3K7/IP123_BIND	TAK1-dependent signaling requires functional interaction with TAB2/TAB3.,Besse A, Lamothe B, Campos AD, Webster WK, Maddineni U, Lin SC, Wu H, Darnay BG.,J Biol Chem.,17158449,2007,2,9

MAP3K7IP_TRAF6_3PHOS	Critical roles of threonine 187 phosphorylation in cellular stress-induced rapid and transient activation of transforming growth factor-beta-activated kinase 1 (TAK1) in a signaling complex containing TAK1-binding protein TAB1 and TAB2.,Singhirunnusorn P, Suzuki S, Kawasaki N, Saiki I, Sakurai H.,J Biol Chem.,15590691,2005,2,25
MAP3K7IP_TRAF6_UBIQ	Role of the TAB2-related protein TAB3 in IL-1 and TNF signaling.,Ishitani T, Takaesu G, Ninomiya-Tsuji J, Shibuya H, Gaynor RB, Matsumoto K.,EMBO J.,14633987,2003,12,1
MAP3K7IP_TRAF6_UBIQ	TAK1 is a ubiquitin-dependent kinase of MKK and IKK.,Wang C, Deng L, Hong M, Akkaraju GR, Inoue J, Chen ZJ.,Nature,11460167,2001,7,19
MAP3K7_IKK	Functional interactions of transforming growth factor beta-activated kinase 1 with IkappaB kinases to stimulate NF-kappaB activation.,Sakurai H, Miyoshi H, Toriumi W, Sugita T.,J Biol Chem.,10187861,1999,4,9
MAP3K7_IKK	TAK1 is a ubiquitin-dependent kinase of MKK and IKK.,Wang C, Deng L, Hong M, Akkaraju GR, Inoue J, Chen ZJ.,Nature,11460167,2001,7,19
MAP3K7_IKK	TAK1 mediates an activation signal from toll-like receptor(s) to nuclear factor-kappaB in lipopolysaccharide-stimulated macrophages.,Irie T, Muta T, Takeshige K.,FEBS Lett.,10675530,2000,2,11
MAP3K7_IKK	TAK1 regulates multiple protein kinase cascades activated by bacterial lipopolysaccharide.,Lee J, Mira-Arbibe L, Ulevitch RJ.,J Leukoc Biol.,11129660,2000,12
MAP3K7_MAP2K3	TGF-beta-activated kinase 1 and TAK1-binding protein 1 cooperate to mediate TGF-beta1-induced MKK3-p38 MAPK activation and stimulation of type I collagen.,Kim SI, Kwak JH, Zachariah M, He Y, Wang L, Choi ME.,Am J Physiol Renal Physiol.,17299140,2007,5
MAP3K7_MAP2K6	TAK1 is a ubiquitin-dependent kinase of MKK and IKK.,Wang C, Deng L, Hong M, Akkaraju GR, Inoue J, Chen ZJ.,Nature,11460167,2001,7,19
MAP3K7_MAP3K14	The kinase TAK1 can activate the NIK-I kappaB as well as the MAP kinase cascade in the IL-1 signalling pathway.,Ninomiya-Tsuji J, Kishimoto K, Hiyama A, Inoue J, Cao Z, Matsumoto K.,Nature,10094049,1999,3,18
MAP3K7_MAPK14	MAPKK-independent activation of p38alpha mediated by TAB1-dependent autophosphorylation of p38alpha.,Ge B, Gram H, Di Padova F, Huang B, New L, Ulevitch RJ, Luo Y, Han J.,Science,11847341,2002,2,15
MAP3K8(p58)_DEGR	Phosphorylation at Thr-290 regulates Tpl2 binding to NF-kappaB1/p105 and Tpl2 activation and degradation by lipopolysaccharide.,Cho J, Tsichlis PN.,Proc Natl Acad Sci USA,15699325,2005,2,15
MAP3K8(p58)_KSR2_BIND	Identification of a novel human kinase supporter of Ras (hKSR-2) that functions as a negative regulator of Cot (Tpl2) signaling.,Channavajhala PL, Wu L, Cuozzo JW, Hall JP, Liu W, Lin LL, Zhang Y.,J Biol Chem.,12975377,2003,11,21
MAP3K8_MAP2K1	Raf kinase inhibitor protein interacts with NF-kappaB-inducing kinase and TAK1 and inhibits NF-kappaB activation.,Yeung KC, Rose DW, Dhillon AS, Yaros D, Gustafsson M, Chatterjee D, McFerran B, Wyche J, Kolch W, Sedivy JM.,Mol Cell Biol.,11585904,2001,11
MAP3K8_MAP2K1	TNF-alpha induction by LPS is regulated posttranscriptionally via a Tpl2/ERK-dependent pathway.,Dumitru CD, Ceci JD, Tsatsanis C, Kontoyiannis D, Stamatakis K, Lin JH, Patriotis C, Jenkins NA, Copeland NG, Kollias G, Tsichlis PN.,Cell,11163183,2000,12,22
MAP3K8_MAP3K14	The proto-oncogene Cot kinase participates in CD3/CD28 induction of NF-kappaB acting through the NF-kappaB-inducing kinase and IkappaB kinases.,Lin X, Cunningham ET, Mu Y, Geleziunas R, Greene WC.,Immunity,10072079,1999,2
MAP3K8_NFKB_BIND	Phosphorylation at Thr-290 regulates Tpl2 binding to NF-kappaB1/p105 and Tpl2 activation and degradation by lipopolysaccharide.,Cho J, Tsichlis PN.,Proc Natl Acad Sci USA,15699325,2005,2,15

MAP3K8_NFKB_DISS	Phosphorylation at Thr-290 regulates Tpl2 binding to NF-kappaB1/p105 and Tpl2 activation and degradation by lipopolysaccharide.,Cho J, Tsichlis PN.,Proc Natl Acad Sci USA,15699325,2005,2,15
MAP3K8_NFKB_DISS	Tpl2 (tumor progression locus 2) phosphorylation at Thr290 is induced by lipopolysaccharide via an Ikappa-B Kinase-beta-dependent pathway and is required for Tpl2 activation by external signals.,Cho J, Melnick M, Solidakis GP, Tsichlis PN.,J Biol Chem.,15778223,2005,5,27
MAPK11_ATF2	MKK3- and MKK6-regulated gene expression is mediated by the p38 mitogen-activated protein kinase signal transduction pathway.,Raingeaud J, Whitmarsh AJ, Barrett T, Dérijard B, Davis RJ.,Mol Cell Biol.,8622669,1996,3
MAPK11_ATF2	Novel homologues of CSBP/p38 MAP kinase: activation, substrate specificity and sensitivity to inhibition by pyridinyl imidazoles.,Kumar S, McDonnell PC, Gum RJ, Hand AT, Lee JC, Young PR.,Biochem Biophys Res Commun.,9207191,1997,6,27
MAPK11_ELK1	MKK3- and MKK6-regulated gene expression is mediated by the p38 mitogen-activated protein kinase signal transduction pathway.,Raingeaud J, Whitmarsh AJ, Barrett T, Dérijard B, Davis RJ.,Mol Cell Biol.,8622669,1996,3
MAPK11_ELK1	The SRF accessory protein Elk-1 contains a growth factor-regulated transcriptional activation domain.,Marais R, Wynne J, Treisman R.,Cell,8386592,1993,4,23
MAPK11_MAPKAPK2	Nuclear export of the stress-activated protein kinase p38 mediated by its substrate MAPKAP kinase-2.,Ben-Levy R, Hooper S, Wilson R, Paterson HF, Marshall CJ.,Curr Biol.,9768359,1998,9,24
MAPK11_MAPKAPK2	Structure of mitogen-activated protein kinase-activated protein (MAPKAP) kinase 2 suggests a bifunctional switch that couples kinase activation with nuclear export.,Meng W, Swenson LL, Fitzgibbon MJ, Hayakawa K, Ter Haar E, Behrens AE, Fulghum JR, Lippke JA.,J Biol Chem.,12171911,2002,10,4
MAPK11_MAPKAPK2_BIND	Nuclear export of the stress-activated protein kinase p38 mediated by its substrate MAPKAP kinase-2.,Ben-Levy R, Hooper S, Wilson R, Paterson HF, Marshall CJ.,Curr Biol.,9768359,1998,9,24
MAPK11_MAPKAPK2_BIND	Structure of mitogen-activated protein kinase-activated protein (MAPKAP) kinase 2 suggests a bifunctional switch that couples kinase activation with nuclear export.,Meng W, Swenson LL, Fitzgibbon MJ, Hayakawa K, Ter Haar E, Behrens AE, Fulghum JR, Lippke JA.,J Biol Chem.,12171911,2002,10,4
MAPK11_MAPKAPK2_TRAN	Nuclear export of the stress-activated protein kinase p38 mediated by its substrate MAPKAP kinase-2.,Ben-Levy R, Hooper S, Wilson R, Paterson HF, Marshall CJ.,Curr Biol.,9768359,1998,9,24
MAPK11_MAPKAPK2_TRAN	Structure of mitogen-activated protein kinase-activated protein (MAPKAP) kinase 2 suggests a bifunctional switch that couples kinase activation with nuclear export.,Meng W, Swenson LL, Fitzgibbon MJ, Hayakawa K, Ter Haar E, Behrens AE, Fulghum JR, Lippke JA.,J Biol Chem.,12171911,2002,10,4
MAPK11_MBP	Novel homologues of CSBP/p38 MAP kinase: activation, substrate specificity and sensitivity to inhibition by pyridinyl imidazoles.,Kumar S, McDonnell PC, Gum RJ, Hand AT, Lee JC, Young PR.,Biochem Biophys Res Commun.,9207191,1997,6,27
MAPK11_MKNK1	MNK1, a new MAP kinase-activated protein kinase, isolated by a novel expression screening method for identifying protein kinase substrates.,Fukunaga R, Hunter T.,EMBO J.,9155018,1997,4,15
MAPK11_MKNK1	Mitogen-activated protein kinases activate the serine/threonine kinases Mnk1 and Mnk2.,Waskiewicz AJ, Flynn A, Proud CG, Cooper JA.,EMBO J.,9155017,1997,4,15
MAPK11_MSK1	MSK1 activity is controlled by multiple phosphorylation sites.,McCoy CE, Campbell DG, Deak M, Bloomberg GB, Arthur JS.,Biochem J.,15568999,2005,4,15

MAPK11_MSK1	Mitogen- and stress-activated protein kinase-1 (MSK1) is directly activated by MAPK and SAPK2/p38, and may mediate activation of CREB.,Deak M, Clifton AD, Lucocq LM, Alessi DR.,EMBO J.,9687510,1998,8,3
MAPK11_TRAN2	Nuclear export of the stress-activated protein kinase p38 mediated by its substrate MAPKAP kinase-2.,Ben-Levy R, Hooper S, Wilson R, Paterson HF, Marshall CJ.,Curr Biol.,9768359,1998,9,24
MAPK11_TRAN2	Structure of mitogen-activated protein kinase-activated protein (MAPKAP) kinase 2 suggests a bifunctional switch that couples kinase activation with nuclear export.,Meng W, Swenson LL, Fitzgibbon MJ, Hayakawa K, Ter Haar E, Behrens AE, Fulghum JR, Lippke JA.,J Biol Chem.,12171911,2002,10,4
MAPK14_ATF2	MKK3- and MKK6-regulated gene expression is mediated by the p38 mitogen-activated protein kinase signal transduction pathway.,Raingeaud J, Whitmarsh AJ, Barrett T, Dérijard B, Davis RJ.,Mol Cell Biol.,8622669,1996,3
MAPK14_ATF2	Novel homologues of CSBP/p38 MAP kinase: activation, substrate specificity and sensitivity to inhibition by pyridinyl imidazoles.,Kumar S, McDonnell PC, Gum RJ, Hand AT, Lee JC, Young PR.,Biochem Biophys Res Commun.,9207191,1997,6,27
MAPK14_ELK1	MKK3- and MKK6-regulated gene expression is mediated by the p38 mitogen-activated protein kinase signal transduction pathway.,Raingeaud J, Whitmarsh AJ, Barrett T, Dérijard B, Davis RJ.,Mol Cell Biol.,8622669,1996,3
MAPK14_ELK1	The SRF accessory protein Elk-1 contains a growth factor-regulated transcriptional activation domain.,Marais R, Wynne J, Treisman R.,Cell,8386592,1993,4,23
MAPK14_MAP3K7IP_TRAF6	Critical roles of threonine 187 phosphorylation in cellular stress-induced rapid and transient activation of transforming growth factor-beta-activated kinase 1 (TAK1) in a signaling complex containing TAK1-binding protein TAB1 and TAB2.,Singhirunnusorn P, Suzuki S, Kawasaki N, Saiki I, Sakurai H.,J Biol Chem.,15590691,2005,2,25
MAPK14_MAP3K7IP_TRAF6	Feedback control of the protein kinase TAK1 by SAPK2a/p38alpha.,Cheung PC, Campbell DG, Nebreda AR, Cohen P.,EMBO J.,14592977,2003,11,3
MAPK14_MAPKAPK2	Nuclear export of the stress-activated protein kinase p38 mediated by its substrate MAPKAP kinase-2.,Ben-Levy R, Hooper S, Wilson R, Paterson HF, Marshall CJ.,Curr Biol.,9768359,1998,9,24
MAPK14_MAPKAPK2	Structure of mitogen-activated protein kinase-activated protein (MAPKAP) kinase 2 suggests a bifunctional switch that couples kinase activation with nuclear export.,Meng W, Swenson LL, Fitzgibbon MJ, Hayakawa K, Ter Haar E, Behrens AE, Fulghum JR, Lippke JA.,J Biol Chem.,12171911,2002,10,4
MAPK14_MAPKAPK2_BIND	Nuclear export of the stress-activated protein kinase p38 mediated by its substrate MAPKAP kinase-2.,Ben-Levy R, Hooper S, Wilson R, Paterson HF, Marshall CJ.,Curr Biol.,9768359,1998,9,24
MAPK14_MAPKAPK2_BIND	Structure of mitogen-activated protein kinase-activated protein (MAPKAP) kinase 2 suggests a bifunctional switch that couples kinase activation with nuclear export.,Meng W, Swenson LL, Fitzgibbon MJ, Hayakawa K, Ter Haar E, Behrens AE, Fulghum JR, Lippke JA.,J Biol Chem.,12171911,2002,10,4
MAPK14_MAPKAPK2_TRAN	Nuclear export of the stress-activated protein kinase p38 mediated by its substrate MAPKAP kinase-2.,Ben-Levy R, Hooper S, Wilson R, Paterson HF, Marshall CJ.,Curr Biol.,9768359,1998,9,24
MAPK14_MAPKAPK2_TRAN	Structure of mitogen-activated protein kinase-activated protein (MAPKAP) kinase 2 suggests a bifunctional switch that couples kinase activation with nuclear export.,Meng W, Swenson LL, Fitzgibbon MJ, Hayakawa K, Ter Haar E, Behrens AE, Fulghum JR, Lippke JA.,J Biol Chem.,12171911,2002,10,4
MAPK14_MBP	Novel homologues of CSBP/p38 MAP kinase: activation, substrate specificity and sensitivity to inhibition by pyridinyl imidazoles.,Kumar S, McDonnell PC, Gum RJ, Hand AT, Lee JC, Young PR.,Biochem Biophys Res Commun.,9207191,1997,6,27

MAPK14_MKNK1	MNK1, a new MAP kinase-activated protein kinase, isolated by a novel expression screening method for identifying protein kinase substrates.,Fukunaga R, Hunter T.,EMBO J.,9155018,1997,4,15
MAPK14_MKNK1	Mitogen-activated protein kinases activate the serine/threonine kinases Mnk1 and Mnk2.,Waskiewicz AJ, Flynn A, Proud CG, Cooper JA.,EMBO J.,9155017,1997,4,15
MAPK14_MSK1	MSK1 activity is controlled by multiple phosphorylation sites.,McCoy CE, Campbell DG, Deak M, Bloomberg GB, Arthur JS.,Biochem J.,15568999,2005,4,15
MAPK14_MSK1	Mitogen- and stress-activated protein kinase-1 (MSK1) is directly activated by MAPK and SAPK2/p38, and may mediate activation of CREB.,Deak M, Clifton AD, Lucocq LM, Alessi DR.,EMBO J.,9687510,1998,8,3
MAPK14_TRAN2	Nuclear export of the stress-activated protein kinase p38 mediated by its substrate MAPKAP kinase-2.,Ben-Levy R, Hooper S, Wilson R, Paterson HF, Marshall CJ.,Curr Biol.,9768359,1998,9,24
MAPK14_TRAN2	Structure of mitogen-activated protein kinase-activated protein (MAPKAP) kinase 2 suggests a bifunctional switch that couples kinase activation with nuclear export.,Meng W, Swenson LL, Fitzgibbon MJ, Hayakawa K, Ter Haar E, Behrens AE, Fulghum JR, Lippke JA.,J Biol Chem.,12171911,2002,10,4
MAPK3/1_DEPH	MKP-1 (3CH134), an immediate early gene product, is a dual specificity phosphatase that dephosphorylates MAP kinase in vivo.,Sun H, Charles CH, Lau LF, Tonks NK.,Cell.,8221888,1993,11,5
MAPK3/1_DIME	Two co-existing mechanisms for nuclear import of MAP kinase: passive diffusion of a monomer and active transport of a dimer.,Adachi M, Fukuda M, Nishida E.,EMBO J.,10508167,1999,10,1
MAPK3/1_ELK1	The SRF accessory protein Elk-1 contains a growth factor-regulated transcriptional activation domain.,Marais R, Wynne J, Treisman R.,Cell,8386592,1993,4,23
MAPK3/1_MBP	Novel homologues of CSBP/p38 MAP kinase: activation, substrate specificity and sensitivity to inhibition by pyridinyl imidazoles.,Kumar S, McDonnell PC, Gum RJ, Hand AT, Lee JC, Young PR.,Biochem Biophys Res Commun.,9207191,1997,6,27
MAPK3/1_MKNK1	Mitogen-activated protein kinases activate the serine/threonine kinases Mnk1 and Mnk2.,Waskiewicz AJ, Flynn A, Proud CG, Cooper JA.,EMBO J.,9155017,1997,4,15
MAPK3/1_MKNK2	Mitogen-activated protein kinases activate the serine/threonine kinases Mnk1 and Mnk2.,Waskiewicz AJ, Flynn A, Proud CG, Cooper JA.,EMBO J.,9155017,1997,4,15
MAPK3/1_MSK1	MSK1 activity is controlled by multiple phosphorylation sites.,McCoy CE, Campbell DG, Deak M, Bloomberg GB, Arthur JS.,Biochem J.,15568999,2005,4,15
MAPK3/1_MSK1	Mitogen- and stress-activated protein kinase-1 (MSK1) is directly activated by MAPK and SAPK2/p38, and may mediate activation of CREB.,Deak M, Clifton AD, Lucocq LM, Alessi DR.,EMBO J.,9687510,1998,8,3
MAPK3/1_MYC	Transactivation of gene expression by Myc is inhibited by mutation at the phosphorylation sites Thr-58 and Ser-62.,Gupta S, Seth A, Davis RJ.,Proc Natl Acad Sci USA,8386367,1993,4,15
MAPK3/1_TRAN	Two co-existing mechanisms for nuclear import of MAP kinase: passive diffusion of a monomer and active transport of a dimer.,Adachi M, Fukuda M, Nishida E.,EMBO J.,10508167,1999,10,1
MAPK8_ATF2	The activation of c-Jun NH2-terminal kinase (JNK) by DNA-damaging agents serves to promote drug resistance via activating transcription factor 2 (ATF2)-dependent enhanced DNA repair.,Hayakawa J, Depatie C, Ohmichi M, Mercola D.,J Biol Chem.,12663670,2003,6,6

MAPK8_FOS	c-Fos phosphorylation induced by H2O2 prevents proteasomal degradation of c-Fos in cardiomyocytes,Coronella-Wood J, Terrand J, Sun H, Chen QM.,J Biol Chem,15136564,2004,8,6
MAPK8_JUN	The JNK signal transduction pathway.,Weston CR, Davis RJ.,Curr Opin Cell Biol.,17303404,2007,4
MAPK8_TRAN	A novel mechanism of JNK1 activation. Nuclear translocation and activation of JNK1 during ischemia and reperfusion.,Mizukami Y, Yoshioka K, Morimoto S, Yoshida K.,J Biol Chem.,9195981,1997,6,27
MAPK8_TRAN	Phosphorylation of JNK is involved in regulation of H(+)-induced c-Jun expression.,Shimokawa N, Qiu CH, Seki T, Dikic I, Koibuchi N.,Cell Signal.,15093613,2004,6
MAPK8_TRAN2	A novel mechanism of JNK1 activation. Nuclear translocation and activation of JNK1 during ischemia and reperfusion.,Mizukami Y, Yoshioka K, Morimoto S, Yoshida K.,J Biol Chem.,9195981,1997,6,27
MAPK8_TRAN2	Phosphorylation of JNK is involved in regulation of H(+)-induced c-Jun expression.,Shimokawa N, Qiu CH, Seki T, Dikic I, Koibuchi N.,Cell Signal.,15093613,2004,6
MAPKAPK2_2PHOS	Identification of novel phosphorylation sites required for activation of MAPKAP kinase-2.,Ben-Levy R, Leighton IA, Doza YN, Attwood P, Morrice N, Marshall CJ, Cohen P.,EMBO J.,8846784,1995,12,1
MAPKAPK2_2PHOS	Structure of mitogen-activated protein kinase-activated protein (MAPKAP) kinase 2 suggests a bifunctional switch that couples kinase activation with nuclear export.,Meng W, Swenson LL, Fitzgibbon MJ, Hayakawa K, Ter Haar E, Behrens AE, Fulghum JR, Lippke JA.,J Biol Chem.,12171911,2002,10,4
MAPKAPK2_CREB	FGF and stress regulate CREB and ATF-1 via a pathway involving p38 MAP kinase and MAPKAP kinase-2.,Tan Y, Rouse J, Zhang A, Cariati S, Cohen P, Comb MJ.,EMBO J.,8887554,1996,9,2
MAPKAPK2_HSP27	Identification of MAPKAP kinase 2 as a major enzyme responsible for the phosphorylation of the small mammalian heat shock proteins.,Stokoe D, Engel K, Campbell DG, Cohen P, Gaestel M.,FEBS Lett.,1332886,1992,11,30
MAPKAPK2_HSP27	Phosphorylation of the 27-kDa heat shock protein via p38 MAP kinase and MAPKAP kinase in smooth muscle.,Larsen JK, Yamboliev IA, Weber LA, Gerthoffer WT.,Am J Physiol.,9374719,1997,11
MD2_DIME	The role of disulfide bonds in the assembly and function of MD-2.,Mullen GE, Kennedy MN, Visintin A, Mazzoni A, Leifer CA, Davies DR, Segal DM.,Proc Natl Acad Sci USA.,12642668,2003,4,1
MKNK1_EIF4E	Mitogen-activated protein kinases activate the serine/threonine kinases Mnk1 and Mnk2.,Waskiewicz AJ, Flynn A, Proud CG, Cooper JA.,EMBO J.,9155017,1997,4,15
MSK1_5PHOS	MSK1 activity is controlled by multiple phosphorylation sites.,McCoy CE, Campbell DG, Deak M, Bloomberg GB, Arthur JS.,Biochem J.,15568999,2005,4,15
MSK1_CREB	MSK1 and MSK2 are required for the mitogen- and stress-induced phosphorylation of CREB and ATF1 in fibroblasts.,Wiggin GR, Soloaga A, Foster JM, Murray-Tait V, Cohen P, Arthur JS.,Mol Cell Biol.,11909979,2002,4
MSK1_HH3	MSK2 and MSK1 mediate the mitogen- and stress-induced phosphorylation of histone H3 and HMG-14.,Soloaga A, Thomson S, Wiggin GR, Rampersaud N, Dyson MH, Hazzalin CA, Mahadevan LC, Arthur JS.,EMBO J.,12773393,2003,6,2
MSK1_HH3	The nucleosomal response associated with immediate-early gene induction is mediated via alternative MAP kinase cascades: MSK1 as a potential histone H3/HMG-14 kinase.,Thomson S, Clayton AL, Hazzalin CA, Rose S, Barratt MJ, Mahadevan LC.,EMBO J.,10469656,1999,9,1

MSK1_HMG14	MSK2 and MSK1 mediate the mitogen- and stress-induced phosphorylation of histone H3 and HMG-14.,Soloaga A, Thomson S, Wiggin GR, Rampersaud N, Dyson MH, Hazzalin CA, Mahadevan LC, Arthur JS.,EMBO J.,12773393,2003,6,2
MSK1_HMG14	The nucleosomal response associated with immediate-early gene induction is mediated via alternative MAP kinase cascades: MSK1 as a potential histone H3/HMG-14 kinase.,Thomson S, Clayton AL, Hazzalin CA, Rose S, Barratt MJ, Mahadevan LC.,EMBO J.,10469656,1999,9,1
MSK1_NFKB	Post-translational modifications regulating the activity and function of the nuclear factor kappa B pathway.,Perkins ND.,Oncogene.,17072324,2006,10,30
MSK1_NFKB	Transcriptional activation of the NF-kappaB p65 subunit by mitogen- and stress-activated protein kinase-1 (MSK1).,Vermeulen L, De Wilde G, Van Damme P, Vanden Berghe W, Haegeman G.,EMBO J.,12628924,2003,3,17
MSK1_NFKB_P1K	Post-translational modifications regulating the activity and function of the nuclear factor kappa B pathway.,Perkins ND.,Oncogene.,17072324,2006,10,30
MSK1_NFKB_P1K	Transcriptional activation of the NF-kappaB p65 subunit by mitogen- and stress-activated protein kinase-1 (MSK1).,Vermeulen L, De Wilde G, Van Damme P, Vanden Berghe W, Haegeman G.,EMBO J.,12628924,2003,3,17
MYD88_DIME	Inhibition of MyD88 dimerization and recruitment of IRAK1 and IRAK4 by a novel peptidomimetic compound.,Loiarro M, Capolunghi F, Fantò N, Gallo G, Campo S, Arseni B, Carsetti R, Carminati P, De Santis R, Ruggiero V, Sette C.,J Leukoc Biol.,17548806,2007,6,4
MYD88_DIME	Structural complementarity of Toll/interleukin-1 receptor domains in Toll-like receptors and the adaptors Mal and MyD88.,Dunne A, Ejdeback M, Ludidi PL, O'Neill LA, Gay NJ.,J Biol Chem.,12888566,2003,10,17
MYD88_IRAK_TOLLIP_2PHOS	Sequential autophosphorylation steps in the interleukin-1 receptor-associated kinase-1 regulate its availability as an adapter in interleukin-1 signaling.,Kollewe C, Mackensen AC, Neumann D, Knop J, Cao P, Li S, Wesche H, Martin MU.,J Biol Chem.,14625308,2004,2,13
MYD88_IRAK_TOLLIP_BIND	Recruitment of IRAK to the interleukin 1 receptor complex requires interleukin 1 receptor accessory protein.,Huang J, Gao X, Li S, Cao Z.,Proc Natl Acad Sci USA.,9371760,1997,11,25
MYD88_IRAK_TOLLIP_BIND	Tollip, a new component of the IL-1RI pathway, links IRAK to the IL-1 receptor.,Burns K, Clatworthy J, Martin L, Martinon F, Plumpton C, Maschera B, Lewis A, Ray K, Tschopp J, Volpe F.,Nat Cell Biol.,10854325,2000,6
MYD88_IRAK_TOLLIP_DISS	Negative regulation of toll-like receptor-mediated signaling by Tollip.,Zhang G, Ghosh S.,J Biol Chem.,11751856,2002,3,1
MYD88_IRAK_TOLLIP_DISS	Sequential autophosphorylation steps in the interleukin-1 receptor-associated kinase-1 regulate its availability as an adapter in interleukin-1 signaling.,Kollewe C, Mackensen AC, Neumann D, Knop J, Cao P, Li S, Wesche H, Martin MU.,J Biol Chem.,14625308,2004,2,13
MYD88_IRAK_TOLLIP_PHOS	IRAK-4: a novel member of the IRAK family with the properties of an IRAK-kinase.,Li S, Strelow A, Fontana EJ, Wesche H.,Proc Natl Acad Sci USA,11960013,2002,4,16
MYD88_IRAK_TOLLIP_PHOS	Sequential autophosphorylation steps in the interleukin-1 receptor-associated kinase-1 regulate its availability as an adapter in interleukin-1 signaling.,Kollewe C, Mackensen AC, Neumann D, Knop J, Cao P, Li S, Wesche H, Martin MU.,J Biol Chem.,14625308,2004,2,13
MYD88_IRF7_BIND	Interferon-alpha induction through Toll-like receptors involves a direct interaction of IRF7 with MyD88 and TRAF6.,Kawai T, Sato S, Ishii KJ, Coban C, Hemmi H, Yamamoto M, Terai K, Matsuda M, Inoue J, Uematsu S, Takeuchi O, Akira S.,Nat Immunol.,15361868,2004,10
MYD88_IRF7_BIND	TLR signaling.,Kawai T, Akira S.,Cell Death Differ.,16410796,2006,5

MYD88_IRF7_BIND2	Interferon-alpha induction through Toll-like receptors involves a direct interaction of IRF7 with MyD88 and TRAF6.,Kawai T, Sato S, Ishii KJ, Coban C, Hemmi H, Yamamoto M, Terai K, Matsuda M, Inoue J, Uematsu S, Takeuchi O, Akira S.,Nat Immunol.,15361868,2004,10
MYD88_IRF7_BIND2	TLR signaling.,Kawai T, Akira S.,Cell Death Differ.,16410796,2006,5
MYD88_IRF7_BIND3	Interferon-alpha induction through Toll-like receptors involves a direct interaction of IRF7 with MyD88 and TRAF6.,Kawai T, Sato S, Ishii KJ, Coban C, Hemmi H, Yamamoto M, Terai K, Matsuda M, Inoue J, Uematsu S, Takeuchi O, Akira S.,Nat Immunol.,15361868,2004,10
MYD88_IRF7_BIND3	TLR signaling.,Kawai T, Akira S.,Cell Death Differ.,16410796,2006,5
MYD88_IRF7_DISS	IRF family of transcription factors as regulators of host defense.,Taniguchi T, Ogasawara K, Takaoka A, Tanaka N.,Annu Rev Immunol.,11244049,2001
MYD88_IRF7_DISS	Triggering the interferon antiviral response through an IKK-related pathway.,Sharma S, tenOever BR, Grandvaux N, Zhou GP, Lin R, Hiscott J.,Science.,12702806,2003,5,16
MYD88_IRF7_DISS2	IRF family of transcription factors as regulators of host defense.,Taniguchi T, Ogasawara K, Takaoka A, Tanaka N.,Annu Rev Immunol.,11244049,2001
MYD88_IRF7_DISS2	Triggering the interferon antiviral response through an IKK-related pathway.,Sharma S, tenOever BR, Grandvaux N, Zhou GP, Lin R, Hiscott J.,Science.,12702806,2003,5,16
MYD88_MYD88S_BIND	Modulation of Toll-interleukin 1 receptor mediated signaling.,Li X, Qin J.,J Mol Med.,15662540,2005,4
MYD88_MYD88S_BIND	Negative regulation of toll-like receptor-mediated immune responses.,Liew FY, Xu D, Brint EK, O'Neill LA.,Nat Rev Immunol.,15928677,2005,6
NFKB(p105)_BTRCP2_BIND	Shared pathways of IkappaB kinase-induced SCF(betaTrCP)-mediated ubiquitination and degradation for the NF-kappaB precursor p105 and IkappaBalpha.,Heissmeyer V, Krappmann D, Hatada EN, Scheidereit C.,Mol Cell Biol.,11158290,2001,2
NFKB(p105)_BTRCP2_BIND	Signaling to NF-kappaB.,Hayden MS, Ghosh S.,Genes Dev.,15371334,2004,9,15
NFKB(p105)_DEGR	Shared pathways of IkappaB kinase-induced SCF(betaTrCP)-mediated ubiquitination and degradation for the NF-kappaB precursor p105 and IkappaBalpha.,Heissmeyer V, Krappmann D, Hatada EN, Scheidereit C.,Mol Cell Biol.,11158290,2001,2
NFKB(p105)_DEGR	Signaling to NF-kappaB.,Hayden MS, Ghosh S.,Genes Dev.,15371334,2004,9,15
NFKB(p105)_DEGR2	Dual effects of IkappaB kinase beta-mediated phosphorylation on p105 Fate: SCF(beta-TrCP)-dependent degradation and SCF(beta-TrCP)-independent processing.,Cohen S, Achbert-Weiner H, Ciechanover A.,Mol Cell Biol.,14673179,2004,1
NFKB(p105)_DEGR2	Signaling to NF-kappaB.,Hayden MS, Ghosh S.,Genes Dev.,15371334,2004,9,15
NFKB(p105)_DEGR3	Signaling to NF-kappaB.,Hayden MS, Ghosh S.,Genes Dev.,15371334,2004,9,15
NFKB(p105)_PROC	Dual effects of IkappaB kinase beta-mediated phosphorylation on p105 Fate: SCF(beta-TrCP)-dependent degradation and SCF(beta-TrCP)-independent processing.,Cohen S, Achbert-Weiner H, Ciechanover A.,Mol Cell Biol.,14673179,2004,1
NFKB(p105)_PROC	Signaling to NF-kappaB.,Hayden MS, Ghosh S.,Genes Dev.,15371334,2004,9,15
NFKB(p105)_PROC2	Signaling to NF-kappaB.,Hayden MS, Ghosh S.,Genes Dev.,15371334,2004,9,15

NFKB(p105)_UBCH5A_BIND	Dual effects of IkappaB kinase beta-mediated phosphorylation on p105 Fate: SCF(beta-TrCP)-dependent degradation and SCF(beta-TrCP)-independent processing.,Cohen S, Achbert-Weiner H, Ciechanover A.,Mol Cell Biol.,14673179,2004,1
NFKB(p105)_UBCH5A_BIND	Signaling to NF-kappaB.,Hayden MS, Ghosh S.,Genes Dev.,15371334,2004,9,15
NFKB(p105)_UBIQ	Shared pathways of IkappaB kinase-induced SCF(betaTrCP)-mediated ubiquitination and degradation for the NF-kappaB precursor p105 and IkappaBalpha.,Heissmeyer V, Krappmann D, Hatada EN, Scheidereit C.,Mol Cell Biol.,11158290,2001,2
NFKB(p105)_UBIQ	Signaling to NF-kappaB.,Hayden MS, Ghosh S.,Genes Dev.,15371334,2004,9,15
NFKB(p105)_UBIQ2	Dual effects of IkappaB kinase beta-mediated phosphorylation on p105 Fate: SCF(beta-TrCP)-dependent degradation and SCF(beta-TrCP)-independent processing.,Cohen S, Achbert-Weiner H, Ciechanover A.,Mol Cell Biol.,14673179,2004,1
NFKB(p105)_UBIQ2	Signaling to NF-kappaB.,Hayden MS, Ghosh S.,Genes Dev.,15371334,2004,9,15
NFKB(p105/p50)_BIND	Processing of p105 is inhibited by docking of p50 active subunits to the ankyrin repeat domain, and inhibition is alleviated by signaling via the carboxyl-terminal phosphorylation/ ubiquitin-ligase binding domain.,Cohen S, Orian A, Ciechanover A.,J Biol Chem.,11350967,2001,7,20
NFKB(p105/p50)_BIND	Regulation of NFKB1 proteins by the candidate oncoprotein BCL-3: generation of NF-kappaB homodimers from the cytoplasmic pool of p50-p105 and nuclear translocation.,Watanabe N, Iwamura T, Shinoda T, Fujita T.,EMBO J.,9218802,1997,6,16
NFKB(p105/p50)_BIND	Shared pathways of IkappaB kinase-induced SCF(betaTrCP)-mediated ubiquitination and degradation for the NF-kappaB precursor p105 and IkappaBalpha.,Heissmeyer V, Krappmann D, Hatada EN, Scheidereit C.,Mol Cell Biol.,11158290,2001,2
NFKB(p105/p50)_DISS	Regulation of NFKB1 proteins by the candidate oncoprotein BCL-3: generation of NF-kappaB homodimers from the cytoplasmic pool of p50-p105 and nuclear translocation.,Watanabe N, Iwamura T, Shinoda T, Fujita T.,EMBO J.,9218802,1997,6,16
NFKB(p50)_BCL3_BIND	NF-kappaB p105 is a target of IkappaB kinases and controls signal induction of Bcl-3-p50 complexes.,Heissmeyer V, Krappmann D, Wulczyn FG, Scheidereit C.,EMBO J.,10469655,1999,9,1
NFKB(p50)_BCL3_BIND	Regulation of NFKB1 proteins by the candidate oncoprotein BCL-3: generation of NF-kappaB homodimers from the cytoplasmic pool of p50-p105 and nuclear translocation.,Watanabe N, Iwamura T, Shinoda T, Fujita T.,EMBO J.,9218802,1997,6,16
NFKB(p50)_BCL3_TRAN	NF-kappaB p105 is a target of IkappaB kinases and controls signal induction of Bcl-3-p50 complexes.,Heissmeyer V, Krappmann D, Wulczyn FG, Scheidereit C.,EMBO J.,10469655,1999,9,1
NFKB(p50)_BCL3_TRAN	Regulation of NFKB1 proteins by the candidate oncoprotein BCL-3: generation of NF-kappaB homodimers from the cytoplasmic pool of p50-p105 and nuclear translocation.,Watanabe N, Iwamura T, Shinoda T, Fujita T.,EMBO J.,9218802,1997,6,16
NFKB(p50)_IKBZ_BIND	Regulation of Toll/IL-1-receptor-mediated gene expression by the inducible nuclear protein IkappaBzeta.,Yamamoto M, Yamazaki S, Uematsu S, Sato S, Hemmi H, Hoshino K, Kaisho T, Kuwata H, Takeuchi O, Takeshige K, Saitoh T, Yamaoka S, Yamamoto N, Yamamoto S, Muta T, Takeda K, Akira S.,Nature,15241416,2004,7,8

NFKB(p50)_IKBZ_BIND	Stimulus-specific induction of a novel nuclear factor-kappaB regulator, IkappaB-zeta, via Toll/Interleukin-1 receptor is mediated by mRNA stabilization.,Yamazaki S, Muta T, Matsuo S, Takeshige K.,J Biol Chem.,15522867,2005,1,14
NFKB(p50)_IMPA3_BIND	NF-{kappa}B is transported into the nucleus by importin {alpha}3 and importin {alpha}4.,Fagerlund R, Kinnunen L, Köhler M, Julkunen I, Melén K.,J Biol Chem.,15677444,2005,4,22
NFKB(p50)_IMPA3_DISS	NF-{kappa}B is transported into the nucleus by importin {alpha}3 and importin {alpha}4.,Fagerlund R, Kinnunen L, Köhler M, Julkunen I, Melén K.,J Biol Chem.,15677444,2005,4,22
NFKB(p50)_IMPA3_TRAN	NF-{kappa}B is transported into the nucleus by importin {alpha}3 and importin {alpha}4.,Fagerlund R, Kinnunen L, Köhler M, Julkunen I, Melén K.,J Biol Chem.,15677444,2005,4,22
NFKB(p50)_KB_SITE_BIND	DNA binding of repressor nuclear factor-kappaB p50/p50 depends on phosphorylation of Ser337 by the protein kinase A catalytic subunit.,Guan H, Hou S, Ricciardi RP.,J Biol Chem.,15642694,2005,3,18
NFKB(p50)_KB_SITE_BIND	Molecular control of immune/inflammatory responses: interactions between nuclear factor-kappa B and steroid receptor-signaling pathways.,McKay LI, Cidlowski JA.,Endocr Rev.,10453354,1999,8
NFKB(p50)_KB_SITE_DISS	Molecular control of immune/inflammatory responses: interactions between nuclear factor-kappa B and steroid receptor-signaling pathways.,McKay LI, Cidlowski JA.,Endocr Rev.,10453354,1999,8
NFKB(p50/p65)_CBP-5P3A_BIND	Post-activation turn-off of NF-kappa B-dependent transcription is regulated by acetylation of p65.,Kiernan R, Brès V, Ng RW, Coudart MP, El Messaoudi S, Sardet C, Jin DY, Emiliani S, Benkirane M.,J Biol Chem.,12419806,2003,1,24
NFKB(p50/p65)_CBP-5P3A_BIND	Post-translational modifications regulating the activity and function of the nuclear factor kappa B pathway.,Perkins ND.,Oncogene.,17072324,2006,10,30
NFKB(p50/p65)_CBP-5P_BIND	Acetylation of RelA at discrete sites regulates distinct nuclear functions of NF-kappaB.,Chen LF, Mu Y, Greene WC.,EMBO J.,12456660,2002,12,2
NFKB(p50/p65)_CBP-5P_BIND	Post-translational modifications regulating the activity and function of the nuclear factor kappa B pathway.,Perkins ND.,Oncogene.,17072324,2006,10,30
NFKB(p65)_DEGR	Regulation of NF-kappaB signaling by Pin1-dependent prolyl isomerization and ubiquitin-mediated proteolysis of p65/RelA.,Ryo A, Suizu F, Yoshida Y, Perrem K, Liou YC, Wulf G, Rottapel R, Yamaoka S, Lu KP.,Mol Cell.,14690596,2003,12
NFKB(p65)_DEGR2	Degradation of promoter-bound p65/RelA is essential for the prompt termination of the nuclear factor kappaB response.,Saccani S, Marazzi I, Beg AA, Natoli G.,J Exp Med.,15226358,2004,7,5
NFKB(p65)_SOCS1_BIND	Regulation of NF-kappaB signaling by Pin1-dependent prolyl isomerization and ubiquitin-mediated proteolysis of p65/RelA.,Ryo A, Suizu F, Yoshida Y, Perrem K, Liou YC, Wulf G, Rottapel R, Yamaoka S, Lu KP.,Mol Cell.,14690596,2003,12
NFKB(p65)_UBIQ	Degradation of promoter-bound p65/RelA is essential for the prompt termination of the nuclear factor kappaB response.,Saccani S, Marazzi I, Beg AA, Natoli G.,J Exp Med.,15226358,2004,7,5
NFKB_2ACTL	Post-activation turn-off of NF-kappa B-dependent transcription is regulated by acetylation of p65.,Kiernan R, Brès V, Ng RW, Coudart MP, El Messaoudi S, Sardet C, Jin DY, Emiliani S, Benkirane M.,J Biol Chem.,12419806,2003,1,24
NFKB_2ACTL	Post-translational modifications regulating the activity and function of the nuclear factor kappa B pathway.,Perkins ND.,Oncogene.,17072324,2006,10,30
NFKB_3ACTL	Acetylation of RelA at discrete sites regulates distinct nuclear functions of NF-kappaB.,Chen LF, Mu Y, Greene WC.,EMBO J.,12456660,2002,12,2
NFKB_3ACTL	Post-translational modifications regulating the activity and function of the nuclear factor kappa B pathway.,Perkins ND.,Oncogene.,17072324,2006,10,30

NFKB_3DACL	Acetylation of RelA at discrete sites regulates distinct nuclear functions of NF-kappaB.,Chen LF, Mu Y, Greene WC.,EMBO J.,12456660,2002,12,2
NFKB_3DACL	Duration of nuclear NF-kappaB action regulated by reversible acetylation.,Chen Lf , Fischle W, Verdin E, Greene WC.,Science.,11533489,2001,8,31
NFKB_3DACL	Post-translational modifications regulating the activity and function of the nuclear factor kappa B pathway.,Perkins ND.,Oncogene.,17072324,2006,10,30
NFKB_5ACTL	Post-activation turn-off of NF-kappa B-dependent transcription is regulated by acetylation of p65.,Kiernan R, Brès V, Ng RW, Coudart MP, El Messaoudi S, Sardet C, Jin DY, Emiliani S, Benkirane M.,J Biol Chem.,12419806,2003,1,24
NFKB_5ACTL	Post-translational modifications regulating the activity and function of the nuclear factor kappa B pathway.,Perkins ND.,Oncogene.,17072324,2006,10,30
NFKB_5DACL	Post-activation turn-off of NF-kappa B-dependent transcription is regulated by acetylation of p65.,Kiernan R, Brès V, Ng RW, Coudart MP, El Messaoudi S, Sardet C, Jin DY, Emiliani S, Benkirane M.,J Biol Chem.,12419806,2003,1,24
NFKB_5DACL	Post-translational modifications regulating the activity and function of the nuclear factor kappa B pathway.,Perkins ND.,Oncogene.,17072324,2006,10,30
NFKB_CBP_KB_BIND	Acetylation of RelA at discrete sites regulates distinct nuclear functions of NF-kappaB.,Chen LF, Mu Y, Greene WC.,EMBO J.,12456660,2002,12,2
NFKB_CBP_KB_BIND	CREB-binding protein/p300 are transcriptional coactivators of p65.,Gerritsen ME, Williams AJ, Neish AS, Moore S, Shi Y, Collins T.,Proc Natl Acad Sci USA.,9096323,1997,4,1
NFKB_CBP_KB_BIND	Signaling to NF-kappaB.,Hayden MS, Ghosh S.,Genes Dev.,15371334,2004,9,15
NFKB_CBP_KB_BIND	Toll-like receptor-mediated cytokine production is differentially regulated by glycogen synthase kinase 3.,Martin M, Rehani K, Jope RS, Michalek SM.,Nat Immunol.,16007092,2005,8
NFKB_CBP_KB_DISS	IKKalpha limits macrophage NF-kappaB activation and contributes to the resolution of inflammation.,Lawrence T, Bebien M, Liu GY, Nizet V, Karin M.,Nature.,15858576,2005,4,28
NFKB_IKBA_BIND	Signaling to NF-kappaB.,Hayden MS, Ghosh S.,Genes Dev.,15371334,2004,9,15
NFKB_IKBA_BIND2	Distinct functional properties of IkappaB alpha and IkappaB beta.,Tran K, Merika M, Thanos D.,Mol Cell Biol.,9271416,1997,9
NFKB_IKBA_BIND2	Nuclear localization of I kappa B alpha promotes active transport of NF-kappa B from the nucleus to the cytoplasm.,Arenzana-Seisdedos F, Turpin P, Rodriguez M, Thomas D, Hay RT, Virelizier JL, Dargemont C.,J Cell Sci.,9057089,1997,2
NFKB_IKBA_BIND2	Post-activation turn-off of NF-kappa B-dependent transcription is regulated by acetylation of p65.,Kiernan R, Brès V, Ng RW, Coudart MP, El Messaoudi S, Sardet C, Jin DY, Emiliani S, Benkirane M.,J Biol Chem.,12419806,2003,1,24
NFKB_IKBA_BIND2	Post-translational modifications regulating the activity and function of the nuclear factor kappa B pathway.,Perkins ND.,Oncogene.,17072324,2006,10,30
NFKB_IKBA_BIND3	Nuclear localization of I kappa B alpha promotes active transport of NF-kappa B from the nucleus to the cytoplasm.,Arenzana-Seisdedos F, Turpin P, Rodriguez M, Thomas D, Hay RT, Virelizier JL, Dargemont C.,J Cell Sci.,9057089,1997,2
NFKB_IKBA_BIND3	Post-activation turn-off of NF-kappa B-dependent transcription is regulated by acetylation of p65.,Kiernan R, Brès V, Ng RW, Coudart MP, El Messaoudi S, Sardet C, Jin DY, Emiliani S, Benkirane M.,J Biol Chem.,12419806,2003,1,24
NFKB_IKBA_BIND3	Post-translational modifications regulating the activity and function of the nuclear factor kappa B pathway.,Perkins ND.,Oncogene.,17072324,2006,10,30
NFKB_IKBA_BIND4	Nuclear localization of I kappa B alpha promotes active transport of NF-kappa B from the nucleus to the cytoplasm.,Arenzana-Seisdedos F, Turpin P, Rodriguez M, Thomas D, Hay RT, Virelizier JL, Dargemont C.,J Cell Sci.,9057089,1997,2

NFKB_IKBA_BIND4	Post-activation turn-off of NF-kappa B-dependent transcription is regulated by acetylation of p65.,Kiernan R, Brès V, Ng RW, Coudart MP, El Messaoudi S, Sardet C, Jin DY, Emiliani S, Benkirane M.,J Biol Chem.,12419806,2003,1,24
NFKB_IKBA_BIND4	Post-translational modifications regulating the activity and function of the nuclear factor kappa B pathway.,Perkins ND.,Oncogene.,17072324,2006,10,30
NFKB_IKBA_BTRCP1_BIND	Impaired degradation of inhibitory subunit of NF-kappa B (I kappa B) and beta-catenin as a result of targeted disruption of the beta-TrCP1 gene.,Nakayama K, Hatakeyama S, Maruyama S, Kikuchi A, Onoé K, Good RA, Nakayama KI.,Proc Natl Acad Sci USA,12843402,2003,7,22
NFKB_IKBA_BTRCP1_BIND	Missing pieces in the NF-kappaB puzzle.,Ghosh S, Karin M.,Cell.,11983155,2002,4
NFKB_IKBA_BTRCP2_BIND	Inducible degradation of IkappaBalpha by the proteasome requires interaction with the F-box protein h-betaTrCP.,Kroll M, Margottin F, Kohl A, Renard P, Durand H, Concordet JP, Bachelerie F, Arenzana-Seisdedos F, Benarous R.,J Biol Chem.,10075690,1999,3,19
NFKB_IKBA_BTRCP2_BIND	Inhibition of NF-kappa-B cellular function via specific targeting of the I-kappa-B-ubiquitin ligase.,Yaron A, Gonen H, Alkalay I, Hatzubai A, Jung S, Beyth S, Mercurio F, Manning AM, Ciechanover A, Ben-Neriah Y.,EMBO J.,9351830,1998,11,3
NFKB_IKBA_BTRCP2_BIND	Signal-induced ubiquitination of IkappaBalpha by the F-box protein Slimb/beta-TrCP.,Spencer E, Jiang J, Chen ZJ.,Genes Dev.,9990853,1999,2,1
NFKB_IKBA_BTRCP2_BIND	beta-TrCP mediates the signal-induced ubiquitination of IkappaBbeta.,Wu C, Ghosh S.,J Biol Chem.,10514424,1999,10,15
NFKB_IKBA_DISS	Missing pieces in the NF-kappaB puzzle.,Ghosh S, Karin M.,Cell.,11983155,2002,4
NFKB_IKBA_DISS	Signaling to NF-kappaB.,Hayden MS, Ghosh S.,Genes Dev.,15371334,2004,9,15
NFKB_IKBA_TRAN	Missing pieces in the NF-kappaB puzzle.,Ghosh S, Karin M.,Cell.,11983155,2002,4
NFKB_IKBA_TRAN2	Missing pieces in the NF-kappaB puzzle.,Ghosh S, Karin M.,Cell.,11983155,2002,4
NFKB_IKBA_TRAN3	Missing pieces in the NF-kappaB puzzle.,Ghosh S, Karin M.,Cell.,11983155,2002,4
NFKB_IKBA_TRAN3	Nuclear localization of I kappa B alpha promotes active transport of NF-kappa B from the nucleus to the cytoplasm.,Arenzana-Seisdedos F, Turpin P, Rodriguez M, Thomas D, Hay RT, Virelizier JL, Dargemont C.,J Cell Sci.,9057089,1997,2
NFKB_IKBA_TRAN3	Post-activation turn-off of NF-kappa B-dependent transcription is regulated by acetylation of p65.,Kiernan R, Brès V, Ng RW, Coudart MP, El Messaoudi S, Sardet C, Jin DY, Emiliani S, Benkirane M.,J Biol Chem.,12419806,2003,1,24
NFKB_IKBA_TRAN3	Post-translational modifications regulating the activity and function of the nuclear factor kappa B pathway.,Perkins ND.,Oncogene.,17072324,2006,10,30
NFKB_IKBA_TRAN4	Nuclear localization of I kappa B alpha promotes active transport of NF-kappa B from the nucleus to the cytoplasm.,Arenzana-Seisdedos F, Turpin P, Rodriguez M, Thomas D, Hay RT, Virelizier JL, Dargemont C.,J Cell Sci.,9057089,1997,2
NFKB_IKBA_TRAN4	Post-activation turn-off of NF-kappa B-dependent transcription is regulated by acetylation of p65.,Kiernan R, Brès V, Ng RW, Coudart MP, El Messaoudi S, Sardet C, Jin DY, Emiliani S, Benkirane M.,J Biol Chem.,12419806,2003,1,24
NFKB_IKBA_TRAN4	Post-translational modifications regulating the activity and function of the nuclear factor kappa B pathway.,Perkins ND.,Oncogene.,17072324,2006,10,30
NFKB_IKBA_TRAN5	Nuclear localization of I kappa B alpha promotes active transport of NF-kappa B from the nucleus to the cytoplasm.,Arenzana-Seisdedos F, Turpin P, Rodriguez M, Thomas D, Hay RT, Virelizier JL, Dargemont C.,J Cell Sci.,9057089,1997,2

NFKB_IKBA_TRAN5	Post-activation turn-off of NF-kappa B-dependent transcription is regulated by acetylation of p65.,Kiernan R, Brès V, Ng RW, Coudart MP, El Messaoudi S, Sardet C, Jin DY, Emiliani S, Benkirane M.,J Biol Chem.,12419806,2003,1,24
NFKB_IKBA_TRAN5	Post-translational modifications regulating the activity and function of the nuclear factor kappa B pathway.,Perkins ND.,Oncogene.,17072324,2006,10,30
NFKB_IKBA_UBCH5_BIND	Inducible degradation of IkappaBalpha by the proteasome requires interaction with the F-box protein h-betaTrCP.,Kroll M, Margottin F, Kohl A, Renard P, Durand H, Concordet JP, Bachelerie F, Arenzana-Seisdedos F, Benarous R.,J Biol Chem.,10075690,1999,3,19
NFKB_IKBA_UBCH5_BIND	Inhibition of NF-kappa-B cellular function via specific targeting of the I-kappa-B-ubiquitin ligase.,Yaron A, Gonen H, Alkalay I, Hatzubai A, Jung S, Beyth S, Mercurio F, Manning AM, Ciechanover A, Ben-Neriah Y.,EMBO J.,9351830,1998,11,3
NFKB_IKBA_UBCH5_BIND	Signal-induced ubiquitination of IkappaBalpha by the F-box protein Slimb/beta-TrCP.,Spencer E, Jiang J, Chen ZJ.,Genes Dev.,9990853,1999,2,1
NFKB_IKBA_UBCH5_BIND	beta-TrCP mediates the signal-induced ubiquitination of IkappaBbeta.,Wu C, Ghosh S.,J Biol Chem.,10514424,1999,10,15
NFKB_IKBA_UBCH5_BIND2	Missing pieces in the NF-kappaB puzzle.,Ghosh S, Karin M.,Cell.,11983155,2002,4
NFKB_IKBB_BIND	Signaling to NF-kappaB.,Hayden MS, Ghosh S.,Genes Dev.,15371334,2004,9,15
NFKB_IKBB_BIND2	Basal phosphorylation of the PEST domain in the I(kappa)B(beta) regulates its functional interaction with the c-rel proto-oncogene product.,Chu ZL, McKinsey TA, Liu L, Qi X, Ballard DW.,Mol Cell Biol.,8887627,1996,9
NFKB_IKBB_BIND2	Distinct functional properties of IkappaB alpha and IkappaB beta.,Tran K, Merika M, Thanos D.,Mol Cell Biol.,9271416,1997,9
NFKB_IKBB_BTRCP2_BIND	Inducible degradation of IkappaBalpha by the proteasome requires interaction with the F-box protein h-betaTrCP.,Kroll M, Margottin F, Kohl A, Renard P, Durand H, Concordet JP, Bachelerie F, Arenzana-Seisdedos F, Benarous R.,J Biol Chem.,10075690,1999,3,19
NFKB_IKBB_BTRCP2_BIND	Signal-induced ubiquitination of IkappaBalpha by the F-box protein Slimb/beta-TrCP.,Spencer E, Jiang J, Chen ZJ.,Genes Dev.,9990853,1999,2,1
NFKB_IKBB_BTRCP2_BIND	Signaling to NF-kappaB.,Hayden MS, Ghosh S.,Genes Dev.,15371334,2004,9,15
NFKB_IKBB_BTRCP2_BIND	beta-TrCP mediates the signal-induced ubiquitination of IkappaBbeta.,Wu C, Ghosh S.,J Biol Chem.,10514424,1999,10,15
NFKB_IKBB_DISS	Missing pieces in the NF-kappaB puzzle.,Ghosh S, Karin M.,Cell.,11983155,2002,4
NFKB_IKBB_DISS	Signaling to NF-kappaB.,Hayden MS, Ghosh S.,Genes Dev.,15371334,2004,9,15
NFKB_IKBB_TRAN	Basal phosphorylation of the PEST domain in the I(kappa)B(beta) regulates its functional interaction with the c-rel proto-oncogene product.,Chu ZL, McKinsey TA, Liu L, Qi X, Ballard DW.,Mol Cell Biol.,8887627,1996,9
NFKB_IKBB_TRAN	Distinct functional properties of IkappaB alpha and IkappaB beta.,Tran K, Merika M, Thanos D.,Mol Cell Biol.,9271416,1997,9
NFKB_P1_PHOS	Essential role of RelA Ser311 phosphorylation by zetaPKC in NF-kappaB transcriptional activation.,Duran A, Diaz-Meco MT, Moscat J.,EMBO J.,12881425,2003,8,1
NFKB_P1_PHOS	IKKbeta phosphorylates p65 at S468 in transactivaton domain 2.,Schwabe RF, Sakurai H. ,FASEB J.,16046471,2005,10
NFKB_P1_PHOS	Inducible phosphorylation of NF-kappa B p65 at serine 468 by T cell costimulation is mediated by IKK epsilon.,Mattioli I, Geng H, Sebald A, Hodel M, Bucher C, Kracht M, Schmitz ML.,J Biol Chem.,16407239,2006,3,10

NFKB_P1_PHOS	Phosphorylation of serine 468 by GSK-3beta negatively regulates basal p65 NF-kappaB activity.,Buss H, Dörrie A, Schmitz ML, Frank R, Livingstone M, Resch K, Kracht M.,J Biol Chem.,15465828,2004,11,26
NFKB_P1_PHOS	Post-translational modifications regulating the activity and function of the nuclear factor kappa B pathway.,Perkins ND.,Oncogene.,17072324,2006,10,30
NFKB_P1_PHOS	TOO LONG: See PubMed ID,Buss H, Dörrie A, Schmitz ML, Hoffmann E, Resch K, Kracht M.,J Biol Chem.,15489227,2004,12,31
NFKB_P1_PHOS	Tumor necrosis factor alpha-induced phosphorylation of RelA/p65 on Ser529 is controlled by casein kinase II.,Wang D, Westerheide SD, Hanson JL, Baldwin AS. ,J Biol Chem.,10938077,2000,10,20
NFKB_P2_PHOS	IKKbeta phosphorylates p65 at S468 in transactivaton domain 2.,Schwabe RF, Sakurai H. ,FASEB J.,16046471,2005,10
NFKB_P2_PHOS	Inducible phosphorylation of NF-kappa B p65 at serine 468 by T cell costimulation is mediated by IKK epsilon.,Mattioli I, Geng H, Sebald A, Hodel M, Bucher C, Kracht M, Schmitz ML.,J Biol Chem.,16407239,2006,3,10
NFKB_P2_PHOS	Phosphorylation of NF-kappa B p65 by PKA stimulates transcriptional activity by promoting a novel bivalent interaction with the coactivator CBP/p300.,Zhong H, Voll RE, Ghosh S.,Mol Cell.,9660950,1998,4
NFKB_P2_PHOS	Phosphorylation of serine 468 by GSK-3beta negatively regulates basal p65 NF-kappaB activity.,Buss H, Dörrie A, Schmitz ML, Frank R, Livingstone M, Resch K, Kracht M.,J Biol Chem.,15465828,2004,11,26
NFKB_P2_PHOS	Post-translational modifications regulating the activity and function of the nuclear factor kappa B pathway.,Perkins ND.,Oncogene.,17072324,2006,10,30
NFKB_P2_PHOS	TOO LONG: See PubMed ID,Buss H, Dörrie A, Schmitz ML, Hoffmann E, Resch K, Kracht M.,J Biol Chem.,15489227,2004,12,31
NFKB_P2_PHOS	The transcriptional activity of NF-kappaB is regulated by the IkappaB-associated PKAc subunit through a cyclic AMP-independent mechanism.,Zhong H, SuYang H, Erdjument-Bromage H, Tempst P, Ghosh S.,Cell.,9150141,1997,5,2
NFKB_P2_PHOS	Transcriptional activation of the NF-kappaB p65 subunit by mitogen- and stress-activated protein kinase-1 (MSK1).,Vermeulen L, De Wilde G, Van Damme P, Vanden Berghe W, Haegeman G.,EMBO J.,12628924,2003,3,17
NFKB_P2_PHOS	Tumor necrosis factor alpha-induced phosphorylation of RelA/p65 on Ser529 is controlled by casein kinase II.,Wang D, Westerheide SD, Hanson JL, Baldwin AS. ,J Biol Chem.,10938077,2000,10,20
NFKB_P3_PHOS	Essential role of RelA Ser311 phosphorylation by zetaPKC in NF-kappaB transcriptional activation.,Duran A, Diaz-Meco MT, Moscat J.,EMBO J.,12881425,2003,8,1
NFKB_P3_PHOS	IKKbeta phosphorylates p65 at S468 in transactivaton domain 2.,Schwabe RF, Sakurai H. ,FASEB J.,16046471,2005,10
NFKB_P3_PHOS	Phosphorylation of NF-kappa B p65 by PKA stimulates transcriptional activity by promoting a novel bivalent interaction with the coactivator CBP/p300.,Zhong H, Voll RE, Ghosh S.,Mol Cell.,9660950,1998,4
NFKB_P3_PHOS	Post-translational modifications regulating the activity and function of the nuclear factor kappa B pathway.,Perkins ND.,Oncogene.,17072324,2006,10,30
NFKB_P3_PHOS	TOO LONG: See PubMed ID,Buss H, Dörrie A, Schmitz ML, Hoffmann E, Resch K, Kracht M.,J Biol Chem.,15489227,2004,12,31
NFKB_P3_PHOS	The transcriptional activity of NF-kappaB is regulated by the IkappaB-associated PKAc subunit through a cyclic AMP-independent mechanism.,Zhong H, SuYang H, Erdjument-Bromage H, Tempst P, Ghosh S.,Cell.,9150141,1997,5,2
NFKB_P3_PHOS	Transcriptional activation of the NF-kappaB p65 subunit by mitogen- and stress-activated protein kinase-1 (MSK1).,Vermeulen L, De Wilde G, Van Damme P, Vanden Berghe W, Haegeman G.,EMBO J.,12628924,2003,3,17

NFKB_P3_PHOS	Tumor necrosis factor alpha-induced phosphorylation of RelA/p65 on Ser529 is controlled by casein kinase II.,Wang D, Westerheide SD, Hanson JL, Baldwin AS. ,J Biol Chem.,10938077,2000,10,20
NFKB_P4_PHOS	Essential role of RelA Ser311 phosphorylation by zetaPKC in NF-kappaB transcriptional activation.,Duran A, Diaz-Meco MT, Moscat J.,EMBO J.,12881425,2003,8,1
NFKB_P4_PHOS	IKKbeta phosphorylates p65 at S468 in transactivaton domain 2.,Schwabe RF, Sakurai H. ,FASEB J.,16046471,2005,10
NFKB_P4_PHOS	Inducible phosphorylation of NF-kappa B p65 at serine 468 by T cell costimulation is mediated by IKK epsilon.,Mattioli I, Geng H, Sebald A, Hodel M, Bucher C, Kracht M, Schmitz ML.,J Biol Chem.,16407239,2006,3,10
NFKB_P4_PHOS	Phosphorylation of NF-kappa B p65 by PKA stimulates transcriptional activity by promoting a novel bivalent interaction with the coactivator CBP/p300.,Zhong H, Voll RE, Ghosh S.,Mol Cell.,9660950,1998,4
NFKB_P4_PHOS	Phosphorylation of serine 468 by GSK-3beta negatively regulates basal p65 NF-kappaB activity.,Buss H, Dörrie A, Schmitz ML, Frank R, Livingstone M, Resch K, Kracht M.,J Biol Chem.,15465828,2004,11,26
NFKB_P4_PHOS	Post-translational modifications regulating the activity and function of the nuclear factor kappa B pathway.,Perkins ND.,Oncogene.,17072324,2006,10,30
NFKB_P4_PHOS	TOO LONG: See PubMed ID,Buss H, Dörrie A, Schmitz ML, Hoffmann E, Resch K, Kracht M.,J Biol Chem.,15489227,2004,12,31
NFKB_P4_PHOS	The transcriptional activity of NF-kappaB is regulated by the IkappaB-associated PKAc subunit through a cyclic AMP-independent mechanism.,Zhong H, SuYang H, Erdjument-Bromage H, Tempst P, Ghosh S.,Cell.,9150141,1997,5,2
NFKB_P4_PHOS	Transcriptional activation of the NF-kappaB p65 subunit by mitogen- and stress-activated protein kinase-1 (MSK1).,Vermeulen L, De Wilde G, Van Damme P, Vanden Berghe W, Haegeman G.,EMBO J.,12628924,2003,3,17
NFKB_P5_PHOS	Essential role of RelA Ser311 phosphorylation by zetaPKC in NF-kappaB transcriptional activation.,Duran A, Diaz-Meco MT, Moscat J.,EMBO J.,12881425,2003,8,1
NFKB_P5_PHOS	IKKbeta phosphorylates p65 at S468 in transactivaton domain 2.,Schwabe RF, Sakurai H. ,FASEB J.,16046471,2005,10
NFKB_P5_PHOS	Inducible phosphorylation of NF-kappa B p65 at serine 468 by T cell costimulation is mediated by IKK epsilon.,Mattioli I, Geng H, Sebald A, Hodel M, Bucher C, Kracht M, Schmitz ML.,J Biol Chem.,16407239,2006,3,10
NFKB_P5_PHOS	Phosphorylation of NF-kappa B p65 by PKA stimulates transcriptional activity by promoting a novel bivalent interaction with the coactivator CBP/p300.,Zhong H, Voll RE, Ghosh S.,Mol Cell.,9660950,1998,4
NFKB_P5_PHOS	Phosphorylation of serine 468 by GSK-3beta negatively regulates basal p65 NF-kappaB activity.,Buss H, Dörrie A, Schmitz ML, Frank R, Livingstone M, Resch K, Kracht M.,J Biol Chem.,15465828,2004,11,26
NFKB_P5_PHOS	Post-translational modifications regulating the activity and function of the nuclear factor kappa B pathway.,Perkins ND.,Oncogene.,17072324,2006,10,30
NFKB_P5_PHOS	The transcriptional activity of NF-kappaB is regulated by the IkappaB-associated PKAc subunit through a cyclic AMP-independent mechanism.,Zhong H, SuYang H, Erdjument-Bromage H, Tempst P, Ghosh S.,Cell.,9150141,1997,5,2
NFKB_P5_PHOS	Transcriptional activation of the NF-kappaB p65 subunit by mitogen- and stress-activated protein kinase-1 (MSK1).,Vermeulen L, De Wilde G, Van Damme P, Vanden Berghe W, Haegeman G.,EMBO J.,12628924,2003,3,17
NFKB_P5_PHOS	Tumor necrosis factor alpha-induced phosphorylation of RelA/p65 on Ser529 is controlled by casein kinase II.,Wang D, Westerheide SD, Hanson JL, Baldwin AS. ,J Biol Chem.,10938077,2000,10,20

NFKB_PKAC_DISS	Signaling to NF-kappaB.,Hayden MS, Ghosh S.,Genes Dev.,15371334,2004,9,15
NFKB_PKAC_IKBA_BIND	The transcriptional activity of NF-kappaB is regulated by the IkappaB-associated PKAc subunit through a cyclic AMP-independent mechanism.,Zhong H, SuYang H, Erdjument-Bromage H, Tempst P, Ghosh S.,Cell.,9150141,1997,5,2
NFKB_PKAC_IKBA_DISS	The transcriptional activity of NF-kappaB is regulated by the IkappaB-associated PKAc subunit through a cyclic AMP-independent mechanism.,Zhong H, SuYang H, Erdjument-Bromage H, Tempst P, Ghosh S.,Cell.,9150141,1997,5,2
NFKB_PKAC_IKBB_BIND	The transcriptional activity of NF-kappaB is regulated by the IkappaB-associated PKAc subunit through a cyclic AMP-independent mechanism.,Zhong H, SuYang H, Erdjument-Bromage H, Tempst P, Ghosh S.,Cell.,9150141,1997,5,2
NFKB_PKAC_IKBB_DISS	The transcriptional activity of NF-kappaB is regulated by the IkappaB-associated PKAc subunit through a cyclic AMP-independent mechanism.,Zhong H, SuYang H, Erdjument-Bromage H, Tempst P, Ghosh S.,Cell.,9150141,1997,5,2
NFKB_TRAN	Post-translational modifications regulating the activity and function of the nuclear factor kappa B pathway.,Perkins ND.,Oncogene.,17072324,2006,10,30
NFKB_TRAN	TLR signaling.,Kawai T, Akira S.,Cell Death Differ.,16410796,2006,5
NFKB_TRAN	TOO LONG: See pubmed ID,Sato S, Sugiyama M, Yamamoto M, Watanabe Y, Kawai T, Takeda K, Akira S.,J Immunol.,14530355,2003,10,15
NFKB_TRAN	Toll-like receptor function and signaling.,Kaisho T, Akira S.,J Allergy Clin Immunol.,16675322,2006,5
NFKB_TRAN	Toll-like receptor signaling.,Akira S.,J Biol Chem.,12893815,2003,10,3
NOD1P_BIND	An essential role for NOD1 in host recognition of bacterial peptidoglycan containing diaminopimelic acid.,Chamaillard M, Hashimoto M, Horie Y, Masumoto J, Qiu S, Saab L, Ogura Y, Kawasaki A, Fukase K, Kusumoto S, Valvano MA, Foster SJ, Mak TW, Nuñez G, Inohara N.,Nat Immunol.,12796777,2003,7
NOD1P_BIND	CATERPILLER: a novel gene family important in immunity, cell death, and diseases.,Ting JP, Davis BK.,Annu Rev Immunol.,15771576,2005
NOD2P_BIND	CATERPILLER: a novel gene family important in immunity, cell death, and diseases.,Ting JP, Davis BK.,Annu Rev Immunol.,15771576,2005
NOD2P_BIND	Nod2 is a general sensor of peptidoglycan through muramyl dipeptide (MDP) detection.,Girardin SE, Boneca IG, Viala J, Chamaillard M, Labigne A, Thomas G, Philpott DJ, Sansonetti PJ.,J Biol Chem.,12527755,2003,3,14
NSF_EEA1_RAB5_BIND	Oligomeric complexes link Rab5 effectors with NSF and drive membrane fusion via interactions between EEA1 and syntaxin 13.,McBride HM, Rybin V, Murphy C, Giner A, Teasdale R, Zerial M.,Cell.,10458612,1999,8,6
NSF_EEA1_RAB5_BIND	Signal transduction pathways mediated by the interaction of CpG DNA with Toll-like receptor 9.,Takeshita F, Gursel I, Ishii KJ, Suzuki K, Gursel M, Klinman DM.,Semin Immunol.,14751759,2004,2
NSF_STXN13_BIND	Oligomeric complexes link Rab5 effectors with NSF and drive membrane fusion via interactions between EEA1 and syntaxin 13.,McBride HM, Rybin V, Murphy C, Giner A, Teasdale R, Zerial M.,Cell.,10458612,1999,8,6
PAK1_CDC42_BIND	p21-activated kinases: three more join the Pak.,Jaffer ZM, Chernoff J.,Int J Biochem Cell Biol.,11950587,2002,7
PAK1_MAP2K1	Cross-cascade activation of ERKs and ternary complex factors by Rho family proteins.,Frost JA, Steen H, Shapiro P, Lewis T, Ahn N, Shaw PE, Cobb MH.,EMBO J.,9351825,1997,11,3
PAK1_MAP2K1-2	Cross-cascade activation of ERKs and ternary complex factors by Rho family proteins.,Frost JA, Steen H, Shapiro P, Lewis T, Ahn N, Shaw PE, Cobb MH.,EMBO J.,9351825,1997,11,3

PAK1_PHOS	Identification of a central phosphorylation site in p21-activated kinase regulating autoinhibition and kinase activity.,Zenke FT, King CC, Bohl BP, Bokoch GM.,J Biol Chem.,10551809,1999,11,12
PAK1_PHOS	p21-activated kinases: three more join the Pak.,Jaffer ZM, Chernoff J.,Int J Biochem Cell Biol.,11950587,2002,7
PAK1_RAC1_BIND	p21-activated kinases: three more join the Pak.,Jaffer ZM, Chernoff J.,Int J Biochem Cell Biol.,11950587,2002,7
PAK1_SRCK_RAF1	Phosphorylation of Raf-1 by p21-activated kinase 1 and Src regulates Raf-1 autoinhibition.,Tran NH, Frost JA.,J Biol Chem.,12551923,2003,3,28
PAK1_SRCK_RAF1	Positive and negative regulation of Raf kinase activity and function by phosphorylation.,Chong H, Lee J, Guan KL.,EMBO J.,11447113,2001,7,16
PAK1_SRCK_RAF1	Untying the regulation of the Raf-1 kinase.,Dhillon AS, Kolch W.,Arch Biochem Biophys.,12127063,2002,8,1
PAK1_SRCK_RAF1-2	Phosphorylation of Raf-1 by p21-activated kinase 1 and Src regulates Raf-1 autoinhibition.,Tran NH, Frost JA.,J Biol Chem.,12551923,2003,3,28
PAK1_SRCK_RAF1-2	Positive and negative regulation of Raf kinase activity and function by phosphorylation.,Chong H, Lee J, Guan KL.,EMBO J.,11447113,2001,7,16
PAK1_SRCK_RAF1-2	Untying the regulation of the Raf-1 kinase.,Dhillon AS, Kolch W.,Arch Biochem Biophys.,12127063,2002,8,1
PCASP8_PROC	Autoproteolytic activation of pro-caspases by oligomerization.,Yang X, Chang HY, Baltimore D.,Mol Cell.,9659928,1998,1
PCASP8_PROC	Membrane oligomerization and cleavage activates the caspase-8 (FLICE/MACHalpha1) death signal.,Martin DA, Siegel RM, Zheng L, Lenardo MJ.,J Biol Chem.,9468483,1998,2,20
PCASP8_PROC	The apoptotic signaling pathway activated by Toll-like receptor-2.,Aliprantis AO, Yang RB, Weiss DS, Godowski P, Zychlinsky A.,EMBO J.,10880445,2000,7,3
PCASP9_NOD1P_BIND	CATERPILLER: a novel gene family important in immunity, cell death, and diseases.,Ting JP, Davis BK.,Annu Rev Immunol.,15771576,2005
PCASP9_NOD1P_BIND	Nod1, an Apaf-1-like activator of caspase-9 and nuclear factor-kappaB.,Inohara N, Koseki T, del Peso L, Hu Y, Yee C, Chen S, Carrio R, Merino J, Liu D, Ni J, Núñez G.,J Biol Chem.,10329646,1999,5,21
PCASP9_NOD2P_BIND	CATERPILLER: a novel gene family important in immunity, cell death, and diseases.,Ting JP, Davis BK.,Annu Rev Immunol.,15771576,2005
PCASP9_NOD2P_BIND	Nod1, an Apaf-1-like activator of caspase-9 and nuclear factor-kappaB.,Inohara N, Koseki T, del Peso L, Hu Y, Yee C, Chen S, Carrio R, Merino J, Liu D, Ni J, Núñez G.,J Biol Chem.,10329646,1999,5,21
PCASP9_PROC	CATERPILLER: a novel gene family important in immunity, cell death, and diseases.,Ting JP, Davis BK.,Annu Rev Immunol.,15771576,2005
PCASP9_PROC	Nod1, an Apaf-1-like activator of caspase-9 and nuclear factor-kappaB.,Inohara N, Koseki T, del Peso L, Hu Y, Yee C, Chen S, Carrio R, Merino J, Liu D, Ni J, Núñez G.,J Biol Chem.,10329646,1999,5,21
PCASP9_PROC2	CATERPILLER: a novel gene family important in immunity, cell death, and diseases.,Ting JP, Davis BK.,Annu Rev Immunol.,15771576,2005
PCASP9_PROC2	Nod1, an Apaf-1-like activator of caspase-9 and nuclear factor-kappaB.,Inohara N, Koseki T, del Peso L, Hu Y, Yee C, Chen S, Carrio R, Merino J, Liu D, Ni J, Núñez G.,J Biol Chem.,10329646,1999,5,21
PDK1_AKT	Unravelling the activation mechanisms of protein kinase B/Akt.,Scheid MP, Woodgett JR.,FEBS Lett.,12829245,2003,7,3
PDK1_PHOS	Phosphorylation of Ser-241 is essential for the activity of 3-phosphoinositide-dependent protein kinase-1: identification of five sites of phosphorylation in vivo.,Casamayor A, Morrice NA, Alessi DR.,Biochem J.,10455013,1999,9,1
PDK1_PHOS	Translocation of PDK-1 to the plasma membrane is important in allowing PDK-1 to activate protein kinase B.,Anderson KE, Coadwell J, Stephens LR, Hawkins PT.,Curr Biol.,9637919,1998,6,4

PDK1_PKCA	Intracellular signalling: PDK1--a kinase at the hub of things.,Belham C, Wu S, Avruch J.,Curr Biol.,10021376,1999,2,11
PDK1_PKCA	New insights into the regulation of protein kinase C and novel phorbol ester receptors.,Ron D, Kazanietz MG.,FASEB J.,10506570,1999,10
PDK1_PKCA	Regulation of protein kinase C.,Newton AC.,Curr Opin Cell Biol.,9069266,1997,4
PDK1_PKCB2	Intracellular signalling: PDK1--a kinase at the hub of things.,Belham C, Wu S, Avruch J.,Curr Biol.,10021376,1999,2,11
PDK1_PKCB2	New insights into the regulation of protein kinase C and novel phorbol ester receptors.,Ron D, Kazanietz MG.,FASEB J.,10506570,1999,10
PDK1_PKCB2	Regulation of protein kinase C.,Newton AC.,Curr Opin Cell Biol.,9069266,1997,4
PDK1_PKCD	Intracellular signalling: PDK1--a kinase at the hub of things.,Belham C, Wu S, Avruch J.,Curr Biol.,10021376,1999,2,11
PDK1_PKCD	New insights into the regulation of protein kinase C and novel phorbol ester receptors.,Ron D, Kazanietz MG.,FASEB J.,10506570,1999,10
PDK1_PKCD	Regulation of protein kinase C.,Newton AC.,Curr Opin Cell Biol.,9069266,1997,4
PDK1_PKCZ	Further evidence that 3-phosphoinositide-dependent protein kinase-1 (PDK1) is required for the stability and phosphorylation of protein kinase C (PKC) isoforms.,Balendran A, Hare GR, Kieloch A, Williams MR, Alessi DR.,FEBS Lett.,11078882,2000,11,10
PDK1_PKCZ	Protein kinase C isotypes controlled by phosphoinositide 3-kinase through the protein kinase PDK1.,Le Good JA, Ziegler WH, Parekh DB, Alessi DR, Cohen P, Parker PJ.,Science.,9748166,1998,9,25
PDK1_pail345p_hs_BIND	Translocation of PDK-1 to the plasma membrane is important in allowing PDK-1 to activate protein kinase B.,Anderson KE, Coadwell J, Stephens LR, Hawkins PT.,Curr Biol.,9637919,1998,6,4
PHOX_GTP_BIND	Phagocyte NADPH oxidase: a multicomponent enzyme essential for host defenses.,El-Benna J, Dang PM, Gougerot-Pocidalo MA, Elbim C.,Arch Immunol Ther Exp (Warsz).,15995580,2005,6
PHOX_GTP_BIND	Rac binding to p67(phox). Structural basis for interactions of the Rac1 effector region and insert region with components of the respiratory burst oxidase.,Nisimoto Y, Freeman JL, Motalebi SA, Hirshberg M, Lambeth JD.,J Biol Chem.,9228059,1997,7,25
PHOX_GTP_BIND	Rho GTPases and their effector proteins.,Bishop AL, Hall A.,Biochem J.,10816416,2000,6,1
PHOX_GTP_BIND2	Phagocyte NADPH oxidase: a multicomponent enzyme essential for host defenses.,El-Benna J, Dang PM, Gougerot-Pocidalo MA, Elbim C.,Arch Immunol Ther Exp (Warsz).,15995580,2005,6
PHOX_GTP_BIND2	Rac binding to p67(phox). Structural basis for interactions of the Rac1 effector region and insert region with components of the respiratory burst oxidase.,Nisimoto Y, Freeman JL, Motalebi SA, Hirshberg M, Lambeth JD.,J Biol Chem.,9228059,1997,7,25
PHOX_GTP_BIND2	Rho GTPases and their effector proteins.,Bishop AL, Hall A.,Biochem J.,10816416,2000,6,1
PI34P5K_pail34p_hs	Phosphoinositides and signal transduction.,Toker A.,Cell Mol Life Sci.,12088277,2002,5
PI34P5K_pail34p_hs	Synthesis and function of 3-phosphorylated inositol lipids.,Vanhaesebroeck B, Leevers SJ, Ahmadi K, Timms J, Katso R, Driscoll PC, Woscholski R, Parker PJ, Waterfield MD.,Annu Rev Biochem.,11395417,2001
PI3K1A_PHOS	Activation of phosphoinositide 3-kinase activity by Cdc42Hs binding to p85.,Zheng Y, Bagrodia S, Cerione RA.,J Biol Chem.,8034624,1994,7,22

PI3K1A_PHOS	Novel roles of TLR3 tyrosine phosphorylation and PI3 kinase in double-stranded RNA signaling.,Sarkar SN, Peters KL, Elco CP, Sakamoto S, Pal S, Sen GC.,Nat Struct Mol Biol.,15502848,2004,11
PI3K1A_PHOS	Phosphatidylinositol 3-kinase is recruited to a specific site in the activated IL-1 receptor I.,Marmiroli S, Bavelloni A, Faenza I, Sirri A, Ognibene A, Cenni V, Tsukada J, Koyama Y, Ruzzene M, Ferri A, Auron PE, Toker A, Maraldi NM.,FEBS Lett.,9821957,1998,10,30
PI3K1A_PHOS	Rac GTPase interacts specifically with phosphatidylinositol 3-kinase.,Bokoch GM, Vlahos CJ, Wang Y, Knaus UG, Traynor-Kaplan AE.,Biochem J.,8645157,1996,5,1
PI3K1A_PHOS	Rho family GTPases bind to phosphoinositide kinases.,Tolias KF, Cantley LC, Carpenter CL.,J Biol Chem.,7629060,1995,7,28
PI3K1A_PHOS	The IL-1 receptor accessory protein is essential for PI 3-kinase recruitment and activation.,Reddy SA, Lin YF, Huang HJ, Samanta AK, Liao WS.,Biochem Biophys Res Commun.,15044087,2004,4,16
PI3K1A_pail45p_hs	Phosphoinositides and signal transduction.,Toker A.,Cell Mol Life Sci.,12088277,2002,5
PI3K1A_pail45p_hs	Synthesis and function of 3-phosphorylated inositol lipids.,Vanhaesebroeck B, Leevers SJ, Ahmadi K, Timms J, Katso R, Driscoll PC, Woscholski R, Parker PJ, Waterfield MD.,Annu Rev Biochem.,11395417,2001
PI3K3_BIND	Role of Rab5 in the recruitment of hVps34/p150 to the early endosome.,Murray JT, Panaretou C, Stenmark H, Miaczynska M, Backer JM.,Traffic.,12010460,2002,6
PI3K3_RAB5_BIND	Role of Rab5 in the recruitment of hVps34/p150 to the early endosome.,Murray JT, Panaretou C, Stenmark H, Miaczynska M, Backer JM.,Traffic.,12010460,2002,6
PI3K3_RAB5_pail_hs_BIND	Phosphoinositide 3-kinases and the regulation of vesicular trafficking.,Backer JM.,Mol Cell Biol Res Commun.,10891392,2000,4
PI3P4K_pail3p_hs	Phosphoinositides and signal transduction.,Toker A.,Cell Mol Life Sci.,12088277,2002,5
PI3P4K_pail3p_hs	Synthesis and function of 3-phosphorylated inositol lipids.,Vanhaesebroeck B, Leevers SJ, Ahmadi K, Timms J, Katso R, Driscoll PC, Woscholski R, Parker PJ, Waterfield MD.,Annu Rev Biochem.,11395417,2001
PI4P5K_PHOS	Phosphatidic acid is a specific activator of phosphatidylinositol-4-phosphate kinase.,Moritz A, De Graan PN, Gispen WH, Wirtz KW.,J Biol Chem.,1313792,1992,4,15
PI4P5K_PHOS	Rho GTPases and their effector proteins.,Bishop AL, Hall A.,Biochem J.,10816416,2000,6,1
PI4P5K_PHOS	Rho family GTPases bind to phosphoinositide kinases.,Tolias KF, Cantley LC, Carpenter CL.,J Biol Chem.,7629060,1995,7,28
PI4P5K_pail4p_hs	Phosphoinositides and signal transduction.,Toker A.,Cell Mol Life Sci.,12088277,2002,5
PI4P5K_pail4p_hs	Synthesis and function of 3-phosphorylated inositol lipids.,Vanhaesebroeck B, Leevers SJ, Ahmadi K, Timms J, Katso R, Driscoll PC, Woscholski R, Parker PJ, Waterfield MD.,Annu Rev Biochem.,11395417,2001
PKAC_NFKB	Phosphorylation of NF-kappa B p65 by PKA stimulates transcriptional activity by promoting a novel bivalent interaction with the coactivator CBP/p300.,Zhong H, Voll RE, Ghosh S.,Mol Cell.,9660950,1998,4
PKAC_NFKB	Post-translational modifications regulating the activity and function of the nuclear factor kappa B pathway.,Perkins ND.,Oncogene.,17072324,2006,10,30
PKAC_NFKB	The transcriptional activity of NF-kappaB is regulated by the IkappaB-associated PKAc subunit through a cyclic AMP-independent mechanism.,Zhong H, SuYang H, Erdjument-Bromage H, Tempst P, Ghosh S.,Cell.,9150141,1997,5,2

PKAC_NFKB(p50)	DNA binding of repressor nuclear factor-kappaB p50/p50 depends on phosphorylation of Ser337 by the protein kinase A catalytic subunit.,Guan H, Hou S, Ricciardi RP.,J Biol Chem.,15642694,2005,3,18
PKAC_NFKB_P1K	Phosphorylation of NF-kappa B p65 by PKA stimulates transcriptional activity by promoting a novel bivalent interaction with the coactivator CBP/p300.,Zhong H, Voll RE, Ghosh S.,Mol Cell.,9660950,1998,4
PKAC_NFKB_P1K	Post-translational modifications regulating the activity and function of the nuclear factor kappa B pathway.,Perkins ND.,Oncogene.,17072324,2006,10,30
PKAC_NFKB_P1K	The transcriptional activity of NF-kappaB is regulated by the IkappaB-associated PKAc subunit through a cyclic AMP-independent mechanism.,Zhong H, SuYang H, Erdjument-Bromage H, Tempst P, Ghosh S.,Cell.,9150141,1997,5,2
PKA_CREB	DNA binding and phosphorylation induce conformational alterations in the kinase inducible domain of CREB: Implications for the mechanism of CREB transcription function.,Sharma N, Lopez DI, Nyborg JK.,J Biol Chem.,17491014,2007,5,9
PKA_RAF1	Cyclic AMP-dependent kinase regulates Raf-1 kinase mainly by phosphorylation of serine 259.,Dhillon AS, Pollock C, Steen H, Shaw PE, Mischak H, Kolch W.,Mol Cell Biol.,11971957,2002,5
PKA_RAF1	Untying the regulation of the Raf-1 kinase.,Dhillon AS, Kolch W.,Arch Biochem Biophys.,12127063,2002,8,1
PKCA_3PHOS	New insights into the regulation of protein kinase C and novel phorbol ester receptors.,Ron D, Kazanietz MG.,FASEB J.,10506570,1999,10
PKCA_ca2_BIND	Ca2+ differentially regulates conventional protein kinase Cs' membrane interaction and activation.,Keranen LM, Newton AC.,J Biol Chem.,9325330,1997,10,10
PKCA_ca2_BIND	New insights into the regulation of protein kinase C and novel phorbol ester receptors.,Ron D, Kazanietz MG.,FASEB J.,10506570,1999,10
PKCA_ca2_BIND	Regulation of protein kinase C.,Newton AC.,Curr Opin Cell Biol.,9069266,1997,4
PKCA_ca2_P47PHOX	Phosphorylation of p47phox sites by PKC alpha, beta II, delta, and zeta: effect on binding to p22phox and on NADPH oxidase activation.,Fontayne A, Dang PM, Gougerot-Pocidalo MA, El-Benna J.,Biochemistry.,12056906,2002,6,18
PKCA_ca2_P47PHOX	Protein kinase C zeta phosphorylates a subset of selective sites of the NADPH oxidase component p47phox and participates in formyl peptide-mediated neutrophil respiratory burst.,Dang PM, Fontayne A, Hakim J, El Benna J, Périanin A.,J Immunol.,11145703,2001,1,15
PKCA_dag_ps_hs_BIND	New insights into the regulation of protein kinase C and novel phorbol ester receptors.,Ron D, Kazanietz MG.,FASEB J.,10506570,1999,10
PKCA_dag_ps_hs_BIND	Regulation of protein kinase C.,Newton AC.,Curr Opin Cell Biol.,9069266,1997,4
PKCA_dag_ps_hs_P47PHOX	Phosphorylation of p47phox sites by PKC alpha, beta II, delta, and zeta: effect on binding to p22phox and on NADPH oxidase activation.,Fontayne A, Dang PM, Gougerot-Pocidalo MA, El-Benna J.,Biochemistry.,12056906,2002,6,18
PKCA_dag_ps_hs_P47PHOX	Protein kinase C zeta phosphorylates a subset of selective sites of the NADPH oxidase component p47phox and participates in formyl peptide-mediated neutrophil respiratory burst.,Dang PM, Fontayne A, Hakim J, El Benna J, Périanin A.,J Immunol.,11145703,2001,1,15
PKCB2_3PHOS	New insights into the regulation of protein kinase C and novel phorbol ester receptors.,Ron D, Kazanietz MG.,FASEB J.,10506570,1999,10
PKCB2_ca2_BIND	Ca2+ differentially regulates conventional protein kinase Cs' membrane interaction and activation.,Keranen LM, Newton AC.,J Biol Chem.,9325330,1997,10,10
PKCB2_ca2_BIND	New insights into the regulation of protein kinase C and novel phorbol ester receptors.,Ron D, Kazanietz MG.,FASEB J.,10506570,1999,10

PKCB2_ca2_BIND	Regulation of protein kinase C.,Newton AC.,Curr Opin Cell Biol.,9069266,1997,4
PKCB2_ca2_P47PHOX	Phosphorylation of p47phox sites by PKC alpha, beta II, delta, and zeta: effect on binding to p22phox and on NADPH oxidase activation.,Fontayne A, Dang PM, Gougerot-Pocidalo MA, El-Benna J.,Biochemistry.,12056906,2002,6,18
PKCB2_ca2_P47PHOX	Protein kinase C zeta phosphorylates a subset of selective sites of the NADPH oxidase component p47phox and participates in formyl peptide-mediated neutrophil respiratory burst.,Dang PM, Fontayne A, Hakim J, El Benna J, Périanin A.,J Immunol.,11145703,2001,1,15
PKCB2_dag_ps_hs_BIND	New insights into the regulation of protein kinase C and novel phorbol ester receptors.,Ron D, Kazanietz MG.,FASEB J.,10506570,1999,10
PKCB2_dag_ps_hs_BIND	Regulation of protein kinase C.,Newton AC.,Curr Opin Cell Biol.,9069266,1997,4
PKCB2_dag_ps_hs_P47PHOX	Phosphorylation of p47phox sites by PKC alpha, beta II, delta, and zeta: effect on binding to p22phox and on NADPH oxidase activation.,Fontayne A, Dang PM, Gougerot-Pocidalo MA, El-Benna J.,Biochemistry.,12056906,2002,6,18
PKCB2_dag_ps_hs_P47PHOX	Protein kinase C zeta phosphorylates a subset of selective sites of the NADPH oxidase component p47phox and participates in formyl peptide-mediated neutrophil respiratory burst.,Dang PM, Fontayne A, Hakim J, El Benna J, Périanin A.,J Immunol.,11145703,2001,1,15
PKCD_2PHOS	Identification of serine 643 of protein kinase C-delta as an important autophosphorylation site for its enzymatic activity.,Li W, Zhang J, Bottaro DP, Pierce JH.,J Biol Chem.,9305920,1997,9,26
PKCD_2PHOS	New insights into the regulation of protein kinase C and novel phorbol ester receptors.,Ron D, Kazanietz MG.,FASEB J.,10506570,1999,10
PKCD_dag_ps_hs_BIND	New insights into the regulation of protein kinase C and novel phorbol ester receptors.,Ron D, Kazanietz MG.,FASEB J.,10506570,1999,10
PKCD_dag_ps_hs_BIND	Regulation of protein kinase C.,Newton AC.,Curr Opin Cell Biol.,9069266,1997,4
PKCD_dag_ps_hs_P47PHOX	Phosphorylation of p47phox sites by PKC alpha, beta II, delta, and zeta: effect on binding to p22phox and on NADPH oxidase activation.,Fontayne A, Dang PM, Gougerot-Pocidalo MA, El-Benna J.,Biochemistry.,12056906,2002,6,18
PKCD_dag_ps_hs_P47PHOX	Protein kinase C zeta phosphorylates a subset of selective sites of the NADPH oxidase component p47phox and participates in formyl peptide-mediated neutrophil respiratory burst.,Dang PM, Fontayne A, Hakim J, El Benna J, Périanin A.,J Immunol.,11145703,2001,1,15
PKCZ_HNRNPA1	Regulation of in vitro nucleic acid strand annealing activity of heterogeneous nuclear ribonucleoprotein protein A1 by reversible phosphorylation.,Idriss H, Kumar A, Casas-Finet JR, Guo H, Damuni Z, Wilson SH.,Biochemistry.,7727389,1994,9,20
PKCZ_NFKB	Essential role of RelA Ser311 phosphorylation by zetaPKC in NF-kappaB transcriptional activation.,Duran A, Diaz-Meco MT, Moscat J.,EMBO J.,12881425,2003,8,1
PKCZ_NFKB	Post-translational modifications regulating the activity and function of the nuclear factor kappa B pathway.,Perkins ND.,Oncogene.,17072324,2006,10,30
PKCZ_NFKB_P2K	Essential role of RelA Ser311 phosphorylation by zetaPKC in NF-kappaB transcriptional activation.,Duran A, Diaz-Meco MT, Moscat J.,EMBO J.,12881425,2003,8,1
PKCZ_NFKB_P2K	Post-translational modifications regulating the activity and function of the nuclear factor kappa B pathway.,Perkins ND.,Oncogene.,17072324,2006,10,30
PKCZ_P47PHOX	Phosphorylation of p47phox sites by PKC alpha, beta II, delta, and zeta: effect on binding to p22phox and on NADPH oxidase activation.,Fontayne A, Dang PM, Gougerot-Pocidalo MA, El-Benna J.,Biochemistry.,12056906,2002,6,18

PKCZ_P47PHOX	Protein kinase C zeta phosphorylates a subset of selective sites of the NADPH oxidase component p47phox and participates in formyl peptide-mediated neutrophil respiratory burst.,Dang PM, Fontayne A, Hakim J, El Benna J, Périanin A.,J Immunol.,11145703,2001,1,15
PKCZ_TRAN	Nuclear import and export signals enable rapid nucleocytoplasmic shuttling of the atypical protein kinase C lambda.,Perander M, Bjorkoy G, Johansen T.,J Biol Chem.,11115515,2001,4,20
PKCZ_TRAN	Protein kinase Czeta (PKCzeta): activation mechanisms and cellular functions.,Hirai T, Chida K.,J Biochem (Tokyo).,12761192,2003,1
PKCZ_TRAN2	Nuclear import and export signals enable rapid nucleocytoplasmic shuttling of the atypical protein kinase C lambda.,Perander M, Bjorkoy G, Johansen T.,J Biol Chem.,11115515,2001,4,20
PKCZ_TRAN2	Protein kinase Czeta (PKCzeta): activation mechanisms and cellular functions.,Hirai T, Chida K.,J Biochem (Tokyo).,12761192,2003,1
PKCZ_pa_hs_BIND	Phosphatidic acid activation of protein kinase C-zeta overexpressed in COS cells: comparison with other protein kinase C isotypes and other acidic lipids.,Limatola C, Schaap D, Moolenaar WH, van Blitterswijk WJ.,Biochem J.,7818462,1994,12,15
PKCZ_pa_hs_PHOS	Phosphatidic acid activation of protein kinase C-zeta overexpressed in COS cells: comparison with other protein kinase C isotypes and other acidic lipids.,Limatola C, Schaap D, Moolenaar WH, van Blitterswijk WJ.,Biochem J.,7818462,1994,12,15
PKCZ_ps_hs_BIND	Phosphatidic acid activation of protein kinase C-zeta overexpressed in COS cells: comparison with other protein kinase C isotypes and other acidic lipids.,Limatola C, Schaap D, Moolenaar WH, van Blitterswijk WJ.,Biochem J.,7818462,1994,12,15
PKCZ_ps_hs_PHOS	Phosphatidic acid activation of protein kinase C-zeta overexpressed in COS cells: comparison with other protein kinase C isotypes and other acidic lipids.,Limatola C, Schaap D, Moolenaar WH, van Blitterswijk WJ.,Biochem J.,7818462,1994,12,15
PKR_2PHOS	Autophosphorylation in the activation loop is required for full kinase activity in vivo of human and yeast eukaryotic initiation factor 2alpha kinases PKR and GCN2.,Romano PR, Garcia-Barrio MT, Zhang X, Wang Q, Taylor DR, Zhang F, Herring C, Mathews MB, Qin J, Hinnebusch AG.,Mol Cell Biol.,9528799,1998,4
PKR_2PHOS	Binding of double-stranded RNA to protein kinase PKR is required for dimerization and promotes critical autophosphorylation events in the activation loop.,Zhang F, Romano PR, Nagamura-Inoue T, Tian B, Dever TE, Mathews MB, Ozato K, Hinnebusch AG.,J Biol Chem.,11337501,2001,7,6
PKR_DIME	Autophosphorylation in the activation loop is required for full kinase activity in vivo of human and yeast eukaryotic initiation factor 2alpha kinases PKR and GCN2.,Romano PR, Garcia-Barrio MT, Zhang X, Wang Q, Taylor DR, Zhang F, Herring C, Mathews MB, Qin J, Hinnebusch AG.,Mol Cell Biol.,9528799,1998,4
PKR_DIME	Binding of double-stranded RNA to protein kinase PKR is required for dimerization and promotes critical autophosphorylation events in the activation loop.,Zhang F, Romano PR, Nagamura-Inoue T, Tian B, Dever TE, Mathews MB, Ozato K, Hinnebusch AG.,J Biol Chem.,11337501,2001,7,6
PKR_EIF2A	Caspase-mediated cleavage of eukaryotic translation initiation factor subunit 2alpha.,Satoh S, Hijikata M, Handa H, Shimotohno K.,Biochem J.,10432301,1999,8,15
PKR_TRAF6_BIND	TRAF family proteins link PKR with NF-kappa B activation.,Gil J, García MA, Gomez-Puertas P, Guerra S, Rullas J, Nakano H, Alcamí J, Esteban M.,Mol Cell Biol.,15121867,2004,5
PLCB2_ACTI	Stimulation of phospholipase C-beta2 by the Rho GTPases Cdc42Hs and Rac1.,Illenberger D, Schwald F, Pimmer D, Binder W, Maier G, Dietrich A, Gierschik P.,EMBO J.,9799233,1998,11,2

PLD_ACTI	Regulation of phospholipase D by protein kinase C is synergistic with ADP-ribosylation factor and independent of protein kinase activity.,Singer WD, Brown HA, Jiang X, Sternweis PC.,J Biol Chem.,8626805,1996,2,23
PLD_ACTI	Rho GTPases and their effector proteins.,Bishop AL, Hall A.,Biochem J.,10816416,2000,6,1
PLD_ACTI	RhoA-mediated Phospholipase D1 signaling is not required for the formation of stress fibers and focal adhesions.,Su W, Chardin P, Yamazaki M, Kanaho Y, Du G.,Cell Signal.,15993039,2006,4
PLD_ACTI	The regulation of phospholipase D by inositol phospholipids and small GTPases.,Powner DJ, Wakelam MJ.,FEBS Lett.,12401204,2002,10,30
PP2A_DEPH	Structure and regulation of MAPK phosphatases.,Farooq A, Zhou MM.,Cell Signal.,15115656,2004,7
PP2C_B1_MAP3K7IP_TRAF6	Regulation of the TAK1 signaling pathway by protein phosphatase 2C.,Hanada M, Ninomiya-Tsuji J, Komaki K, Ohnishi M, Katsura K, Kanamaru R, Matsumoto K, Tamura S.,J Biol Chem.,11104763,2001,2,23
PP2C_E_MAP3K7IP_TRAF6	Regulation of the interleukin-1-induced signaling pathways by a novel member of the protein phosphatase 2C family (PP2Cepsilon).,Li MG, Katsura K, Nomiyama H, Komaki K, Ninomiya-Tsuji J, Matsumoto K, Kobayashi T, Tamura S.,J Biol Chem.,12556533,2003,4,4
PRE_IL1A_CALPN_3ca2_BIND	Differential sensitivity of interleukin-1 alpha and -beta precursor proteins to cleavage by calpain, a calcium-dependent protease.,Kavita U, Mizel SB.,J Biol Chem.,7499244,1995,11,17
PRE_IL1A_CALPN_3ca2_BIND	Identification of calcium-activated neutral protease as a processing enzyme of human interleukin 1 alpha.,Kobayashi Y, Yamamoto K, Saido T, Kawasaki H, Oppenheim JJ, Matsushima K.,Proc Natl Acad Sci USA,2115174,1990,7
PRE_IL1A_PROC	Differential sensitivity of interleukin-1 alpha and -beta precursor proteins to cleavage by calpain, a calcium-dependent protease.,Kavita U, Mizel SB.,J Biol Chem.,7499244,1995,11,17
PRE_IL1A_PROC	Identification of calcium-activated neutral protease as a processing enzyme of human interleukin 1 alpha.,Kobayashi Y, Yamamoto K, Saido T, Kawasaki H, Oppenheim JJ, Matsushima K.,Proc Natl Acad Sci USA,2115174,1990,7
PRE_IL1B_CASP1_BIND	A novel heterodimeric cysteine protease is required for interleukin-1 beta processing in monocytes.,Thornberry NA, Bull HG, Calaycay JR, Chapman KT, Howard AD, Kostura MJ, Miller DK, Molineaux SM, Weidner JR, Aunins J, et al.,Nature.,1574116,1992,4,30
PRE_IL1B_CASP1_BIND	Identification of a monocyte specific pre-interleukin 1 beta convertase activity.,Kostura MJ, Tocci MJ, Limjuco G, Chin J, Cameron P, Hillman AG, Chartrain NA, Schmidt JA.,Proc Natl Acad Sci USA.,2787508,1989,7
PRE_IL1B_PROC	A novel heterodimeric cysteine protease is required for interleukin-1 beta processing in monocytes.,Thornberry NA, Bull HG, Calaycay JR, Chapman KT, Howard AD, Kostura MJ, Miller DK, Molineaux SM, Weidner JR, Aunins J, et al.,Nature.,1574116,1992,4,30
PRE_IL1B_PROC	Identification of a monocyte specific pre-interleukin 1 beta convertase activity.,Kostura MJ, Tocci MJ, Limjuco G, Chin J, Cameron P, Hillman AG, Chartrain NA, Schmidt JA.,Proc Natl Acad Sci USA.,2787508,1989,7
RAB5_PHOS	Functional synergy between Rab5 effector Rabaptin-5 and exchange factor Rabex-5 when physically associated in a complex.,Lippé R, Miaczynska M, Rybin V, Runge A, Zerial M.,Mol Biol Cell.,11452015,2001,7
RAB5_RBPTIN5_RBX5_BIND	Functional synergy between Rab5 effector Rabaptin-5 and exchange factor Rabex-5 when physically associated in a complex.,Lippé R, Miaczynska M, Rybin V, Runge A, Zerial M.,Mol Biol Cell.,11452015,2001,7
RAC1_DEPH	Phosphotyrosine-dependent activation of Rac-1 GDP/GTP exchange by the vav proto-oncogene product.,Crespo P, Schuebel KE, Ostrom AA, Gutkind JS, Bustelo XR.,Nature.,8990121,1997,1,9

RAC1_PHOS	Phosphotyrosine-dependent activation of Rac-1 GDP/GTP exchange by the vav proto-oncogene product.,Crespo P, Schuebel KE, Ostrom AA, Gutkind JS, Bustelo XR.,Nature.,8990121,1997,1,9
RAC1_VAV1_BIND	Phosphotyrosine-dependent activation of Rac-1 GDP/GTP exchange by the vav proto-oncogene product.,Crespo P, Schuebel KE, Ostrom AA, Gutkind JS, Bustelo XR.,Nature.,8990121,1997,1,9
RAF1_DEPH1	Protein phosphatase 2A positively regulates Ras signaling by dephosphorylating KSR1 and Raf-1 on critical 14-3-3 binding sites.,Ory S, Zhou M, Conrads TP, Veenstra TD, Morrison DK.,Curr Biol.,12932319,2003,8,19
RAF1_DEPH1	Untying the regulation of the Raf-1 kinase.,Dhillon AS, Kolch W.,Arch Biochem Biophys.,12127063,2002,8,1
RAF1_DEPH2	Protein phosphatase 2A positively regulates Ras signaling by dephosphorylating KSR1 and Raf-1 on critical 14-3-3 binding sites.,Ory S, Zhou M, Conrads TP, Veenstra TD, Morrison DK.,Curr Biol.,12932319,2003,8,19
RAF1_DEPH2	Untying the regulation of the Raf-1 kinase.,Dhillon AS, Kolch W.,Arch Biochem Biophys.,12127063,2002,8,1
RAS_DEPH	Human RAS superfamily proteins and related GTPases.,Colicelli J.,Sci STKE.,15367757,2004,9,7
RAS_GTP_RAF1_BIND	Phosphorylation and regulation of Raf by Akt (protein kinase B).,Zimmermann S, Moelling K.,Science.,10576742,1999,11,26
RAS_GTP_RAF1_BIND	Untying the regulation of the Raf-1 kinase.,Dhillon AS, Kolch W.,Arch Biochem Biophys.,12127063,2002,8,1
RAS_GTP_RIN1_BIND	A human protein selected for interference with Ras function interacts directly with Ras and competes with Raf1.,Han L, Colicelli J.,Mol Cell Biol.,7862125,1995,3
RAS_GTP_RIN1_BIND	The RAS effector RIN1 directly competes with RAF and is regulated by 14-3-3 proteins.,Wang Y, Waldron RT, Dhaka A, Patel A, Riley MM, Rozengurt E, Colicelli J.,Mol Cell Biol.,11784866,2002,2
RAS_PHOS	Human RAS superfamily proteins and related GTPases.,Colicelli J.,Sci STKE.,15367757,2004,9,7
RAS_PHOS	Ras participates in CpG oligodeoxynucleotide signaling through association with toll-like receptor 9 and promotion of interleukin-1 receptor-associated kinase/tumor necrosis factor receptor-associated factor 6 complex formation in macrophages.,Xu H, An H, Yu Y, Zhang M, Qi R, Cao X.,J Biol Chem.,12867418,2003,9,19
RBPTIN5_RBX5_BIND	Functional synergy between Rab5 effector Rabaptin-5 and exchange factor Rabex-5 when physically associated in a complex.,Lippé R, Miaczynska M, Rybin V, Runge A, Zerial M.,Mol Biol Cell.,11452015,2001,7
RHOA_PHOS	The IL-1 receptor and Rho directly associate to drive cell activation in inflammation.,Singh R, Wang B, Shirvaikar A, Khan S, Kamat S, Schelling JR, Konieczkowski M, Sedor JR.,J Clin Invest.,10359565,1999,6
RHOA_PHOS	The low molecular weight GTPase RhoA and atypical protein kinase Czeta are required for TLR2-mediated gene transcription.,Teusch N, Lombardo E, Eddleston J, Knaus UG.,J Immunol.,15210811,2004,7,1
RIP1_TICAM1P_BIND	RIP1 is an essential mediator of Toll-like receptor 3-induced NF-kappa B activation.,Meylan E, Burns K, Hofmann K, Blancheteau V, Martinon F, Kelliher M, Tschopp J.,Nat Immunol.,15064760,2004,5
RIP1_TICAM1P_BIND	TOO LONG: See pubmed ID,Sato S, Sugiyama M, Yamamoto M, Watanabe Y, Kawai T, Takeda K, Akira S.,J Immunol.,14530355,2003,10,15
RIP2_NOD1P_BIND	CATERPILLER: a novel gene family important in immunity, cell death, and diseases.,Ting JP, Davis BK.,Annu Rev Immunol.,15771576,2005

RIP2_NOD1P_BIND	Nod1, an Apaf-1-like activator of caspase-9 and nuclear factor-kappaB.,Inohara N, Koseki T, del Peso L, Hu Y, Yee C, Chen S, Carrio R, Merino J, Liu D, Ni J, Núñez G.,J Biol Chem.,10329646,1999,5,21
RIP2_NOD2P_BIND	CATERPILLER: a novel gene family important in immunity, cell death, and diseases.,Ting JP, Davis BK.,Annu Rev Immunol.,15771576,2005
RIP2_NOD2P_BIND	Nod2, a Nod1/Apaf-1 family member that is restricted to monocytes and activates NF-kappaB.,Ogura Y, Inohara N, Benito A, Chen FF, Yamaoka S, Nunez G.,J Biol Chem.,11087742,2001,2,16
RIP2_TRIP6_NOD1P_BIND	TRIP6 is a RIP2-associated common signaling component of multiple NF-kappaB activation pathways.,Li L, Bin LH, Li F, Liu Y, Chen D, Zhai Z, Shu HB.,J Cell Sci.,15657077,2005,2,1
RIP2_TRIP6_TRAF2_BIND	TRIP6 is a RIP2-associated common signaling component of multiple NF-kappaB activation pathways.,Li L, Bin LH, Li F, Liu Y, Chen D, Zhai Z, Shu HB.,J Cell Sci.,15657077,2005,2,1
RKIP_MAP3K14_BIND	Raf kinase inhibitor protein interacts with NF-kappaB-inducing kinase and TAK1 and inhibits NF-kappaB activation.,Yeung KC, Rose DW, Dhillon AS, Yaros D, Gustafsson M, Chatterjee D, McFerran B, Wyche J, Kolch W, Sedivy JM.,Mol Cell Biol.,11585904,2001,11
RKIP_MAP3K7IP_TRAF6_BIND	Raf kinase inhibitor protein interacts with NF-kappaB-inducing kinase and TAK1 and inhibits NF-kappaB activation.,Yeung KC, Rose DW, Dhillon AS, Yaros D, Gustafsson M, Chatterjee D, McFerran B, Wyche J, Kolch W, Sedivy JM.,Mol Cell Biol.,11585904,2001,11
SGK_MAP3K3	Inhibition of mitogen-activated kinase kinase kinase 3 activity through phosphorylation by the serum- and glucocorticoid-induced kinase 1.,Chun J, Kwon T, Kim DJ, Park I, Chung G, Lee EJ, Hong SK, Chang SI, Kim HY, Kang SS.,J Biochem (Tokyo),12761204,2003,1
SIGIRR_DIME	Modulation of Toll-interleukin 1 receptor mediated signaling.,Li X, Qin J.,J Mol Med.,15662540,2005,4
SIGIRR_DIME	SIGIRR, a negative regulator of Toll-like receptor-interleukin 1 receptor signaling.,Wald D, Qin J, Zhao Z, Qian Y, Naramura M, Tian L, Towne J, Sims JE, Stark GR, Li X.,Nat Immunol.,12925853,2003,9
SQSTM1_PKCZ_BIND	Localization of atypical protein kinase C isoforms into lysosome-targeted endosomes through interaction with p62.,Sanchez P, De Carcer G, Sandoval IV, Moscat J, Diaz-Meco MT.,Mol Cell Biol.,9566925,1998,5
SRCK_BTK	Bruton's tyrosine kinase (Btk)-the critical tyrosine kinase in LPS signalling?,Jefferies CA, O'Neill LA.,Immunol Lett.,15081522,2004,3,29
SRCK_BTK	Transphosphorylation of Bruton's tyrosine kinase on tyrosine 551 is critical for B cell antigen receptor function.,Kurosaki T, Kurosaki M.,J Biol Chem.,9188445,1997,6,20
SRCK_CBL	Inhibition of Src family kinases blocks epidermal growth factor (EGF)-induced activation of Akt, phosphorylation of c-Cbl, and ubiquitination of the EGF receptor.,Kassenbrock CK, Hunter S, Garl P, Johnson GL, Anderson SM.,J Biol Chem.,11994282,2002,7,12
SRCK_CBL	Molecular complexes that contain both c-Cbl and c-Src associate with Golgi membranes.,Bard F, Patel U, Levy JB, Jurdic P, Horne WC, Baron R.,Eur J Cell Biol.,11893076,2002,1
SRCK_PHOS	Activation of src-family tyrosine kinases by LPS regulates cytokine production in dendritic cells by controlling AP-1 formation.,Napolitani G, Bortoletto N, Racioppi L, Lanzavecchia A, D'Oro U.,Eur J Immunol.,14515267,2003,10
SRCK_PHOS	Lipopolysaccharide (LPS)-induced macrophage activation and signal transduction in the absence of Src-family kinases Hck, Fgr, and Lyn.,Meng F, Lowell CA.,J Exp Med.,9151903,1997,5,5

SRCK_PHOS	The regulation of the expression of inducible nitric oxide synthase by Src-family tyrosine kinases mediated through MyD88-independent signaling pathways of Toll-like receptor 4.,Lee JY, Lowell CA, Lemay DG, Youn HS, Rhee SH, Sohn KH, Jang B, Ye J, Chung JH, Hwang DH.,Biochem Pharmacol.,16140274,2005,10,15
SRTK_PHOS	Role of vav1- and src-related tyrosine kinases in macrophage activation by CpG DNA.,Stovall SH, Yi AK, Meals EA, Talati AJ, Godambe SA, English BK.,J Biol Chem.,14749335,2004,4,2
SRTK_VAV1	Role of vav1- and src-related tyrosine kinases in macrophage activation by CpG DNA.,Stovall SH, Yi AK, Meals EA, Talati AJ, Godambe SA, English BK.,J Biol Chem.,14749335,2004,4,2
ST2L_TIRAP_BIND	A novel negative regulator for IL-1 receptor and Toll-like receptor 4.,Liew FY, Liu H, Xu D.,Immunol Lett.,15585304,2005,1,15
ST2L_TIRAP_BIND	Modulation of Toll-interleukin 1 receptor mediated signaling.,Li X, Qin J.,J Mol Med.,15662540,2005,4
ST2L_TIRAP_BIND	Negative regulation of toll-like receptor-mediated immune responses.,Liew FY, Xu D, Brint EK, O'Neill LA.,Nat Rev Immunol.,15928677,2005,6
ST2L_TIRAP_BIND	ST2 is an inhibitor of interleukin 1 receptor and Toll-like receptor 4 signaling and maintains endotoxin tolerance.,Brint EK, Xu D, Liu H, Dunne A, McKenzie AN, O'Neill LA, Liew FY.,Nat Immunol.,15004556,2004,4
STLR2/L_SCD14_BIND	Negative regulation of toll-like receptor-mediated immune responses.,Liew FY, Xu D, Brint EK, O'Neill LA.,Nat Rev Immunol.,15928677,2005,6
STLR2/L_SCD14_BIND	Soluble forms of Toll-like receptor (TLR)2 capable of modulating TLR2 signaling are present in human plasma and breast milk.,LeBouder E, Rey-Nores JE, Rushmere NK, Grigorov M, Lawn SD, Affolter M, Griffin GE, Ferrara P, Schiffrin EJ, Morgan BP, Labéta MO.,J Immunol.,14662871,2003,12,15
STLR2/L_SCD14_BIND	The extracellular toll-like receptor 2 domain directly binds peptidoglycan derived from Staphylococcus aureus.,Iwaki D, Mitsuzawa H, Murakami S, Sano H, Konishi M, Akino T, Kuroki Y.,J Biol Chem.,11986301,2002,7,5
STLR2_CD14_BIND	Negative regulation of toll-like receptor-mediated immune responses.,Liew FY, Xu D, Brint EK, O'Neill LA.,Nat Rev Immunol.,15928677,2005,6
STLR4_BIND	Interaction of soluble form of recombinant extracellular TLR4 domain with MD-2 enables lipopolysaccharide binding and attenuates TLR4-mediated signaling.,Hyakushima N, Mitsuzawa H, Nishitani C, Sano H, Kuronuma K, Konishi M, Himi T, Miyake K, Kuroki Y.,J Immunol.,15557191,2004,12,1
STLR4_MD2_BIND	Interaction of soluble form of recombinant extracellular TLR4 domain with MD-2 enables lipopolysaccharide binding and attenuates TLR4-mediated signaling.,Hyakushima N, Mitsuzawa H, Nishitani C, Sano H, Kuronuma K, Konishi M, Himi T, Miyake K, Kuroki Y.,J Immunol.,15557191,2004,12,1
TBK1_IRF3	A20 is a negative regulator of IFN regulatory factor 3 signaling.,Saitoh T, Yamamoto M, Miyagishi M, Taira K, Nakanishi M, Fujita T, Akira S, Yamamoto N, Yamaoka S.,J Immunol.,15661910,2005,2,1
TBK1_IRF3	IKKepsilon and TBK1 are essential components of the IRF3 signaling pathway.,Fitzgerald KA, McWhirter SM, Faia KL, Rowe DC, Latz E, Golenbock DT, Coyle AJ, Liao SM, Maniatis T.,Nat Immunol.,12692549,2003,5
TBK1_IRF3	Identification of Ser-386 of interferon regulatory factor 3 as critical target for inducible phosphorylation that determines activation.,Mori M, Yoneyama M, Ito T, Takahashi K, Inagaki F, Fujita T.,J Biol Chem.,14703513,2004,3,12
TBK1_IRF3	Structural and functional analysis of interferon regulatory factor 3: localization of the transactivation and autoinhibitory domains.,Lin R, Mamane Y, Hiscott J.,Mol Cell Biol.,10082512,1999,4
TBK1_NFKB	Post-translational modifications regulating the activity and function of the nuclear factor kappa B pathway.,Perkins ND.,Oncogene.,17072324,2006,10,30
TBK1_NFKB	TOO LONG: See PubMed ID,Buss H, Dörrie A, Schmitz ML, Hoffmann E, Resch K, Kracht M.,J Biol Chem.,15489227,2004,12,31

TBK1_NFKB_P5K	Post-translational modifications regulating the activity and function of the nuclear factor kappa B pathway.,Perkins ND.,Oncogene.,17072324,2006,10,30
TBK1_NFKB_P5K	TOO LONG: See PubMed ID,Buss H, Dörrie A, Schmitz ML, Hoffmann E, Resch K, Kracht M.,J Biol Chem.,15489227,2004,12,31
TBK1_PHOS	TOO LONG: See pubmed ID,Sato S, Sugiyama M, Yamamoto M, Watanabe Y, Kawai T, Takeda K, Akira S.,J Immunol.,14530355,2003,10,15
TICAM1P_IKKE_BIND	TOO LONG: See pubmed ID,Sato S, Sugiyama M, Yamamoto M, Watanabe Y, Kawai T, Takeda K, Akira S.,J Immunol.,14530355,2003,10,15
TICAM1P_IKKE_BIND	Triggering the interferon antiviral response through an IKK-related pathway.,Sharma S, tenOever BR, Grandvaux N, Zhou GP, Lin R, Hiscott J.,Science.,12702806,2003,5,16
TICAM1P_TBK1_BIND	TOO LONG: See pubmed ID,Sato S, Sugiyama M, Yamamoto M, Watanabe Y, Kawai T, Takeda K, Akira S.,J Immunol.,14530355,2003,10,15
TICAM2_TICAM1	TICAM-1, an adaptor molecule that participates in Toll-like receptor 3-mediated interferon-beta induction.,Oshiumi H, Matsumoto M, Funami K, Akazawa T, Seya T.,Nat Immunol.,12539043,2003,2
TICAM2_TICAM1	TIR-containing adapter molecule (TICAM)-2, a bridging adapter recruiting to toll-like receptor 4 TICAM-1 that induces interferon-beta.,Oshiumi H, Sasai M, Shida K, Fujita T, Matsumoto M, Seya T.,J Biol Chem.,14519765,2003,12,12
TICAM2_TLR4/L_MD2_BIND	TIR-containing adapter molecule (TICAM)-2, a bridging adapter recruiting to toll-like receptor 4 TICAM-1 that induces interferon-beta.,Oshiumi H, Sasai M, Shida K, Fujita T, Matsumoto M, Seya T.,J Biol Chem.,14519765,2003,12,12
TIR_MYD_BIND	Cooperation of Toll-like receptor signals in innate immune defence.,Trinchieri G, Sher A.,Nat Rev Immunol.,17318230,2007,3
TIR_MYD_BIND	Toll-like receptor function and signaling.,Kaisho T, Akira S.,J Allergy Clin Immunol.,16675322,2006,5
TIR_MYD_BIND2	Cooperation of Toll-like receptor signals in innate immune defence.,Trinchieri G, Sher A.,Nat Rev Immunol.,17318230,2007,3
TIR_MYD_BIND2	Toll-like receptor function and signaling.,Kaisho T, Akira S.,J Allergy Clin Immunol.,16675322,2006,5
TIR_MYD_BIND3	Cooperation of Toll-like receptor signals in innate immune defence.,Trinchieri G, Sher A.,Nat Rev Immunol.,17318230,2007,3
TIR_MYD_BIND3	Toll-like receptor function and signaling.,Kaisho T, Akira S.,J Allergy Clin Immunol.,16675322,2006,5
TIR_MYD_BIND4	Cooperation of Toll-like receptor signals in innate immune defence.,Trinchieri G, Sher A.,Nat Rev Immunol.,17318230,2007,3
TIR_MYD_BIND4	Toll-like receptor function and signaling.,Kaisho T, Akira S.,J Allergy Clin Immunol.,16675322,2006,5
TIR_MYD_BIND5	Cooperation of Toll-like receptor signals in innate immune defence.,Trinchieri G, Sher A.,Nat Rev Immunol.,17318230,2007,3
TIR_MYD_BIND5	Toll-like receptor function and signaling.,Kaisho T, Akira S.,J Allergy Clin Immunol.,16675322,2006,5
TIR_MYD_BIND6	Cooperation of Toll-like receptor signals in innate immune defence.,Trinchieri G, Sher A.,Nat Rev Immunol.,17318230,2007,3
TIR_MYD_BIND6	Toll-like receptor function and signaling.,Kaisho T, Akira S.,J Allergy Clin Immunol.,16675322,2006,5
TIR_MYD_BIND7	Cooperation of Toll-like receptor signals in innate immune defence.,Trinchieri G, Sher A.,Nat Rev Immunol.,17318230,2007,3
TIR_MYD_BIND7	Toll-like receptor function and signaling.,Kaisho T, Akira S.,J Allergy Clin Immunol.,16675322,2006,5
TIR_MYD_BIND8	Cooperation of Toll-like receptor signals in innate immune defence.,Trinchieri G, Sher A.,Nat Rev Immunol.,17318230,2007,3
TIR_MYD_BIND8	Toll-like receptor function and signaling.,Kaisho T, Akira S.,J Allergy Clin Immunol.,16675322,2006,5

TIR_MYD_BIND9	Cooperation of Toll-like receptor signals in innate immune defence.,Trinchieri G, Sher A.,Nat Rev Immunol.,17318230,2007,3
TIR_MYD_BIND9	Toll-like receptor function and signaling.,Kaisho T, Akira S.,J Allergy Clin Immunol.,16675322,2006,5
TIR_MYD_FADD_BIND	Membrane oligomerization and cleavage activates the caspase-8 (FLICE/MACHalpha1) death signal.,Martin DA, Siegel RM, Zheng L, Lenardo MJ.,J Biol Chem.,9468483,1998,2,20
TIR_MYD_FADD_BIND	The apoptotic signaling pathway activated by Toll-like receptor-2.,Aliprantis AO, Yang RB, Weiss DS, Godowski P, Zychlinsky A.,EMBO J.,10880445,2000,7,3
TIR_MYD_IRAK4_BIND	Inhibition of interleukin 1 receptor/Toll-like receptor signaling through the alternatively spliced, short form of MyD88 is due to its failure to recruit IRAK-4.,Burns K, Janssens S, Brissoni B, Olivos N, Beyaert R, Tschopp J.,J Exp Med.,12538665,2003,1,20
TIR_MYD_IRAK4_BIND	MyD88: an adapter that recruits IRAK to the IL-1 receptor complex.,Wesche H, Henzel WJ, Shillinglaw W, Li S, Cao Z.,Immunity,9430229,1997,12
TIR_MYD_TOLLIP_BIND	A novel splice variant of interleukin-1 receptor (IL-1R)-associated kinase 1 plays a negative regulatory role in Toll/IL-1R-induced inflammatory signaling.,Rao N, Nguyen S, Ngo K, Fung-Leung WP.,Mol Cell Biol.,16024789,2005,8
TIR_TIRAP_BIND	Cooperation of Toll-like receptor signals in innate immune defence.,Trinchieri G, Sher A.,Nat Rev Immunol.,17318230,2007,3
TIR_TIRAP_BIND	Essential role for TIRAP in activation of the signalling cascade shared by TLR2 and TLR4.,Yamamoto M, Sato S, Hemmi H, Sanjo H, Uematsu S, Kaisho T, Hoshino K, Takeuchi O, Kobayashi M, Fujita T, Takeda K, Akira S.,Nature,12447441,2002,11,21
TIR_TIRAP_BIND	Toll-like receptor function and signaling.,Kaisho T, Akira S.,J Allergy Clin Immunol.,16675322,2006,5
TIR_TIRAP_BIND2	Cooperation of Toll-like receptor signals in innate immune defence.,Trinchieri G, Sher A.,Nat Rev Immunol.,17318230,2007,3
TIR_TIRAP_BIND2	Essential role for TIRAP in activation of the signalling cascade shared by TLR2 and TLR4.,Yamamoto M, Sato S, Hemmi H, Sanjo H, Uematsu S, Kaisho T, Hoshino K, Takeuchi O, Kobayashi M, Fujita T, Takeda K, Akira S.,Nature,12447441,2002,11,21
TIR_TIRAP_BIND2	Toll-like receptor function and signaling.,Kaisho T, Akira S.,J Allergy Clin Immunol.,16675322,2006,5
TIR_TIRAP_BIND3	Cooperation of Toll-like receptor signals in innate immune defence.,Trinchieri G, Sher A.,Nat Rev Immunol.,17318230,2007,3
TIR_TIRAP_BIND3	Essential role for TIRAP in activation of the signalling cascade shared by TLR2 and TLR4.,Yamamoto M, Sato S, Hemmi H, Sanjo H, Uematsu S, Kaisho T, Hoshino K, Takeuchi O, Kobayashi M, Fujita T, Takeda K, Akira S.,Nature,12447441,2002,11,21
TIR_TIRAP_BIND3	Toll-like receptor function and signaling.,Kaisho T, Akira S.,J Allergy Clin Immunol.,16675322,2006,5
TIR_TIRAP_BIND4	Cooperation of Toll-like receptor signals in innate immune defence.,Trinchieri G, Sher A.,Nat Rev Immunol.,17318230,2007,3
TIR_TIRAP_BIND4	Essential role for TIRAP in activation of the signalling cascade shared by TLR2 and TLR4.,Yamamoto M, Sato S, Hemmi H, Sanjo H, Uematsu S, Kaisho T, Hoshino K, Takeuchi O, Kobayashi M, Fujita T, Takeda K, Akira S.,Nature,12447441,2002,11,21
TIR_TIRAP_BIND4	Toll-like receptor function and signaling.,Kaisho T, Akira S.,J Allergy Clin Immunol.,16675322,2006,5

TIR_TIRAP_IRAK2_BIND	Mal (MyD88-adapter-like) is required for Toll-like receptor-4 signal transduction.,Fitzgerald KA, Palsson-McDermott EM, Bowie AG, Jefferies CA, Mansell AS, Brady G, Brint E, Dunne A, Gray P, Harte MT, McMurray D, Smith DE, Sims JE, Bird TA, O'Neill LA.,Nature,11544529,2001,9,6
TIR_TIRAP_IRAK2_BIND	Toll-like receptor signaling.,Akira S.,J Biol Chem.,12893815,2003,10,3
TIR_TIRAP_IRAK4_BIND	IRAK-4: a novel member of the IRAK family with the properties of an IRAK-kinase.,Li S, Strelow A, Fontana EJ, Wesche H.,Proc Natl Acad Sci USA,11960013,2002,4,16
TIR_TIRAP_IRAK4_BIND	Toll-like receptor signaling.,Akira S.,J Biol Chem.,12893815,2003,10,3
TIR_TIRAP_MYD_BIND	Essential role for TIRAP in activation of the signalling cascade shared by TLR2 and TLR4.,Yamamoto M, Sato S, Hemmi H, Sanjo H, Uematsu S, Kaisho T, Hoshino K, Takeuchi O, Kobayashi M, Fujita T, Takeda K, Akira S.,Nature,12447441,2002,11,21
TIR_TIRAP_MYD_BIND	The adaptor molecule TIRAP provides signalling specificity for Toll-like receptors.,Horng T, Barton GM, Flavell RA, Medzhitov R.,Nature,12447442,2002,11,21
TIR_TIRAP_MYD_FADD_BIND	Membrane oligomerization and cleavage activates the caspase-8 (FLICE/MACHalpha1) death signal.,Martin DA, Siegel RM, Zheng L, Lenardo MJ.,J Biol Chem.,9468483,1998,2,20
TIR_TIRAP_MYD_FADD_BIND	The apoptotic signaling pathway activated by Toll-like receptor-2.,Aliprantis AO, Yang RB, Weiss DS, Godowski P, Zychlinsky A.,EMBO J.,10880445,2000,7,3
TIR_TIRAP_TOLLIP_2PHOS	Negative regulation of toll-like receptor-mediated signaling by Tollip.,Zhang G, Ghosh S.,J Biol Chem.,11751856,2002,3,1
TIR_TIRAP_TOLLIP_2PHOS	Sequential autophosphorylation steps in the interleukin-1 receptor-associated kinase-1 regulate its availability as an adapter in interleukin-1 signaling.,Kollewe C, Mackensen AC, Neumann D, Knop J, Cao P, Li S, Wesche H, Martin MU.,J Biol Chem.,14625308,2004,2,13
TIR_TIRAP_TOLLIP_BIND	Negative regulation of toll-like receptor-mediated signaling by Tollip.,Zhang G, Ghosh S.,J Biol Chem.,11751856,2002,3,1
TIR_TIRAP_TOLLIP_BIND	Toll-like receptor signaling.,Akira S.,J Biol Chem.,12893815,2003,10,3
TIR_TIRAP_TOLLIP_PHOS	IRAK-4: a novel member of the IRAK family with the properties of an IRAK-kinase.,Li S, Strelow A, Fontana EJ, Wesche H.,Proc Natl Acad Sci USA,11960013,2002,4,16
TIR_TIRAP_TOLLIP_PHOS	Negative regulation of toll-like receptor-mediated immune responses.,Liew FY, Xu D, Brint EK, O'Neill LA.,Nat Rev Immunol.,15928677,2005,6
TIR_TIRAP_TOLLIP_PHOS	Negative regulation of toll-like receptor-mediated signaling by Tollip.,Zhang G, Ghosh S.,J Biol Chem.,11751856,2002,3,1
TIR_TIRAP_TOLLIP_PHOS	Sequential autophosphorylation steps in the interleukin-1 receptor-associated kinase-1 regulate its availability as an adapter in interleukin-1 signaling.,Kollewe C, Mackensen AC, Neumann D, Knop J, Cao P, Li S, Wesche H, Martin MU.,J Biol Chem.,14625308,2004,2,13
TLR1/10_BIND	Human TLR10 is a functional receptor, expressed by B cells and plasmacytoid dendritic cells, which activates gene transcription through MyD88.,Hasan U, Chaffois C, Gaillard C, Saulnier V, Merck E, Tancredi S, Guiet C, Brière F, Vlach J, Lebecque S, Trinchieri G, Bates EE.,J Immunol.,15728506,2005,3,1
TLR1/10_BIND	Inferences, questions and possibilities in Toll-like receptor signalling.,Beautler B.,Nature.,15241424,2004,7,8
TLR1/10_BIND	TLR signaling.,Kawai T, Akira S.,Cell Death Differ.,16410796,2006,5
TLR1/10_BIND	Toll-like receptor function and signaling.,Kaisho T, Akira S.,J Allergy Clin Immunol.,16675322,2006,5
TLR1/10_BIND	Toll-like receptor signaling.,Akira S.,J Biol Chem.,12893815,2003,10,3

TLR1/10_BIND	Toll-like receptors and innate immunity.,Uematsu S, Akira S.,J Mol Med.,16924467,2006,9
TLR1/2_BIND	Inferences, questions and possibilities in Toll-like receptor signalling.,Beautler B.,Nature.,15241424,2004,7,8
TLR1/2_BIND	TLR signaling.,Kawai T, Akira S.,Cell Death Differ.,16410796,2006,5
TLR1/2_BIND	Toll-like receptor function and signaling.,Kaisho T, Akira S.,J Allergy Clin Immunol.,16675322,2006,5
TLR1/2_BIND	Toll-like receptor signaling.,Akira S.,J Biol Chem.,12893815,2003,10,3
TLR1/2_BIND	Toll-like receptors and innate immunity.,Uematsu S, Akira S.,J Mol Med.,16924467,2006,9
TLR10_BIND	Human TLR10 is a functional receptor, expressed by B cells and plasmacytoid dendritic cells, which activates gene transcription through MyD88.,Hasan U, Chaffois C, Gaillard C, Saulnier V, Merck E, Tancredi S, Guiet C, Brière F, Vlach J, Lebecque S, Trinchieri G, Bates EE.,J Immunol.,15728506,2005,3,1
TLR10_BIND	Inferences, questions and possibilities in Toll-like receptor signalling.,Beautler B.,Nature.,15241424,2004,7,8
TLR10_BIND	TLR signaling.,Kawai T, Akira S.,Cell Death Differ.,16410796,2006,5
TLR10_BIND	Toll-like receptor function and signaling.,Kaisho T, Akira S.,J Allergy Clin Immunol.,16675322,2006,5
TLR10_BIND	Toll-like receptor signaling.,Akira S.,J Biol Chem.,12893815,2003,10,3
TLR10_BIND	Toll-like receptors and innate immunity.,Uematsu S, Akira S.,J Mol Med.,16924467,2006,9
TLR2/10_BIND	Human TLR10 is a functional receptor, expressed by B cells and plasmacytoid dendritic cells, which activates gene transcription through MyD88.,Hasan U, Chaffois C, Gaillard C, Saulnier V, Merck E, Tancredi S, Guiet C, Brière F, Vlach J, Lebecque S, Trinchieri G, Bates EE.,J Immunol.,15728506,2005,3,1
TLR2/10_BIND	Inferences, questions and possibilities in Toll-like receptor signalling.,Beautler B.,Nature.,15241424,2004,7,8
TLR2/10_BIND	TLR signaling.,Kawai T, Akira S.,Cell Death Differ.,16410796,2006,5
TLR2/10_BIND	Toll-like receptor function and signaling.,Kaisho T, Akira S.,J Allergy Clin Immunol.,16675322,2006,5
TLR2/10_BIND	Toll-like receptor signaling.,Akira S.,J Biol Chem.,12893815,2003,10,3
TLR2/10_BIND	Toll-like receptors and innate immunity.,Uematsu S, Akira S.,J Mol Med.,16924467,2006,9
TLR2/6_BIND	Inferences, questions and possibilities in Toll-like receptor signalling.,Beautler B.,Nature.,15241424,2004,7,8
TLR2/6_BIND	TLR signaling.,Kawai T, Akira S.,Cell Death Differ.,16410796,2006,5
TLR2/6_BIND	Toll-like receptor function and signaling.,Kaisho T, Akira S.,J Allergy Clin Immunol.,16675322,2006,5
TLR2/6_BIND	Toll-like receptor signaling.,Akira S.,J Biol Chem.,12893815,2003,10,3
TLR2/6_BIND	Toll-like receptors and innate immunity.,Uematsu S, Akira S.,J Mol Med.,16924467,2006,9
TLR2/L-D_BIND	Essential roles of CD14 and lipopolysaccharide-binding protein for activation of toll-like receptor (TLR)2 as well as TLR4 Reconstitution of TLR2- and TLR4-activation by distinguishable ligands in LPS preparations.,Muta T, Takeshige K.,Eur J Biochem.,11502220,2001,8
TLR2/L-D_BIND	Inferences, questions and possibilities in Toll-like receptor signalling.,Beautler B.,Nature.,15241424,2004,7,8
TLR2_2PHOS	Toll-like receptor 2-mediated NF-kappa B activation requires a Rac1-dependent pathway.,Arbibe L, Mira JP, Teusch N, Kline L, Guha M, Mackman N, Godowski PJ, Ulevitch RJ, Knaus UG.,Nat Immunol.,11101877,2000,12
TLR2_PI3K1A	Toll-like receptor 2-mediated NF-kappa B activation requires a Rac1-dependent pathway.,Arbibe L, Mira JP, Teusch N, Kline L, Guha M, Mackman N, Godowski PJ, Ulevitch RJ, Knaus UG.,Nat Immunol.,11101877,2000,12

TLR3_2PHOS	Inferences, questions and possibilities in Toll-like receptor signalling.,Beautler B.,Nature.,15241424,2004,7,8
TLR3_2PHOS	TLR signaling.,Kawai T, Akira S.,Cell Death Differ.,16410796,2006,5
TLR3_2PHOS	Toll-like receptor function and signaling.,Kaisho T, Akira S.,J Allergy Clin Immunol.,16675322,2006,5
TLR3_2PHOS	Toll-like receptor signaling.,Akira S.,J Biol Chem.,12893815,2003,10,3
TLR3_2PHOS	Toll-like receptors and innate immunity.,Uematsu S, Akira S.,J Mol Med.,16924467,2006,9
TLR3_BIND	Inferences, questions and possibilities in Toll-like receptor signalling.,Beautler B.,Nature.,15241424,2004,7,8
TLR3_BIND	TLR signaling.,Kawai T, Akira S.,Cell Death Differ.,16410796,2006,5
TLR3_BIND	Toll-like receptor function and signaling.,Kaisho T, Akira S.,J Allergy Clin Immunol.,16675322,2006,5
TLR3_BIND	Toll-like receptor signaling.,Akira S.,J Biol Chem.,12893815,2003,10,3
TLR3_BIND	Toll-like receptors and innate immunity.,Uematsu S, Akira S.,J Mol Med.,16924467,2006,9
TLR3_PI3K1A	Novel roles of TLR3 tyrosine phosphorylation and PI3 kinase in double-stranded RNA signaling.,Sarkar SN, Peters KL, Elco CP, Sakamoto S, Pal S, Sen GC.,Nat Struct Mol Biol.,15502848,2004,11
TLR3_TICAM1	Cooperation of Toll-like receptor signals in innate immune defence.,Trinchieri G, Sher A.,Nat Rev Immunol.,17318230,2007,3
TLR3_TICAM1	TICAM-1, an adaptor molecule that participates in Toll-like receptor 3-mediated interferon-beta induction.,Oshiumi H, Matsumoto M, Funami K, Akazawa T, Seya T.,Nat Immunol.,12539043,2003,2
TLR3_TICAM1	Toll-like receptor function and signaling.,Kaisho T, Akira S.,J Allergy Clin Immunol.,16675322,2006,5
TLR4/L_MD2_BIND	Essential roles of CD14 and lipopolysaccharide-binding protein for activation of toll-like receptor (TLR)2 as well as TLR4 Reconstitution of TLR2- and TLR4-activation by distinguishable ligands in LPS preparations.,Muta T, Takeshige K.,Eur J Biochem.,11502220,2001,8
TLR4/L_MD2_BIND	Inferences, questions and possibilities in Toll-like receptor signalling.,Beautler B.,Nature.,15241424,2004,7,8
TLR4/L_MD2_BIND	Lysines 128 and 132 enable lipopolysaccharide binding to MD-2, leading to Toll-like receptor-4 aggregation and signal transduction.,Visintin A, Latz E, Monks BG, Espevik T, Golenbock DT.,J Biol Chem.,12960171,2003,11,28
TLR4/L_MD2_TRD3A_BIND	Triad3A, an E3 ubiquitin-protein ligase regulating Toll-like receptors.,Chuang TH, Ulevitch RJ.,Nat Immunol.,15107846,2004,5
TLR4/L_MD2_TRD3A_DEGR	Triad3A, an E3 ubiquitin-protein ligase regulating Toll-like receptors.,Chuang TH, Ulevitch RJ.,Nat Immunol.,15107846,2004,5
TLR4_MD2_BIND	Endotoxin recognition and signal transduction by the TLR4/MD2-complex.,Fitzgerald KA, Rowe DC, Golenbock DT.,Microbes Infect.,15596121,2004,12
TLR4_MD2_BIND	Monomeric recombinant MD-2 binds toll-like receptor 4 tightly and confers lipopolysaccharide responsiveness.,Re F, Strominger JL.,J Biol Chem.,11976338,2002,6,28
TLR4_SIGIRR_BIND	SIGIRR, a negative regulator of Toll-like receptor-interleukin 1 receptor signaling.,Wald D, Qin J, Zhao Z, Qian Y, Naramura M, Tian L, Towne J, Sims JE, Stark GR, Li X.,Nat Immunol.,12925853,2003,9
TLR4_SIGIRR_TRAF6_BIND	SIGIRR, a negative regulator of Toll-like receptor-interleukin 1 receptor signaling.,Wald D, Qin J, Zhao Z, Qian Y, Naramura M, Tian L, Towne J, Sims JE, Stark GR, Li X.,Nat Immunol.,12925853,2003,9
TLR5_BIND	TLR signaling.,Kawai T, Akira S.,Cell Death Differ.,16410796,2006,5
TLR5_BIND	TOO LONG: See pubmed ID,Sato S, Sugiyama M, Yamamoto M, Watanabe Y, Kawai T, Takeda K, Akira S.,J Immunol.,14530355,2003,10,15

TLR5_BIND	Toll-like receptor function and signaling.,Kaisho T, Akira S.,J Allergy Clin Immunol.,16675322,2006,5
TLR5_BIND	Toll-like receptor signaling.,Akira S.,J Biol Chem.,12893815,2003,10,3
TLR5_BIND	Toll-like receptors and innate immunity.,Uematsu S, Akira S.,J Mol Med.,16924467,2006,9
TLR5_SIGIRR_BIND	SIGIRR, a negative regulator of Toll-like receptor-interleukin 1 receptor signaling.,Wald D, Qin J, Zhao Z, Qian Y, Naramura M, Tian L, Towne J, Sims JE, Stark GR, Li X.,Nat Immunol.,12925853,2003,9
TLR5_SIGIRR_TRAF6_BIND	SIGIRR, a negative regulator of Toll-like receptor-interleukin 1 receptor signaling.,Wald D, Qin J, Zhao Z, Qian Y, Naramura M, Tian L, Towne J, Sims JE, Stark GR, Li X.,Nat Immunol.,12925853,2003,9
TLR7_BIND	Inferences, questions and possibilities in Toll-like receptor signalling.,Beautler B.,Nature.,15241424,2004,7,8
TLR7_BIND	TLR signaling.,Kawai T, Akira S.,Cell Death Differ.,16410796,2006,5
TLR7_BIND	Toll-like receptor function and signaling.,Kaisho T, Akira S.,J Allergy Clin Immunol.,16675322,2006,5
TLR7_BIND	Toll-like receptor signaling.,Akira S.,J Biol Chem.,12893815,2003,10,3
TLR7_BIND	Toll-like receptors and innate immunity.,Uematsu S, Akira S.,J Mol Med.,16924467,2006,9
TLR8_BIND	Inferences, questions and possibilities in Toll-like receptor signalling.,Beautler B.,Nature.,15241424,2004,7,8
TLR8_BIND	TLR signaling.,Kawai T, Akira S.,Cell Death Differ.,16410796,2006,5
TLR8_BIND	Toll-like receptor function and signaling.,Kaisho T, Akira S.,J Allergy Clin Immunol.,16675322,2006,5
TLR8_BIND	Toll-like receptor signaling.,Akira S.,J Biol Chem.,12893815,2003,10,3
TLR8_BIND	Toll-like receptors and innate immunity.,Uematsu S, Akira S.,J Mol Med.,16924467,2006,9
TLR9/L_TRD3A_BIND	Triad3A, an E3 ubiquitin-protein ligase regulating Toll-like receptors.,Chuang TH, Ulevitch RJ.,Nat Immunol.,15107846,2004,5
TLR9/L_TRD3A_DEGR	Triad3A, an E3 ubiquitin-protein ligase regulating Toll-like receptors.,Chuang TH, Ulevitch RJ.,Nat Immunol.,15107846,2004,5
TLR9_BIND	Inferences, questions and possibilities in Toll-like receptor signalling.,Beautler B.,Nature.,15241424,2004,7,8
TLR9_BIND	Mechanisms of TLR9 activation.,Latz E, Visintin A, Espevik T, Golenbock DT.,J Endotoxin Res.,15588423,2004
TLR9_BIND	TLR signaling.,Kawai T, Akira S.,Cell Death Differ.,16410796,2006,5
TLR9_BIND	TLR9 signals after translocating from the ER to CpG DNA in the lysosome.,Latz E, Schoenemeyer A, Visintin A, Fitzgerald KA, Monks BG, Knetter CF, Lien E, Nilsen NJ, Espevik T, Golenbock DT.,Nat Immunol.,14716310,2004,2
TLR9_BIND	Toll-like receptor function and signaling.,Kaisho T, Akira S.,J Allergy Clin Immunol.,16675322,2006,5
TLR9_BIND	Toll-like receptor signaling.,Akira S.,J Biol Chem.,12893815,2003,10,3
TLR9_BIND	Toll-like receptors and innate immunity.,Uematsu S, Akira S.,J Mol Med.,16924467,2006,9
TLR9_SIGIRR_BIND	SIGIRR, a negative regulator of Toll-like receptor-interleukin 1 receptor signaling.,Wald D, Qin J, Zhao Z, Qian Y, Naramura M, Tian L, Towne J, Sims JE, Stark GR, Li X.,Nat Immunol.,12925853,2003,9
TLR9_SIGIRR_TRAF6_BIND	SIGIRR, a negative regulator of Toll-like receptor-interleukin 1 receptor signaling.,Wald D, Qin J, Zhao Z, Qian Y, Naramura M, Tian L, Towne J, Sims JE, Stark GR, Li X.,Nat Immunol.,12925853,2003,9
TLR9_TRAN	TLR9 signals after translocating from the ER to CpG DNA in the lysosome.,Latz E, Schoenemeyer A, Visintin A, Fitzgerald KA, Monks BG, Knetter CF, Lien E, Nilsen NJ, Espevik T, Golenbock DT.,Nat Immunol.,14716310,2004,2

TOLLIP_DEGR	Negative regulation of toll-like receptor-mediated signaling by Tollip.,Zhang G, Ghosh S.,J Biol Chem.,11751856,2002,3,1
TOLLIP_DEGR2	Negative regulation of toll-like receptor-mediated signaling by Tollip.,Zhang G, Ghosh S.,J Biol Chem.,11751856,2002,3,1
TOLLIP_PHOS	Negative regulation of toll-like receptor-mediated signaling by Tollip.,Zhang G, Ghosh S.,J Biol Chem.,11751856,2002,3,1
TOLLIP_UBIQ	Negative regulation of toll-like receptor-mediated signaling by Tollip.,Zhang G, Ghosh S.,J Biol Chem.,11751856,2002,3,1
TOLLIP_pail345p_hs_BIND	Characterization of Tollip protein upon Lipopolysaccharide challenge.,Li T, Hu J, Li L.,Mol Immunol.,15140579,2004,5
TOLLIP_pail3p_hs_BIND	Characterization of Tollip protein upon Lipopolysaccharide challenge.,Li T, Hu J, Li L.,Mol Immunol.,15140579,2004,5
TRAF6_DIME	Cutting edge: TNFR-associated factor (TRAF) 6 is essential for MyD88-dependent pathway but not toll/IL-1 receptor domain-containing adaptor-inducing IFN-beta (TRIF)-dependent pathway in TLR signaling.,Gohda J, Matsumura T, Inoue J.,J Immunol.,15322147,2004,9,1
TRAF6_DIME	Toll-like receptor signaling.,Akira S.,J Biol Chem.,12893815,2003,10,3
TRAF6_SIGIRR_BIND	SIGIRR, a negative regulator of Toll-like receptor-interleukin 1 receptor signaling.,Wald D, Qin J, Zhao Z, Qian Y, Naramura M, Tian L, Towne J, Sims JE, Stark GR, Li X.,Nat Immunol.,12925853,2003,9
TRX_NADPH_BIND	Reactive oxygen species, antioxidants, and the mammalian thioredoxin system.,Nordberg J, Arnér ES.,Free Radic Biol Med.,11728801,2001,12,1
TRX_NADPH_BIND	Thioredoxin: a multifunctional antioxidant enzyme in kidney, heart and vessels.,Yamawaki H, Berk BC.,Curr Opin Nephrol Hypertens.,15687841,2005,3
TRX_OXID	Reactive oxygen species, antioxidants, and the mammalian thioredoxin system.,Nordberg J, Arnér ES.,Free Radic Biol Med.,11728801,2001,12,1
TRX_REDU	Reactive oxygen species, antioxidants, and the mammalian thioredoxin system.,Nordberg J, Arnér ES.,Free Radic Biol Med.,11728801,2001,12,1
TRX_REDU	Thioredoxin: a multifunctional antioxidant enzyme in kidney, heart and vessels.,Yamawaki H, Berk BC.,Curr Opin Nephrol Hypertens.,15687841,2005,3
ca2_TRAN	Inositol 1,4,5-trisphosphate receptors as signal integrators.,Patterson RL, Boehning D, Snyder SH.,Annu Rev Biochem.,15189149,2004
ca2_TRAN2	Identification of Ser38 as the site in cardiac sarcoplasmic reticulum Ca(2+)-ATPase that is phosphorylated by Ca2+/calmodulin-dependent protein kinase.,Toyofuku T, Curotto Kurzydlowski K, Narayanan N, MacLennan DH.,J Biol Chem.,7929371,1994,10,21
pa_hs_DEPH	Phosphatidate phosphatases of mammals, yeast, and higher plants.,Kocsis MG, Weselake RJ.,Lipids.,8869881,1996,8
pail45p_hs_DISS	Regulation of phosphoinositide-specific phospholipase C isozymes.,Rhee SG, Bae YS.,J Biol Chem.,9182519,1997,6,13
pail45p_hs_DISS	Synthesis and function of 3-phosphorylated inositol lipids.,Vanhaesebroeck B, Leevers SJ, Ahmadi K, Timms J, Katso R, Driscoll PC, Woscholski R, Parker PJ, Waterfield MD.,Annu Rev Biochem.,11395417,2001
pail45p_hs_DISS2	Regulation of phosphoinositide-specific phospholipase C isozymes.,Rhee SG, Bae YS.,J Biol Chem.,9182519,1997,6,13
pail45p_hs_DISS2	Synthesis and function of 3-phosphorylated inositol lipids.,Vanhaesebroeck B, Leevers SJ, Ahmadi K, Timms J, Katso R, Driscoll PC, Woscholski R, Parker PJ, Waterfield MD.,Annu Rev Biochem.,11395417,2001
pail45p_hs_PLCB2_BIND	Regulation of phosphoinositide-specific phospholipase C isozymes.,Rhee SG, Bae YS.,J Biol Chem.,9182519,1997,6,13
pail45p_hs_PLCB2_BIND	Synthesis and function of 3-phosphorylated inositol lipids.,Vanhaesebroeck B, Leevers SJ, Ahmadi K, Timms J, Katso R, Driscoll PC, Woscholski R, Parker PJ, Waterfield MD.,Annu Rev Biochem.,11395417,2001

pail45p_hs_PLCG_BIND	Regulation of phosphoinositide-specific phospholipase C isozymes.,Rhee SG, Bae YS.,J Biol Chem.,9182519,1997,6,13
pail45p_hs_PLCG_BIND	Synthesis and function of 3-phosphorylated inositol lipids.,Vanhaesebroeck B, Leevers SJ, Ahmadi K, Timms J, Katso R, Driscoll PC, Woscholski R, Parker PJ, Waterfield MD.,Annu Rev Biochem.,11395417,2001
pail_hs_PHOS	Phosphoinositide 3-kinases and the regulation of vesicular trafficking.,Backer JM.,Mol Cell Biol Res Commun.,10891392,2000,4
pchol_hs_DISS	Phospholipase D-mediated hydrolysis of phosphatidylcholine: role in cell signalling.,Liscovitch M, Ben-Av P, Danin M, Faiman G, Eldar H, Livneh E.,J Lipid Mediat.,8268464,1993,11
pchol_hs_PLDP_BIND	Phospholipase D-mediated hydrolysis of phosphatidylcholine: role in cell signalling.,Liscovitch M, Ben-Av P, Danin M, Faiman G, Eldar H, Livneh E.,J Lipid Mediat.,8268464,1993,11
